# Supplementary material for: Systematic review of shared decision-making interventions for people living with chronic respiratory diseases
Source: BMJ Open. 2023 May 2;13(5):e069461. doi: 10.1136/bmjopen-2022-069461 (PMC10163462; doi:10.1136/bmjopen-2022-069461)
Supplement: Supplementary data [file bmjopen-2022-069461supp001.pdf]

Supplementary Material

Title:

A systematic review of shared decision-making interventions for people living with chronic respiratory diseases

Authors:

Barradell, AC; Gerlis, C; Houchen-Wolloff L; Bekker, HL; Robertson, N; Singh, SJ.

Supplementary material V1.0

Supplementary Material 1:

MEDLINE search strategy

1. ((joint* or share* or sharing* or informed*) adj3 (decision* or decid*3 or choice*)).mp. [mp=title, abstract, original title, name of substance word, subject heading word, floating sub-heading word, keyword heading word, organism supplementary concept word, protocol supplementary concept word, rare disease supplementary concept word, unique identifier, synonyms]
2. "DECISION MAKING, SHARED"/
3. exp "DECISION MAKING"/
4. "DECISION SUPPORT TECHNIQUES"/
5. DECISION SUPPORT SYSTEMS, CLINICAL/
6. ((decision* or choice*) and (making* or support* or behavior* or behaviour*)).ti,ab.
7. PATIENT PARTICIPATION/
8. ((patient* or consumer*) adj3 (involvement* or involving* or participation* or participating*)).ti,ab.
9. "PATIENT-CENTERED CARE"/
10. "PROFESSIONAL-PATIENT RELATIONS"/
11. "PHYSICIAN-PATIENT RELATIONS"/
12. ((patient* or person* or client* or consumer*) adj3 (centred or centered or focused or oriented)).ti,ab.
13. "decision tool*".ti,ab.
14. "clinical counsel*".ti,ab.
15. "patient decision guide*".ti,ab.
16. "decision coaching".ti,ab.
17. "decision aid*".ti,ab.
18. or/1-17
19. LUNG DISEASES, OBSTRUCTIVE/
20. exp PULMONARY DISEASE, CHRONIC OBSTRUCTIVE/
21. emphysema*.ti,ab.
22. (chronic* adj3 bronchiti*).ti,ab.
23. (obstruct* adj3 (pulmonary or lung* or airway* or airflow* or bronch* or respirat*)).ti,ab.
24. (coad or cobd or copd or aecb).ti,ab.
25. asthma*.ti,ab.
26. exp ASTHMA/
27. (idiopathic and pulmonary and fibrosis).ti,ab.
28. IDIOPATHIC PULMONARY FIBROSIS/
29. (interstitial and lung and disease).ti,ab.
30. LUNG DISEASES, INTERSTITIAL/
31. (extrinsic and allergic and alveolitis).ti,ab.
32. ALVEOLITIS, EXTRINSIC ALLERGIC/
33. (non and specific and interstitial and (pneumonitis or NSIP)).ti,ab.

Supplementary material V1.0

34. (Usual and interstitial and (pneumonia or UIP)).ti,ab.
35. "LUNG DISEASES, OBSTRUCTIVE"/
36. ((lung or pulmonary or airway) adj disease).ti,ab.
37. "cystic fibrosis".ti,ab.
38. CYSTIC FIBROSIS/
39. "SARCOIDOSIS, PULMONARY"/
40. sarcoidosis.ti,ab.
41. exp "LUNG NEOPLASMS"/
42. (lung adj (cancer* or tumo?r* or neoplas*)).ti,ab.
43. "TUBERCULOSIS, PULMONARY"/
44. tuberculosis.ti,ab.
45. BRONCHIECTASIS/
46. bronchiectasis.ti,ab.
47. exp PNEUMOCONIOSIS/
48. (autoimmune adj3 (lung or airway or pulmonary)).ti,ab.
49. or/19-48
50. randomized controlled trial.pt.
51. controlled clinical trial.pt.
52. randomized.ab.
53. placebo.ab.
54. clinical trials as topic.sh.
55. randomly.ab.
56. trial.ti.
57. or/50-56
58. exp ANIMALS/ not humans.sh.
59. 57 not 58
60. 59 and 18 and 49

Supplementary Material 2:

Data items

Extracted items included: population (age, sex, health literacy skill, diagnosis, disease severity, length of diagnosis, baseline measures, total number randomised), intervention (group names, group sample size, adherence to SDM principles, adherence to complex intervention development guidelines, content, training, timing, frequency, duration and any other interventions), comparator (group names, group sample size, content, training, timing, frequency, duration) and outcomes (type, unit of analysis, reported missing data). Data was also extracted on each study's objectives, study design, unit of allocation, decision posed (e.g. medication, pulmonary rehabilitation, surgery, end of life), duration of the study and follow up.

Sub-group analysis

The following additional data was collected for sub-group analysis: diagnosis, characteristics (age, sex, health literacy skill), decision posed (medication, pulmonary rehabilitation, surgery, end of life), intervention dose (number and length of sessions) and length of follow-up.

Supplementary material V1.0

Supplementary Material 3:

Characteristics of excluded studies

Lead Author	Year	Reason for exclusion	Categorised reason for exclusion
Aaron[1]	2006	Abstract only (results in Vandemheen 2009)	Type of publication
Aboumatar[2]	2017	No results in paper (in Aboumatar 2019)	Type of publication
Aboumatar[3]	2019	No SDM within intervention	Type of intervention
Braun[4]	2005	Abstract only (in Menon 2016)	Type of publication
Brown [5]	2012	CRD participant data not distinguishable from analysis	Participant population
Brown [6]	2004	CRD participant data not distinguishable from analysis	Participant population
Brundage[7]	1998	CRD participant data not distinguishable from analysis	Participant population
Butow[8]	2015	No CRD participants	Participant population
Carlucci [9]	2016	No comparator group	Study design
Cook[10]	2016	No adult CRD participants	Participant population
Coultas[11]	2005	No SDM within intervention	Type of intervention
Cvietusa[12]	2020	No SDM within intervention	Type of intervention
Daly[13]	2013	CRD participant data not distinguishable from analysis	Participant population
Detmar[14]	2002	CRD participant data not distinguishable from analysis	Participant population
Dyar[15]	2012	CRD participant data not distinguishable from analysis	Participant population
Early[16]	2015	No SDM within intervention	Type of intervention
Fiks[17]	2015	No CRD participants	Participant population
Fors[18]	2018	CRD participant data not distinguishable from analysis	Participant population
Foster[19]	2016	No SDM within intervention	Type of intervention
Foster[20]	2014	No more than one choice	Type of intervention
Fraenkel[21]	2016	No CRD participants	Participant population

Supplementary material V1.0

Gagne[22]	2016	Abstract only (results in Gagne 2017)	Type of publication
George[23]	2017	Abstract only - (results in George 2020)	Type of publication
George[24]	2020	No more than one choice	Type of intervention
Gupta[25]	2019	No SDM within intervention	Type of intervention
Hagan[26]	2008	No SDM within intervention	Type of intervention
Halley[27]	2015	No CRD participants	Participant population
Hilberink[28]	2011	No SDM within intervention	Type of intervention
Hoerger[29]	2017	CRD participant data not distinguishable from analysis	Participant population
Hollen[30]	2013	No comparator group	Study design
Hoskins[31]	2016	No SDM within intervention	Type of intervention
Houben[32]	2019	No SDM within intervention	Type of intervention
Hyatt[33]	2018	CRD participant data not distinguishable from analysis	Participant population
Izumi[34]	2018	CRD participant data not distinguishable from analysis	Participant population
Jonsdottir[35]	2015	No SDM within intervention	Type of intervention
Kass[36]	2009	CRD participant data not distinguishable from analysis	Participant population
Kennedy[37]	2013	CRD participant data not distinguishable from analysis	Participant population
Koerner[38]	2014	No CRD participants	Participant population
Krug[39]	2021	No SDM within intervention	Type of intervention
Lainscak[40]	2013	No SDM within intervention	Type of intervention
Latimer[41]	2007	CRD participant data not distinguishable from analysis	Participant population
Lavoie[42]	2014	No SDM within intervention	Type of intervention
Leung[43]	2012	Cross-sectional study	Study design
Lim[44]	2021	Hypothetical decision making	Type of intervention
Llanos-Paez[45]	2021	No SDM within intervention	Type of intervention

Supplementary material V1.0

Lopez-Lopez[46]	2020	No SDM within intervention	Type of intervention
Ludden[47]	2018	Adult CRD participant data not distinguishable from analysis	Participant population
Makimoto[48]	2020	Letter to editor	Type of publication
Markman[49]	2005	No comparator group	Study design
Markun[50]	2018	No participant-facing content	Type of intervention
Mayer[51]	2016	CRD participant data not distinguishable from analysis	Participant population
Meline[52]	2021	No CRD participants	Participant population
Melzer[53]	2018	No SDM within intervention	Type of intervention
Menon[54]	2016	CRD participant data not distinguishable from analysis	Participant population
Meropol[55]	2013	CRD participant data not distinguishable from analysis	Participant population
Moullec[56]	2012	No SDM within intervention	Type of intervention
Murray[57]	2010	CRD participant data not distinguishable from analysis	Participant population
Nedjat-Haiem[58]	2017	CRD participant data not distinguishable from analysis	Participant population
Nugent[59]	2018	No comparator group	Study design
Patel[60]	2019	No participant-facing content	Type of intervention
Rabow[61]	2004	CRD participant data not distinguishable from analysis	Participant population
Rea[62]	2004	No SDM within intervention	Type of intervention
Reddel[63]	2019	Abstract only (results not yet published)	Type of publication
Reinke[64]	2017	No SDM outcomes	Outcome measures
Rhee[65]	2008	Participants <18years old	Participant population
Roelands[66]	2004	No CRD participants	Participant population
Rosemann[67]	2013	Abstract only (results in Steurer 2017)	Type of publication
Ruparel[68]	2019	No CRD participants	Participant population
Rutten-vanMolken[69]	2017	Abstract only - (results in Slok 2016)	Type of publication

Supplementary material V1.0

Sands[70]	2014	Abstract only - (results not yet published)	Type of publication
Schofield[71]	2013	No SDM within intervention	Type of intervention
Schubart[72]	2015	CRD participant data not distinguishable from analysis	Participant population
Schumacher[73]	2002	No quantitative data	Study design
Schwarze[74]	2020	CRD participant data not distinguishable from analysis	Participant population
Shade[75]	2019	Adult CRD participant data not distinguishable from analysis	Participant population
Shirai[76]	2012	CRD participant data not distinguishable from analysis	Participant population
Sinclair[77]	2017	No SDM within intervention	Type of intervention
Skorstengaard[78]	2019	CRD participant data not distinguishable from analysis	Participant population
Slok[79]	2016a	Abstract only (results in Slok 2016b)	Type of publication
Slok[80]	2016b	No SDM within intervention	Type of intervention
Smidth[81]	2013	No comparator group	Study design
Steinhauser[82]	2008	CRD participant data not distinguishable from analysis	Participant population
Steurer[83]	2017	Abstract only (results in Steurer 2015)	Type of publication
Steurer-Stey[84]	2015	No SDM within intervention	Type of intervention
Sullivan[85]	2020	Abstract only	Type of publication
Tai-Seale[86]	2016	CRD participant data not distinguishable from analysis	Participant population
Tamblyn[87]	2005	Abstract only (results in Tamblyn 2015)	Type of publication
Tamblyn[88]	2015	No SDM within intervention	Type of intervention
Tang[89]	2008	No comparator group	Study design
Tapp[90]	2017	No SDM outcomes	Outcome measures
Thee[91]	2021	Protocol paper	Type of publication
Thiam[92]	2007	No SDM within intervention	Type of intervention
Totten[93]	2018	Abstract only (results not yet published)	Type of publication

Supplementary material V1.0

Tsao[94]	2021	No SDM within intervention	Type of intervention
Vandemheen[95]	2009	No SDM within intervention	Type of intervention
Vandemheen[95]	2009	Supplement (to be read with Vandemheen 2009)	Type of publication
Wahlberg[96]	2016	CRD participant data not distinguishable from analysis	Participant population
Waller[97]	2012	CRD participant data not distinguishable from analysis	Participant population
Wang[98]	2010	No SDM within intervention	Type of intervention
Weekes[99]	2009	No SDM within intervention	Type of intervention
Williams[100]	2013	CRD participant data not distinguishable from analysis	Participant population
Wilson[101]	2009	Abstract only (results in Wilson 2010)	Type of publication
Wilson[102]	2005	Abstract only (results in Wilson 2010)	Type of publication
Wilson[103]	2005	Abstract only (results in Wilson 2010)	Type of publication
Wood-Baker[104]	2012	No SDM within intervention	Type of intervention
Yoo[105]	2018	No CRD participants	Participant population
Zakrisson[106]	2019	CRD participant data not distinguishable from analysis	Participant population
Zanartu[107]	2017	Letter to editor	Type of publication

Supplementary material V1.0

Supplementary Material 4:

Summary of studies (extended version)

First Author, Year, Country, Sample size, Study design, Population	Outcome(s)	Summary of results	Certainty of evidence (GRADE score)
Au, 2012, USA, Clinicians = 92 Patients = 376, RCT, Outpatients with COPD	*Validated:* Quality of communication (QOC). 19-items scored from 0-100 (higher score=better communication). *Non-validated:* Occurrence of discussions about end of life preferences between patients and either clinician or surrogate. 4-item self-reported questionnaire scored from 0-100% (higher%=greater occurrence).	Baseline quality of communication was poor in both groups. Modest improvements in both groups, but significant mean between group difference favouring the intervention group (5.7 points; p<0.05). The occurrence (%) of patient communication about end of life care was significantly higher in the intervention group for three of four questions (mean(SD)): 1. Int: 35.2(74.6) Con: 15.9(58.2) p<0.05, 2. Int: 60.3(122.9) Con: 30.8(175.5) p<0.05, 3. Int: 53.6(115.1) Con: 45.2(73.3) p>0.05, 4. Int: 86.2(84.6) Con: 75.2(115.0) p<0.05.	Moderate ⊕⊕⊕◯ Moderate certainty because of risk of bias.
Brundage, 2001, Canada, Patients = 20, Feasibility CBA, Outpatients with lung cancer	*Validated:* Decisional conflict (DCS). 16-items using a 4-point Likert scale anchored with strongly agree and strongly disagree. Converted scale scores >37.5 indicate meaningful decisional conflict or delayed decision implementation. *Non-validated:* Knowledge of treatment options. Three open-ended questions. Strength of treatment preference. 1 item using a 7-point Likert scale anchored with definitely wish treatment 1 and definitely wish treatment 2.	Decisional conflict reduced after receiving the intervention in 87% and increased in 13% of participants. Knowledge of the available treatment options improved following the intervention for both 3-year outcome survival[a] and median survival outcome[b]: 1. Knowledge of survival outcomes with treatment 1 alone (mean between group difference 60%[a]; 54%[b]), 2. Knowledge of direction of survival difference between treatments (mean between group difference: 27%[a]; 40%[b]), 3. Knowledge of the magnitude of survival difference between treatments (mean between group difference: 73%[a]; 67%[b]). The number of participants with a treatment preference increased from 80% to 100% following the intervention.	Low ⊕⊕◯◯ Low certainty because of risk of bias and minor publication bias.
Collinsworth, 2018, USA, Patients = 308, RCT, Inpatients with COPD	*Validated:* Patient activation (PAM). 22-items using a 4-point Likert scale anchored with patient is disengaged and overwhelmed and patient has adopted and is maintaining healthy behaviours. Quality of life health status (CAT). 8 item scale with scores ranging from 0-40. Lower scores (<10) indicate COPD	Both groups had a significant improvement in PAM scores following the intervention (mean(SD) difference: 0.52(0.9) and 0.69(1.0) points respectively, p<0.05). Only the intervention group had significantly increased scores in the CAT following the intervention (mean(SD) difference: Int: 5.27(10.3), p<0.05; Con: 0.38(7.8), p>0.05). There were no significant differences in the total number of all-cause or COPD-related readmissions between groups at 1, 2, 3, 6 and 9 months (p>0.05).	Moderate ⊕⊕⊕◯ Moderate certainty because of risk of bias.

Supplementary material V1.0

	causes few problems and higher scores (>30) indicate COPD prevents patients from doing most things they want to do. *Non-validated:* All-cause hospital admissions extracted from electronic health records. Reported as frequency count.		
Gagne, 2017, Canada, Patients = 51, RCT, Outpatients with asthma	*Validated:* Asthma knowledge (QCALF). 37-items with scores ranging from -37 to +37 (higher scores indicate greater knowledge). Decisional conflict (DCS; see above). *Non-validated*: Appropriate use of asthma pharmacotherapy. 4-item face-to-face interviewer-administered questionnaire. The cut off for appropriate use was meeting eleven hierarchical criteria.	Both groups had significantly increased knowledge scores following the intervention (mean(SD) difference: Int: 3.6(8.9)*, p<0.001; Con: 2(7.3)*, p<0.05). There was no significant between group difference (p>0.05). Both groups had significantly reduced decisional conflict scores following the intervention (mean(SD) difference: Int: 8.1(22.5)*, p<0.05; Con: 9.1(27.7)*, p>0.05). There was no significant between group difference (p>0.05). Both groups had modest, but non-significant, within group improvements in their appropriate use of pharmacotherapy (mean(SD) difference: Int: 0.15(0.7)*, p>0.05; Con: 0.16(0.7)*, p>0.05). There was no significant between group difference (mean(SD) between group difference = 0.17(0.7), p>0.05).	Moderate ⊕⊕⊕◯ Moderate certainty because of risk of bias.
Granados-Santiago, 2019, Spain, Patients = 42, RCT, Inpatients with COPD	*Validated:* Quality of life and health status (EQ-5D). 5-items with scores ranging from 0 (defined as the worst imaginable health state) to 100 (defined as the best imaginable health state). COPD knowledge (COPD-Q). 13-items with scores ranging from 0 (defined as lowest knowledge) to 13 (defined as highest knowledge). Medication adherence (TAI).10-items, with higher scores indicating greater adherence. Physical activity (steps/day) measured using the Fitbit Flex 2 Pedometer. Nutritional status (MNA). 18-items with scores lower than 17 considered malnutrition. *Non-validated:* -	Both groups had significant and clinically important improvements in their quality of life and health status at discharge (mean(SD) between group difference: 6.15(27.5)*, p<0.05) but only the intervention group maintained this benefit 3 months later (mean(SD) between group difference: 8.28(50.6)*, p<0.05). Knowledge scores significantly improved in the intervention group alone at discharge (mean(SD) between group difference: 3.89(0.7)*, p<0.05) and maintained at 3 months follow up (mean(SD) between group difference: 3.88(0.8)*, p<0.05). Adherence significantly improved at discharge in the intervention group alone (mean(SD) difference: Int: 3.58(1.5)*, p<0.05; Con: 1.36(1.6)*, p>0.05) but there was no significant between group difference. At 3 months post intervention there was a significant mean(SD) between group difference favouring the intervention group (1.8(2.4)*, p<0.05). Upon discharge, there was a significant reduction in physical activity in both groups (mean(SD) difference: Int: -348.27(2.0)*, p<0.05; Con: -410.05(4.66)*, p<0.05). At 3 months post intervention there was a significant improvement in the intervention group alone (mean(SD) difference: Int: 1371.97(895.2)*, p<0.05; Con: 620.18(12.5)*, p>0.05). At 3 months post intervention there was a significant improvement in nutritional status both within and between groups for the intervention group alone (mean(SD) difference: Int: 4.15(0.1)*, p<0.05; Con: -3.43(0.12)*, p>0.05	Moderate ⊕⊕⊕◯ Moderate certainty because of risk of bias.
Myers, 2021, USA, Patients = 5,	*Validated:* Decisional Conflict (DCS SURE Test). 4-item scale with scores from 0-4, a score <4 indicates clinically significant	Decisional conflict reduced post intervention (mean difference=1). Awareness of treatment options increased from 40% to all 100% participants post intervention. At 30 days post intervention, 80% of participants had made a decision. 40% of participants had	Low ⊕⊕◯◯ Low certainty

Supplementary material V1.0

Pilot CBA, Outpatients with non-small cell lung cancer	decisional conflict. *Non-validated:* Knowledge of treatment options. 3-item survey using a 3-point Likert scale (Yes/No/Unsure) to elicit patient beliefs regarding curability of treatment options, awareness of treatment options, and treatment costs. Awareness of treatment options only item included in this analysis as only item measuring knowledge. Treatment status. 30-day treatment status extracted from electronic health records. This was compared to treatment preferences immediately post intervention.	a treatment that matched their treatment preference.	because of risk of bias.
Walters, 2013, Australia, Patients = 182, Cluster RCT, Outpatients with COPD	*Validated:* Quality of life (SF-36). 8-items with scores from 0-100, higher score indicates better quality of life. Quality of life (SGRQ). 76-items with scores ranging from 0-100, higher score indicates worse quality of life. Patient involvement in healthcare decisions (PIH). 12-items with scores ranging from 0-8 (higher score indicates more involvement). *Non-validated:* Respiratory hospital admissions extracted from electronic health records. Reported as frequency count. Intervention fidelity. Recorded telephone calls from the first three intervention and 10% of control contacts were coded by two raters for content and fidelity to prespecified components of the intervention to assess adherence. To ensure concordance, both raters coded a random sample of 20 calls.	Neither group had a significant change to either their physical[a] or mental[b] health status at 6 months (mean(SD) between group difference: 1.5(0.2)*[a]; 0.8(1.4)*[b], p>0.05) and 12 months (mean(SD) between group difference: 0.0(0.9)*[a]; 0.3(0.9)*[b], p>0.05) post intervention. Neither group had a significant change to their overall quality of life at 6 months (mean(SD) between group difference: 1.9(2.7)*, p>0.05) and 12 months (mean(SD) between group difference: 1.4(1.5)*, p>0.05 post intervention. Only the intervention group had a significant improvement in PIH scores at 6 months (mean(SD) between group difference: 0.4(0.2)*, p<0.05) and 12 months (mean(SD) between group difference: 0.3(0.1)*, p<0.05) post intervention. No significant change in the number of hospital admissions for either group over the 12 months study period (χ^2=2.61, p>0.05). Concordance between two raters was high (overall weighted κ 0.72). Fidelity assessments confirmed specific health mentoring components were addressed with some clarity: 1. COPD symptom management, 49%, 2. Unhelpful self-talk explored and identified, 37%, 3. Unhelpful self-talk challenged, and new self-talk developed, 35%, 4. Action plan for achieving goals made, 54%, 5. Problems and barriers to achieving goals identified and clarified, 46%, 6. Positive changes in behaviour praised, 83%. Raters concluded there was a lower delivery of cognitive behavioural components of intervention.	Very low ⊕◯◯◯ Very low certainty because of significant risk of bias and some inconsistency.
Wilson, 2010, Hawaii, Patients = 612, RCT, Outpatients	*Validated:* Quality of life (MiniAQLQ). 5-items with Likert scale ranging from 0 (all of the time) to 7 (none of the time). *Non-validated:* Healthcare use extracted from electronic health records. Reported as frequency count. Patients' perceived role in treatment decision.	Only intervention groups had a significant improvement in quality of life scores at 12 months post intervention (SDM to control mean(SD) between group difference = 0.39(1.0)*, p<0.05; SDM–clinician decision-making mean(SD) between group difference = 0.11(1.1)*, p>0.05; clinician decision-making-control mean(SD) between group difference = 0.28(1.0)*, p<0.05. Only intervention groups had a significantly reduced healthcare utilisation (SDM-control mean(SD) between group difference =20.36(-1.5)*, p<0.05; SDM-	Moderate ⊕⊕⊕◯ Moderate certainty because of risk of bias.

Supplementary material V1.0

with asthma	1-item using a 5-point Likert scale ranging from 0 (low involvement in decision-making) to 5 (high involvement in treatment decision-making). Medication adherence. Data extracted from pharmacy records to calculate a continuous medication acquisition index for each year. Intervention fidelity. Audiotapes of both intervention groups of 10% of patients were scored on a detailed performance checklist to determine whether the two protocols were delivered as intended. Scores ranged from 0-4 with higher scores indicated greater adherence.	clinician decision-making mean(SD) between group difference =0.01(2.7)*, p>0.05; clinician decision-making-control mean(SD) between group difference= 20.37(-1.5*), p<0.05. The SDM group rated their influence on the treatment decision as being approximately the same as the clinicians' influence (mean(SD)= 3.1(0.6). The SDM group ratings were significantly different from the clinician decision-making group, with the latter feeling that their clinician had a greater influence (mean(SD)=2.5(0.9), p<0.05) than they did themselves. Medication adherence significantly increased only in intervention groups (SDM-control mean(SD) between group difference=0.21(0.5)*, p<0.05; SDM–clinician decision-making mean(SD) between group difference= 0.08(0.4)*, p>0.05; CDM-control mean(SD) between group difference= 0.13(0.4)*, p<0.05. This was not sustained at 2 years (SDM-control mean(SD) between group difference= 0.03(0.4)*, p>0.05; SDM-clinician decision-making mean(SD) between group difference= 0.04(0.4)*, p>0.05; clinician decision-making-control mean(SD) between group difference=-0.01(-0.4)*, p>0.05. Protocol adherence scores assigned by the raters were high (SDM=4.0, clinician decision-making=3.9) and did not differ significantly between the two groups (p>0.05).

Supplementary material V1.0

Supplementary Material 5:

5a: Sub-group analysis

Despite the broad inclusion criteria for respiratory conditions, studies only included those with COPD, lung cancer and asthma (Table below). Participants diagnosed with lung cancer and COPD were older than those diagnosed with asthma. The split between male and female participants were even across studies apart from in one study where nearly all were male[108] and another where three quarters were female[109]. No specific health literacy outcome measures were used across studies. Baseline educational attainment was reported across all but two studies[110,111]. Across other studies, the percentage of participants who completed higher education varied between 17-59.2%.

SDM interventions for COPD participants aimed to facilitate decisions regarding end-of-life care and positive self-management. Those for lung cancer and asthma participants aimed to facilitate treatment decision-making. Four studies reported a single session SDM intervention[108,109,112,113]. Three studies reported multiple sessions of SDM delivered face to face and with follow up telephone calls[110,114,115]. One study did not report an intervention dose and instead stated the intervention was integrated into routine inpatient care[111]. The length of study follow-up varied from immediately post decision-making to 12 months later.

Barradell AC, *et al. BMJ Open* 2023; 13:e069461. doi: 10.1136/bmjopen-2022-069461

5b: Sub-group analysis of CRD patients from included studies

First Author (Year)	CRD diagnosis	Age (mean (SD))	Gender (% male)	Measure of health literacy (% achievement)	Decision posed	Intervention dose	Length of follow up
Au (2012)	COPD	IG: 69.4(10.0) years	IG: 97.9%	IG: Higher education = 58.3%	IG: End of life care	IG: Single session	IG: 2 weeks
		CG: 69.4(10.0) years	CG: 96.2%	CG: Higher education = 59.2%	CG: End of life care	CG: N/A	CG: 2 weeks
Brundage (2001)	Lung cancer	IG: 69.2(8.1) years	IG: 55%	IG: <High school = 5% High school education = 50% Higher education = 45%	IG: Lung cancer treatments	IG: Single session	IG: Immediately post intervention
Collinsworth (2018)	COPD	IG: 70.0(11.9)	IG: 39.7%	IG: Not disclosed	IG: Self-management plans	IG: Single session and 4 telephone calls	IG: 6 months
		CG: 70.9(12.5)	CG: 43.1%	CG: Not disclosed	CG: Self-management plans	CG: N/A	CG: 6months
Gagne (2017)	Asthma	IG: 46(13.0)	IG: 26.9%	IG: High school education = 57.7% Higher education = 42.3%	IG: Asthma treatments	IG: Single session	IG: 2months
		CG: 41(13.0)	CG: 48%	CG: High school education = 52.0% Higher education = 48.0%	CG: Asthma treatments	CG: N/A	CG: 2months
Granados-Santiago (2019)	COPD	IG: 69.3(9.9)	IG: Not disclosed	IG: Not disclosed	IG: Self-management plans	IG: Integrated into inpatient care	IG: 3 months
		CG: 74.20(9.25)	CG: Not disclosed	CG: Not disclosed	CG: Self-management plans	CG: N/A	CG: 3 months
Myers (2021)	Lung cancer	IG: Not reported	IG: 40%	IG: <High school education = 0% High school education = 40%	IG: Lung cancer treatments	IG: Single session	IG: 30 days

Supplementary material V1.0

				Higher education = 60%			
Walters (2013)	COPD	IG: 68.2(7.9)	IG: 54.4%	IG:<br><High school education = 68%<br>High school education = 13%<br>Higher education = 20%	IG: Self-management plans	IG: Single session plus multiple (x16) telephone calls	IG: 12 months
		CG: 67.3(7.6)	CG: 51.1%	CG:<br><High school education = 75%<br>High school education = 8%<br>Higher education = 17%	CG: Self-management plans	CG: Single session plus multiple (x12) telephone calls	CG: 12 months
Wilson (2010)	Asthma	IG: 45.7(13.3)	IG: 43.6%	IG:<br><High school education = 2.9%<br>High school education = 55.9%<br>Higher education = 41.1%	IG: Asthma treatments	IG: Multiple sessions (x2) plus multiple telephone calls (x3)	IG: 12 months
		IG2: 46.9(12.1)	IG2: 44.1%	IG2:<br><High school education = 0.98%<br>High school education = 64.7%<br>Higher education = 33.8%	IG2: Asthma treatments	IG2: Multiple sessions (x2) plus multiple telephone calls (x3)	IG2: 9 months
		CG: 45.1(12.4)	CG: 42.6%	CG:<br><High school education= 2.9%<br>High school education = 56.9%<br>Higher education = 40.2%	CG: Asthma treatments	CG: Multiple sessions (x2) plus multiple telephone calls (x3)	CG: 9 months

IG = Intervention group; CG = Control group; IG2 = Intervention group 2 (Clinician Decision-Making)

Supplementary material V1.0

References

E1. Aaron SD. Evaluation of a Decision Aid for Adult Cystic Fibrosis Patients Considering Bilateral Lung Transplantation. *Clin Regist Clin trials*. 2006.

2. Aboumatar H, Naqibuddin M, Chung S, et al. Better Respiratory Education and Treatment Help Empower (BREATHE) study: Methodology and baseline characteristics of a randomized controlled trial testing a transitional care program to improve patient-centered care delivery among chronic obstructive pulm. *Contemp Clin Trials*. 2017;62(June):159-167. doi:10.1016/j.cct.2017.08.018

3. Aboumatar H, Naqibuddin M, Chung S, et al. Effect of a Hospital-Initiated Program Combining Transitional Care and Long-term Self-management Support on Outcomes of Patients Hospitalized with Chronic Obstructive Pulmonary Disease: A Randomized Clinical Trial. *JAMA - J Am Med Assoc*. 2019;322(14):1371-1380. doi:10.1001/jama.2019.11982

4. Braun U. A Culturally Sensitive Values-Guided Aid for End of Life Decision-Making. *Clin Regist Clin Trials*. 2005. doi:10.1002/CENTRAL/CN-01510546

5. Brown RF, Bylund CL, Li Y, Edgerson S, Butow P. Testing the utility of a cancer clinical trial specific Question Prompt List (QPL-CT) during oncology consultations. *Patient Educ Couns*. 2012;88(2):311-317. doi:10.1016/j.pec.2012.02.009

6. Brown RF, Butow PN, Sharrock MA, et al. Education and role modelling for clinical decisions with female cancer patients. *Heal Expect*. 2004;7(4):303-316. doi:10.1111/j.1369-7625.2004.00294.x

7. Brundage MD, Davidson JR, Mackillop WJ, Feldman-Stewart DEB, Groome P. Using a treatment-tradeoff method to elicit preferences for the treatment of locally advanced non-small-cell lung cancer. *Med Decis Mak*. 1998;18(3):256-267. doi:10.1177/0272989X9801800302

8. Butow P, Brown R, Aldridge J, et al. Can consultation skills training change doctors' behaviour to increase involvement of patients in making decisions about standard treatment and clinical trials: A randomized controlled trial. *Heal Expect*. 2015;18(6):2570-2583. doi:10.1111/hex.12229

9. Carlucci A, Vitacca M, Malovini A, et al. End-of-Life Discussion, Patient Understanding and Determinants of Preferences in Very Severe COPD Patients: A Multicentric Study. *COPD J Chronic Obstr Pulm Dis*. 2016;13(5):632-638. doi:10.3109/15412555.2016.1154034

Supplementary material V1.0

10. Cook KA, Modena BD, Simon RA. Improvement in Asthma Control Using a Minimally Burdensome and Proactive Smartphone Application. *J Allergy Clin Immunol Pract*. 2016;4(4):730-737.e1. doi:10.1016/j.jaip.2016.03.005

11. Coultas D, Frederick J, Barnett B, Singh G, Wludyka P. A randomized trial of two types of nurse-assisted home care for patients with COPD. *Chest*. 2005;128(4):2017-2024. doi:10.1378/chest.128.4.2017

12. Cvietusa PJ, Wagner NM, Shoup JA, et al. Digital Communication Technology: Does Offering a Choice of Modality Improve Medication Adherence and Outcomes in a Persistent Asthma Population? *Perm J*. 2020;25:1. doi:10.7812/TPP/20.189

13. Daly BJ, Douglas SL, Gunzler D, Lipson AR. Clinical trial of a supportive care team for patients with advanced cancer. *J Pain Symptom Manage*. 2013;46(6):775-784. doi:10.1016/j.jpainsymman.2012.12.008

14. Detmar SB, Muller MJ, Schornagel JH, Wever LDV, Aaronson NK. Health-related quality-of-life assessments and patient-physician communication: A randomized controlled trial. *J Am Med Assoc*. 2002;288(23):3027-3034. doi:10.1001/jama.288.23.3027

15. Dyar S, Lesperance M, Shannon R, Sloan J, Colon-Otero G. A nurse practitioner directed intervention improves the quality of life of patients with metastatic cancer: Results of a randomized pilot study. *J Palliat Med*. 2012;15(8):890-895. doi:10.1089/jpm.2012.0014

16. Early F, Everden AJT, OBrien CM, Fagan PL, Fuld JP. Patient agenda setting in respiratory outpatients. *Chron Respir Dis*. 2015;12(4):347-356. doi:10.1177/1479972315598696

17. Fiks AG, Mayne SL, Karavite DJ, et al. Parent-reported outcomes of a shared decision-making portal in asthma: A practice-based RCT. *Pediatrics*. 2015;135(4):e965-e973. doi:10.1542/peds.2014-3167

18. Fors A, Blanck E, Ali L, et al. Effects of a person-centred telephone-support in patients with chronic obstructive pulmonary disease and/or chronic heart failure – A randomized controlled trial. *PLoS One*. 2018;13(8). doi:10.1371/journal.pone.0203031

19. Foster JM, Smith L, Usherwood T, Sawyer SM, Reddel HK. General practitioner-delivered adherence counseling in asthma: Feasibility and usefulness of skills, training and support tools. *J Asthma*. 2016;53(3):311-320. doi:10.3109/02770903.2015.1091473

20. Foster JM, Usherwood T, Smith L, et al. Inhaler reminders improve adherence with controller treatment in primary care patients with asthma. *J Allergy Clin Immunol*. 2014;134(6):1260-

1268.e3. doi:10.1016/j.jaci.2014.05.041

21. Fraenkel L, Peters E, Tyra S, Oelberg D. Shared Medical Decision Making in Lung Cancer Screening: Experienced versus Descriptive Risk Formats. *Med Decis Mak*. 2016;36(4):518-525. doi:10.1177/0272989X15611083

22. Gagné M, Légaré F, Moisan J, Boulet L. Adding A Decision Aid to Asthma Education: Impact on Decisional Conflict and Appropriate Medication Usage. *Value Heal*. 2016;19(7):A557. doi:10.1016/j.jval.2016.09.1216

23. George M. BREATHE asthma intervention trial. *Clin Regist Clin Trials*. 2017. doi:10.1002/CENTRAL/CN-01380211

24. George M, Bruzzese JM, S. Sommers M, et al. Group-randomized trial of tailored brief shared decision-making to improve asthma control in urban black adults. *J Adv Nurs*. 2020;(September):1-17. doi:10.1111/jan.14646

25. Gupta S, Price C, Agarwal G, et al. *The Electronic Asthma Management System (EAMS) Improves Primary Care Asthma Management*. Vol 53.; 2019. doi:10.1183/13993003.02241-2018

26. Hagan L, Valois P, Patenaude H, Boutin H, Boulet LP, Lafrenière F. Asthma counselling targeted to removal of domestic animals. *Can Respir J*. 2008;15(1):33-38. doi:10.1155/2008/247027

27. Halley MC, Rendle KAS, Gillespie KA, Stanley KM, Frosch DL. An exploratory mixed-methods crossover study comparing DVD- vs. Web-based patient decision support in three conditions: The importance of patient perspectives. *Heal Expect*. 2015;18(6):2880-2891. doi:10.1111/hex.12273

28. Hilberink SR, Jacobs JE, Breteler MHM, de Vries H, Grol RPTM. General practice counseling for patients with chronic obstructive pulmonary disease to quit smoking: Impact after 1year of two complex interventions. *Patient Educ Couns*. 2011;83(1):120-124. doi:10.1016/j.pec.2010.04.009

29. Hoerger M, Perry LM, Gramling R, Epstein RM, Duberstein PR. Does educating patients about the early palliative care study increase preferences for outpatient palliative cancer care? Findings from project EMPOWER. *Heal Psychol*. 2017;36(6):538-548. doi:10.1037/hea0000489

30. Hollen PJ, Gralla RJ, Jones RA, et al. A theory-based decision aid for patients with cancer:

Results of feasibility and acceptability testing of DecisionKEYS for cancer. *Support Care Cancer*. 2013;21(3):889-899. doi:10.1007/s00520-012-1603-8

31. Hoskins G, Williams B, Abhyankar P, et al. Achieving Good Outcomes for Asthma Living (GOAL): Mixed methods feasibility and pilot cluster randomised controlled trial of a practical intervention for eliciting, setting and achieving goals for adults with asthma. *Trials*. 2016;17(1):1-17. doi:10.1186/s13063-016-1684-7

32. Houben CHM, Spruit MA, Luyten H, et al. Cluster-randomised trial of a nurse-led advance care planning session in patients with COPD and their loved ones. *Thorax*. 2019;74(4):328-336. doi:10.1136/thoraxjnl-2018-211943

33. Hyatt A, Lipson-Smith R, Gough K, et al. Culturally and linguistically diverse oncology patients' perspectives of consultation audio-recordings and question prompt lists. *Psychooncology*. 2018;27(9):2180-2188. doi:10.1002/pon.4789

34. Izumi SS, Basin B, Presley M, et al. Feasibility and Acceptability of Nurse-Led Primary Palliative Care for Older Adults with Chronic Conditions: A Pilot Study. *J Palliat Med*. 2018;21(8):1114-1121. doi:10.1089/jpm.2017.0666

35. Jonsdottir H, Amundadottir OR, Gudmundsson G, et al. Effectiveness of a partnership-based self-management programme for patients with mild and moderate chronic obstructive pulmonary disease: A pragmatic randomized controlled trial. *J Adv Nurs*. 2015;71(11):2634-2649. doi:10.1111/jan.12728

36. Kass NE, Sugarman J, Medley AM, et al. An intervention to improve cancer patients' understanding of early-phase clinical trials. *IRB Ethics Hum Res*. 2009;31(3):1-8.

37. Kennedy A, Bower P, Reeves D, et al. Implementation of self management support for long term conditions in routine primary care settings: Cluster randomised controlled trial. *BMJ*. 2013;346(7913):1-11. doi:10.1136/bmj.f2882

38. Koerner M, Wirtz M, Michaelis M, et al. A multicentre cluster-randomized controlled study to evaluate a train-the-trainer programme for implementing internal and external participation in medical rehabilitation. *Clin Rehabil*. 2014;28(1):20-35. doi:10.1177/0269215513494874

39. Krug K, Bossert J, Deis N, et al. Effects of an Interprofessional Communication Approach on Support Needs, Quality of Life, and Mood of Patients with Advanced Lung Cancer: A Randomized Trial. *Oncologist*. 2021;26(8):e1445-e1459. doi:10.1002/ONCO.13790

40. Lainscak M, Kadivec S, Kosnik M, et al. Discharge coordinator intervention prevents

hospitalizations in patients with COPD: A randomized controlled trial. *J Am Med Dir Assoc*. 2013;14(6):450.e1-450.e6. doi:10.1016/j.jamda.2013.03.003

41. Latimer AE, Williams-Piehota P, Cox A, Katulak NA, Salovey P, Mowad L. Encouraging cancer patients to talk to their physicians about clinical trials: Considering patients' information needs. *J Appl Biobehav Res*. 2007;12(3-4):178-195. doi:10.1111/j.1751-9861.2008.00020.x

42. Lavoie KL, Moullec G, Lemiere C, et al. Efficacy of brief motivational interviewing to improve adherence to inhaled corticosteroids among adult asthmatics: Results from a randomized controlled pilot feasibility trial. *Patient Prefer Adherence*. 2014;8:1555-1569. doi:10.2147/PPA.S66966

43. Leung JM, Udris EM, Uman J, Au DH. The effect of end-of-life discussions on perceived quality of care and health status among patients with COPD. *Chest*. 2012;142(1):128-133. doi:10.1378/chest.11-2222

44. Lim RK, Semitala FC, Atuhumuza E, et al. Patient choice improves self-efficacy and intention to complete tuberculosis preventive therapy in a routine HIV program setting in Uganda. *PLoS One*. 2021;16(2):e0246113. doi:10.1371/JOURNAL.PONE.0246113

45. Llanos-Paez C, Ambery C, Yang S, et al. Improved Decision-Making Confidence Using Item-Based Pharmacometric Model: Illustration with a Phase II Placebo-Controlled Trial. *AAPS J*. 2021;23(4). doi:10.1208/s12248-021-00600-1

46. Lopez-Lopez L, Valenza MC, Rodriguez-Torres J, Torres-Sanchez I, Granados-Santiago M, Valenza-Demet G. Results on health-related quality of life and functionality of a patient-centered self-management program in hospitalized COPD: a randomized control trial. *Disabil Rehabil*. 2020;42(25):3687-3695. doi:10.1080/09638288.2019.1609099

47. Ludden T, Shade L, Reeves K, et al. Asthma dissemination around patient-centered treatments in North Carolina (ADAPT-NC): a cluster randomized control trial evaluating dissemination of an evidence-based shared decision-making intervention for asthma management. *J Asthma*. September 2018:1-12. doi:10.1080/02770903.2018.1514630

48. Makimoto G, Hotta K, Oze I, et al. Patients' preferences and perceptions of lung cancer treatment decision making: results from Okayama lung cancer study group trial 1406. *Acta Oncol (Madr)*. 2020;59(3):324-328. doi:10.1080/0284186X.2019.1679880

49. Markman M, Petersen J, Montgomery R. An examination of characteristics of lung and colon cancer patients participating in a web-based decision support program: Internet-based

decision support programs. *Oncology*. 2005;69(4):311-316. doi:10.1159/000089763

50. Markun S, Rosemann T, Dalla-Lana K, Steurer-Stey C. Care in Chronic Obstructive Lung Disease (CAROL): A randomised trial in general practice. *Eur Respir J*. 2018;51(5). doi:10.1183/13993003.01873-2017

51. Mayer DK, Deal AM, Crane JM, et al. Using survivorship care plans to enhance communication and cancer care coordination: Results of a pilot study. *Oncol Nurs Forum*. 2016;43(5):636-645. doi:10.1188/16.ONF.636-645

52. Meline J, Prigge JM, Dye D, et al. Adapting the design of a Web-based decision support clinical trial during the COVID-19 pandemic. *Trials*. 2021;22(1):1-6. doi:10.1186/S13063-021-05700-Z/PEER-REVIEW

53. Melzer AC, Clothier BA, Japuntich SJ, et al. Comparative effectiveness of proactive tobacco treatment among smokers with and without chronic lower respiratory disease. *Ann Am Thorac Soc*. 2018;15(3):341-347. doi:10.1513/AnnalsATS.201707-582OC

54. Menon S, Mccullough LB, Beyth RJ, Ford ME, Espadas D, Braun UK. Use of a values inventory as a discussion aid about end-of-life care: A pilot randomized controlled trial. *Palliat Support Care*. 2016;14(4):330-340. doi:10.1017/S1478951515001091

55. Meropol NJ, Egleston BL, Buzaglo JS, et al. A Web-based communication aid for patients with cancer: The CONNECT Study. *Cancer*. 2013;119(7):1437-1445. doi:10.1002/cncr.27874

56. Moullec G, Lavoie KL, Rabhi K, Julien M, Favreau H, Labrecque M. Effect of an integrated care programme on re-hospitalization of patients with chronic obstructive pulmonary disease. *Respirology*. 2012;17(4):707-714. doi:10.1111/j.1440-1843.2012.02168.x

57. Murray MA, Stacey D, Wilson KG, O'Connor AM. Skills training to support patients considering place of end-of-life care: A randomized control trial. *J Palliat Care*. 2010;26(2):112-121. doi:10.1177/082585971002600207

58. Nedjat-Haiem FR, Carrion I V., Gonzalez K, et al. Implementing an Advance Care Planning Intervention in Community Settings with Older Latinos: A Feasibility Study. *J Palliat Med*. 2017;20(9):984-993. doi:10.1089/jpm.2016.0504

59. Nugent SM, Golden SE, Thomas CR, et al. Patient-clinician communication among patients with stage I lung cancer. *Support Care Cancer*. 2018;26(5):1625-1633. doi:10.1007/s00520-017-3992-1

60. Patel MR, Smith A, Leo H, Hao W, Zheng K. Improving Patient–Provider Communication and Therapeutic Practice Through Better Integration of Electronic Health Records in the Exam Room: A Pilot Study. *Heal Educ Behav*. 2019;46(3):484-493. doi:10.1177/1090198118796879

61. Rabow MW, Dibble SL, Pantilat SZ, McPhee SJ. The Comprehensive Care Team. *Arch Intern Med*. 2004;164(1):83. doi:10.1001/archinte.164.1.83

62. Rea H, McAuley S, Stewart A, Lamont C, Roseman P, Didsbury P. A chronic disease management programme can reduce days in hospital for patients with chronic obstructive pulmonary disease. *Intern Med J*. 2004;34(11):608-614. doi:10.1111/j.1445-5994.2004.00672.x

63. Reddel HK, Correll J, Foster J, Karlsson N, Martin M, Keen C. Patient Decision-Making Around Use of Reliever Inhalers in Mild Asthma. In: *American Thoracic Society International Conference Meetings Abstracts*. American Thoracic Society; 2019:A5935-A5935. doi:10.1164/ajrccm-conference.2019.199.1_meetingabstracts.a5935

64. Reinke LF, Feemster LC, McDowell J, et al. The long term impact of an end-of-life communication intervention among veterans with COPD. *Hear Lung J Acute Crit Care*. 2017;46(1):30-34. doi:10.1016/j.hrtlng.2016.10.003

65. Rhee H, Hollen PJ, Belyea MJ, Sutherland MA. Decision-Making Program for Rural Adolescents With Asthma: A Pilot Study. *J Pediatr Nurs*. 2008;23(6):439-450. doi:10.1016/j.pedn.2008.01.079

66. Roelands M, Van Oost P, Stevens V, Depoorter AM, Buysse A. Clinical practice guidelines to improve shared decision-making about assistive device use in home care: A pilot intervention study. *Patient Educ Couns*. 2004;55(2):252-264. doi:10.1016/j.pec.2003.10.002

67. Rosemann T. The Improving Care in Chronic Obstructive Lung Disease Study A Cluster Randomized Trial. *https://clinicaltrials.gov/show/NCT01921556*. 2013. doi:10.1002/CENTRAL/CN-02032168

68. Ruparel M, Quaife SL, Ghimire B, et al. Impact of a lung cancer screening information film on informed decision-making: A randomized trial. *Ann Am Thorac Soc*. 2019;16(6):744-751. doi:10.1513/AnnalsATS.201811-841OC

69. Rutten-vanMolken M, Goossens L, Boland M, et al. The Assessment of Burden of COPD (ABC) tool: a shared decision-making instrument that is predictive of healthcare costs. *Int J Integr Care*. 2017;17(5):320. doi:10.5334/ijic.3637

Supplementary material V1.0

70. Sands M. A Randomised Control Trial for Advance Care Planning and Symptom Management for patients identified in the emergency department and followed up at home. *Clin Regist Clin Trials*. 2014. doi:10.1002/CENTRAL/CN-01881924

71. Schofield P, Ugalde A, Gough K, et al. A tailored, supportive care intervention using systematic assessment designed for people with inoperable lung cancer: A randomised controlled trial. *Psychooncology*. 2013;22(11):2445-2453. doi:10.1002/pon.3306

72. Schubart JR, Green MJ, Van Scoy LJ, et al. Advanced Cancer and End-of-Life Preferences: Curative Intent Surgery Versus Noncurative Intent Treatment. *J Palliat Med*. 2015;18(12):1015-1018. doi:10.1089/jpm.2015.0021

73. Schumacher KL, Koresawa S, West C, et al. Putting cancer pain management regimens into practice at home. *J Pain Symptom Manage*. 2002;23(5):369-382. doi:10.1016/S0885-3924(02)00385-8

74. Schwarze ML, Buffington A, Tucholka JL, et al. Effectiveness of a Question Prompt List Intervention for Older Patients Considering Major Surgery: A Multisite Randomized Clinical Trial. *JAMA Surg*. 2020;155(1):6-13. doi:10.1001/jamasurg.2019.3778

75. Ludden T, Shade L, Reeves K, et al. Asthma dissemination around patient-centered treatments in North Carolina (ADAPT-NC): a cluster randomized control trial evaluating dissemination of an evidence-based shared decision-making intervention for asthma management. *J Asthma*. 2019;56(10):1087-1098. doi:10.1080/02770903.2018.1514630

76. Shirai Y, Fujimori M, Ogawa A, et al. Patients' perception of the usefulness of a question prompt sheet for advanced cancer patients when deciding the initial treatment: A randomized, controlled trial. *Psychooncology*. 2012;21(7):706-713. doi:10.1002/pon.1955

77. Sinclair C, Auret KA, Evans SF, et al. Advance care planning uptake among patients with severe lung disease: A randomised patient preference trial of a nurse-led, facilitated advance care planning intervention. *BMJ Open*. 2017;7(2):1-11. doi:10.1136/bmjopen-2016-013415

78. Skorstengaard MH, Brogaard T, Bonde Jensen A, et al. Advance care planning for patients and their relatives. *Int J Palliat Nurs*. 2019;25(3):112-127. doi:10.12968/ijpn.2019.25.3.112

79. Slok A, Kotz D, van Breukelen G, et al. The assessment of burden of COPD tool improves health related quality of life. In: *European Respiratory Journal*. Vol 48. European Respiratory Society (ERS); 2016:OA1997. doi:10.1183/13993003.congress-2016.oa1997

80. Slok AHM, Kotz D, Van Breukelen G, et al. Effectiveness of the Assessment of Burden of COPD

(ABC) tool on health-related quality of life in patients with COPD: A cluster randomised controlled trial in primary and hospital care. *BMJ Open*. 2016;6(7). doi:10.1136/bmjopen-2016-011519

81. Smidth M, Olesen F, Fenger-Grøn M, Vedsted P. Patient-experienced effect of an active implementation of a disease management programme for COPD - A randomised trial. *BMC Fam Pract*. 2013;14(1):1. doi:10.1186/1471-2296-14-147

82. Steinhauser KE, Alexander SC, Byock IR, George LK, Olsen MK, Tulsky JA. Do preparation and life completion discussions improve functioning and quality of life in seriously ill patients? Pilot randomized control trial. *J Palliat Med*. 2008;11(9):1234-1240. doi:10.1089/jpm.2008.0078

83. Steurer C, Markun S, Rosemann T, Dalla Lana K. Improving Care in Chronic Obstructive Lung Disease (CAROL): a cluster randomized trial in primary care. In: European Respiratory Society (ERS); 2017:OA2915. doi:10.1183/1393003.congress-2017.oa2915

84. Steurer-Stey C, Storch M, Benz S, et al. Motivational training improves self-efficacy but not short-term adherence with asthma self-management: a randomized controlled trial. *Prim Health Care Res Dev*. 2015;16(1):32-41. doi:10.1017/S1463423613000480

85. Sullivan DR, Slatore CG, Stone K, et al. Associations Between Treatment and Team Composition with Decisional Regret Among Patients with Early Stage Lung Cancer. May 2020:A4726-A4726. doi:10.1164/AJRCCM-CONFERENCE.2020.201.1_MEETINGABSTRACTS.A4726

86. Tai-Seale M, Elwyn G, Wilson CJ, et al. Enhancing shared decision making through carefully designed interventions that target patient and provider behavior. *Health Aff*. 2016;35(4):605-612. doi:10.1377/hlthaff.2015.1398

87. Tamblyn R. Computer-Based Decision Support in Managing Asthma in Primary Care. *Clin Regist Clin Trials*. 2005. doi:10.1002/CENTRAL/CN-02024751

88. Tamblyn R, Ernst P, Winslade N, et al. Evaluating the impact of an integrated computer-based decision support with person-centered analytics for the management of asthma in primary care: A randomized controlled trial. *J Am Med Informatics Assoc*. 2015;22(4):773-783. doi:10.1093/jamia/ocu009

89. Tang JI, Shakespeare TP, Lu JJ, et al. Patients' preference for radiotherapy fractionation schedule in the palliation of symptomatic unresectable lung cancer. *J Med Imaging Radiat*

Oncol. 2008;52(5):497-502. doi:10.1111/j.1440-1673.2008.02002.x

90. Tapp H, Shade L, Mahabaleshwarkar R, Taylor YJ, Ludden T, Dulin MF. Results from a pragmatic prospective cohort study: Shared decision making improves outcomes for children with asthma. *J Asthma*. 2017;54(4):392-402. doi:10.1080/02770903.2016.1227333

91. Thee S, Stahl M, Fischer R, et al. A multi-centre, randomized, controlled trial on coaching and telemonitoring in patients with cystic fibrosis: conneCT CF. *BMC Pulm Med*. 2021;21(1):1-11. doi:10.1186/S12890-021-01500-Y/TABLES/1

92. Thiam S, LeFevre AM, Hane F, et al. Effectiveness of a strategy to improve adherence to tuberculosis treatment in a resource-poor setting: A cluster randomized controlled trial. *J Am Med Assoc*. 2007;297(4):380-386. doi:10.1001/jama.297.4.380

93. Totten A. Team-based Versus Primary Care Clinician-led Advance Care Planning in Practice-based Research Networks. *Clin Regist Clin Trials*. 2018. doi:10.1002/CENTRAL/CN-01660857

94. Tsao S, Willard-Grace R, Wolf J, et al. Implementation and Impact of the Pulmonary Specialist Health Coach Consultation Model to Improve Care for Patients with COPD. *Jt Comm J Qual patient Saf*. 2021;47(11):739-747. doi:10.1016/J.JCJQ.2021.08.003

95. Vandemheen KL, O'Connor A, Bell SC, et al. Randomized trial of a decision aid for patients with cystic fibrosis considering lung transplantation. *Am J Respir Crit Care Med*. 2009;180(8):761-768. doi:10.1164/rccm.200903-0421OC

96. Wahlberg H, Braaten T, Broderstad AR. Impact of referral templates on patient experience of the referral and care process: A cluster randomised trial. *BMJ Open*. 2016;6(10):1-8. doi:10.1136/bmjopen-2016-011651

97. Waller A, Girgis A, Johnson C, et al. Improving outcomes for people with progressive cancer: Interrupted time series trial of a needs assessment intervention. *J Pain Symptom Manage*. 2012;43(3):569-581. doi:10.1016/j.jpainsymman.2011.04.020

98. Wang KY, Chian CF, Lai HR, Tarn YH, Wu CP. Clinical pharmacist counseling improves outcomes for Taiwanese asthma patients. *Pharm World Sci*. 2010;32(6):721-729. doi:10.1007/s11096-010-9427-4

99. Weekes CE, Emery PW, Elia M. Dietary counselling and food fortification in stable copd: A randomised trial. *Thorax*. 2009;64(4):326-331. doi:10.1136/thx.2008.097352

100. Williams PD, Graham KM, Storlie DL, et al. Therapy-related symptom checklist use during

treatments at a cancer center. *Cancer Nurs*. 2013;36(3):245-254. doi:10.1097/NCC.0b013e3182595406

101. Wilson SR, Strub P, Knowles SB, Buist AS, Huang Q, Nguyen M. Ethnicity, Income, and Education as Potential Modifiers of the Effects of Shared Treatment Decision-Making (SDM) Between Asthma Care Manager and Patient on Asthma Controller Medication Adherence: The Better Outcomes of Asthma Treatment (BOAT) Trial. *J Allergy Clin Immunol*. 2009;123(2):S72-S72. doi:10.1016/j.jaci.2008.12.247

102. Wilson S. Does Shared Decision-Making Improve Asthma Outcomes? *Clin Regist Clin Trials*. 2005. doi:10.1002/CENTRAL/CN-02031001

103. Wilson S. Does shared decision-making improve adherence in asthma. *Clin Regist Clin Trials*. 2005. doi:10.1002/CENTRAL/CN-01427587

104. Wood-baker R, Reid D, Robinson A. Wood-Baker et al: Clinical trial of community nurse mentoring to improve self-management in patients with chronic obstructive pulmonary disease. International Journal of COPD 2012, 7:407-13. 2012:407-413. http://www.pubmedcentral.nih.gov/articlerender.fcgi?artid=3402057&tool=pmcentrez&rendertype=abstract.

105. Yoo SH, Yun YH, Kim KN, et al. The impact of caregiver's role preference on decisional conflicts and psychiatric distresses in decision making to help caregiver's disclosure of terminal disease status. *Qual Life Res*. 2018;27(6):1571-1581. doi:10.1007/s11136-018-1814-7

106. Zakrisson AB, Arne M, Hasselgren M, Lisspers K, Ställberg B, Theander K. A complex intervention of self-management for patients with COPD or CHF in primary care improved performance and satisfaction with regard to own selected activities; A longitudinal follow-up. *J Adv Nurs*. 2019;75(1):175-186. doi:10.1111/jan.13899

107. Zanartu CP, Camacho FJ, Nevadunski NS, et al. Speaking the same language: a feasibility trial for a novel visual communication tool for oncologist-patient treatment discussions. *Psychooncology*. 2017;26(7):1050-1052. doi:10.1002/pon.4246

108. Au DH, Udris EM, Engelberg RA, et al. A randomized trial to improve communication about end-of-life care among patients with COPD. *Chest*. 2012;141(3):726-735. doi:10.1378/chest.11-0362

109. Gagné ME, Légaré F, Moisan J, Boulet L-P. Impact of Adding a Decision Aid to Patient

Education in Adults with Asthma: A Randomized Clinical Trial. Leroyer C, ed. *PLoS One*. 2017;12(1):e0170055. doi:10.1371/journal.pone.0170055

110. Collinsworth AW, Brown RM, Sjames C, stanford RH, Alemayehu D, Priest EL. The impact of patient education and shared decision making on hospital readmissions for COPD. *Int J COPD*. 2018;13:1325-1332. doi:10.2147/COPD.S154414

111. Granados-Santiago M, Valenza MC, López-López L, Prados-Román E, Rodríguez-Torres J, Cabrera-Martos I. Shared decision-making and patient engagement program during acute exacerbation of COPD hospitalization: A randomized control trial. *Patient Educ Couns*. 2019;103(4):702-708. doi:10.1016/j.pec.2019.12.004

112. Brundage MD, Feldman-Stewart D, Cosby R, et al. Phase I study of a decision aid for patients with locally advanced non-small-cell lung cancer. *J Clin Oncol*. 2001;19(5):1326-1335. doi:10.1200/JCO.2001.19.5.1326

113. Myers RE, Advani SM, Myers P, et al. Engaging Patients with Late-Stage Non-Small Cell Lung Cancer in Shared Decision Making about Treatment. *J Pers Med*. 2021;11(10). doi:10.3390/JPM11100998

114. Walters J, Cameron-Tucker H, Wills K, et al. Effects of telephone health mentoring in community-recruited chronic obstructive pulmonary disease on self-management capacity, quality of life and psychological morbidity: A randomised controlled trial. *BMJ Open*. 2013;3(9). doi:10.1136/bmjopen-2013-003097

115. Wilson SR, Strub P, Buist AS, et al. Shared treatment decision making improves adherence and outcomes in poorly controlled asthma. *Am J Respir Crit Care Med*. 2010;181(6):566-577. doi:10.1164/rccm.200906-0907OC
