# Supplementary material for: Systematic review of shared decision-making interventions for people living with chronic respiratory diseases
Source: BMJ Open. 2023 May 2;13(5):e069461. doi: 10.1136/bmjopen-2022-069461 (PMC10163462; doi:10.1136/bmjopen-2022-069461)
Supplement: Author's manuscript [file bmjopen-2022-069461.draft_revisions.pdf]

BMJ Open

BMJ Open is committed to open peer review. As part of this commitment we make the peer review history of every article we publish publicly available.

When an article is published we post the peer reviewers' comments and the authors' responses online. We also post the versions of the paper that were used during peer review. These are the versions that the peer review comments apply to.

The versions of the paper that follow are the versions that were submitted during the peer review process. They are not the versions of record or the final published versions. They should not be cited or distributed as the published version of this manuscript.

BMJ Open is an open access journal and the full, final, typeset and author-corrected version of record of the manuscript is available on our site with no access controls, subscription charges or pay-per-view fees (http://bmjopen.bmj.com).

If you have any questions on BMJ Open's open peer review process please email info.bmjopen@bmj.com

BMJ Open

A systematic review of shared decision-making interventions for people living with chronic respiratory diseases

Journal:	*BMJ Open*
Manuscript ID	bmjopen-2022-069461
Article Type:	Original research
Date Submitted by the Author:	21-Oct-2022

Complete List of Authors:	Barradell, Amy; University of Leicester, Department of Respiratory Sciences; University Hospitals of Leicester NHS Trust, Centre for Exercise and Rehabilitation Science<br>Gerlis, Charlotte; University Hospitals of Leicester NHS Trust, Centre of Exercise and Rehabilitation Science<br>Houchen-Wolloff, Linzy; University Hospitals of Leicester NHS Trust, Centre of Exercise and Rehabilitation Science<br>Bekker, Hilary; University of Leeds, Leeds Institute of Health Sciences; Central Denmark Region and Aarhus University, Research Centre for Patient Involvement<br>Robertson, Noelle; University of Leicester, School of Psychology<br>Singh, Sally; University Hospitals of Leicester NHS Trust, Cardiac/Pulmonary Rehabilitation; University of Leicester, Respiratory Sciences

Keywords:	RESPIRATORY MEDICINE (see Thoracic Medicine), REHABILITATION MEDICINE, Quality in health care < HEALTH SERVICES ADMINISTRATION & MANAGEMENT

SCHOLARONE™
Manuscripts

I, the Submitting Author has the right to grant and does grant on behalf of all authors of the Work (as defined in the below author licence), an exclusive licence and/or a non-exclusive licence for contributions from authors who are: i) UK Crown employees; ii) where BMJ has agreed a CC-BY licence shall apply, and/or iii) in accordance with the terms applicable for US Federal Government officers or employees acting as part of their official duties; on a worldwide, perpetual, irrevocable, royalty-free basis to BMJ Publishing Group Ltd ("BMJ") its licensees and where the relevant Journal is co-owned by BMJ to the co-owners of the Journal, to publish the Work in this journal and any other BMJ products and to exploit all rights, as set out in our licence.

The Submitting Author accepts and understands that any supply made under these terms is made by BMJ to the Submitting Author unless you are acting as an employee on behalf of your employer or a postgraduate student of an affiliated institution which is paying any applicable article publishing charge ("APC") for Open Access articles. Where the Submitting Author wishes to make the Work available on an Open Access basis (and intends to pay the relevant APC), the terms of reuse of such Open Access shall be governed by a Creative Commons licence – details of these licences and which Creative Commons licence will apply to this Work are set out in our licence referred to above.

Other than as permitted in any relevant BMJ Author's Self Archiving Policies, I confirm this Work has not been accepted for publication elsewhere, is not being considered for publication elsewhere and does not duplicate material already published. I confirm all authors consent to publication of this Work and authorise the granting of this licence.

For peer review only

SDM Systematic Review

A systematic review of shared decision-making interventions for people living with chronic respiratory diseases

Authors

Barradell, AC[1-3]; Gerlis, C[3]; Houchen-Wolloff L[1,3]; Bekker, HL[5,6]; Robertson, N[4]; Singh, SJ[1,3].

Author affiliations:

1. Department of Respiratory Sciences, University of Leicester, University Road, Leicester LE1 7RH
2. National Institute for Health Research (NIHR) Applied Research Collaboration (East Midlands), College of Medicine, Biological Sciences & Psychology, Leicester General Hospital, Gwendolen Road, Leicester, LE5 4PW
3. Centre for Exercise and Rehabilitation Science, Leicester Biomedical Research Centre-Respiratory, Glenfield Hospital, Groby Road, Leicester, LE3 9QP
4. Department of Neuroscience, Psychology and Behaviour, University of Leicester, University Road, Leicester LE1 7RH
5. Leeds Unit of Complex Intervention Development (LUICD), School of Medicine, University of Leeds, Leeds, LS2 9JT
6. Research Centre for Patient Involvement, Central Denmark Region and Aarhus University, DK-8000 Aarhus C

Corresponding author:

Name: Amy C Barradell

Address: Centre for Exercise and Rehabilitation Science, Leicester Biomedical Research Centre-Respiratory, Glenfield Hospital, Groby Road, Leicester, LE3 9QP

Email: ab1081@le.ac.uk

For peer review only - http://bmjopen.bmj.com/site/about/guidelines.xhtml

Tel: 0116 258 3035

Short running title

A systematic review of SDM interventions in CRD

Authorship statement

ACB conceived the review. All authors contributed the protocol development. ACB conducted the search in collaboration with the University Hospitals of Leicester NHS Trust Librarian. ACB and CG screened all citations. All authors confirmed studies to be included. ACB conducted data extraction and CG conducted a 10% data quality check to ensure accuracy in this process. ACB and CG conducted risk of bias and certainty of evidence assessments. ACB drafted the manuscript and all authors critically revised for significant intellectual content and insight. In addition, all authors gave final approval of the manuscript version for publication. All authors are responsible for the overall content as guarantors. All authors report no conflicts of interest.

Disclosure statement

The National Institute for Health Research (NIHR Applied Research Collaboration [East Midlands] (NIHR ARC East Midlands) and the Centre for Exercise and Rehabilitation Science (CERS) funded this research. The funders had no role in the inception, data extraction or analysis phases of this review. The views expressed in this article do not communicate an official position of the University of Leicester, NIHR, CERS, University of Leeds, or Aarhus University.

For peer review only

For peer review only - http://bmjopen.bmj.com/site/about/guidelines.xhtml

Abstract

Shared Decision-Making (SDM) supports patients to make informed and value-based decisions about their care. Patients with Chronic Obstructive Pulmonary Disease (COPD) express barriers to accessing Pulmonary Rehabilitation (PR). We are developing a complex intervention to enable healthcare professionals to support PR decision-making for patients living with COPD. To identify components for this we need to evaluate others carried out in Chronic Respiratory Diseases (CRD).

We aimed to evaluate the impact of SDM interventions on patient decision-making (primary outcome) and downstream health-related outcomes (secondary outcome) using systematic review methods.

Electronic databases and trial registers were searched for trials evaluating SDM interventions in patients living with CRD using quantitative or mixed methods. Risk of bias and certainty of evidence was assessed using the Cochrane ROB2, ROBINS-I, and GRADE tools. The Making Informed Decisions Individually and Together (MIND-IT) conceptual model guided the descriptive and narrative synthesis.

Eight studies [n=1,596 (of 17,466 citations identified)] fulfilled the inclusion criteria.

Five studies included components targeting the patient, healthcare professionals and consultation process (demonstrating adherence to the MIND-IT model). All studies reported their interventions improved patient decision-making and health-related outcomes. No outcome was reported consistently across studies. Four studies had high risk of bias, three had low quality of evidence. Intervention fidelity was reported in two studies.

These findings suggest developing a SDM intervention including a patient decision aid, healthcare professional training, and a consultation prompt is likely to support patient PR decisions, and health-related outcomes. Using a complex intervention development and evaluation research framework

For peer review only

For peer review only - http://bmjopen.bmj.com/site/about/guidelines.xhtml

will likely lead to more robust research, and a greater understanding of service needs when integrating the intervention within practice.

Strengths and limitations

- The inclusion of studies from across the chronic respiratory disease population enabled us to draw conclusions about the efficacy of shared decision-making within this population when other reviews of specific respiratory conditions have been unable to.
- The search criteria may have meant we missed studies which used alternative terminology for shared decision making.
- We were unable to perform meta-analysis or sub-group analysis due to the heterogeneity of included studies and their outcome measures.

Key Messages

What is already known on this topic

SDM supports patients to make informed and value-based decisions about their care. However, the efficacy of SDM interventions for adults living with CRD is largely unknown.

What this study adds

This review synthesises the evidence for SDM interventions in those living with CRD who are making decisions about their care. It finds that SDM interventions improve both decision-making and health-related outcomes for these patients.

How this study might affect research, practice or policy

This review suggests the development of a SDM intervention, including a patient decision aid, healthcare professionals training, and a consultation prompt, will likely to support patient PR decisions, and health-related outcomes.

Medical Subject Headings

Informed decision making

Decision support

Patient-centred care

Professional-patient relations

Lung disease

For peer review only - http://bmjopen.bmj.com/site/about/guidelines.xhtml

Registration:

The systematic review protocol was registered on PROSPERO on 8th April 2020 (ID: CRD42020169897).

Primary source of funding:

This project is funded by the National Institute for Health Research (NIHR) Applied Research Collaboration East Midlands (ARC EM) and the Centre for Exercise and Rehabilitation Science (CERS). The views expressed are those of the authors and not necessarily those of the NIHR or the Department of Health and Social Care.

For peer review only

For peer review only - http://bmjopen.bmj.com/site/about/guidelines.xhtml

Introduction

Patients living with chronic obstructive pulmonary disease (COPD) account for a significant proportion of death and disability worldwide[1,2]. Pulmonary rehabilitation (PR) is a recommended evidence-based intervention to improve the physical and psychological health of this population[3] but patients express significant barriers to accepting PR[4,5]. It is therefore proposed, healthcare professionals should seek to engage patients in informed decisions about their enrolment into a programme[5,6].

Shared Decision-Making (SDM) is a core principle of personalised care, which encourages healthcare professionals to actively engage patients in healthcare decisions[7]. This style of communication requires patients and healthcare professionals to share knowledge about the health condition (i.e., the lived experience and evidence-based treatment), then engage in a period of deliberation to review the pros and cons of each option for the patients' life and agree upon an optimal healthcare choice.

This systematic review will inform the development and evaluation of a SDM intervention enabling healthcare professionals to support patients living with COPD make informed decisions about PR. SDM can be viewed as a complex intervention as it requires engagement from multiple stakeholders to enhance communication within a consultation. The Making Informed Decisions Individually and Together (MIND-IT) model[8] provides a conceptual framework to represent components within a multiple stakeholder decision support intervention and its evaluation (Figure 1). There are a range of interventions currently supporting SDM in consultations, including Patient Decision Aids (PtDAs) with or without decision coaching, and SDM training for healthcare professionals.

PtDAs are resources providing accurate information to help people make informed, value-based decisions between healthcare options[7]. They are informed by decision science research to structure the content in a way that reduces the cognitive effort needed to process facts, present balanced and neutral information, and provide prompts encouraging people to trade-off their

For peer review only

evaluations of the consequences, and choose an option that is best for their life[9]. The integration of PtDAs into multiple healthcare contexts has shown to improve the frequency of SDM and patient-centred decisions, increase patient knowledge of their condition and the options available, enhance involvement in the decision-making process, and reduce decisional conflict[10–14].

Decision Coaching training for healthcare professionals aims to develop their skills in supporting patients to reason between healthcare options rather than providing information about a medical best treatment plan[15]. The training guides healthcare professionals to adopt a non-directive approach in SDM consultations by supporting patients to consider all available options, preparing them for deliberating over options, and ensuring any chosen option is then implemented. Research suggests decision coaching supports patient engagement in SDM[13,16–18] and in combination with a PtDA, is a robust method to facilitate SDM[16].

SDM training for healthcare professionals aims to help them adopt a SDM approach to delivering care[19]. There is no consensus guiding the content of this training, however NICE offers a training package[19] which includes a range of knowledge and skills-based modules on patient-centred care, presenting risk information and uncertainty, decision and cognitive science, evidence-based medicine, multiple stakeholder collaboration, and consultation skills. The evidence evaluating the effectiveness of this training on SDM use and impact in practice is weak due to the heterogeneity in study designs, methodology, and evaluation strategies[16,17].

SDM interventions are assessed using multiple outcome measures and include: within-consultation communication using healthcare professionals observation (e.g. the Observer OPTION 5 tool[20]) and patient self-report questionnaires (e.g. the Shared Decision-Making Questionnaire[21]), patient knowledge of healthcare options, attitudes, and skills (e.g. self-reported perception of risk, the Values' scale[22], the Decision Self Efficacy Scale[23]), patient decision outcomes (e.g. the choice made, the Decisional Conflict Scale[24], the Decision Regret Scale[25]), overall SDM experience from patient and healthcare professionals perspectives (e.g. the iSHARE tool[26]), and downstream

For peer review only - http://bmjopen.bmj.com/site/about/guidelines.xhtml

patient health-related outcomes (e.g. medication/treatment adherence, healthcare utilisation, disease knowledge, and quality of life measures).

In this systematic review we sought to evaluate the efficacy of SDM interventions compared to usual care in patients living with Chronic Respiratory Disease (CRD). To date, the efficacy of SDM interventions for adults living with CRD is largely unknown. Two systematic reviews have been published to synthesise evidence on the impact of SDM interventions on people with asthma[27] and cystic fibrosis[28] but they found the evidence base to be too heterogeneous to draw meaningful conclusions about the relationship between the interventions and improved SDM or downstream health-related outcomes and nearly all the review population were children and adolescents. Therefore, there is a clear need to evaluate the efficacy of SDM interventions upon adult patients living with Chronic Respiratory Disease (CRD; e.g. COPD, asthma, idiopathic pulmonary fibrosis, lung cancer).

In this review, we aim to identify the components used to support SDM within this health context and synthesise evidence of their impact on patient decision-making and downstream health-related outcomes. We use the Preferred Reporting in Systematic Reviews and Meta-Analyses (PRISMA) guidelines to report this review.

(Insert Figure I)

Methods

Eligibility criteria

Participants

Adults (aged 18+ years) diagnosed with any CRD (and their carers).

Intervention

Any intervention which orchestrated SDM between patients and healthcare professionals, with or without disease education, decision coaching training, or SDM training.

For peer review only

For peer review only - http://bmjopen.bmj.com/site/about/guidelines.xhtml

Comparison

Any concurrent control groups enrolled in the included studies, but not receiving a SDM intervention.

Outcomes

Primary outcomes of interest included measures of the quality of patient decision-making (e.g. participants' preparedness for decision-making, healthcare professionals and patient involvement in the decision-making process, knowledge of the available options and associated risks, decisional conflict, concordance between the participants' values and the decision made, decision confidence, and decisional regret).

Secondary outcomes of interest were patients' downstream health-related outcomes (e.g. behavioural activation, quality of life, knowledge of condition, healthcare utilisation). Specific qualitative measures of healthcare professionals' attitudes and experience of the SDM intervention, attendance, attrition, and fidelity were also included.

Study design

Randomised and non-randomised quantitative or mixed method controlled trials were sought for inclusion in this review.

Exclusions were hypothetical decision scenarios, and studies not reported in English.

Information sources

An electronic search was conducted on inception through to 14th December 2021 using the following databases and registries: MEDLINE (1966-), EMBASE (1947-), PSYCHINFO (1887-), CINAHL (1937-), PEDRO (1999-), Cochrane Central Register of Controlled Trials (1993-) the International Clinical Trials Registry Platform Search Portal (2006-), ClinicalTrials.gov (2000-), PROSPERO (2011-), ISRCTN (2000-).

For peer review only - http://bmjopen.bmj.com/site/about/guidelines.xhtml

Search strategy

The search algorithm (Supplementary Material) was developed and piloted with support from the University Hospitals of Leicester NHS Trust Library Services. The algorithm was piloted using the MEDLINE database with limits imposed to retrieve articles in English. The algorithm was adapted to the syntax and MESH headings for each database.

Selection process

Articles were uploaded to the web-based platform, Rayaan (https://rayyan.qcri.org/welcome) to facilitate independent review. Duplicate articles were removed and articles reporting findings across multiple outputs were highlighted to review in tandem. Two reviewers (AB, CG) screened titles and abstracts using the inclusion and exclusion criteria. Eligible full texts were reviewed by the same two authors. Any ambiguity in the eligibility of an article was discussed and resolved with the co-authors.

Data collection process

A review-specific data extraction form was used, informed by Cochrane data extraction forms[29]. This was piloted prior to use to ensure consistency in the extraction process. One reviewer (AB) conducted data extraction. A random 10% selection was reviewed by a co-author (CG) to validate the extracted data. Since there were no errors in data extraction were identified, no further validation was considered necessary. Ambiguity in the extracted data was discussed by all authors and resolved by contacting individual study teams. Specific data items for primary and sub-group analysis are outlined in the Supplementary Material.

Study risk of bias assessment

RCTs were assessed using the Cochrane Risk of Bias tool[30] which measures the following domains: randomisation process, deviations from intended interventions, missing outcome data, measurement of the outcomes, and selection of the reported result. Non-RCTs were assessed using the Cochrane ROBINS-I tool[31] which measures the following domains: confounding, selection of

For peer review only

study participants, classification of interventions, deviations from intended interventions, missing data, measurement of outcomes, and selection of the reported result. Two authors (AB, CG) independently rated the studies. Any discrepancy in judgements were reviewed and resolved by the co-authors.

Certainty of evidence

The strength of evidence for all reported outcomes was assessed using the Grading of Recommendations Assessment, Development & Evaluation working group methodology (GRADE) which measures the following domains: risk of bias, inconsistency, indirectness, imprecision, and publication bias. Initial judgements were based upon study designs and then systematically reduced if the quality of evidence was poor or increased if a significant effect, dose response or evidence of the elimination of all plausible residual confounding variables and bias was observed. Two authors (AB, CG) independently assessed the criteria. Any discrepancies were resolved with the co-authors.

Synthesis methods

All outcomes were tabulated with continuous data presented as mean (standard deviation) and categorical data as percentage proportions.

Statistical analysis of the results was not appropriate due to the clinical and methodological heterogeneity of the outcome measures between studies. A narrative synthesis[32], with reference to the MIND-IT model, was undertaken to identify the components used to support SDM in CRD and synthesise the evidence of their impact on patient decision-making and downstream health-related outcomes both between and within included studies.

Results

Study selection

The search generated 17,466 articles. Following the removal of duplicates, 15,861 articles were retained for abstract screening. Of these, 117 underwent full text screening and 8 were retained for

For peer review only - http://bmjopen.bmj.com/site/about/guidelines.xhtml

inclusion in this analysis (109 excluded). Most studies were excluded because of the participant population (Figure II). A full list of excluded studies is provided in the Supplementary Material.

(Insert Figure II)

Study characteristics

Characteristics of the eight included studies[33–40] are provided in Tables I and II. Six studies were RCTs[33,35–39], two were non-RCT[34,40]. Two studies were conducted with COPD outpatients[33,38], two with COPD inpatients[35,37], two with lung cancer outpatients[34,40], and two with asthma outpatients[36,39]. The theoretical underpinning of interventions, reported by study authors, included social cognitive theory[33], principles of self-management and SDM[35], SDM[36,39,40], self-management[37] and cognitive behavioural psychology[38]. One study[34] did not report a theoretical underpinning. The interventions of five studies included all three components of the MIND-IT multiple stakeholder model (Figure III). Four studies included additional components to SDM within their intervention groups; two included a self-management programme[37,38], and three included an educational component[35,36,38].

(Insert Tables I and II)

(Insert Figure III)

Risk of bias within studies

Two RCTs were judged to have high risk of bias[35,38] and four RCTs were judged to have some concerns [33,36,37,39] (Figure IV). One study was judged as high due to insufficient efforts in randomisation, blinding and attrition[35], the other was due to attrition and poor fidelity[38]. The two non-RCTs were both judged to have serious risk of bias because of insufficient efforts in blinding[34,40] (Figure V).

(Insert Figure IV and V)

For peer review only - http://bmjopen.bmj.com/site/about/guidelines.xhtml

Results of synthesis

Results of individual studies are presented in turn alongside certainty of evidence assessments in Table I.

Decision-making outcomes

Five outcomes captured the quality of patient decision-making (Table I); quality of communication, involvement in decision-making, decisional conflict, strength of treatment preference, and knowledge of treatment options.

Quality of communication

One study reported that quality of communication increased in intervention and control groups, however, a significantly greater increase occurred in the intervention group alone [between group difference: 5.7 points; p<0.05][33]. The same study reported an increase in the occurrence of patient-centred communication in the intervention group alone, as measured by a study-specific 4-item self-reported questionnaire [Item 1. Intervention: 35.2(74.6) Control: 15.9(58.2) p<0.05; Item 2. Intervention: 60.3(122.9) Control: 30.8(175.5) p<0.05; Item 3. Intervention: 53.6(115.1) Control: 45.2(73.3) p>0.05; Item 4. Intervention: 86. 2(84.6) Control: 75.2(115.0) p<0.05].

Involvement in decision-making

Two studies reported significantly increased patient involvement in the decision-making process when compared to a control group [6months between group difference: 0.4(0.2), p<0.05; 12 months between group difference: 0.3(0.1), p<0.05][38] and a group where clinicians made treatment decisions [immediately post-intervention between group difference: 2.5(0.9), p<0.05][39].

Decisional conflict

Two studies (one feasibility, one pilot controlled before and after study) reported a trend for reducing decisional conflict. The first reported 87% reduction in decisional conflict[34], the other reported a mean difference of +1 indicating increased decision certainty[40]. Elsewhere, a RCT measuring disease education with and without a patient decision aid reported significantly reduced

For peer review only

For peer review only - http://bmjopen.bmj.com/site/about/guidelines.xhtml

decisional conflict in the intervention and control groups [Intervention difference from baseline: 8.1(22.5), p<0.05; Control difference from baseline: 9.1(27.7), p<0.05], but no significant difference between groups [between group difference: 2.5, p>0.05)[36].

Strength of treatment preference

One feasibility study reported the strength of patients' treatment preference increased from 80-100% following exposure to a patient decision aid[34].

Knowledge of treatment options

One feasibility study reported an increasing trend for treatment specific knowledge for three year outcome survival[a] and median survival outcome[b] [Knowledge of survival outcomes with treatment 1 alone (between group difference: 60%[a]; 54%[b]); Knowledge of direction of survival difference between treatments (between group difference: 27%[a]; 40%[b]); Knowledge of the magnitude of survival difference between treatments (between group difference: 73%[a]; 67%[b])][34].

A pilot controlled before and after study reported awareness of treatment options increased from 40% to 100% of participants post intervention[40].

Health-related outcomes

Six outcomes captured downstream health-related outcomes (Table I); disease knowledge, hospitalisations, behavioural activation quality of life, intervention fidelity, attendance, and attrition.

Disease knowledge

One study reported significantly increased disease-specific knowledge scores in the intervention group [between group difference: 3.89(0.7), p<0.05] which maintained at 3 months follow up [between group difference: 3.88(0.8), p<0.05][37]. However, another study reported knowledge scores increased equally amongst intervention and control groups [Intervention: 3.6(8.9), p<0.05; Control: 2(7.3), p<0.05][36].

For peer review only

For peer review only - http://bmjopen.bmj.com/site/about/guidelines.xhtml

Hospitalisations

Two studies reported no change in all-cause hospitalisations between intervention and control groups[35,38]. One study observed a significant reduction in healthcare utilisation in the SDM and clinician decision-making groups but not in the control group [SDM-control between group difference: 20.36(1.5), p<0.05; SDM-clinician decision-making between group difference: 0.01(2.7), p>0.05; clinician decision-making-control: 20.37(1.5), p<0.05][39].

Behavioural activation

One feasibility study showed both the intervention and control group had a trend for increased levels of activation post intervention [between group difference: 0.52(0.9) and 0.69(1.0) points respectively, p<0.05][35].

One pilot controlled before and after study reported 80% of participants had made a treatment decision at 30 days and 40% had begun a treatment which matched their treatment preference[40].

Two studies reported medication adherence significantly improved in the intervention groups alone[37,39]. However, the control group receiving a clinician decision-making intervention in one of these studies[39] also showed significantly adherent medication behaviour and no between group difference [SDM–clinician decision-making between group difference: 0.08(0.4), p>0.05]. One study measured the correct administration of medication and post intervention reported non-significant improvements in the correct use of asthma medication in the intervention and the control group with no significant difference between groups [between group difference: 0.17(0.7), p>0.05][36].

One study reported significant between group differences for improvements in nutritional behaviour at 3 months post inpatient intervention initiation [between group difference: 5.03; p<0.05) and physical activity at 3 months post intervention initiation (between group difference: 716.01; p<0.05][37].

For peer review only

Quality of life

Two studies reported no significant difference between intervention and control groups post intervention [Discharge between group difference: 6.15(27.5), p>0.05); 6 months between group difference: 1.9(2.7), p>0.05); 12 months between group difference: 1.4(1.5), p>0.05][37,38]. However, one of the studies did at 3 months post discharge [between group difference: 8.28(50.6), p<0.05][37]. Whilst two further studies reported either a trend for improvement [between group difference: 4.89; p<0.05)[35] or a significant improvement in the intervention group alone [SDM to control between group difference: 0.39(1.0), p<0.05][39].

Intervention fidelity

Only two studies reported intervention fidelity explicitly[38,39]. Both studies rated a specified selection of intervention audio recordings. One reported high adherence to the intervention protocol [SDM, 4/5; clinician decision-making, 3.9/5][39]. The other reported wide variation in adherence between the different intervention components, with lower delivery of cognitive-behavioural components which were described as the backbone of the intervention[38].

Attendance and attrition

For inventions involving single sessions, there was no attrition during intervention delivery[33,34,36,40]. For interventions involving multiple sessions, completion of inpatient interventions ranged from 37%[35] to 100%[37], whereas, outpatient interventions ranged from 72%[38] to 75.5%[39].

Results from sub-group analysis are provided in the Supplementary Material.

Discussion

This review provides the first synthesis of studies evaluating interventions to support SDM between patients with CRD and healthcare professionals. All eight studies reported their interventions improved patient decision-making and downstream health-related outcomes. There were

For peer review only

For peer review only - http://bmjopen.bmj.com/site/about/guidelines.xhtml

improvements in the quality of communication with healthcare professionals[33], the occurrence of patient-centred communication[33], involvement in decision-making[38,39], decisional conflict[36,40], health-related knowledge[37], behavioural activation[37,39,40], quality of life[37,39], and healthcare utilisation[39]. There was no one consistent outcome measure used in all studies to enable comparison across studies. Additionally, three studies measured intervention feasibility and therefore were unable to observe intervention efficacy[34,35,40].

The most consistently reported outcomes were the downstream health-related outcomes behavioural activation and quality of life. Five studies reported improvements in behavioural activation across six outcome measures[35–37,39,40]. Between group differences were only observed for two of the outcomes (nutritional status and physical activity) and this was in just one study[37]. Two studies reported improved health-related quality of life post intervention[35,39] but one of these was a feasibility study[35].

Unlike other reviews in the CRD field[27,28], this review found variation in the intervention components. Only five studies contained all active SDM components from the MIND-IT model (Figure III). Gagne's[36] and Au's[33] study did not report a period of deliberation between patients and healthcare professionals, suggesting the decision-making process was not shared, and in Walters[38] study, there was no report of patients and healthcare professionals exchanging personal and evidence-based information suggesting the decision-making process was not informed. Without adequate inclusion of all SDM components, it is difficult to conclude which intervention components support which outcomes.

Furthermore, there was little evidence in the descriptions of the interventions to show they had been developed in-line with recommended SDM intervention and user-centred design guidelines (e.g. [41],[42] and [43]). There was also limited evidence to suggest included studies had identified their target populations' (e.g. patient or healthcare professionals) needs when developing their intervention and its evaluation. For example, despite the vast array of participants' educational

For peer review only - http://bmjopen.bmj.com/site/about/guidelines.xhtml

attainment across studies, there was little evidence studies observed this and tailored the SDM materials accordingly. This observation is of concern because lower health literacy has been shown to affect key decision-making outcomes[44]. A recent expanded model of SDM has been proposed to reflect the necessity of patients' health literacy skills as this enables them to have meaningful engagement in the decision-making process[45]. In our review, no study had considered the health literacy of their participants prior to enrolment through the use of validated measures (e.g. test of functional health literacy in adults[46]), nor the use of a standardising health literacy resources such as the Plain English Campaign[47] to ensure materials were at the appropriate comprehension level and, finally, no study had considered using the low literacy version of the Decisional Conflict Scale[48], despite its ease of access. Furthermore, no study reported healthcare professional-directed intervention components (e.g. healthcare professional training) or used a user-centred design or implementation process.

Additionally, studies made little reference to social or familial support in the decision-making process, which is unusual as social support is often associated with improvements in self-management behaviour amongst patients with CRD[49,50]. Social support may contribute to informed information exchange and provide confidence in the decision-making process. Interestingly, one study specifically excluded family members or friends from the SDM consultation as they believed it would introduce bias[34].

Limitations of the evidence included in the review

The interpretation of these results should be considered in light of the inclusion of studies which utilised non-validated outcome measures. Only one study used a dose-matched intervention in the control group[38] and fidelity was only reported explicitly in two studies[38,39].

Limitations of the review process

The inconsistent outcome measures within this review precluded meta-analysis. Four studies had a high risk of bias[34,35,38,40] and three had low or very low quality of evidence[34,38,40], thereby

For peer review only

For peer review only - http://bmjopen.bmj.com/site/about/guidelines.xhtml

limiting the validity and generalisability of the results. The search criteria was broad, however, if studies had used alternate terminology for SDM, it is possible some may have been missed. The multi-component interventions meant intervention effects could not be discernibly attributed to the SDM component or the education and self-management components. Furthermore, two studies both provided their control groups with disease education which may have driven the positive effects observed by the control groups[35,36]. Included studies encompassed a variety of healthcare settings with decisions varying in focus from self-management to treatment decision-making and end of life care. Despite excluding studies involving hypothetical decision-making to reduce the disparity in importance attributed to the decision-making process, we cannot assume participants attributed the same importance to all decisions. Additionally, the studies length of follow-ups varied considerably, however, as the type of decisions were so varied the allocated follow-up period may have been appropriate. For example, Janssen and colleagues showed that patients with COPD change their mind about end of life care throughout the course of their healthcare journey[51], suggesting a long-term follow-up period for end of life decision-making is appropriate. Whereas health decisions with more immediate affect (e.g. acceptance of a referral to pulmonary rehabilitation) may require a shorter follow-up period. As more SDM intervention studies are conducted in CRD, an updated review may well benefit from statistical sub-group analysis for studies length of follow up and/or decision posed. Additionally, the decision to exclude qualitative and cross-sectional studies may have limited our ability to fully explore participants' experiences of the SDM process and the development and implementation of interventions.

Implications of the results

This synthesis provides some evidence that CRD SDM interventions improve the patient decision-making process and downstream health-related outcomes. However, the evidence is weak, and the interventions at times incongruent with all active SDM components. Therefore, this limits the rigour

For peer review only - http://bmjopen.bmj.com/site/about/guidelines.xhtml

in evaluating the impact of SDM interventions within CRD. There were also no studies exploring the efficacy of SDM in PR decision-making and implementation in practice.

Based on the available evidence, this review supports the development of a SDM intervention to enable healthcare professionals to support COPD patients informed decision-making for PR and for it to be evaluated within a complex intervention development and evaluation research framework. This is especially relevant given the rise in alternate PR delivery models due to the Covid-19 pandemic. Importantly, this intervention should be underpinned by SDM theoretical models (e.g. MIND-IT[8], extended SDM model involving consideration of health literacy[45]), adhere to the appropriate intervention development guidelines (e.g.[41–43]), and be evaluated in a single or double-blind RCT with dose-matched controls.

Other information

Protocol and registration

This review was conducted in accordance with the Preferred Reporting in Systematic Reviews and Meta-Analyses (PRISMA)[52] and registered on PROSPERO (registration number: CRD42020169897). The completed PRISMA checklist is provided in the Supplementary Material.

Support

The National Institute for Health Research (NIHR Applied Research Collaboration [East Midlands] (NIHR ARC East Midlands) and the Centre for Exercise and Rehabilitation Science (CERS) funded this research.

Competing interests

The authors have no conflicts of interest to declare.

Availability of data, code and other materials

The MEDLINE search strategy, data items sought, methods for sub-group analysis, characteristics of excluded studies, and results of sub-group analysis are provided in the Supplementary Materials.

For peer review only

For peer review only - http://bmjopen.bmj.com/site/about/guidelines.xhtml

Acknowledgements

The authors would like to thank the UHL Library Services for their contribution to the search criteria and piloting the search. Furthermore, we would like to thank all study authors for sharing their data, when requested.

References

1 Lozano R, Naghavi M, Foreman K, *et al.* Global and regional mortality from 235 causes of death for 20 age groups in 1990 and 2010: A systematic analysis for the Global Burden of Disease Study 2010. *Lancet* 2012;**380**:2095–128. doi:10.1016/S0140-6736(12)61728-0

2 Vos T, Flaxman AD, Naghavi M, *et al.* Years lived with disability (YLDs) for 1160 sequelae of 289 diseases and injuries 1990-2010: A systematic analysis for the Global Burden of Disease Study 2010. *Lancet* 2012;**380**:2163–96. doi:10.1016/S0140-6736(12)61729-2

3 Mccarthy B, Casey D, Devane D, *et al.* Pulmonary rehabilitation for chronic obstructive pulmonary disease. Cochrane Database Syst. Rev. 2015;**23**. doi:10.1002/14651858.CD003793.pub3

4 Keating A, Lee A, Holland AE. What prevents people with chronic obstructive pulmonary disease from attending pulmonary rehabilitation? A systematic review. Chron. Respir. Dis. 2011;**8**:89–99. doi:10.1177/1479972310393756

5 Keating A, Lee AL, Holland AE. Lack of perceived benefit and inadequate transport influence uptake and completion of pulmonary rehabilitation in people with chronic obstructive pulmonary disease: A qualitative study. *J Physiother* 2011;**57**:183–90. doi:10.1016/S1836-9553(11)70040-6

6 Blackstock FC, Wallack RZ, Nici L, *et al.* Why don't our patients with chronic obstructive pulmonary disease listen to us? the enigma of nonadherence. Ann. Am. Thorac. Soc.

For peer review only

For peer review only - http://bmjopen.bmj.com/site/about/guidelines.xhtml

2016;**13**:317–23. doi:10.1513/AnnalsATS.201509-600PS

7 NICE. Shared decision making | NICE guidelines | NICE guidance | Our programmes | What we do | About | NICE. NICE 2021. https://www.nice.org.uk/about/what-we-do/our-programmes/nice-guidance/nice-guidelines/shared-decision-making (accessed 7 Feb 2022).

8 Breckenridge K, Bekker HL, Gibbons E, *et al.* How to routinely collect data on patient-reported outcome and experience measures in renal registries in Europe: An expert consensus meeting. Nephrol. Dial. Transplant. 2015;**30**:1605–14. doi:10.1093/ndt/gfv209

9 Bekker H, Thornton J, Airey C, *et al.* Informed decision making: An annotated bibliography and systematic review. Health Technol. Assess. (Rockv). 1999;**3**. doi:10.3310/hta3010

10 Stacey D, Bennett CL, Barry MJ, *et al.* Decision aids for people facing health treatment or screening decisions. In: Stacey D, ed. *Cochrane Database of Systematic Reviews*. Chichester, UK: : John Wiley & Sons, Ltd 2011. CD001431. doi:10.1002/14651858.cd001431.pub3

11 Stacey D, Légaré F, Lewis K, *et al.* Decision aids for people facing health treatment or screening decisions. Cochrane Database Syst. Rev. 2017;**2017**. doi:10.1002/14651858.CD001431.pub5

12 Hoffman AS, Volk RJ, Saarimaki A, *et al.* Delivering patient decision aids on the Internet: Definitions, theories, current evidence, and emerging research areas. BMC Med. Inform. Decis. Mak. 2013;**13**:S13. doi:10.1186/1472-6947-13-S2-S13

13 Sepucha KR, Borkhoff CM, Lally J, *et al.* Establishing the effectiveness of patient decision aids: Key constructs and measurement instruments. *BMC Med Inform Decis Mak* 2013;**13**:S12. doi:10.1186/1472-6947-13-S2-S12

14 Feldman-Stewart D, O'Brien MA, Clayman ML, *et al.* Providing information about options in patient decision aids. *BMC Med Inform Decis Mak* 2013;**13**:S4. doi:10.1186/1472-6947-13-S2-S4

For peer review only

For peer review only - http://bmjopen.bmj.com/site/about/guidelines.xhtml

15 Jull J, Köpke S, Smith M, *et al.* Decision coaching for people making healthcare decisions. *Cochrane Database Syst Rev* 2021;**2021**. doi:10.1002/14651858.CD013385.PUB2/MEDIA/CDSR/CD013385/IMAGE_N/NCD013385-CMP-004.01.SVG

16 Légaré F, Ratté S, Stacey D, *et al.* Interventions for improving the adoption of shared decision making by healthcare professionals. In: *Cochrane Database of Systematic Reviews*. John Wiley & Sons, Ltd 2010. doi:10.1002/14651858.cd006732.pub2

17 Müller E, Diesing A, Rosahl A, *et al.* Evaluation of a shared decision-making communication skills training for physicians treating patients with asthma: A mixed methods study using simulated patients. *BMC Health Serv Res* 2019;**19**:612. doi:10.1186/s12913-019-4445-y

18 Stacey D, Kryworuchko J, Belkora J, *et al.* Coaching and guidance with patient decision aids: A review of theoretical and empirical evidence. *BMC Med Inform Decis Mak* 2013;**13**. doi:10.1186/1472-6947-13-S2-S11

19 NICE. Shared decision making learning package. 2021.https://www.nice.org.uk/guidance/ng197/resources/shared-decision-making-learning-package-9142488109 (accessed 7 Feb 2022).

20 Elwyn G, Tsulukidze M, Edwards A, *et al.* Using a 'talk' model of shared decision making to propose an observation-based measure: Observer OPTION5 Item. *Patient Educ Couns* 2013;**93**:265–71. doi:10.1016/j.pec.2013.08.005

21 Simon D, Schorr G, Wirtz M, *et al.* Development and first validation of the shared decision-making questionnaire (SDM-Q). *Patient Educ Couns* 2006;**63**:319–27. doi:10.1016/j.pec.2006.04.012

22 O'Connor A. Sample Tool: Values (Tamoxifen). 1999.www.ohri.ca/decisionaid (accessed 19 Feb 2020).

For peer review only

For peer review only - http://bmjopen.bmj.com/site/about/guidelines.xhtml

23 O'Connor AM. Sample Tool: Decision Self-Efficacy Scale. 1995.www.ohri.ca/decisionaid (accessed 19 Feb 2020).

24 O'Connor AM. Validation of a Decisional Conflict Scale. *Med Decis Mak* 1995;**15**:25–30. doi:10.1177/0272989X9501500105

25 Brehaut JC, O'Connor AM, Wood TJ, Hack TF, Siminoff L, Gordon E F-SD. Validation of a decision regret scale. *Med Decis Making* 2003;**23**:281–92.

26 Bomhof-Roordink H, Gärtner FR, van Duijn-Bakker N, *et al.* Measuring shared decision making in oncology: Development and first testing of the iSHAREpatient and iSHAREphysician questionnaires. *Heal Expect* 2020;**23**:496–508. doi:10.1111/HEX.13015

27 Kew KM, Malik P, Aniruddhan K NR. Shared decision-making for people with asthma. *Cochrane Database Syst Rev* 2017.

28 Malone H, Biggar S, Javadpour S, *et al.* Interventions for promoting participation in shared decision-making for children and adolescents with cystic fibrosis. *Cochrane Database Syst Rev* 2019;**2019**. doi:10.1002/14651858.CD012578.pub2

29 Cochrane. Data extraction forms | Cochrane Developmental, Psychosocial and Learning Problems. https://dplp.cochrane.org/data-extraction-forms (accessed 16 Mar 2021).

30 Sterne JAC, Savović J, Page MJ, *et al.* RoB 2: A revised tool for assessing risk of bias in randomised trials. *BMJ* 2019;**366**:l4898. doi:10.1136/bmj.l4898

31 Sterne JA, Hernán MA, Reeves BC, *et al.* ROBINS-I: a tool for assessing risk of bias in non-randomised studies of interventions. *BMJ* 2016;**355**. doi:10.1136/BMJ.I4919

32 J P, H R, A S, *et al.* Guidance on the conduct of narrative synthesis in systematic reviews. A product from the ESRC methods programme. Version, 1.No Title. 2006. doi:10.13140/2.1.1018.4643

For peer review only

For peer review only - http://bmjopen.bmj.com/site/about/guidelines.xhtml

33 Au DH, Udris EM, Engelberg RA, *et al.* A randomized trial to improve communication about end-of-life care among patients with COPD. *Chest* 2012;**141**:726–35. doi:10.1378/chest.11-0362

34 Brundage MD, Feldman-Stewart D, Cosby R, *et al.* Phase I study of a decision aid for patients with locally advanced non-small-cell lung cancer. *J Clin Oncol* 2001;**19**:1326–35. doi:10.1200/JCO.2001.19.5.1326

35 Collinsworth AW, Brown RM, Sjames C, *et al.* The impact of patient education and shared decision making on hospital readmissions for COPD. *Int J COPD* 2018;**13**:1325–32. doi:10.2147/COPD.S154414

36 Gagné ME, Légaré F, Moisan J, *et al.* Impact of Adding a Decision Aid to Patient Education in Adults with Asthma: A Randomized Clinical Trial. *PLoS One* 2017;**12**:e0170055. doi:10.1371/journal.pone.0170055

37 Granados-Santiago M, Valenza MC, López-López L, *et al.* Shared decision-making and patient engagement program during acute exacerbation of COPD hospitalization: A randomized control trial. *Patient Educ Couns* 2019;**103**:702–8. doi:10.1016/j.pec.2019.12.004

38 Walters J, Cameron-Tucker H, Wills K, *et al.* Effects of telephone health mentoring in community-recruited chronic obstructive pulmonary disease on self-management capacity, quality of life and psychological morbidity: A randomised controlled trial. *BMJ Open* 2013;**3**. doi:10.1136/bmjopen-2013-003097

39 Wilson SR, Strub P, Buist AS, *et al.* Shared treatment decision making improves adherence and outcomes in poorly controlled asthma. *Am J Respir Crit Care Med* 2010;**181**:566–77. doi:10.1164/rccm.200906-0907OC

40 Myers RE, Advani SM, Myers P, *et al.* Engaging Patients with Late-Stage Non-Small Cell Lung Cancer in Shared Decision Making about Treatment. *J Pers Med* 2021;**11**.

For peer review only

doi:10.3390/JPM11100998

41 Coulter A, Stilwell D, Kryworuchko J, *et al.* A systematic development process for patient decision aids. *BMC Med Inform Decis Mak* 2013;**13**:S2. doi:10.1186/1472-6947-13-S2-S2

42 Stacey D, Légaré F, Boland L, *et al.* 20th Anniversary Ottawa Decision Support Framework: Part 3 Overview of Systematic Reviews and Updated Framework. Med. Decis. Mak. 2020;**40**:379–98. doi:10.1177/0272989X20911870

43 O'Cathain A, Croot L, Duncan E, *et al.* Guidance on how to develop complex interventions to improve health and healthcare. *BMJ Open* 2019;**9**:e029954. doi:10.1136/bmjopen-2019-029954

44 Mccaffery KJ, Holmes-Rovner M, Smith SK, *et al.* Addressing health literacy in patient decision aids. 2012. doi:10.1186/1472-6947-13-S2-S10

45 Muscat DM, Shepherd HL, Nutbeam D, *et al.* Health Literacy and Shared Decision-making: Exploring the Relationship to Enable Meaningful Patient Engagement in Healthcare. *J Gen Intern Med* 2020;**36**:521–5. doi:10.1007/s11606-020-05912-0

46 Parker RM, Baker DW, Willia M V., *et al.* The test of functional health literacy in adults. *J Gen Intern Med 1995 1010* 1995;**10**:537–41. doi:10.1007/BF02640361

47 Plain English Campaign. http://www.plainenglish.co.uk/ (accessed 6 Aug 2021).

48 Linder SK, Swank PR, Vernon SW, *et al.* Validity of a low literacy version of the Decisional Conflict Scale. *Patient Educ Couns* 2011;**85**:521–4. doi:10.1016/j.pec.2010.12.012

49 Chen Z, Fan VS, Belza B, *et al.* Association between Social Support and Self-Care Behaviors in Adults with Chronic Obstructive Pulmonary Disease. *Ann Am Thorac Soc* 2017;**14**:1419–27. doi:10.1513/AnnalsATS.201701-026OC

50 Lind N, Nordin M, Palmquist E, *et al.* Coping and social support in asthma and allergy: the

For peer review only

For peer review only - http://bmjopen.bmj.com/site/about/guidelines.xhtml

603 Västerbotten Environmental Health Study. *J Asthma* 2015;**52**:622–9.

604 doi:10.3109/02770903.2014.991970

605 51 Janssen DJA, Curtis JR, Au DH, *et al.* Patient-clinician communication about end-of-life care

606 for Dutch and US patients with COPD. *Eur Respir J* 2011;**38**:268–76.

607 doi:10.1183/09031936.00157710

608 52 Page MJ, McKenzie JE, Bossuyt PM, *et al.* The PRISMA 2020 statement: An updated guideline

609 for reporting systematic reviews. BMJ. 2021;**372**. doi:10.1136/bmj.n71

For peer review only

Figure legends

Figure I: MIND-IT model; a framework representing informed, evidence-based and shared decisions with patients and their healthcare professional (Bekker et al., 2015)

Figure II: PRISMA flow diagram of review process

Figure III: SDM intervention principles aligned to the MIND-IT model (Bekker et al., 2015)

Figure IV: Risk of bias in RCT studies

Figure V: Risk of bias in non-RCT studies

Table I: Summary of studies

Table II: Summary of interventions

For peer review only

For peer review only - http://bmjopen.bmj.com/site/about/guidelines.xhtml

Tables

Table I: Summary of studies

**First Author, Year, Country**	**Sample size**	**Study design**	**Participant population**	**Outcome(s)**	**Summary of results**	**Certainty of evidence (GRADE score)**
**Au, 2012, USA**	Clinicians = 92<br>Patients = 376	RCT	Outpatients with COPD	*Validated:*<br>Quality of communication (QOC). 19 items scored from 0-100 (higher score=better communication)<br><br>*Non-validated:* Occurrence of discussions about end of life preferences between patients and either clinician or surrogate. 4-item self-reported questionnaire scored from 0-100% (higher % =greater occurrence).	Baseline quality of communication was poor in both groups. Modest improvements in both groups, but significant between group difference favouring the intervention group (5.7 points; p<0.05).<br><br>The occurrence (%) of patient communication about end of life care was significantly higher in the intervention group for three of four questions:<br>1. Int: 35.2(74.6) Con: 15.9(58.2) p<0.05<br>2. Int: 60.3(122.9) Con: 30.8(175.5) p<0.05<br>3. Int: 53.6(115.1) Con: 45.2(73.3) p>0.05<br>4. Int: 86.2(84.6) Con: 75.2(115.0) p<0.05	Moderate ⊕⊕⊕◯ Moderate certainty because of risk of bias.
**Brundage, 2001, Canada**	Patients = 20	Feasibility CBA	Outpatients with lung cancer	*Validated:*<br>Decisional conflict (DCS). 16 item scale using a 4-point Likert scale anchored with strongly agree and strongly disagree. Converted scale scores >37.5 indicate meaningful decisional conflict or delayed decision implementation.<br><br>*Non-validated:*<br>Knowledge of treatment options. 3 open-ended questions.<br><br>Strength of treatment preference. 1 item using a 7-point Likert scale anchored with definitely wish treatment 1 and definitely wish treatment 2.	Decisional conflict reduced after receiving the intervention in 87% and increased in 13% of participants.<br><br>Knowledge of the available treatment options improved following the intervention for both 3-year outcome survival[a] and median survival outcome[b]:<br>1. Knowledge of survival outcomes with treatment 1 alone (between group difference 60%[a]; 54%[b])<br>2. Knowledge of direction of survival difference between treatments (between group difference: 27%[a]; 40%[b])<br>3. Knowledge of the magnitude of survival difference between treatments (between group difference: 73%[a]; 67%[b]).<br><br>The number of participants with a treatment preference increased from 80% to 100% following the intervention.	Low ⊕⊕◯◯ Low certainty because of risk of bias and minor publication bias.
**Collinsworth, 2018, USA**	Patients = 308	RCT	Inpatients with COPD	*Validated:*<br>Patient activation (PAM). A 22 items scale using a 4-point Likert scale anchored with patient is disengaged and	Both groups had a significant improvement in PAM scores following the intervention (difference: 0.52(0.9) and 0.69(1.0) points respectively, p<0.05).	Moderate ⊕⊕⊕◯ Moderate certainty

For peer review only - http://bmjopen.bmj.com/site/about/guidelines.xhtml

				overwhelmed and patient has adopted and is maintaining healthy behaviours.<br><br>Quality of life health status (CAT). An 8 item scale with scores ranging from 0-40. Lower scores (<10) indicate COPD causes few problems and higher scores (>30) indicate COPD prevents patients from doing most things they want to do.<br><br>*Non-validated:*<br>All-cause hospital admissions extracted from electronic health records. Reported as frequency count.	Only patients in the intervention group had significantly increased scores in the CAT following the intervention (difference: Int: 5.27(10.3), p<0.05; Con: 0.38(7.8), p>0.05).<br><br>There were no significant differences in the total number of all-cause or COPD-related readmissions between groups at 1, 2, 3, 6 and 9 months (p>0.05).	because of risk of bias.
**Gagne, 2017, Canada**	Patients = 51	RCT	Outpatients with asthma	*Validated:*<br>Asthma knowledge (QCALF). 37 item scale with scores ranging from -37 to +37 (higher scores indicate greater knowledge)<br><br>Decisional conflict (DCS). 16 item scale using a 4-point Likert scale anchored with strongly agree and strongly disagree. Converted scale scores >37.5 indicate meaningful decisional conflict or delayed decision implementation.<br><br>*Non-validated:*<br>Appropriate use of asthma pharmacotherapy. A 4-item face-to-face interviewer-administered questionnaire. The cut off for appropriate use was meeting 11 hierarchical criteria.	Both groups had significantly increased knowledge scores following the intervention (difference: Int: 3.6(8.9)*, p<0.001; Con: 2(7.3)*, p<0.05). There was no significant between group difference (p>0.05).<br><br>Both groups had significantly reduced decisional conflict scores following the intervention (difference: Int: 8.1(22.5)*, p<0.05; Con: 9.1(27.7)*, p>0.05). There was no significant between group difference (p>0.05).<br><br>Both groups had modest, but non-significant, within group improvements in their appropriate use of pharmacotherapy (difference: Int: 0.15(0.7)*, p>0.05; Con: 0.16(0.7)*, p>0.05). There was no significant between group difference (between group difference = 0.17(0.7), p>0.05).	Moderate<br>⨁⨁⨁◯<br>Moderate certainty because of risk of bias.
**Granados-Santiago, 2019, Spain**	Patients = 42	RCT	Inpatients with COPD	*Validated:*<br>Quality of life and health status (EQ-5D). A 5-item scale with scores ranging from 0 (defined as the worst imaginable health state) to 100 (defined as the best imaginable health state).	Both groups achieved a significant and clinically important improvement in their quality of life and health status at discharge (between group difference: 6.15(27.5)*, p<0.05) but only the intervention group maintained this benefit 3 month later (between group difference: 8.28(50.6)*, p<0.05).	Moderate<br>⨁⨁⨁◯<br>Moderate certainty because of risk of bias.

For peer review only - http://bmjopen.bmj.com/site/about/guidelines.xhtml

				COPD knowledge (COPD-Q). A 13 item scale with scores ranging from 0 (defined as lowest knowledge) to 13 (defined as highest knowledge).<br><br>Medication adherence (TAI). A 10 item questionnaire, with higher scores indicating greater adherence.<br><br>Physical activity (steps/day) measured using the Fitbit Flex 2 Pedometer.<br><br>Nutritional status (MNA). An 18 item questionnaire. Scores lower than 17 are considered malnutrition<br><br>*Non-validated:*<br>-	Knowledge scores significantly improved in the intervention group alone at discharge (between group difference: 3.89(0.7)*, p<0.05) and maintained at 3 months follow up (between group difference: 3.88(0.8)*, p<0.05).<br><br>Adherence significantly improved at discharge in the intervention group alone (difference: Int: 3.58(1.5)*, p<0.05; Con: 1.36(1.6)*, p>0.05) but there was no significant between group difference. At 3 months post intervention there was a significant between group difference favouring the intervention group (1.8(2.4)*, p<0.05).<br><br>Upon discharge, there was a significant reduction in physical activity in both groups (difference: Int: -348.27(2.0)*, p<0.05; Con: -410.05(4.66)*, p<0.05). At 3 months post intervention there was a significant improvement in the intervention group alone (difference: Int: 1371.97(895.2)*, p<0.05; Con: 620.18(12.5)*, p>0.05).<br><br>At 3 months post intervention there was a significant improvement in nutritional status both within and between groups for the intervention group alone (difference: Int: 4.15(0.1)*, p<0.05; Con: -3.43(0.12)*, p>0.05	
**Myers, 2021, USA**	Patients = 5	Pilot CBA	Outpatients with non-small cell lung cancer	*Validated:*<br>Decisional Conflict (DCS SURE Test). A 4-item scale with scores from 0-4, a score <4 indicates clinically significant decisional conflict.<br><br>*Non-validated:*<br>Knowledge of treatment options. 3-item survey using a 3-point Likert scale (Yes/No/Unsure) to elicit patient beliefs regarding curability of treatment options, awareness of treatment options, and treatment costs. Awareness of treatment options only item included in this analysis as only item measuring knowledge.	Decisional conflict reduced post intervention (mean difference=1).<br><br>Awareness of treatment options increased from 40% to all 100% participants post intervention.	Low ⊕⊕◯◯ Low certainty because of risk of bias.

For peer review only - http://bmjopen.bmj.com/site/about/guidelines.xhtml

				Treatment status. 30-day treatment status extracted from electronic health records. This was compared to treatment preferences immediately post intervention.	At 30 days post intervention, 80% of participants had made a decision. 40% of participants had a treatment that matched their treatment preference.	
**Walters, 2013, Australia**	Patients = 182	Cluster RCT	Outpatients with COPD	*Validated:*<br>Quality of life (SF-36). An 8 item scale with scores from 0-100, higher score indicates better quality of life.<br><br>Quality of life (SGRQ). A 76 items scale with scores ranging from 0-100, higher score indicates worse quality of life.<br><br>Patient involvement in healthcare decisions (PIH). A 12 item scale with scores ranging from 0-8 (higher score indicates more involvement).<br><br>*Non-validated:*<br>Respiratory hospital admissions extracted from electronic health records. Reported as frequency count.<br><br>Intervention fidelity. Recorded telephone calls from the first three intervention and 10% of control contacts were coded by two raters for content and fidelity to prespecified components of the intervention to assess adherence. To ensure concordance, both raters coded a random sample of 20 calls.	Neither group had a significant change to either their physical[a] or mental[b] health status at 6 months (between group difference: 1.5(0.2)*[a]; 0.8(1.4)*[b], p>0.05) and 12 months (between group difference: 0.0(0.9)*[a]; 0.3(0.9)*[b], p>0.05) post intervention.<br><br>Neither group had a significant change to their overall quality of life at 6 months (between group difference: 1.9(2.7)*, p>0.05) and 12 months (between group difference: 1.4(1.5)*, p>0.05 post intervention.<br><br>Only the intervention group had a significant improvement in PIH scores at 6 months (between group difference: 0.4(0.2)*, p<0.05) and 12 months (between group difference: 0.3(0.1)*, p<0.05) post intervention.<br><br>No significant change in the number of hospital admissions for either group over the 12 months study period (χ^2=2.61, p>0.05).<br><br>Concordance between two raters was high (overall weighted κ 0.72). Fidelity assessments confirmed specific health mentoring components were addressed with some clarity in the following proportions:<br>1. COPD symptom management, 49%<br>2. Unhelpful self-talk explored and identified, 37%<br>3. Unhelpful self-talk challenged, and new self-talk developed, 35%<br>4. Action plan for achieving goals made, 54%<br>5. Problems and barriers to achieving goals identified and clarified, 46%<br>6. Positive changes in behaviour praised, 83%. Raters concluded there was a lower delivery of cognitive behavioural components of intervention.	Very low<br>⊕○○○<br>Very low certainty because of significant risk of bias and some inconsistency.

For peer review only - http://bmjopen.bmj.com/site/about/guidelines.xhtml

**Wilson, 2010, Hawaii**	Patients = 612	RCT	Outpatients with asthma	*Validated:*<br>Quality of life (MiniAQLQ). A 5 item scale with Likert scale ranging from 0 (all of the time) to 7 (none of the time).<br><br>*Non-validated:*<br>Healthcare use extracted from electronic health records. Reported as frequency count.<br><br>Patients' perceived role in treatment decision. A 1 item scale using a 5-point Likert scale ranging from 0 (low involvement in decision-making) to 5 (high involvement in treatment decision-making).<br><br>Medication adherence. Data extracted from pharmacy records to calculate a continuous medication acquisition index for each year.<br><br>Intervention fidelity. Audiotapes of both intervention groups of 10% of patients were scored on a detailed performance checklist to determine whether the two protocols were delivered as intended. Scores ranged from 0-4 with higher scores indicated greater adherence.	Both intervention groups, and not the control group, had a significant improvement in quality of life scores at 12 months post intervention (SDM to control = 0.39(1.0)*, p<0.05; SDM–clinician decision-making = 0.11(1.1)*, p>0.05; clinician decision-making-control= 0.28(1.0)*, p<0.05.<br><br>Both intervention groups, and not the control group, had a significantly reduced healthcare utilisation (SDM-control =20.36(-1.5)*, p<0.05; SDM-clinician decision-making=0.01(2.7)*, p>0.05; clinician decision-making-control= 20.37(-1.5*), p<0.05.<br><br>The SDM group rated their influence on the treatment decision as being approximately the same as the clinicians' influence (3.1(0.6). The SDM group ratings were significantly different from the clinician decision-making group, with the latter feeling that their clinician had a greater influence (2.5(0.9), p<0.05) than they did themselves.<br><br>Medication adherence significantly increased in both intervention groups but not the control group (SDM-control=0.21(0.5)*, p<0.05; SDM–clinician decision-making = 0.08(0.4)*, p>0.05; CDM-control = 0.13(0.4)*, p<0.05. This was not sustained at 2 years post intervention (SDM-control = 0.03(0.4)*, p>0.05; SDM-clinician decision-making = 0.04(0.4)*, p>0.05; clinician decision-making-control =-0.01(-0.4)*, p>0.05.<br><br>Protocol adherence scores assigned by the raters were high (SDM=4.0, clinician decision-making=3.9) and did not differ significantly between the two groups (p>0.05).	Moderate<br>⊕⊕⊕○<br>Moderate certainty because of risk of bias.

Int = Intervention
Con = Control
RCT = randomised controlled trial
CBA = Controlled before and after study

For peer review only - http://bmjopen.bmj.com/site/about/guidelines.xhtml

Table II: Summary of interventions

First Author (Year)	Description of intervention	Interventionist (including any training provided)	Intervention dose	Description of control group
**Au (2012)**	Consultation using a completed patient-specific feedback form which describes patient preferences regarding end of life care. The feedback form was not endorsed for use.	Clinicians.<br>No specific training described.	Single session.	Routine outpatient COPD care.
**Brundage (2001)**	Consultation using a patient decision aid to facilitate cancer treatment decision-making.	Researcher.<br>No specific training described.	Single session.	Participant outcomes prior to receipt of intervention.
**Collinsworth (2018)**	COPD education and SDM self-management planning.	Registered respiratory therapists.<br>No specific training described.	Single session and 4 telephone calls.	Routine inpatient care and COPD education.
**Gagne (2017)**	Patient decision aid to facilitate asthma treatment decision-making (and asthma education).	Certified asthma educators from the Quebec Asthma and COPD Network.	Single session.	Routine outpatient asthma care, education, and action plan generation.
**Granados-Santiago (2019)**	A SDM patient involvement programme focussing on COPD self-management goals to facilitate self-management decision-making.	Healthcare professionals.<br>No specific training described.	Delivered alongside routine inpatient care.	Routine inpatient care for an exacerbation of COPD.
**Myers (2021)**	Personalised patient decision aid used to facilitate 15-20min decision coaching consultation.	Oncology nurses.<br>No training provided.	Single session.	Participant outcomes prior to receipt of intervention.
**Walters (2013)**	Psychoeducation about common psychological reactions to COPD diagnosis and treatment, self-management skills training, cognitive coping skills training to identify and challenge negative COPD-related cognitions, communication skills to facilitate discussion between the health mentor and the patient, and promoting self-efficacy to manage chronic illness.	Community health nurses, termed 'Health Mentors.'<br>Training included 12 hours of training over 2 days that covered COPD management (1 h), chronic disease self-management and health behaviour change components including practice role plays (7.25 hours), online training and study methods (3.75 hours).	Single session and 16 telephone calls.	Routine outpatient COPD care and monthly telephone calls for 12 months (excluded intervention components).
**Wilson (2010)**	*Shared decision-making intervention:*	Healthcare professionals involved in routine asthma care (nurses,	Two sessions and 3 telephone calls.	Routine asthma outpatient care (including, in some sites, the

For peer review only - http://bmjopen.bmj.com/site/about/guidelines.xhtml

	Consultation to facilitate asthma treatment decision-making followed by 4 contacts to assess patient progress and medication changes as needed.	respiratory therapists, pharmacists, nurse practitioners and physician assistants).<br>Training provided but not described.		opportunity to of referral to an asthma care a management programme).
	Clinician decision-making intervention:<br>Consultation for clinicians to obtains a patient's level of asthma control, prescribe an appropriate treatment regimen and communicate that to the patient.	Healthcare professionals involved in routine asthma care (nurses, respiratory therapists, pharmacists, nurse practitioners and physician assistants).<br>Training provided but not described.	Two sessions and 3 telephone calls.	

For peer review only - http://bmjopen.bmj.com/site/about/guidelines.xhtml

623 **Figures**

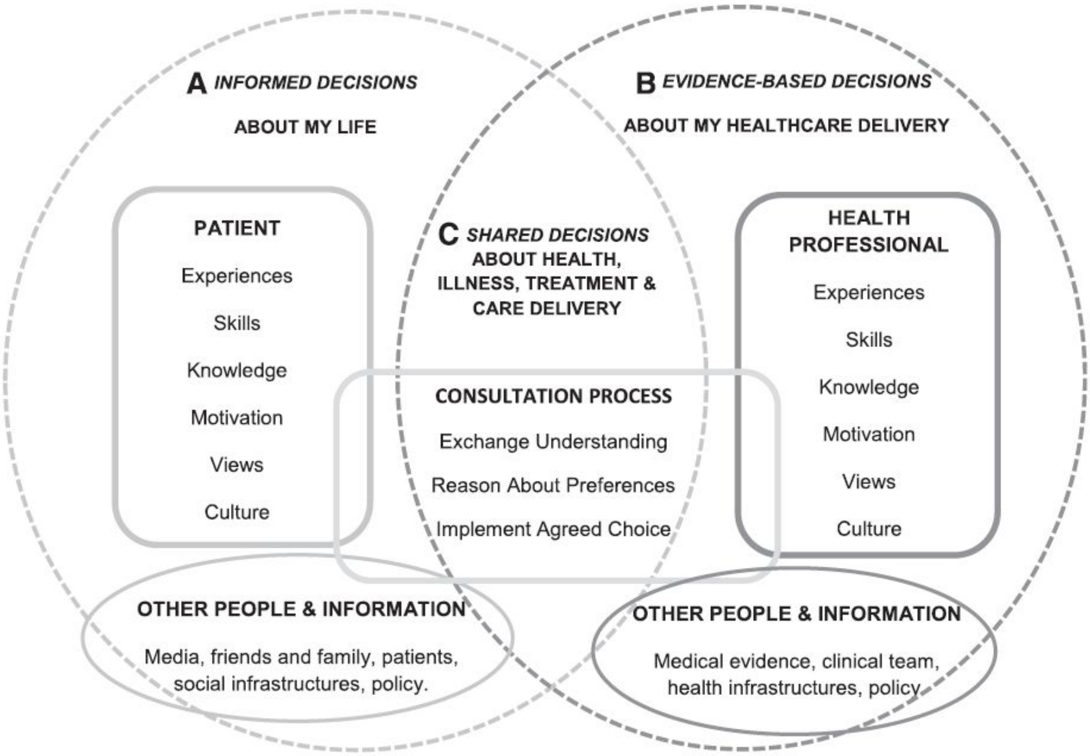

624

review only

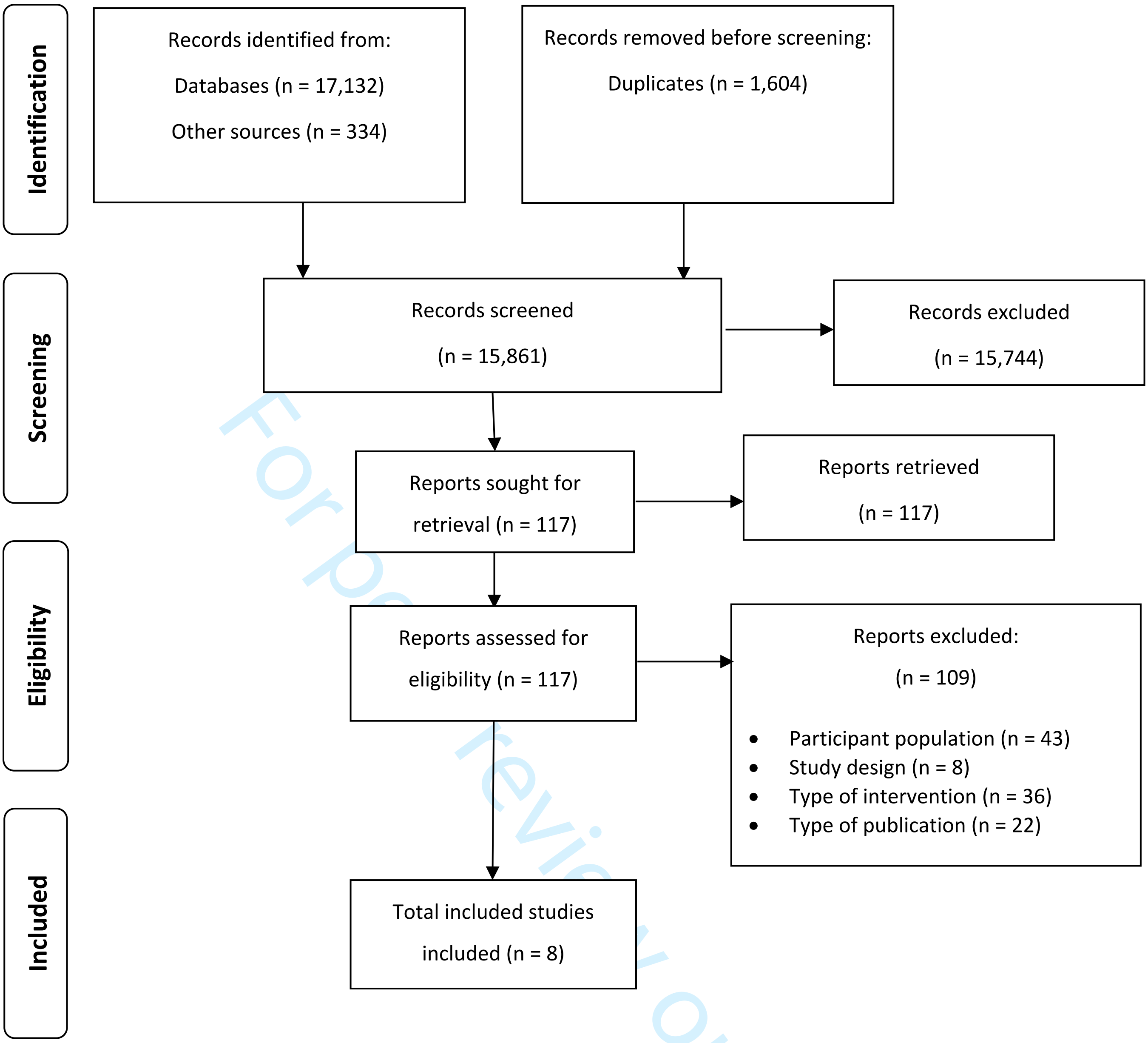

For peer review only

For peer review only - http://bmjopen.bmj.com/site/about/guidelines.xhtml

SDM Systematic Review

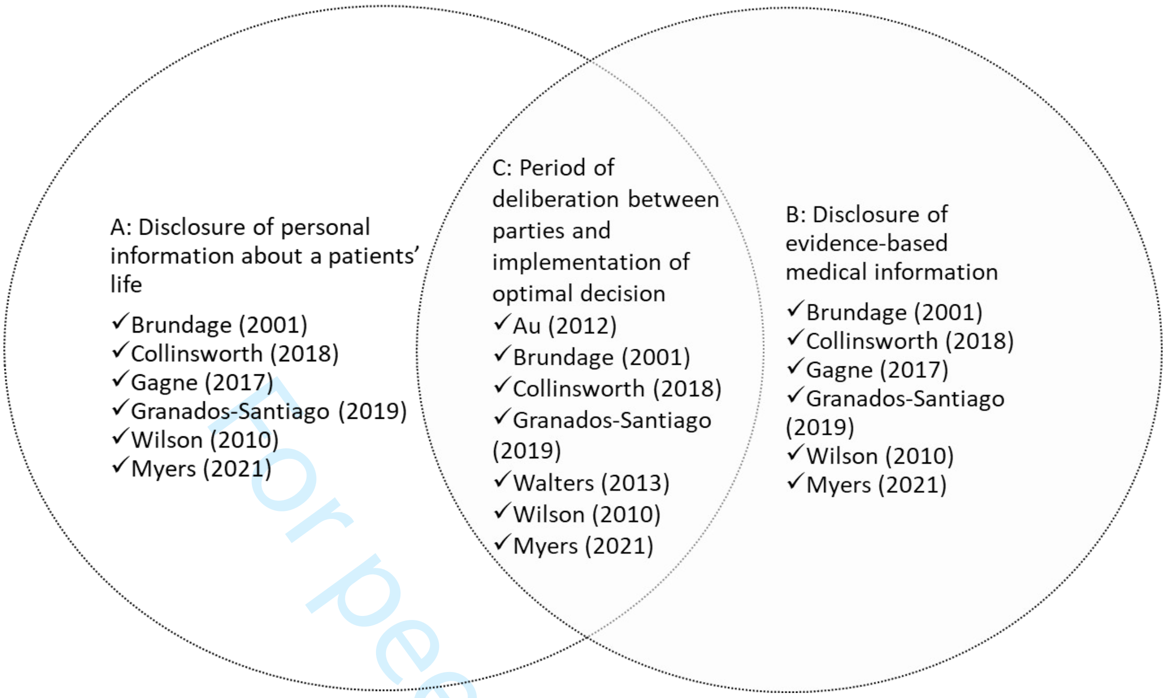

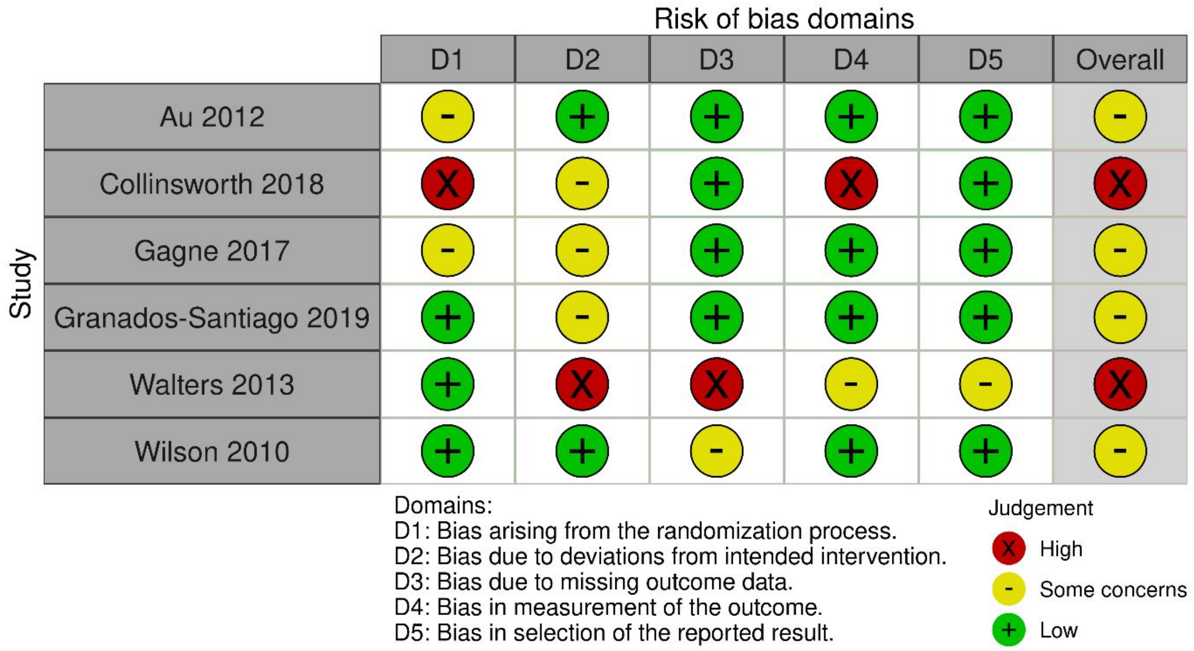

For peer review only - http://bmjopen.bmj.com/site/about/guidelines.xhtml

SDM Systematic Review

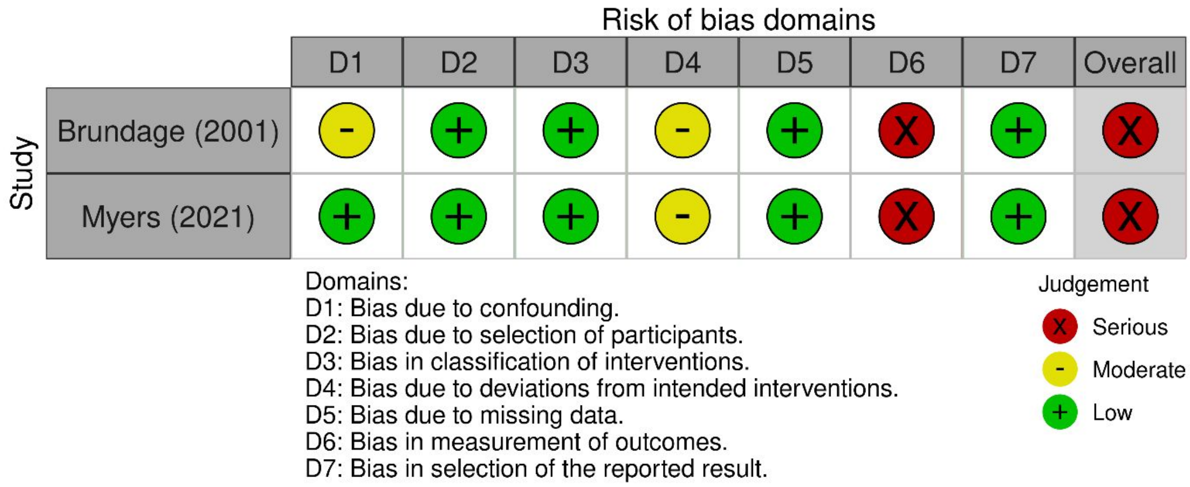

For peer review only - http://bmjopen.bmj.com/site/about/guidelines.xhtml

Supplementary Material

Title:

A systematic review of shared decision-making interventions for people living with chronic respiratory diseases

Authors:

Barradell, AC; Gerlis, C; Houchen-Wolloff L; Bekker, HL; Robertson, N; Singh, SJ.

For peer review only

For peer review only - http://bmjopen.bmj.com/site/about/guidelines.xhtml

Supplementary material for Methods:

MEDLINE search strategy

1. ((joint* or share* or sharing* or informed*) adj3 (decision* or decid*3 or choice*)).mp. [mp=title, abstract, original title, name of substance word, subject heading word, floating sub-heading word, keyword heading word, organism supplementary concept word, protocol supplementary concept word, rare disease supplementary concept word, unique identifier, synonyms]
2. "DECISION MAKING, SHARED"/
3. exp "DECISION MAKING"/
4. "DECISION SUPPORT TECHNIQUES"/
5. DECISION SUPPORT SYSTEMS, CLINICAL/
6. ((decision* or choice*) and (making* or support* or behavior* or behaviour*)).ti,ab.
7. PATIENT PARTICIPATION/
8. ((patient* or consumer*) adj3 (involvement* or involving* or participation* or participating*)).ti,ab.
9. "PATIENT-CENTERED CARE"/
10. "PROFESSIONAL-PATIENT RELATIONS"/
11. "PHYSICIAN-PATIENT RELATIONS"/
12. ((patient* or person* or client* or consumer*) adj3 (centred or centered or focused or oriented)).ti,ab.
13. "decision tool*".ti,ab.
14. "clinical counsel*".ti,ab.
15. "patient decision guide*".ti,ab.
16. "decision coaching".ti,ab.
17. "decision aid*".ti,ab.
18. or/1-17
19. LUNG DISEASES, OBSTRUCTIVE/
20. exp PULMONARY DISEASE, CHRONIC OBSTRUCTIVE/
21. emphysema*.ti,ab.
22. (chronic* adj3 bronchiti*).ti,ab.
23. (obstruct* adj3 (pulmonary or lung* or airway* or airflow* or bronch* or respirat*)).ti,ab.
24. (coad or cobd or copd or aecb).ti,ab.
25. asthma*.ti,ab.
26. exp ASTHMA/
27. (idiopathic and pulmonary and fibrosis).ti,ab.
28. IDIOPATHIC PULMONARY FIBROSIS/
29. (interstitial and lung and disease).ti,ab.
30. LUNG DISEASES, INTERSTITIAL/
31. (extrinsic and allergic and alveolitis).ti,ab.
32. ALVEOLITIS, EXTRINSIC ALLERGIC/

For peer review only - http://bmjopen.bmj.com/site/about/guidelines.xhtml
For peer review only

33. (non and specific and interstitial and (pneumonitis or NSIP)).ti,ab.

34. (Usual and interstitial and (pneumonia or UIP)).ti,ab.

35. "LUNG DISEASES, OBSTRUCTIVE"/

36. ((lung or pulmonary or airway) adj disease).ti,ab.

37. "cystic fibrosis".ti,ab.

38. CYSTIC FIBROSIS/

39. "SARCOIDOSIS, PULMONARY"/

40. sarcoidosis.ti,ab.

41. exp "LUNG NEOPLASMS"/

42. (lung adj (cancer* or tumo?r* or neoplas*)).ti,ab.

43. "TUBERCULOSIS, PULMONARY"/

44. tuberculosis.ti,ab.

45. BRONCHIECTASIS/

46. bronchiectasis.ti,ab.

47. exp PNEUMOCONIOSIS/

48. (autoimmune adj3 (lung or airway or pulmonary)).ti,ab.

49. or/19-48

50. randomized controlled trial.pt.

51. controlled clinical trial.pt.

52. randomized.ab.

53. placebo.ab.

54. clinical trials as topic.sh.

55. randomly.ab.

56. trial.ti.

57. or/50-56

58. exp ANIMALS/ not humans.sh.

59. 57 not 58

60. 59 and 18 and 49

Data items

Extracted items included: population (age, sex, health literacy skill, diagnosis, disease severity, length of diagnosis, baseline measures, total number randomised), intervention (group names, group sample size, adherence to SDM principles, adherence to complex intervention development guidelines, content, training, timing, frequency, duration and any other interventions), comparator (group names, group sample size, content, training, timing, frequency, duration) and outcomes (type, unit of analysis, reported missing data). Data was also extracted on each study’s objectives, study design, unit of allocation, decision posed (e.g. medication, pulmonary rehabilitation, surgery, end of life), duration of the study and follow up.

For peer review only - http://bmjopen.bmj.com/site/about/guidelines.xhtml

Sub-group analysis

The following additional data was collected for sub-group analysis: diagnosis, characteristics (age, sex, health literacy skill), decision posed (medication, pulmonary rehabilitation, surgery, end of life), intervention dose (number and length of sessions) and length of follow-up.

For peer review only

For peer review only - http://bmjopen.bmj.com/site/about/guidelines.xhtml

Supplementary Material for Results:

Table 1: Characteristics of excluded studies

Lead Author	Year	Reason for exclusion	Categorised reason for exclusion
Aaron[1]	2006	Abstract only (results in Vandemheen 2009)	Type of publication
Aboumatar[2]	2017	No results in paper (in Aboumatar 2019)	Type of publication
Aboumatar[3]	2019	No SDM within intervention	Type of intervention
Braun[4]	2005	Abstract only (in Menon 2016)	Type of publication
Brown [5]	2012	CRD participant data not distinguishable from analysis	Participant population
Brown [6]	2004	CRD participant data not distinguishable from analysis	Participant population
Brundage[7]	1998	CRD participant data not distinguishable from analysis	Participant population
Butow[8]	2015	No CRD participants	Participant population
Carlucci [9]	2016	No comparator group	Study design
Cook[10]	2016	No adult CRD participants	Participant population
Coultas[11]	2005	No SDM within intervention	Type of intervention
Cvietusa[12]	2020	No SDM within intervention	Type of intervention
Daly[13]	2013	CRD participant data not distinguishable from analysis	Participant population
Detmar[14]	2002	CRD participant data not distinguishable from analysis	Participant population
Dyar[15]	2012	CRD participant data not distinguishable from analysis	Participant population
Early[16]	2015	No SDM within intervention	Type of intervention
Fiks[17]	2015	No CRD participants	Participant population
Fors[18]	2018	CRD participant data not distinguishable from analysis	Participant population
Foster[19]	2016	No SDM within intervention	Type of intervention
Foster[20]	2014	No more than one choice	Type of intervention

For peer review only - http://bmjopen.bmj.com/site/about/guidelines.xhtml

Fraenkel[21]	2016	No CRD participants	Participant population
Gagne[22]	2016	Abstract only (results in Gagne 2017)	Type of publication
George[23]	2017	Abstract only - (results in George 2020)	Type of publication
George[24]	2020	No more than one choice	Type of intervention
Gupta[25]	2019	No SDM within intervention	Type of intervention
Hagan[26]	2008	No SDM within intervention	Type of intervention
Halley[27]	2015	No CRD participants	Participant population
Hilberink[28]	2011	No SDM within intervention	Type of intervention
Hoerger[29]	2017	CRD participant data not distinguishable from analysis	Participant population
Hollen[30]	2013	No comparator group	Study design
Hoskins[31]	2016	No SDM within intervention	Type of intervention
Houben[32]	2019	No SDM within intervention	Type of intervention
Hyatt[33]	2018	CRD participant data not distinguishable from analysis	Participant population
Izumi[34]	2018	CRD participant data not distinguishable from analysis	Participant population
Jonsdottir[35]	2015	No SDM within intervention	Type of intervention
Kass[36]	2009	CRD participant data not distinguishable from analysis	Participant population
Kennedy[37]	2013	CRD participant data not distinguishable from analysis	Participant population
Koerner[38]	2014	No CRD participants	Participant population
Krug[39]	2021	No SDM within intervention	Type of intervention
Lainscak[40]	2013	No SDM within intervention	Type of intervention
Latimer[41]	2007	CRD participant data not distinguishable from analysis	Participant population
Lavoie[42]	2014	No SDM within intervention	Type of intervention
Leung[43]	2012	Cross-sectional study	Study design
Lim[44]	2021	Hypothetical decision making	Type of intervention

For peer review only - http://bmjopen.bmj.com/site/about/guidelines.xhtml

Llanos-Paez[45]	2021	No SDM within intervention	Type of intervention
Lopez-Lopez[46]	2020	No SDM within intervention	Type of intervention
Ludden[47]	2018	Adult CRD participant data not distinguishable from analysis	Participant population
Makimoto[48]	2020	Letter to editor	Type of publication
Markman[49]	2005	No comparator group	Study design
Markun[50]	2018	No participant-facing content	Type of intervention
Mayer[51]	2016	CRD participant data not distinguishable from analysis	Participant population
Meline[52]	2021	No CRD participants	Participant population
Melzer[53]	2018	No SDM within intervention	Type of intervention
Menon[54]	2016	CRD participant data not distinguishable from analysis	Participant population
Meropol[55]	2013	CRD participant data not distinguishable from analysis	Participant population
Moullec[56]	2012	No SDM within intervention	Type of intervention
Murray[57]	2010	CRD participant data not distinguishable from analysis	Participant population
Nedjat-Haiem[58]	2017	CRD participant data not distinguishable from analysis	Participant population
Nugent[59]	2018	No comparator group	Study design
Patel[60]	2019	No participant-facing content	Type of intervention
Rabow[61]	2004	CRD participant data not distinguishable from analysis	Participant population
Rea[62]	2004	No SDM within intervention	Type of intervention
Reddel[63]	2019	Abstract only (results not yet published)	Type of publication
Reinke[64]	2017	No SDM outcomes	Outcome measures
Rhee[65]	2008	Participants <18years old	Participant population
Roelands[66]	2004	No CRD participants	Participant population
Rosemann[67]	2013	Abstract only (results in Steurer 2017)	Type of publication
Ruparel[68]	2019	No CRD participants	Participant population

Supplementary material V1.0

Rutten-vanMolken[69]	2017	Abstract only - (results in Slok 2016)	Type of publication
Sands[70]	2014	Abstract only - (results not yet published)	Type of publication
Schofield[71]	2013	No SDM within intervention	Type of intervention
Schubart[72]	2015	CRD participant data not distinguishable from analysis	Participant population
Schumacher[73]	2002	No quantitative data	Study design
Schwarze[74]	2020	CRD participant data not distinguishable from analysis	Participant population
Shade[75]	2019	Adult CRD participant data not distinguishable from analysis	Participant population
Shirai[76]	2012	CRD participant data not distinguishable from analysis	Participant population
Sinclair[77]	2017	No SDM within intervention	Type of intervention
Skorstengaard[78]	2019	CRD participant data not distinguishable from analysis	Participant population
Slok[79]	2016a	Abstract only (results in Slok 2016b)	Type of publication
Slok[80]	2016b	No SDM within intervention	Type of intervention
Smidth[81]	2013	No comparator group	Study design
Steinhauser[82]	2008	CRD participant data not distinguishable from analysis	Participant population
Steurer[83]	2017	Abstract only (results in Steurer 2015)	Type of publication
Steurer-Stey[84]	2015	No SDM within intervention	Type of intervention
Sullivan[85]	2020	Abstract only	Type of publication
Tai-Seale[86]	2016	CRD participant data not distinguishable from analysis	Participant population
Tamblyn[87]	2005	Abstract only (results in Tamblyn 2015)	Type of publication
Tamblyn[88]	2015	No SDM within intervention	Type of intervention
Tang[89]	2008	No comparator group	Study design
Tapp[90]	2017	No SDM outcomes	Outcome measures
Thee[91]	2021	Protocol paper	Type of publication
Thiam[92]	2007	No SDM within intervention	Type of intervention

For peer review only - http://bmjopen.bmj.com/site/about/guidelines.xhtml

Totten[93]	2018	Abstract only (results not yet published)	Type of publication
Tsao[94]	2021	No SDM within intervention	Type of intervention
Vandemheen[95]	2009	No SDM within intervention	Type of intervention
Vandemheen[95]	2009	Supplement (to be read with Vandemheen 2009)	Type of publication
Wahlberg[96]	2016	CRD participant data not distinguishable from analysis	Participant population
Waller[97]	2012	CRD participant data not distinguishable from analysis	Participant population
Wang[98]	2010	No SDM within intervention	Type of intervention
Weekes[99]	2009	No SDM within intervention	Type of intervention
Williams[100]	2013	CRD participant data not distinguishable from analysis	Participant population
Wilson[101]	2009	Abstract only (results in Wilson 2010)	Type of publication
Wilson[102]	2005	Abstract only (results in Wilson 2010)	Type of publication
Wilson[103]	2005	Abstract only (results in Wilson 2010)	Type of publication
Wood-Baker[104]	2012	No SDM within intervention	Type of intervention
Yoo[105]	2018	No CRD participants	Participant population
Zakrisson[106]	2019	CRD participant data not distinguishable from analysis	Participant population
Zanartu[107]	2017	Letter to editor	Type of publication

only

For peer review only - http://bmjopen.bmj.com/site/about/guidelines.xhtml

Sub-group analysis

Despite the broad inclusion criteria for respiratory conditions, studies only included those with COPD, lung cancer and asthma (Table 2). Participants diagnosed with lung cancer and COPD were older than those diagnosed with asthma. The split between male and female participants were even across studies apart from in one study where nearly all were male[108] and another where three quarters were female[109]. No specific health literacy outcome measures were used across studies. Baseline educational attainment was reported across all but two studies[110,111]. Across other studies, the percentage of participants who completed higher education varied between 17-59.2%.

SDM interventions for COPD participants aimed to facilitate decisions regarding end-of-life care and positive self-management. Those for lung cancer and asthma participants aimed to facilitate treatment decision-making. Four studies reported a single session SDM intervention[108,109,112,113]. Three studies reported multiple sessions of SDM delivered face to face and with follow up telephone calls[110,114,115]. One study did not report an intervention dose and instead stated the intervention was integrated into routine inpatient care[111]. The length of study follow-up varied from immediately post decision-making to 12 months later.

For peer review only

For peer review only - http://bmjopen.bmj.com/site/about/guidelines.xhtml

Table 2: Sub-group analysis of CRD patients from included studies

First Author (Year)	CRD diagnosis	Age (mean (SD))	Gender (% male)	Measure of health literacy (% achievement)	Decision posed	Intervention dose	Length of follow up
Au (2012)	COPD	IG: 69.4(10.0) years	IG: 97.9%	IG: Higher education = 58.3%	IG: End of life care	IG: Single session	IG: 2 weeks
		CG: 69.4(10.0) years	CG: 96.2%	CG: Higher education = 59.2%	CG: End of life care	CG: N/A	CG: 2 weeks
Brundage (2001)	Lung cancer	IG: 69.2(8.1) years	IG: 55%	IG: <High school = 5% High school education = 50% Higher education = 45%	IG: Lung cancer treatments	IG: Single session	IG: Immediately post intervention
Collinsworth (2018)	COPD	IG: 70.0(11.9)	IG: 39.7%	IG: Not disclosed	IG: Self-management plans	IG: Single session and 4 telephone calls	IG: 6 months
		CG: 70.9(12.5)	CG: 43.1%	CG: Not disclosed	CG: Self-management plans	CG: N/A	CG: 6months
Gagne (2017)	Asthma	IG: 46(13.0)	IG: 26.9%	IG: High school education = 57.7% Higher education = 42.3%	IG: Asthma treatments	IG: Single session	IG: 2months
		CG: 41(13.0)	CG: 48%	CG: High school education = 52.0% Higher education = 48.0%	CG: Asthma treatments	CG: N/A	CG: 2months
Granados-Santiago (2019)	COPD	IG: 69.3(9.9)	IG: Not disclosed	IG: Not disclosed	IG: Self-management plans	IG: Integrated into inpatient care	IG: 3 months
		CG: 74.20(9.25)	CG: Not disclosed	CG: Not disclosed	CG: Self-management plans	CG: N/A	CG: 3 months
Myers (2021)	Lung cancer	IG: Not reported	IG: 40%	IG: <High school education = 0%	IG: Lung cancer treatments	IG: Single session	IG: 30 days

Supplementary material V1.0

For peer review only - http://bmjopen.bmj.com/site/about/guidelines.xhtml

				High school education = 40%<br>Higher education = 60%			
Walters (2013)	COPD	IG: 68.2(7.9)	IG: 54.4%	IG:<br><High school education = 68%<br>High school education = 13%<br>Higher education = 20%	IG: Self-management plans	IG: Single session plus multiple (x16) telephone calls	IG: 12 months
		CG: 67.3(7.6)	CG: 51.1%	CG:<br><High school education = 75%<br>High school education = 8%<br>Higher education = 17%	CG: Self-management plans	CG: Single session plus multiple (x12) telephone calls	CG: 12 months
Wilson (2010)	Asthma	IG: 45.7(13.3)	IG: 43.6%	IG:<br><High school education = 2.9%<br>High school education = 55.9%<br>Higher education = 41.1%	IG: Asthma treatments	IG: Multiple sessions (x2) plus multiple telephone calls (x3)	IG: 12 months
		IG2: 46.9(12.1)	IG2: 44.1%	IG2:<br><High school education = 0.98%<br>High school education = 64.7%<br>Higher education = 33.8%	IG2: Asthma treatments	IG2: Multiple sessions (x2) plus multiple telephone calls (x3)	IG2: 9 months
		CG: 45.1(12.4)	CG: 42.6%	CG:<br><High school education= 2.9%<br>High school education = 56.9%<br>Higher education = 40.2%	CG: Asthma treatments	CG: Multiple sessions (x2) plus multiple telephone calls (x3)	CG: 9 months

IG = Intervention group; CG = Control group; IG2 = Intervention group 2 (Clinician Decision-Making)

For peer review only - http://bmjopen.bmj.com/site/about/guidelines.xhtml

For peer review only

References

E1. Aaron SD. Evaluation of a Decision Aid for Adult Cystic Fibrosis Patients Considering Bilateral Lung Transplantation. *Clin Regist Clin trials*. 2006.

2. Aboumatar H, Naqibuddin M, Chung S, et al. Better Respiratory Education and Treatment Help Empower (BREATHE) study: Methodology and baseline characteristics of a randomized controlled trial testing a transitional care program to improve patient-centered care delivery among chronic obstructive pulm. *Contemp Clin Trials*. 2017;62(June):159-167. doi:10.1016/j.cct.2017.08.018

3. Aboumatar H, Naqibuddin M, Chung S, et al. Effect of a Hospital-Initiated Program Combining Transitional Care and Long-term Self-management Support on Outcomes of Patients Hospitalized with Chronic Obstructive Pulmonary Disease: A Randomized Clinical Trial. *JAMA - J Am Med Assoc*. 2019;322(14):1371-1380. doi:10.1001/jama.2019.11982

4. Braun U. A Culturally Sensitive Values-Guided Aid for End of Life Decision-Making. *Clin Regist Clin Trials*. 2005. doi:10.1002/CENTRAL/CN-01510546

5. Brown RF, Bylund CL, Li Y, Edgerson S, Butow P. Testing the utility of a cancer clinical trial specific Question Prompt List (QPL-CT) during oncology consultations. *Patient Educ Couns*. 2012;88(2):311-317. doi:10.1016/j.pec.2012.02.009

6. Brown RF, Butow PN, Sharrock MA, et al. Education and role modelling for clinical decisions with female cancer patients. *Heal Expect*. 2004;7(4):303-316. doi:10.1111/j.1369-7625.2004.00294.x

7. Brundage MD, Davidson JR, Mackillop WJ, Feldman-Stewart DEB, Groome P. Using a treatment-tradeoff method to elicit preferences for the treatment of locally advanced non-small-cell lung cancer. *Med Decis Mak*. 1998;18(3):256-267. doi:10.1177/0272989X9801800302

8. Butow P, Brown R, Aldridge J, et al. Can consultation skills training change doctors' behaviour to increase involvement of patients in making decisions about standard treatment and clinical trials: A randomized controlled trial. *Heal Expect*. 2015;18(6):2570-2583. doi:10.1111/hex.12229

9. Carlucci A, Vitacca M, Malovini A, et al. End-of-Life Discussion, Patient Understanding and Determinants of Preferences in Very Severe COPD Patients: A Multicentric Study. *COPD J Chronic Obstr Pulm Dis*. 2016;13(5):632-638. doi:10.3109/15412555.2016.1154034

For peer review only

For peer review only - http://bmjopen.bmj.com/site/about/guidelines.xhtml

10. Cook KA, Modena BD, Simon RA. Improvement in Asthma Control Using a Minimally Burdensome and Proactive Smartphone Application. *J Allergy Clin Immunol Pract*. 2016;4(4):730-737.e1. doi:10.1016/j.jaip.2016.03.005

11. Coultas D, Frederick J, Barnett B, Singh G, Wludyka P. A randomized trial of two types of nurse-assisted home care for patients with COPD. *Chest*. 2005;128(4):2017-2024. doi:10.1378/chest.128.4.2017

12. Cvietusa PJ, Wagner NM, Shoup JA, et al. Digital Communication Technology: Does Offering a Choice of Modality Improve Medication Adherence and Outcomes in a Persistent Asthma Population? *Perm J*. 2020;25:1. doi:10.7812/TPP/20.189

13. Daly BJ, Douglas SL, Gunzler D, Lipson AR. Clinical trial of a supportive care team for patients with advanced cancer. *J Pain Symptom Manage*. 2013;46(6):775-784. doi:10.1016/j.jpainsymman.2012.12.008

14. Detmar SB, Muller MJ, Schornagel JH, Wever LDV, Aaronson NK. Health-related quality-of-life assessments and patient-physician communication: A randomized controlled trial. *J Am Med Assoc*. 2002;288(23):3027-3034. doi:10.1001/jama.288.23.3027

15. Dyar S, Lesperance M, Shannon R, Sloan J, Colon-Otero G. A nurse practitioner directed intervention improves the quality of life of patients with metastatic cancer: Results of a randomized pilot study. *J Palliat Med*. 2012;15(8):890-895. doi:10.1089/jpm.2012.0014

16. Early F, Everden AJT, OBrien CM, Fagan PL, Fuld JP. Patient agenda setting in respiratory outpatients. *Chron Respir Dis*. 2015;12(4):347-356. doi:10.1177/1479972315598696

17. Fiks AG, Mayne SL, Karavite DJ, et al. Parent-reported outcomes of a shared decision-making portal in asthma: A practice-based RCT. *Pediatrics*. 2015;135(4):e965-e973. doi:10.1542/peds.2014-3167

18. Fors A, Blanck E, Ali L, et al. Effects of a person-centred telephone-support in patients with chronic obstructive pulmonary disease and/or chronic heart failure – A randomized controlled trial. *PLoS One*. 2018;13(8). doi:10.1371/journal.pone.0203031

19. Foster JM, Smith L, Usherwood T, Sawyer SM, Reddel HK. General practitioner-delivered adherence counseling in asthma: Feasibility and usefulness of skills, training and support tools. *J Asthma*. 2016;53(3):311-320. doi:10.3109/02770903.2015.1091473

20. Foster JM, Usherwood T, Smith L, et al. Inhaler reminders improve adherence with controller treatment in primary care patients with asthma. *J Allergy Clin Immunol*. 2014;134(6):1260-

For peer review only - http://bmjopen.bmj.com/site/about/guidelines.xhtml

1268.e3. doi:10.1016/j.jaci.2014.05.041

21. Fraenkel L, Peters E, Tyra S, Oelberg D. Shared Medical Decision Making in Lung Cancer Screening: Experienced versus Descriptive Risk Formats. *Med Decis Mak*. 2016;36(4):518-525. doi:10.1177/0272989X15611083

22. Gagné M, Légaré F, Moisan J, Boulet L. Adding A Decision Aid to Asthma Education: Impact on Decisional Conflict and Appropriate Medication Usage. *Value Heal*. 2016;19(7):A557. doi:10.1016/j.jval.2016.09.1216

23. George M. BREATHE asthma intervention trial. *Clin Regist Clin Trials*. 2017. doi:10.1002/CENTRAL/CN-01380211

24. George M, Bruzzese JM, S. Sommers M, et al. Group-randomized trial of tailored brief shared decision-making to improve asthma control in urban black adults. *J Adv Nurs*. 2020;(September):1-17. doi:10.1111/jan.14646

25. Gupta S, Price C, Agarwal G, et al. *The Electronic Asthma Management System (EAMS) Improves Primary Care Asthma Management*. Vol 53.; 2019. doi:10.1183/13993003.02241-2018

26. Hagan L, Valois P, Patenaude H, Boutin H, Boulet LP, Lafrenière F. Asthma counselling targeted to removal of domestic animals. *Can Respir J*. 2008;15(1):33-38. doi:10.1155/2008/247027

27. Halley MC, Rendle KAS, Gillespie KA, Stanley KM, Frosch DL. An exploratory mixed-methods crossover study comparing DVD- vs. Web-based patient decision support in three conditions: The importance of patient perspectives. *Heal Expect*. 2015;18(6):2880-2891. doi:10.1111/hex.12273

28. Hilberink SR, Jacobs JE, Breteler MHM, de Vries H, Grol RPTM. General practice counseling for patients with chronic obstructive pulmonary disease to quit smoking: Impact after 1year of two complex interventions. *Patient Educ Couns*. 2011;83(1):120-124. doi:10.1016/j.pec.2010.04.009

29. Hoerger M, Perry LM, Gramling R, Epstein RM, Duberstein PR. Does educating patients about the early palliative care study increase preferences for outpatient palliative cancer care? Findings from project EMPOWER. *Heal Psychol*. 2017;36(6):538-548. doi:10.1037/hea0000489

30. Hollen PJ, Gralla RJ, Jones RA, et al. A theory-based decision aid for patients with cancer:

For peer review only - http://bmjopen.bmj.com/site/about/guidelines.xhtml
For peer review only

Results of feasibility and acceptability testing of DecisionKEYS for cancer. *Support Care Cancer*. 2013;21(3):889-899. doi:10.1007/s00520-012-1603-8

31. Hoskins G, Williams B, Abhyankar P, et al. Achieving Good Outcomes for Asthma Living (GOAL): Mixed methods feasibility and pilot cluster randomised controlled trial of a practical intervention for eliciting, setting and achieving goals for adults with asthma. *Trials*. 2016;17(1):1-17. doi:10.1186/s13063-016-1684-7

32. Houben CHM, Spruit MA, Luyten H, et al. Cluster-randomised trial of a nurse-led advance care planning session in patients with COPD and their loved ones. *Thorax*. 2019;74(4):328-336. doi:10.1136/thoraxjnl-2018-211943

33. Hyatt A, Lipson-Smith R, Gough K, et al. Culturally and linguistically diverse oncology patients' perspectives of consultation audio-recordings and question prompt lists. *Psychooncology*. 2018;27(9):2180-2188. doi:10.1002/pon.4789

34. Izumi SS, Basin B, Presley M, et al. Feasibility and Acceptability of Nurse-Led Primary Palliative Care for Older Adults with Chronic Conditions: A Pilot Study. *J Palliat Med*. 2018;21(8):1114-1121. doi:10.1089/jpm.2017.0666

35. Jonsdottir H, Amundadottir OR, Gudmundsson G, et al. Effectiveness of a partnership-based self-management programme for patients with mild and moderate chronic obstructive pulmonary disease: A pragmatic randomized controlled trial. *J Adv Nurs*. 2015;71(11):2634-2649. doi:10.1111/jan.12728

36. Kass NE, Sugarman J, Medley AM, et al. An intervention to improve cancer patients' understanding of early-phase clinical trials. *IRB Ethics Hum Res*. 2009;31(3):1-8.

37. Kennedy A, Bower P, Reeves D, et al. Implementation of self management support for long term conditions in routine primary care settings: Cluster randomised controlled trial. *BMJ*. 2013;346(7913):1-11. doi:10.1136/bmj.f2882

38. Koerner M, Wirtz M, Michaelis M, et al. A multicentre cluster-randomized controlled study to evaluate a train-the-trainer programme for implementing internal and external participation in medical rehabilitation. *Clin Rehabil*. 2014;28(1):20-35. doi:10.1177/0269215513494874

39. Krug K, Bossert J, Deis N, et al. Effects of an Interprofessional Communication Approach on Support Needs, Quality of Life, and Mood of Patients with Advanced Lung Cancer: A Randomized Trial. *Oncologist*. 2021;26(8):e1445-e1459. doi:10.1002/ONCO.13790

40. Lainscak M, Kadivec S, Kosnik M, et al. Discharge coordinator intervention prevents

For peer review only - http://bmjopen.bmj.com/site/about/guidelines.xhtml

hospitalizations in patients with COPD: A randomized controlled trial. *J Am Med Dir Assoc*. 2013;14(6):450.e1-450.e6. doi:10.1016/j.jamda.2013.03.003

41. Latimer AE, Williams-Piehota P, Cox A, Katulak NA, Salovey P, Mowad L. Encouraging cancer patients to talk to their physicians about clinical trials: Considering patients' information needs. *J Appl Biobehav Res*. 2007;12(3-4):178-195. doi:10.1111/j.1751-9861.2008.00020.x

42. Lavoie KL, Moullec G, Lemiere C, et al. Efficacy of brief motivational interviewing to improve adherence to inhaled corticosteroids among adult asthmatics: Results from a randomized controlled pilot feasibility trial. *Patient Prefer Adherence*. 2014;8:1555-1569. doi:10.2147/PPA.S66966

43. Leung JM, Udris EM, Uman J, Au DH. The effect of end-of-life discussions on perceived quality of care and health status among patients with COPD. *Chest*. 2012;142(1):128-133. doi:10.1378/chest.11-2222

44. Lim RK, Semitala FC, Atuhumuza E, et al. Patient choice improves self-efficacy and intention to complete tuberculosis preventive therapy in a routine HIV program setting in Uganda. *PLoS One*. 2021;16(2):e0246113. doi:10.1371/JOURNAL.PONE.0246113

45. Llanos-Paez C, Ambery C, Yang S, et al. Improved Decision-Making Confidence Using Item-Based Pharmacometric Model: Illustration with a Phase II Placebo-Controlled Trial. *AAPS J*. 2021;23(4). doi:10.1208/s12248-021-00600-1

46. Lopez-Lopez L, Valenza MC, Rodriguez-Torres J, Torres-Sanchez I, Granados-Santiago M, Valenza-Demet G. Results on health-related quality of life and functionality of a patient-centered self-management program in hospitalized COPD: a randomized control trial. *Disabil Rehabil*. 2020;42(25):3687-3695. doi:10.1080/09638288.2019.1609099

47. Ludden T, Shade L, Reeves K, et al. Asthma dissemination around patient-centered treatments in North Carolina (ADAPT-NC): a cluster randomized control trial evaluating dissemination of an evidence-based shared decision-making intervention for asthma management. *J Asthma*. September 2018:1-12. doi:10.1080/02770903.2018.1514630

48. Makimoto G, Hotta K, Oze I, et al. Patients' preferences and perceptions of lung cancer treatment decision making: results from Okayama lung cancer study group trial 1406. *Acta Oncol (Madr)*. 2020;59(3):324-328. doi:10.1080/0284186X.2019.1679880

49. Markman M, Petersen J, Montgomery R. An examination of characteristics of lung and colon cancer patients participating in a web-based decision support program: Internet-based

For peer review only - http://bmjopen.bmj.com/site/about/guidelines.xhtml

decision support programs. *Oncology*. 2005;69(4):311-316. doi:10.1159/000089763

50. Markun S, Rosemann T, Dalla-Lana K, Steurer-Stey C. Care in Chronic Obstructive Lung Disease (CAROL): A randomised trial in general practice. *Eur Respir J*. 2018;51(5). doi:10.1183/13993003.01873-2017

51. Mayer DK, Deal AM, Crane JM, et al. Using survivorship care plans to enhance communication and cancer care coordination: Results of a pilot study. *Oncol Nurs Forum*. 2016;43(5):636-645. doi:10.1188/16.ONF.636-645

52. Meline J, Prigge JM, Dye D, et al. Adapting the design of a Web-based decision support clinical trial during the COVID-19 pandemic. *Trials*. 2021;22(1):1-6. doi:10.1186/S13063-021-05700-Z/PEER-REVIEW

53. Melzer AC, Clothier BA, Japuntich SJ, et al. Comparative effectiveness of proactive tobacco treatment among smokers with and without chronic lower respiratory disease. *Ann Am Thorac Soc*. 2018;15(3):341-347. doi:10.1513/AnnalsATS.201707-582OC

54. Menon S, Mccullough LB, Beyth RJ, Ford ME, Espadas D, Braun UK. Use of a values inventory as a discussion aid about end-of-life care: A pilot randomized controlled trial. *Palliat Support Care*. 2016;14(4):330-340. doi:10.1017/S1478951515001091

55. Meropol NJ, Egleston BL, Buzaglo JS, et al. A Web-based communication aid for patients with cancer: The CONNECT Study. *Cancer*. 2013;119(7):1437-1445. doi:10.1002/cncr.27874

56. Moullec G, Lavoie KL, Rabhi K, Julien M, Favreau H, Labrecque M. Effect of an integrated care programme on re-hospitalization of patients with chronic obstructive pulmonary disease. *Respirology*. 2012;17(4):707-714. doi:10.1111/j.1440-1843.2012.02168.x

57. Murray MA, Stacey D, Wilson KG, O'Connor AM. Skills training to support patients considering place of end-of-life care: A randomized control trial. *J Palliat Care*. 2010;26(2):112-121. doi:10.1177/082585971002600207

58. Nedjat-Haiem FR, Carrion I V., Gonzalez K, et al. Implementing an Advance Care Planning Intervention in Community Settings with Older Latinos: A Feasibility Study. *J Palliat Med*. 2017;20(9):984-993. doi:10.1089/jpm.2016.0504

59. Nugent SM, Golden SE, Thomas CR, et al. Patient-clinician communication among patients with stage I lung cancer. *Support Care Cancer*. 2018;26(5):1625-1633. doi:10.1007/s00520-017-3992-1

For peer review only - http://bmjopen.bmj.com/site/about/guidelines.xhtml

For peer review only

60. Patel MR, Smith A, Leo H, Hao W, Zheng K. Improving Patient–Provider Communication and Therapeutic Practice Through Better Integration of Electronic Health Records in the Exam Room: A Pilot Study. *Heal Educ Behav*. 2019;46(3):484-493. doi:10.1177/1090198118796879

61. Rabow MW, Dibble SL, Pantilat SZ, McPhee SJ. The Comprehensive Care Team. *Arch Intern Med*. 2004;164(1):83. doi:10.1001/archinte.164.1.83

62. Rea H, McAuley S, Stewart A, Lamont C, Roseman P, Didsbury P. A chronic disease management programme can reduce days in hospital for patients with chronic obstructive pulmonary disease. *Intern Med J*. 2004;34(11):608-614. doi:10.1111/j.1445-5994.2004.00672.x

63. Reddel HK, Correll J, Foster J, Karlsson N, Martin M, Keen C. Patient Decision-Making Around Use of Reliever Inhalers in Mild Asthma. In: *American Thoracic Society International Conference Meetings Abstracts*. American Thoracic Society; 2019:A5935-A5935. doi:10.1164/ajrccm-conference.2019.199.1_meetingabstracts.a5935

64. Reinke LF, Feemster LC, McDowell J, et al. The long term impact of an end-of-life communication intervention among veterans with COPD. *Hear Lung J Acute Crit Care*. 2017;46(1):30-34. doi:10.1016/j.hrtlng.2016.10.003

65. Rhee H, Hollen PJ, Belyea MJ, Sutherland MA. Decision-Making Program for Rural Adolescents With Asthma: A Pilot Study. *J Pediatr Nurs*. 2008;23(6):439-450. doi:10.1016/j.pedn.2008.01.079

66. Roelands M, Van Oost P, Stevens V, Depoorter AM, Buysse A. Clinical practice guidelines to improve shared decision-making about assistive device use in home care: A pilot intervention study. *Patient Educ Couns*. 2004;55(2):252-264. doi:10.1016/j.pec.2003.10.002

67. Rosemann T. The Improving Care in Chronic Obstructive Lung Disease Study A Cluster Randomized Trial. *https://clinicaltrials.gov/show/NCT01921556*. 2013. doi:10.1002/CENTRAL/CN-02032168

68. Ruparel M, Quaife SL, Ghimire B, et al. Impact of a lung cancer screening information film on informed decision-making: A randomized trial. *Ann Am Thorac Soc*. 2019;16(6):744-751. doi:10.1513/AnnalsATS.201811-841OC

69. Rutten-vanMolken M, Goossens L, Boland M, et al. The Assessment of Burden of COPD (ABC) tool: a shared decision-making instrument that is predictive of healthcare costs. *Int J Integr Care*. 2017;17(5):320. doi:10.5334/ijic.3637

For peer review only - http://bmjopen.bmj.com/site/about/guidelines.xhtml

70. Sands M. A Randomised Control Trial for Advance Care Planning and Symptom Management for patients identified in the emergency department and followed up at home. *Clin Regist Clin Trials*. 2014. doi:10.1002/CENTRAL/CN-01881924

71. Schofield P, Ugalde A, Gough K, et al. A tailored, supportive care intervention using systematic assessment designed for people with inoperable lung cancer: A randomised controlled trial. *Psychooncology*. 2013;22(11):2445-2453. doi:10.1002/pon.3306

72. Schubart JR, Green MJ, Van Scoy LJ, et al. Advanced Cancer and End-of-Life Preferences: Curative Intent Surgery Versus Noncurative Intent Treatment. *J Palliat Med*. 2015;18(12):1015-1018. doi:10.1089/jpm.2015.0021

73. Schumacher KL, Koresawa S, West C, et al. Putting cancer pain management regimens into practice at home. *J Pain Symptom Manage*. 2002;23(5):369-382. doi:10.1016/S0885-3924(02)00385-8

74. Schwarze ML, Buffington A, Tucholka JL, et al. Effectiveness of a Question Prompt List Intervention for Older Patients Considering Major Surgery: A Multisite Randomized Clinical Trial. *JAMA Surg*. 2020;155(1):6-13. doi:10.1001/jamasurg.2019.3778

75. Ludden T, Shade L, Reeves K, et al. Asthma dissemination around patient-centered treatments in North Carolina (ADAPT-NC): a cluster randomized control trial evaluating dissemination of an evidence-based shared decision-making intervention for asthma management. *J Asthma*. 2019;56(10):1087-1098. doi:10.1080/02770903.2018.1514630

76. Shirai Y, Fujimori M, Ogawa A, et al. Patients' perception of the usefulness of a question prompt sheet for advanced cancer patients when deciding the initial treatment: A randomized, controlled trial. *Psychooncology*. 2012;21(7):706-713. doi:10.1002/pon.1955

77. Sinclair C, Auret KA, Evans SF, et al. Advance care planning uptake among patients with severe lung disease: A randomised patient preference trial of a nurse-led, facilitated advance care planning intervention. *BMJ Open*. 2017;7(2):1-11. doi:10.1136/bmjopen-2016-013415

78. Skorstengaard MH, Brogaard T, Bonde Jensen A, et al. Advance care planning for patients and their relatives. *Int J Palliat Nurs*. 2019;25(3):112-127. doi:10.12968/ijpn.2019.25.3.112

79. Slok A, Kotz D, van Breukelen G, et al. The assessment of burden of COPD tool improves health related quality of life. In: *European Respiratory Journal*. Vol 48. European Respiratory Society (ERS); 2016:OA1997. doi:10.1183/13993003.congress-2016.oa1997

80. Slok AHM, Kotz D, Van Breukelen G, et al. Effectiveness of the Assessment of Burden of COPD

For peer review only - http://bmjopen.bmj.com/site/about/guidelines.xhtml

(ABC) tool on health-related quality of life in patients with COPD: A cluster randomised controlled trial in primary and hospital care. *BMJ Open*. 2016;6(7). doi:10.1136/bmjopen-2016-011519

81. Smidth M, Olesen F, Fenger-Grøn M, Vedsted P. Patient-experienced effect of an active implementation of a disease management programme for COPD - A randomised trial. *BMC Fam Pract*. 2013;14(1):1. doi:10.1186/1471-2296-14-147

82. Steinhauser KE, Alexander SC, Byock IR, George LK, Olsen MK, Tulsky JA. Do preparation and life completion discussions improve functioning and quality of life in seriously ill patients? Pilot randomized control trial. *J Palliat Med*. 2008;11(9):1234-1240. doi:10.1089/jpm.2008.0078

83. Steurer C, Markun S, Rosemann T, Dalla Lana K. Improving Care in Chronic Obstructive Lung Disease (CAROL): a cluster randomized trial in primary care. In: European Respiratory Society (ERS); 2017:OA2915. doi:10.1183/1393003.congress-2017.oa2915

84. Steurer-Stey C, Storch M, Benz S, et al. Motivational training improves self-efficacy but not short-term adherence with asthma self-management: a randomized controlled trial. *Prim Health Care Res Dev*. 2015;16(1):32-41. doi:10.1017/S1463423613000480

85. Sullivan DR, Slatore CG, Stone K, et al. Associations Between Treatment and Team Composition with Decisional Regret Among Patients with Early Stage Lung Cancer. May 2020:A4726-A4726. doi:10.1164/AJRCCM-CONFERENCE.2020.201.1_MEETINGABSTRACTS.A4726

86. Tai-Seale M, Elwyn G, Wilson CJ, et al. Enhancing shared decision making through carefully designed interventions that target patient and provider behavior. *Health Aff*. 2016;35(4):605-612. doi:10.1377/hlthaff.2015.1398

87. Tamblyn R. Computer-Based Decision Support in Managing Asthma in Primary Care. *Clin Regist Clin Trials*. 2005. doi:10.1002/CENTRAL/CN-02024751

88. Tamblyn R, Ernst P, Winslade N, et al. Evaluating the impact of an integrated computer-based decision support with person-centered analytics for the management of asthma in primary care: A randomized controlled trial. *J Am Med Informatics Assoc*. 2015;22(4):773-783. doi:10.1093/jamia/ocu009

89. Tang JI, Shakespeare TP, Lu JJ, et al. Patients' preference for radiotherapy fractionation schedule in the palliation of symptomatic unresectable lung cancer. *J Med Imaging Radiat*

For peer review only - http://bmjopen.bmj.com/site/about/guidelines.xhtml

Oncol. 2008;52(5):497-502. doi:10.1111/j.1440-1673.2008.02002.x

90. Tapp H, Shade L, Mahabaleshwarkar R, Taylor YJ, Ludden T, Dulin MF. Results from a pragmatic prospective cohort study: Shared decision making improves outcomes for children with asthma. *J Asthma*. 2017;54(4):392-402. doi:10.1080/02770903.2016.1227333

91. Thee S, Stahl M, Fischer R, et al. A multi-centre, randomized, controlled trial on coaching and telemonitoring in patients with cystic fibrosis: conneCT CF. *BMC Pulm Med*. 2021;21(1):1-11. doi:10.1186/S12890-021-01500-Y/TABLES/1

92. Thiam S, LeFevre AM, Hane F, et al. Effectiveness of a strategy to improve adherence to tuberculosis treatment in a resource-poor setting: A cluster randomized controlled trial. *J Am Med Assoc*. 2007;297(4):380-386. doi:10.1001/jama.297.4.380

93. Totten A. Team-based Versus Primary Care Clinician-led Advance Care Planning in Practice-based Research Networks. *Clin Regist Clin Trials*. 2018. doi:10.1002/CENTRAL/CN-01660857

94. Tsao S, Willard-Grace R, Wolf J, et al. Implementation and Impact of the Pulmonary Specialist Health Coach Consultation Model to Improve Care for Patients with COPD. *Jt Comm J Qual patient Saf*. 2021;47(11):739-747. doi:10.1016/J.JCJQ.2021.08.003

95. Vandemheen KL, O'Connor A, Bell SC, et al. Randomized trial of a decision aid for patients with cystic fibrosis considering lung transplantation. *Am J Respir Crit Care Med*. 2009;180(8):761-768. doi:10.1164/rccm.200903-0421OC

96. Wahlberg H, Braaten T, Broderstad AR. Impact of referral templates on patient experience of the referral and care process: A cluster randomised trial. *BMJ Open*. 2016;6(10):1-8. doi:10.1136/bmjopen-2016-011651

97. Waller A, Girgis A, Johnson C, et al. Improving outcomes for people with progressive cancer: Interrupted time series trial of a needs assessment intervention. *J Pain Symptom Manage*. 2012;43(3):569-581. doi:10.1016/j.jpainsymman.2011.04.020

98. Wang KY, Chian CF, Lai HR, Tarn YH, Wu CP. Clinical pharmacist counseling improves outcomes for Taiwanese asthma patients. *Pharm World Sci*. 2010;32(6):721-729. doi:10.1007/s11096-010-9427-4

99. Weekes CE, Emery PW, Elia M. Dietary counselling and food fortification in stable copd: A randomised trial. *Thorax*. 2009;64(4):326-331. doi:10.1136/thx.2008.097352

100. Williams PD, Graham KM, Storlie DL, et al. Therapy-related symptom checklist use during

For peer review only

For peer review only - http://bmjopen.bmj.com/site/about/guidelines.xhtml

treatments at a cancer center. *Cancer Nurs*. 2013;36(3):245-254. doi:10.1097/NCC.0b013e3182595406

101. Wilson SR, Strub P, Knowles SB, Buist AS, Huang Q, Nguyen M. Ethnicity, Income, and Education as Potential Modifiers of the Effects of Shared Treatment Decision-Making (SDM) Between Asthma Care Manager and Patient on Asthma Controller Medication Adherence: The Better Outcomes of Asthma Treatment (BOAT) Trial. *J Allergy Clin Immunol*. 2009;123(2):S72-S72. doi:10.1016/j.jaci.2008.12.247

102. Wilson S. Does Shared Decision-Making Improve Asthma Outcomes? *Clin Regist Clin Trials*. 2005. doi:10.1002/CENTRAL/CN-02031001

103. Wilson S. Does shared decision-making improve adherence in asthma. *Clin Regist Clin Trials*. 2005. doi:10.1002/CENTRAL/CN-01427587

104. Wood-baker R, Reid D, Robinson A. Wood-Baker et al: Clinical trial of community nurse mentoring to improve self-management in patients with chronic obstructive pulmonary disease. International Journal of COPD 2012, 7:407-13. 2012:407-413. http://www.pubmedcentral.nih.gov/articlerender.fcgi?artid=3402057&tool=pmcentrez&rendertype=abstract.

105. Yoo SH, Yun YH, Kim KN, et al. The impact of caregiver's role preference on decisional conflicts and psychiatric distresses in decision making to help caregiver's disclosure of terminal disease status. *Qual Life Res*. 2018;27(6):1571-1581. doi:10.1007/s11136-018-1814-7

106. Zakrisson AB, Arne M, Hasselgren M, Lisspers K, Ställberg B, Theander K. A complex intervention of self-management for patients with COPD or CHF in primary care improved performance and satisfaction with regard to own selected activities; A longitudinal follow-up. *J Adv Nurs*. 2019;75(1):175-186. doi:10.1111/jan.13899

107. Zanartu CP, Camacho FJ, Nevadunski NS, et al. Speaking the same language: a feasibility trial for a novel visual communication tool for oncologist-patient treatment discussions. *Psychooncology*. 2017;26(7):1050-1052. doi:10.1002/pon.4246

108. Au DH, Udris EM, Engelberg RA, et al. A randomized trial to improve communication about end-of-life care among patients with COPD. *Chest*. 2012;141(3):726-735. doi:10.1378/chest.11-0362

109. Gagné ME, Légaré F, Moisan J, Boulet L-P. Impact of Adding a Decision Aid to Patient

For peer review only - http://bmjopen.bmj.com/site/about/guidelines.xhtml

Education in Adults with Asthma: A Randomized Clinical Trial. Leroyer C, ed. *PLoS One*. 2017;12(1):e0170055. doi:10.1371/journal.pone.0170055

110. Collinsworth AW, Brown RM, Sjames C, stanford RH, Alemayehu D, Priest EL. The impact of patient education and shared decision making on hospital readmissions for COPD. *Int J COPD*. 2018;13:1325-1332. doi:10.2147/COPD.S154414

111. Granados-Santiago M, Valenza MC, López-López L, Prados-Román E, Rodríguez-Torres J, Cabrera-Martos I. Shared decision-making and patient engagement program during acute exacerbation of COPD hospitalization: A randomized control trial. *Patient Educ Couns*. 2019;103(4):702-708. doi:10.1016/j.pec.2019.12.004

112. Brundage MD, Feldman-Stewart D, Cosby R, et al. Phase I study of a decision aid for patients with locally advanced non-small-cell lung cancer. *J Clin Oncol*. 2001;19(5):1326-1335. doi:10.1200/JCO.2001.19.5.1326

113. Myers RE, Advani SM, Myers P, et al. Engaging Patients with Late-Stage Non-Small Cell Lung Cancer in Shared Decision Making about Treatment. *J Pers Med*. 2021;11(10). doi:10.3390/JPM11100998

114. Walters J, Cameron-Tucker H, Wills K, et al. Effects of telephone health mentoring in community-recruited chronic obstructive pulmonary disease on self-management capacity, quality of life and psychological morbidity: A randomised controlled trial. *BMJ Open*. 2013;3(9). doi:10.1136/bmjopen-2013-003097

115. Wilson SR, Strub P, Buist AS, et al. Shared treatment decision making improves adherence and outcomes in poorly controlled asthma. *Am J Respir Crit Care Med*. 2010;181(6):566-577. doi:10.1164/rccm.200906-0907OC

For peer review only

For peer review only - http://bmjopen.bmj.com/site/about/guidelines.xhtml

PRISMA 2020 Checklist

Section and Topic	Item #	Checklist item	Location where item is reported
**TITLE**			
Title	1	Identify the report as a systematic review.	Title, pg1
**ABSTRACT**			
Abstract	2	See the PRISMA 2020 for Abstracts checklist.	Abstract, pg3-4
**INTRODUCTION**			
Rationale	3	Describe the rationale for the review in the context of existing knowledge.	Introduction, pg6-8
Objectives	4	Provide an explicit statement of the objective(s) or question(s) the review addresses.	Introduction, pg8
**METHODS**			
Eligibility criteria	5	Specify the inclusion and exclusion criteria for the review and how studies were grouped for the syntheses.	Methods, pg8-9
Information sources	6	Specify all databases, registers, websites, organisations, reference lists and other sources searched or consulted to identify studies. Specify the date when each source was last searched or consulted.	Methods, pg9
Search strategy	7	Present the full search strategies for all databases, registers and websites, including any filters and limits used.	Methods, pg10 & Supplementary Material
Selection process	8	Specify the methods used to decide whether a study met the inclusion criteria of the review, including how many reviewers screened each record and each report retrieved, whether they worked independently, and if applicable, details of automation tools used in the process.	Methods, pg10
Data collection process	9	Specify the methods used to collect data from reports, including how many reviewers collected data from each report, whether they worked independently, any processes for obtaining or confirming data from study investigators, and if applicable, details of automation tools used in the process.	Methods, pg10
Data items	10a	List and define all outcomes for which data were sought. Specify whether all results that were compatible with each outcome domain in each study were sought (e.g. for all measures, time points, analyses), and if not, the methods used to decide which results to collect.	Supplementary Material
	10b	List and define all other variables for which data were sought (e.g. participant and intervention characteristics, funding sources). Describe any assumptions made about any missing or unclear information.	Supplementary Material
Study risk of bias assessment	11	Specify the methods used to assess risk of bias in the included studies, including details of the tool(s) used, how many reviewers assessed each study and whether they worked independently, and if applicable, details of automation tools used in the process.	Methods, pg10-11
Effect measures	12	Specify for each outcome the effect measure(s) (e.g. risk ratio, mean difference) used in the synthesis or presentation of results.	Methods, pg11
Synthesis methods	13a	Describe the processes used to decide which studies were eligible for each synthesis (e.g. tabulating the study intervention characteristics and comparing against the planned groups for each synthesis (item #5)).	Methods, pg11
	13b	Describe any methods required to prepare the data for presentation or synthesis, such as handling of missing summary statistics, or data conversions.	Methods, pg11
	13c	Describe any methods used to tabulate or visually display results of individual studies and syntheses.	Methods, pg11
	13d	Describe any methods used to synthesize results and provide a rationale for the choice(s). If meta-analysis was performed, describe the model(s), method(s) to identify the presence and extent of statistical heterogeneity, and software package(s) used.	Methods, pg11

For peer review only

PRISMA 2020 Checklist

Section and Topic	Item #	Checklist item	Location where item is reported
	13e	Describe any methods used to explore possible causes of heterogeneity among study results (e.g. subgroup analysis, meta-regression).	Methods, pg11
	13f	Describe any sensitivity analyses conducted to assess robustness of the synthesized results.	N/A
Reporting bias assessment	14	Describe any methods used to assess risk of bias due to missing results in a synthesis (arising from reporting biases).	Methods, pg10-11
Certainty assessment	15	Describe any methods used to assess certainty (or confidence) in the body of evidence for an outcome.	Methods, pg11
**RESULTS**			
Study selection	16a	Describe the results of the search and selection process, from the number of records identified in the search to the number of studies included in the review, ideally using a flow diagram.	Results, pg11-12, Figure II
	16b	Cite studies that might appear to meet the inclusion criteria, but which were excluded, and explain why they were excluded.	Results, pg11-12, Figure II, Supplementary Material
Study characteristics	17	Cite each included study and present its characteristics.	Results, pg12, Tables I and II
Risk of bias in studies	18	Present assessments of risk of bias for each included study.	Results, pg12, Figure IV and V
Results of individual studies	19	For all outcomes, present, for each study: (a) summary statistics for each group (where appropriate) and (b) an effect estimate and its precision (e.g. confidence/credible interval), ideally using structured tables or plots.	Table I
Results of syntheses	20a	For each synthesis, briefly summarise the characteristics and risk of bias among contributing studies.	Results, pg12, Table I, Figure IV and V
	20b	Present results of all statistical syntheses conducted. If meta-analysis was done, present for each the summary estimate and its precision (e.g. confidence/credible interval) and measures of statistical heterogeneity. If comparing groups, describe the direction of the effect.	Results, pg13-16, Table I
	20c	Present results of all investigations of possible causes of heterogeneity among study results.	Supplementary Material
	20d	Present results of all sensitivity analyses conducted to assess the robustness of the synthesized results.	N/A
Reporting biases	21	Present assessments of risk of bias due to missing results (arising from reporting biases) for each synthesis assessed.	Results, pg12, Figure IV and V
Certainty of evidence	22	Present assessments of certainty (or confidence) in the body of evidence for each outcome assessed.	Table I
**DISCUSSION**			
Discussion	23a	Provide a general interpretation of the results in the context of other evidence.	Discussion, pg16-18
	23b	Discuss any limitations of the evidence included in the review.	Discussion, pg18

For peer review only - http://bmjopen.bmj.com/site/about/guidelines.xhtml

PRISMA 2020 Checklist

Section and Topic	Item #	Checklist item	Location where item is reported
	23c	Discuss any limitations of the review processes used.	Discussion, pg18-19
	23d	Discuss implications of the results for practice, policy, and future research.	Discussion, pg19
**OTHER INFORMATION**			
Registration and protocol	24a	Provide registration information for the review, including register name and registration number, or state that the review was not registered.	Other information, pg20
	24b	Indicate where the review protocol can be accessed, or state that a protocol was not prepared.	Other information, pg20
	24c	Describe and explain any amendments to information provided at registration or in the protocol.	N/A
Support	25	Describe sources of financial or non-financial support for the review, and the role of the funders or sponsors in the review.	Other information, pg20
Competing interests	26	Declare any competing interests of review authors.	Other information, pg20
Availability of data, code and other materials	27	Report which of the following are publicly available and where they can be found: template data collection forms; data extracted from included studies; data used for all analyses; analytic code; any other materials used in the review.	Other information, pg20

From: Page MJ, McKenzie JE, Bossuyt PM, Boutron I, Hoffmann TC, Mulrow CD, et al. The PRISMA 2020 statement: an updated guideline for reporting systematic reviews. BMJ 2021;372:n71. doi: 10.1136/bmj.n71

For more information, visit: http://www.prisma-statement.org/

For peer review only

BMJ Open

A systematic review of shared decision-making interventions for people living with chronic respiratory diseases

Journal:	*BMJ Open*
Manuscript ID	bmjopen-2022-069461.R1
Article Type:	Original research
Date Submitted by the Author:	24-Feb-2023
Complete List of Authors:	Barradell, Amy; University of Leicester, Department of Respiratory Sciences; University Hospitals of Leicester NHS Trust, Centre for Exercise and Rehabilitation Science<br>Gerlis, Charlotte; University Hospitals of Leicester NHS Trust, Centre of Exercise and Rehabilitation Science<br>Houchen-Wolloff, Linzy; University Hospitals of Leicester NHS Trust, Centre of Exercise and Rehabilitation Science<br>Bekker, Hilary; University of Leeds, Leeds Institute of Health Sciences; Central Denmark Region and Aarhus University, Research Centre for Patient Involvement<br>Robertson, Noelle; University of Leicester, School of Psychology<br>Singh, Sally; University Hospitals of Leicester NHS Trust, Cardiac/Pulmonary Rehabilitation; University of Leicester, Respiratory Sciences
**Primary Subject Heading**:	Respiratory medicine
Secondary Subject Heading:	Patient-centred medicine, Research methods
Keywords:	RESPIRATORY MEDICINE (see Thoracic Medicine), REHABILITATION MEDICINE, Quality in health care < HEALTH SERVICES ADMINISTRATION & MANAGEMENT, Decision Making, Chronic airways disease < THORACIC MEDICINE

SCHOLARONE™
Manuscripts

BMJ

I, the Submitting Author has the right to grant and does grant on behalf of all authors of the Work (as defined in the below author licence), an exclusive licence and/or a non-exclusive licence for contributions from authors who are: i) UK Crown employees; ii) where BMJ has agreed a CC-BY licence shall apply, and/or iii) in accordance with the terms applicable for US Federal Government officers or employees acting as part of their official duties; on a worldwide, perpetual, irrevocable, royalty-free basis to BMJ Publishing Group Ltd ("BMJ") its licensees and where the relevant Journal is co-owned by BMJ to the co-owners of the Journal, to publish the Work in this journal and any other BMJ products and to exploit all rights, as set out in our licence.

The Submitting Author accepts and understands that any supply made under these terms is made by BMJ to the Submitting Author unless you are acting as an employee on behalf of your employer or a postgraduate student of an affiliated institution which is paying any applicable article publishing charge ("APC") for Open Access articles. Where the Submitting Author wishes to make the Work available on an Open Access basis (and intends to pay the relevant APC), the terms of reuse of such Open Access shall be governed by a Creative Commons licence – details of these licences and which Creative Commons licence will apply to this Work are set out in our licence referred to above.

Other than as permitted in any relevant BMJ Author's Self Archiving Policies, I confirm this Work has not been accepted for publication elsewhere, is not being considered for publication elsewhere and does not duplicate material already published. I confirm all authors consent to publication of this Work and authorise the granting of this licence.

For peer review only

SDM Systematic Review

A systematic review of shared decision-making interventions for people living with chronic respiratory diseases

Authors

Barradell, AC[1-3]; Gerlis, C[3]; Houchen-Wolloff L[1,3]; Bekker, HL[5,6]; Robertson, N[4]; Singh, SJ[1,3].

Author affiliations:

1. Department of Respiratory Sciences, University of Leicester, University Road, Leicester LE1 7RH
2. National Institute for Health Research (NIHR) Applied Research Collaboration (East Midlands), College of Medicine, Biological Sciences & Psychology, Leicester General Hospital, Gwendolen Road, Leicester, LE5 4PW
3. Centre for Exercise and Rehabilitation Science, Leicester Biomedical Research Centre-Respiratory, Glenfield Hospital, Groby Road, Leicester, LE3 9QP
4. Department of Neuroscience, Psychology and Behaviour, University of Leicester, University Road, Leicester LE1 7RH
5. Leeds Unit of Complex Intervention Development (LUICD), School of Medicine, University of Leeds, Leeds, LS2 9JT
6. Research Centre for Patient Involvement, Central Denmark Region and Aarhus University, DK-8000 Aarhus C

Corresponding author:

Name: Amy C Barradell

Address: Centre for Exercise and Rehabilitation Science, Leicester Biomedical Research Centre-Respiratory, Glenfield Hospital, Groby Road, Leicester, LE3 9QP

Email: ab1081@le.ac.uk

Tel: 0116 258 3035

Short running title

A systematic review of SDM interventions in CRD

For peer review only

For peer review only - http://bmjopen.bmj.com/site/about/guidelines.xhtml

Disclosure statement

The National Institute for Health Research (NIHR Applied Research Collaboration [East Midlands] (NIHR ARC East Midlands) and the Centre for Exercise and Rehabilitation Science (CERS) funded this research. Award/Grant number is not applicable. The funders had no role in the inception, data extraction or analysis phases of this review. The views expressed in this article do not communicate an official position of the University of Leicester, NIHR, CERS, University of Leeds, or Aarhus University.

For peer review only

For peer review only - http://bmjopen.bmj.com/site/about/guidelines.xhtml

Abstract

Objective

Shared Decision-Making (SDM) supports patients to make informed and value-based decisions about their care. We are developing an intervention to enable healthcare professionals to support patients' PR decision-making. To identify intervention components we needed to evaluate others carried out in Chronic Respiratory Diseases (CRD). We aimed to evaluate the impact of SDM interventions on patient decision-making (primary outcome) and downstream health-related outcomes (secondary outcome).

Design:

We conducted a systematic review using the risk of bias (Cochrane ROB2, ROBINS-I) and certainty of evidence (GRADE) tools.

Data sources:

MEDLINE, EMBASE, PSYCHINFO, CINAHL, PEDRO, Cochrane Central Register of Controlled Trials, the International Clinical Trials Registry Platform Search Portal, ClinicalTrials.gov, PROSPERO, ISRCTN were search through to 14th December 2021.

Eligibility criteria:

Trials evaluating SDM interventions in patients living with CRD using quantitative or mixed methods were included.

Data extraction and synthesis:

Two independent reviewers extracted data, assessed risk of bias, and certainty of evidence. A narrative synthesis, with reference to the The Making Informed Decisions Individually and Together (MIND-IT) model, was undertaken.

For peer review only

For peer review only - http://bmjopen.bmj.com/site/about/guidelines.xhtml

Results:

Eight studies [n=1,596 (of 17,466 citations identified)] fulfilled the inclusion criteria.

Five studies included components targeting the patient, healthcare professionals and consultation process (demonstrating adherence to the MIND-IT model). All studies reported their interventions improved patient decision-making and health-related outcomes. No outcome was reported consistently across studies. Four studies had high risk of bias, three had low quality of evidence. Intervention fidelity was reported in two studies.

Conclusions:

These findings suggest developing a SDM intervention including a patient decision aid, healthcare professional training, and a consultation prompt could support patient PR decisions, and health-related outcomes. Using a complex intervention development and evaluation research framework will likely lead to more robust research, and a greater understanding of service needs when integrating the intervention within practice.

Strengths and limitations

- The inclusion of studies from across the chronic respiratory disease population enabled us to draw conclusions about the efficacy of shared decision-making within this population when other reviews of specific respiratory conditions have been unable to.
- The search criteria may have meant we missed studies which used alternative terminology for shared decision making.
- We were unable to perform meta-analysis or sub-group analysis due to the heterogeneity of included studies and their outcome measures.

Medical Subject Headings

Informed decision making

Decision support

Patient-centred care

Professional-patient relations

For peer review only - http://bmjopen.bmj.com/site/about/guidelines.xhtml

Lung disease

Registration:

The systematic review protocol was registered on PROSPERO on 8th April 2020 (ID: CRD42020169897).

Primary source of funding:

This project is funded by the National Institute for Health Research (NIHR) Applied Research Collaboration East Midlands (ARC EM) and the Centre for Exercise and Rehabilitation Science (CERS). Award/Grant number is not applicable. The views expressed are those of the authors and not necessarily those of the NIHR or the Department of Health and Social Care.

For peer review only

For peer review only - http://bmjopen.bmj.com/site/about/guidelines.xhtml

Introduction

Patients living with chronic obstructive pulmonary disease (COPD) account for a significant proportion of death and disability worldwide[1,2]. Pulmonary rehabilitation (PR) is a recommended evidence-based intervention to improve the physical and psychological health of this population[3] but patients express significant barriers to accepting PR[4,5]. It is therefore proposed, healthcare professionals should seek to engage patients in informed decisions about their enrolment into a programme[5,6].

Shared Decision-Making (SDM) is a core principle of personalised care, which encourages healthcare professionals to actively engage patients in healthcare decisions[7]. This style of communication requires patients and healthcare professionals to share knowledge about the health condition (i.e., the lived experience and evidence-based treatment), then engage in a period of deliberation to review the pros and cons of each option for the patients' life and agree upon an optimal healthcare choice.

This systematic review will inform the development and evaluation of a SDM intervention enabling healthcare professionals to support patients living with COPD make informed decisions about PR. SDM can be viewed as a complex intervention as it requires engagement from multiple stakeholders to enhance communication within a consultation. The Making Informed Decisions Individually and Together (MIND-IT) model[8] provides a conceptual framework to represent components within a multiple stakeholder decision support intervention and its evaluation (Figure 1). There are a range of interventions currently supporting SDM in consultations, including Patient Decision Aids (PtDAs) with or without decision coaching, and SDM training for healthcare professionals.

PtDAs are resources providing accurate information to help people make informed, value-based decisions between healthcare options[7]. They are informed by decision science research to structure the content in a way that reduces the cognitive effort needed to process facts, present balanced and neutral information, and provide prompts encouraging people to trade-off their

For peer review only

116 evaluations of the consequences, and choose an option that is best for their life[9]. The integration
117 of PtDAs into multiple healthcare contexts has shown to improve the frequency of SDM and patient-
118 centred decisions, increase patient knowledge of their condition and the options available, enhance
119 involvement in the decision-making process, and reduce decisional conflict[10–14].

120 Decision Coaching training for healthcare professionals aims to develop their skills in supporting
121 patients to reason between healthcare options rather than providing information about a medical
122 best treatment plan[15]. The training guides healthcare professionals to adopt a non-directive
123 approach in SDM consultations by supporting patients to consider all available options, preparing
124 them for deliberating over options, and ensuring any chosen option is then implemented. Research
125 suggests decision coaching supports patient engagement in SDM[13,16–18] and in combination with
126 a PtDA, is a robust method to facilitate SDM[16].

127 SDM training for healthcare professionals aims to help them adopt a SDM approach to delivering
128 care[19]. There is no consensus guiding the content of this training, however NICE offers a training
129 package[19] which includes a range of knowledge and skills-based modules on patient-centred care,
130 presenting risk information and uncertainty, decision and cognitive science, evidence-based
131 medicine, multiple stakeholder collaboration, and consultation skills. The evidence evaluating the
132 effectiveness of this training on SDM use and impact in practice is weak due to the heterogeneity in
133 study designs, methodology, and evaluation strategies[16,17].

134 SDM interventions are assessed using multiple outcome measures and include: within-consultation
135 communication using healthcare professionals observation (e.g. the Observer OPTION 5 tool[20])
136 and patient self-report questionnaires (e.g. the Shared Decision-Making Questionnaire[21]), patient
137 knowledge of healthcare options, attitudes, and skills (e.g. self-reported perception of risk, the
138 Values' scale[22], the Decision Self Efficacy Scale[23]), patient decision outcomes (e.g. the choice
139 made, the Decisional Conflict Scale[24], the Decision Regret Scale[25]), overall SDM experience from
140 patient and healthcare professionals perspectives (e.g. the iSHARE tool[26]), and downstream

For peer review only - http://bmjopen.bmj.com/site/about/guidelines.xhtml

patient health-related outcomes (e.g. medication/treatment adherence, healthcare utilisation, disease knowledge, and quality of life measures).

In this systematic review we sought to evaluate the efficacy of SDM interventions compared to usual care in patients living with Chronic Respiratory Disease (CRD). To date, the efficacy of SDM interventions for adults living with CRD is largely unknown. Two systematic reviews have been published to synthesise evidence on the impact of SDM interventions on people with asthma[27] and cystic fibrosis[28] but they found the evidence base to be too heterogeneous to draw meaningful conclusions about the relationship between the interventions and improved SDM or downstream health-related outcomes and nearly all the review population were children and adolescents. Therefore, there is a clear need to evaluate the efficacy of SDM interventions upon adult patients living with Chronic Respiratory Disease (CRD; e.g. COPD, asthma, idiopathic pulmonary fibrosis, lung cancer).

In this review, we aim to identify the components used to support SDM within this health context and synthesise evidence of their impact on patient decision-making and downstream health-related outcomes. We use the Preferred Reporting in Systematic Reviews and Meta-Analyses (PRISMA) guidelines to report this review[29].

(Insert Figure I)

Methods

Eligibility criteria

Participants

Adults (aged 18+ years) diagnosed with any CRD (and their carers).

Intervention

Any intervention which orchestrated SDM between patients and healthcare professionals, with or without disease education, decision coaching training, or SDM training. For the purpose of this

For peer review only - http://bmjopen.bmj.com/site/about/guidelines.xhtml

165 review, interventions were accepted if they were patient-facing and purposely stated that were to
166 support SDM between a patient and healthcare professional.

167 *Comparison*

168 Any concurrent control groups enrolled in the included studies, but not receiving a SDM
169 intervention.

170 *Outcomes*

171 Primary outcomes of interest included measures of the quality of patient decision-making (e.g.
172 participants' preparedness for decision-making, healthcare professionals and patient involvement in
173 the decision-making process, knowledge of the available options and associated risks, decisional
174 conflict, concordance between the participants' values and the decision made, decision confidence,
175 and decisional regret).

176 Secondary outcomes of interest were patients' downstream health-related outcomes (e.g.
177 behavioural activation, quality of life, knowledge of condition, healthcare utilisation). Specific
178 qualitative measures of healthcare professionals' attitudes and experience of the SDM intervention,
179 attendance, attrition, and fidelity were also included.

180 *Study design*

181 Randomised and non-randomised quantitative or mixed method controlled trials were sought for
182 inclusion in this review. This included cluster trials, interrupted time series (ITS) studies with at least
183 one data point before and after the intervention and controlled before-after (CBA) studies.

184 Exclusions were hypothetical decision scenarios, and studies not reported in English.

185 **Information sources**

186 An electronic search was conducted on the review's inception until 14th December 2021 using the
187 following databases and registries: MEDLINE (1966-), EMBASE (1947-), PSYCHINFO (1887-), CINAHL
188 (1937-), PEDRO (1999-), Cochrane Central Register of Controlled Trials (1993-) the International

For peer review only

For peer review only - http://bmjopen.bmj.com/site/about/guidelines.xhtml

Clinical Trials Registry Platform Search Portal (2006-), ClinicalTrials.gov (2000-), PROSPERO (2011-), ISRCTN (2000-). Reference lists of selected studies and relevant systematic reviews were also searched to identify further articles for inclusion.

Search strategy

The search algorithm (Supplementary Material) was developed and piloted with support from the University Hospitals of Leicester NHS Trust Library Services. The algorithm was piloted using the MEDLINE database with limits imposed to retrieve articles in English. The algorithm was adapted to the syntax and MESH headings for each database.

Selection process

Articles were uploaded to the web-based platform, Rayaan (https://rayyan.qcri.org/welcome) to facilitate independent review. Duplicate articles were removed and articles reporting findings across multiple outputs were highlighted to review in tandem. Two reviewers (AB, CG) screened titles and abstracts using the inclusion and exclusion criteria. Eligible full texts were reviewed by the same two authors. Any ambiguity in the eligibility of an article was discussed and resolved with the co-authors.

Data collection process

A review-specific data extraction form was used, informed by Cochrane data extraction forms[30]. This was piloted prior to use to ensure consistency in the extraction process. One reviewer (AB) conducted data extraction. A random 10% selection was reviewed by a co-author (CG) to validate the extracted data. Since there were no errors in data extraction were identified, no further validation was considered necessary. Ambiguity in the extracted data was discussed by all authors and resolved by contacting individual study teams. Specific data items for primary and sub-group analysis are outlined in the Supplementary Material.

For peer review only

For peer review only - http://bmjopen.bmj.com/site/about/guidelines.xhtml

Study risk of bias assessment

RCTs were assessed using the Cochrane Risk of Bias tool[31,32] which measures the following domains: randomisation process, deviations from intended interventions, missing outcome data, measurement of the outcomes, and selection of the reported result. Non-RCTs were assessed using the Cochrane ROBINS-I tool[33] which measures the following domains: confounding, selection of study participants, classification of interventions, deviations from intended interventions, missing data, measurement of outcomes, and selection of the reported result. Two authors (AB, CG) independently rated the studies. Any discrepancy in judgements were reviewed and resolved by the co-authors.

Certainty of evidence

The strength of evidence for all reported outcomes was assessed using the Grading of Recommendations Assessment, Development & Evaluation working group methodology (GRADE) which measures the following domains: risk of bias, inconsistency, indirectness, imprecision, and publication bias. Initial judgements were based upon study designs and then systematically reduced if the quality of evidence was poor or increased if a significant effect, dose response or evidence of the elimination of all plausible residual confounding variables and bias was observed. Two authors (AB, CG) independently assessed the criteria. Any discrepancies were resolved with the co-authors.

Synthesis methods

Statistical analysis of the results was not appropriate due to the clinical and methodological heterogeneity of the outcome measures between studies. A narrative synthesis, with reference to the MIND-IT model, was undertaken to identify the components used to support SDM in CRD and synthesise the evidence of their impact on patient decision-making and downstream health-related outcomes both between and within included studies. Guidance on the conduct of narrative synthesis in systematic reviews [34] was consulted to guide this process.

For peer review only

For peer review only - http://bmjopen.bmj.com/site/about/guidelines.xhtml

All outcomes were tabulated with continuous data presented as mean (standard deviation) and categorical data as percentage proportions. To allow consistency in reporting and synthesis of the results, data that was not published as means and standard deviations (e.g. instead reported as means and confidence intervals (CIs)) were transposed to this format using the formula reported in chapter 6 of the Cochrane Handbook for Systematic Reviews of Interventions (Version 6.3, 2022; (Higgins et al., 2022). These are acknowledged with an '*' in Table I.

Patient and Public Involvement

No patient and public involvement occurred during the development of the systematic review's research question, the choice of outcomes measures, the design and implementation of the review, or the interpretation of the results.

Results

Study selection

The search generated 17,466 articles. Following the removal of duplicates, 15,861 articles were retained for abstract screening. Of these, 117 underwent full text screening and eight were retained for inclusion in this analysis (109 excluded). Most studies were excluded because of the participant population (Figure II). A full list of excluded studies is provided in the Supplementary Material.

(Insert Figure II)

Study characteristics

Characteristics of the eight included studies[35–42] are provided in Tables I and II (an extended version of Table I is provided in the Supplementary Material). Six studies were RCTs[35,37–41], two were non-RCT[36,42]. Two studies were conducted with COPD outpatients[35,40], two with COPD inpatients[37,39], two with lung cancer outpatients[36,42], and two with asthma outpatients[38,41]. The theoretical underpinning of interventions, reported by study authors, included social cognitive theory[35], principles of self-management and SDM[37], SDM[38,41,42], self-management[39] and

For peer review only - http://bmjopen.bmj.com/site/about/guidelines.xhtml

cognitive behavioural psychology[40]. One study[36] did not report a theoretical underpinning. The interventions of five studies included all three components of the MIND-IT multiple stakeholder model (Figure III). Four studies included additional components to SDM within their intervention groups; two included a self-management programme[39,40], and three included an educational component[37,38,40].

(Insert Tables I and II)

(Insert Figure III)

Risk of bias within studies

Two RCTs were judged to have high risk of bias[37,40] and four RCTs were judged to have some concerns [35,38,39,41] (Figure IV). One study was judged as high due to insufficient efforts in randomisation, blinding and attrition[37], the other was due to attrition and poor fidelity[40]. The two non-RCTs were both judged to have serious risk of bias because of insufficient efforts in blinding[36,42] (Figure V).

(Insert Figure IV and V)

Results of synthesis

Results of individual studies are presented in turn alongside certainty of evidence assessments in Table I.

Decision-making outcomes

Five outcomes captured the quality of patient decision-making (Table I); quality of communication, involvement in decision-making, decisional conflict, strength of treatment preference, and knowledge of treatment options.

Quality of communication

One study reported that quality of communication increased in intervention and control groups, however, a significantly greater increase occurred in the intervention group alone [mean between

For peer review only - http://bmjopen.bmj.com/site/about/guidelines.xhtml

group difference: 5.7 points; p<0.05][35]. The same study reported an increase in the occurrence of patient-centred communication in the intervention group alone, as measured by a study-specific 4-item self-reported questionnaire [mean(SD) Item 1. Intervention: 35.2(74.6) Control: 15.9(58.2) p<0.05; Item 2. Intervention: 60.3(122.9) Control: 30.8(175.5) p<0.05; Item 3. Intervention: 53.6(115.1) Control: 45.2(73.3) p>0.05; Item 4. Intervention: 86. 2(84.6) Control: 75.2(115.0) p<0.05].

Involvement in decision-making

Two studies reported significantly increased patient involvement in the decision-making process when compared to a control group [6months mean(SD) between group difference: 0.4(0.2), p<0.05; 12 months mean(SD) between group difference: 0.3(0.1), p<0.05][40] and a group where clinicians made treatment decisions [immediately post-intervention mean(SD) between group difference: 2.5(0.9), p<0.05][41].

Decisional conflict

Two studies (one feasibility, one pilot controlled before and after study) reported a trend for reducing decisional conflict. The first reported 87% reduction in decisional conflict[36], the other reported a mean difference of +1 indicating increased decision certainty[42]. Elsewhere, a RCT measuring disease education with and without a patient decision aid reported significantly reduced decisional conflict in the intervention and control groups [Intervention mean(SD) difference from baseline: 8.1(22.5), p<0.05; Control mean (SD) difference from baseline: 9.1(27.7), p<0.05], but no significant mean difference between groups [between group mean difference: 2.5, p>0.05)[38].

Strength of treatment preference

One feasibility study reported the strength of patients' treatment preference increased from 80-100% following exposure to a patient decision aid[36].

Knowledge of treatment options

One feasibility study reported an increasing trend for treatment specific knowledge for three year outcome survival[a] and median survival outcome[b] [Knowledge of survival outcomes with treatment 1

For peer review only

For peer review only - http://bmjopen.bmj.com/site/about/guidelines.xhtml

309 alone (mean between group difference: 60%[a]; 54%[b]); Knowledge of direction of survival difference
310 between treatments (mean between group difference: 27%[a]; 40%[b]); Knowledge of the magnitude of
311 survival difference between treatments (mean between group difference: 73%[a]; 67%[b])][36].

312 A pilot controlled before and after study reported awareness of treatment options increased from
313 40% to 100% of participants post intervention[42].

314 *Health-related outcomes*

315 Six outcomes captured downstream health-related outcomes (Table I); disease knowledge,
316 hospitalisations, behavioural activation quality of life, intervention fidelity, attendance, and attrition.

317 Disease knowledge

318 One study reported significantly increased disease-specific knowledge scores in the intervention
319 group [mean(SD) between group difference: 3.89(0.7), p<0.05] which maintained at 3 months follow
320 up [mean(SD) between group difference: 3.88(0.8), p<0.05][39]. However, another study reported
321 knowledge scores increased equally amongst intervention and control groups [mean difference (SD)
322 Intervention: 3.6(8.9), p<0.05; Control: 2(7.3), p<0.05][38].

323 Hospitalisations

324 Two studies reported no change in all-cause hospitalisations between intervention and control
325 groups[37,40]. One study observed a significant reduction in healthcare utilisation in the SDM and
326 clinician decision-making groups but not in the control group [SDM-control mean(SD) between
327 group difference: 20.36(1.5), p<0.05; SDM-clinician decision-making mean(SD) between group
328 difference: 0.01(2.7), p>0.05; clinician decision-making-control mean(SD) between group difference:
329 20.37(1.5), p<0.05][41].

330 Behavioural activation

331 One feasibility study showed both the intervention and control group had a trend for increased
332 levels of activation post intervention [mean(SD) between group difference: 0.52(0.9) and 0.69(1.0)
333 points respectively, p<0.05][37].

For peer review only - http://bmjopen.bmj.com/site/about/guidelines.xhtml

334 One pilot controlled before and after study reported 80% of participants had made a treatment decision at 30 days and 40% had begun a treatment which matched their treatment preference[42].

336 Two studies reported medication adherence significantly improved in the intervention groups alone[39,41]. However, the control group receiving a clinician decision-making intervention in one of these studies[41] also showed significantly adherent medication behaviour and no between group difference [SDM–clinician decision-making mean(SD) between group difference: 0.08(0.4), p>0.05]. One study measured the correct administration of medication and post intervention reported non-significant improvements in the correct use of asthma medication in the intervention and the control group with no significant difference between groups [mean(SD) between group difference: 0.17(0.7), p>0.05][38].

344 One study reported significant between group differences for improvements in nutritional behaviour at 3 months post inpatient intervention initiation [mean between group difference: 5.03; p<0.05) and physical activity at 3 months post intervention initiation (mean between group difference: 716.01; p<0.05][39].

348 Quality of life

349 Two studies reported no significant difference between intervention and control groups post intervention [Discharge mean(SD) between group difference: 6.15(27.5), p>0.05); 6 months mean(SD) between group difference: 1.9(2.7), p>0.05); 12 months mean(SD) between group difference: 1.4(1.5), p>0.05][39,40]. However, one of the studies did at 3 months post discharge [mean(SD) between group difference: 8.28(50.6), p<0.05][39]. Whilst two further studies reported either a trend for improvement [mean(SD) between group difference: 4.89; p<0.05)[37] or a significant improvement in the intervention group alone [SDM to control mean(SD) between group difference: 0.39(1.0), p<0.05][41].

For peer review only - http://bmjopen.bmj.com/site/about/guidelines.xhtml

Intervention fidelity

Only two studies reported intervention fidelity explicitly[40,41]. Both studies rated a specified selection of intervention audio recordings. One reported high adherence to the intervention protocol [SDM, 4/5; clinician decision-making, 3.9/5][41]. The other reported wide variation in adherence between the different intervention components, with lower delivery of cognitive-behavioural components which were described as the backbone of the intervention[40].

Attendance and attrition

For inventions involving single sessions, there was no attrition during intervention delivery[35,36,38,42]. For interventions involving multiple sessions, completion of inpatient interventions ranged from 37%[37] to 100%[39], whereas, outpatient interventions ranged from 72%[40] to 75.5%[41].

Results from sub-group analysis are provided in the Supplementary Material.

Discussion

This review provides the first synthesis of studies evaluating interventions to support SDM between patients with CRD and healthcare professionals. All eight studies reported their interventions improved patient decision-making and downstream health-related outcomes. There were improvements in the quality of communication with healthcare professionals[35], the occurrence of patient-centred communication[35], involvement in decision-making[40,41], decisional conflict[38,42], health-related knowledge[39], behavioural activation[39,41,42], quality of life[39,41], and healthcare utilisation[41]. There was no one consistent outcome measure used in all studies to enable comparison across studies. Additionally, three studies measured intervention feasibility and therefore were unable to observe intervention efficacy[36,37,42].

The most consistently reported outcomes were the downstream health-related outcomes behavioural activation and quality of life. Five studies reported improvements in behavioural activation across six outcome measures[37–39,41,42]. Between group differences were only

For peer review only - http://bmjopen.bmj.com/site/about/guidelines.xhtml

observed for two of the outcomes (nutritional status and physical activity) and this was in just one study[39]. Two studies reported improved health-related quality of life post intervention[37,41] but one of these was a feasibility study[37].

Unlike other reviews in the CRD field[27,28], this review found variation in the intervention components. Our appraisal showed that only five studies contained all active SDM components from the MIND-IT model (Figure III). Gagne's[38] and Au's[35] study did not report a period of deliberation between patients and healthcare professionals, suggesting the decision-making process was not shared, and in Walters[40] study, there was no report of patients and healthcare professionals exchanging personal and evidence-based information suggesting the decision-making process was not informed. Without adequate inclusion of all SDM components, it is difficult to conclude which intervention components support which outcomes.

Furthermore, there was little evidence in the descriptions of the interventions to show they had been developed in-line with recommended SDM intervention and user-centred design guidelines (e.g. [43],[44] and [45]). There was also limited evidence to suggest included studies had identified their target populations' (e.g. patient or healthcare professionals) needs when developing their intervention and its evaluation. For example, despite the vast array of participants' educational attainment across studies, there was little evidence studies observed this and tailored the SDM materials accordingly. This observation is of concern because lower health literacy has been shown to affect key decision-making outcomes[46]. A recent expanded model of SDM has been proposed to reflect the necessity of patients' health literacy skills as this enables them to have meaningful engagement in the decision-making process[47]. In our review, no study had considered the health literacy of their participants prior to enrolment through the use of validated measures (e.g. test of functional health literacy in adults[48]), nor the use of a standardising health literacy resources such as the Plain English Campaign[49] to ensure materials were at the appropriate comprehension level and, finally, no study had considered using the low literacy version of the Decisional Conflict

For peer review only

For peer review only - http://bmjopen.bmj.com/site/about/guidelines.xhtml

Scale[50], despite its ease of access. Furthermore, no study reported healthcare professional-directed intervention components (e.g. healthcare professional training) or used a user-centred design or implementation process.

Additionally, studies made little reference to social or familial support in the decision-making process, which is unusual as social support is often associated with improvements in self-management behaviour amongst patients with CRD[51,52]. Social support may contribute to informed information exchange and provide confidence in the decision-making process. Interestingly, one study specifically excluded family members or friends from the SDM consultation as they believed it would introduce bias[36].

Limitations of the evidence included in the review

The interpretation of these results should be considered in light of the inclusion of studies which utilised non-validated outcome measures. Only one study used a dose-matched intervention in the control group[40] and fidelity was only reported explicitly in two studies[40,41].

Limitations of the review process

The inconsistent outcome measures within this review precluded meta-analysis. Four studies had a high risk of bias[36,37,40,42] and three had low or very low quality of evidence[36,40,42], thereby limiting the validity and generalisability of the results. The search criteria were broad, however, if studies had used alternate terminology for SDM, it is possible some may have been missed. The multi-component interventions meant intervention effects could not be discernibly attributed to the SDM component or the education and self-management components. Furthermore, two studies both provided their control groups with disease education which may have driven the positive effects observed by the control groups[37,38]. Included studies encompassed a variety of healthcare settings with decisions varying in focus from self-management to treatment decision-making and end of life care. Despite excluding studies involving hypothetical decision-making to reduce the disparity in importance attributed to the decision-making process, we cannot assume participants

For peer review only

For peer review only - http://bmjopen.bmj.com/site/about/guidelines.xhtml

attributed the same importance to all decisions. Additionally, the studies length of follow-ups varied considerably, however, as the type of decisions were so varied the allocated follow-up period may have been appropriate. For example, Janssen and colleagues showed that patients with COPD change their mind about end of life care throughout the course of their healthcare journey[53], suggesting a long-term follow-up period for end of life decision-making is appropriate. Whereas health decisions with more immediate affect (e.g. acceptance of a referral to pulmonary rehabilitation) may require a shorter follow-up period. As more SDM intervention studies are conducted in CRD, an updated review may well benefit from statistical sub-group analysis for studies length of follow up and/or decision posed. Additionally, the decision to exclude qualitative and cross-sectional studies may have limited our ability to fully explore participants' experiences of the SDM process and the development and implementation of interventions.

Implications of the results

This synthesis provides some evidence that CRD SDM interventions improve the patient decision-making process and downstream health-related outcomes. However, the evidence is weak, and the interventions at times incongruent with all active SDM components. Therefore, this limits the rigour in evaluating the impact of SDM interventions within CRD. There were also no studies exploring the efficacy of SDM in PR decision-making and implementation in practice.

Based on the available evidence, this review suggests the development of a SDM intervention to enable healthcare professionals to support COPD patients informed decision-making for PR may be beneficial. It recommends that such intervention should be evaluated within a complex intervention development and evaluation research framework. This is especially relevant given the rise in alternate PR delivery models due to the Covid-19 pandemic. Importantly, this intervention should be underpinned by SDM theoretical models (e.g. MIND-IT[8], extended SDM model involving consideration of health literacy[47]), adhere to the appropriate intervention development guidelines (e.g.[43–45]), and be evaluated in a single or double-blind RCT with dose-matched controls.

For peer review only

For peer review only - http://bmjopen.bmj.com/site/about/guidelines.xhtml

Other information

Protocol and registration

This review was conducted in accordance with the Preferred Reporting in Systematic Reviews and Meta-Analyses (PRISMA) and registered on PROSPERO (registration number: CRD42020169897). The completed PRISMA checklist is provided in the Supplementary Material.

Support

The National Institute for Health Research (NIHR Applied Research Collaboration [East Midlands] (NIHR ARC East Midlands) and the Centre for Exercise and Rehabilitation Science (CERS) funded this research. Award/Grant number is not applicable.

Competing interests

The authors have no conflicts of interest to declare.

Availability of data, code, and other materials

The MEDLINE search strategy, data items sought, methods for sub-group analysis, characteristics of excluded studies, and results of sub-group analysis are provided in the Supplementary Materials. No additional data available.

Acknowledgements

The authors would like to thank the UHL Library Services for their contribution to the search criteria and piloting the search. We would like to thank all study authors for sharing their data, when requested. Professor Singh is a National Institute for Health Research (NIHR) Senior Investigator. The views expressed in this article are those of the author(s) and not necessarily those of the NIHR, or the Department of Health and Social Care.

For peer review only

Contributorship statement

ACB conceived the review. ACB, CG, LHW, HLB, NR and SJS contributed the planning, design, and protocol development. ACB conducted the search in collaboration with the University Hospitals of Leicester NHS Trust Librarian. ACB and CG screened all citations. ACB contacted study authors for additional data acquisition. ACB, CG, LHW, NR and SJS confirmed studies to be included. ACB conducted data extraction and CG conducted a 10% data quality check to ensure accuracy in this process. ACB and CG conducted risk of bias and certainty of evidence assessments. ACB conducted the narrative synthesis and ACB, CG, LHW, NR and SJS contributed to interpretation of the results. ACB drafted the manuscript, and CG, LHW, NR and SJS critically revised it for significant intellectual content and insight. In addition, ACB, CG, LHW, NR and SJS gave final approval of the manuscript version for publication. ACB, CG, LHW, NR and SJS are responsible for the overall content as guarantors. ACB, CG, LHW, NR and SJS report no conflicts of interest.

References

1 Lozano R, Naghavi M, Foreman K, *et al.* Global and regional mortality from 235 causes of death for 20 age groups in 1990 and 2010: A systematic analysis for the Global Burden of Disease Study 2010. *Lancet* 2012;**380**:2095–128. doi:10.1016/S0140-6736(12)61728-0

2 Vos T, Flaxman AD, Naghavi M, *et al.* Years lived with disability (YLDs) for 1160 sequelae of 289 diseases and injuries 1990-2010: A systematic analysis for the Global Burden of Disease Study 2010. *Lancet* 2012;**380**:2163–96. doi:10.1016/S0140-6736(12)61729-2

3 Mccarthy B, Casey D, Devane D, *et al.* Pulmonary rehabilitation for chronic obstructive pulmonary disease. Cochrane Database Syst. Rev. 2015;**23**. doi:10.1002/14651858.CD003793.pub3

4 Keating A, Lee A, Holland AE. What prevents people with chronic obstructive pulmonary disease from attending pulmonary rehabilitation? A systematic review. Chron. Respir. Dis.

For peer review only

For peer review only - http://bmjopen.bmj.com/site/about/guidelines.xhtml

2011;**8**:89–99. doi:10.1177/1479972310393756

5 Keating A, Lee AL, Holland AE. Lack of perceived benefit and inadequate transport influence uptake and completion of pulmonary rehabilitation in people with chronic obstructive pulmonary disease: A qualitative study. *J Physiother* 2011;**57**:183–90. doi:10.1016/S1836-9553(11)70040-6

6 Blackstock FC, Wallack RZ, Nici L, *et al.* Why don't our patients with chronic obstructive pulmonary disease listen to us? the enigma of nonadherence. Ann. Am. Thorac. Soc. 2016;**13**:317–23. doi:10.1513/AnnalsATS.201509-600PS

7 NICE. Shared decision making | NICE guidelines | NICE guidance | Our programmes | What we do | About | NICE. NICE 2021. https://www.nice.org.uk/about/what-we-do/our-programmes/nice-guidance/nice-guidelines/shared-decision-making (accessed 7 Feb 2022).

8 Breckenridge K, Bekker HL, Gibbons E, *et al.* How to routinely collect data on patient-reported outcome and experience measures in renal registries in Europe: An expert consensus meeting. Nephrol. Dial. Transplant. 2015;**30**:1605–14. doi:10.1093/ndt/gfv209

9 Bekker H, Thornton J, Airey C, *et al.* Informed decision making: An annotated bibliography and systematic review. Health Technol. Assess. (Rockv). 1999;**3**. doi:10.3310/hta3010

10 Stacey D, Bennett CL, Barry MJ, *et al.* Decision aids for people facing health treatment or screening decisions. In: Stacey D, ed. *Cochrane Database of Systematic Reviews*. Chichester, UK: : John Wiley & Sons, Ltd 2011. CD001431. doi:10.1002/14651858.cd001431.pub3

11 Stacey D, Légaré F, Lewis K, *et al.* Decision aids for people facing health treatment or screening decisions. Cochrane Database Syst. Rev. 2017;**2017**. doi:10.1002/14651858.CD001431.pub5

12 Hoffman AS, Volk RJ, Saarimaki A, *et al.* Delivering patient decision aids on the Internet:

For peer review only

For peer review only - http://bmjopen.bmj.com/site/about/guidelines.xhtml

Definitions, theories, current evidence, and emerging research areas. BMC Med. Inform. Decis. Mak. 2013;**13**:S13. doi:10.1186/1472-6947-13-S2-S13

13 Sepucha KR, Borkhoff CM, Lally J, *et al.* Establishing the effectiveness of patient decision aids: Key constructs and measurement instruments. *BMC Med Inform Decis Mak* 2013;**13**:S12. doi:10.1186/1472-6947-13-S2-S12

14 Feldman-Stewart D, O'Brien MA, Clayman ML, *et al.* Providing information about options in patient decision aids. *BMC Med Inform Decis Mak* 2013;**13**:S4. doi:10.1186/1472-6947-13-S2-S4

15 Jull J, Köpke S, Smith M, *et al.* Decision coaching for people making healthcare decisions. *Cochrane Database Syst Rev* 2021;:CD013385. doi:10.1002/14651858.CD013385.PUB2/MEDIA/CDSR/CD013385/IMAGE_N/NCD013385-CMP-004.01.SVG

16 Légaré F, Ratté S, Stacey D, *et al.* Interventions for improving the adoption of shared decision making by healthcare professionals. In: *Cochrane Database of Systematic Reviews*. John Wiley & Sons, Ltd 2010. doi:10.1002/14651858.cd006732.pub2

17 Müller E, Diesing A, Rosahl A, *et al.* Evaluation of a shared decision-making communication skills training for physicians treating patients with asthma: A mixed methods study using simulated patients. *BMC Health Serv Res* 2019;**19**:612. doi:10.1186/s12913-019-4445-y

18 Stacey D, Kryworuchko J, Belkora J, *et al.* Coaching and guidance with patient decision aids: A review of theoretical and empirical evidence. *BMC Med Inform Decis Mak* 2013;**13**. doi:10.1186/1472-6947-13-S2-S11

19 NICE. Shared decision making learning package. 2021.https://www.nice.org.uk/guidance/ng197/resources/shared-decision-making-learning-

For peer review only - http://bmjopen.bmj.com/site/about/guidelines.xhtml

package-9142488109 (accessed 7 Feb 2022).

20 Elwyn G, Tsulukidze M, Edwards A, *et al.* Using a 'talk' model of shared decision making to propose an observation-based measure: Observer OPTION5 Item. *Patient Educ Couns* 2013;**93**:265–71. doi:10.1016/j.pec.2013.08.005

21 Simon D, Schorr G, Wirtz M, *et al.* Development and first validation of the shared decision-making questionnaire (SDM-Q). *Patient Educ Couns* 2006;**63**:319–27. doi:10.1016/j.pec.2006.04.012

22 O'Connor A. Sample Tool: Values (Tamoxifen). 1999.www.ohri.ca/decisionaid (accessed 19 Feb 2020).

23 O'Connor AM. Sample Tool: Decision Self-Efficacy Scale. 1995.

24 O'Connor AM. Validation of a Decisional Conflict Scale. *Med Decis Mak* 1995;**15**:25–30. doi:10.1177/0272989X9501500105

25 Brehaut JC, O'Connor AM, Wood TJ, Hack TF, Siminoff L, Gordon E F-SD. Validation of a decision regret scale. *Med Decis Making* 2003;**23**:281–92.

26 Bomhof-Roordink H, Gärtner FR, van Duijn-Bakker N, *et al.* Measuring shared decision making in oncology: Development and first testing of the iSHAREpatient and iSHAREphysician questionnaires. *Heal Expect* 2020;**23**:496–508. doi:10.1111/HEX.13015

27 Kew KM, Malik P, Aniruddhan K NR. Shared decision-making for people with asthma. *Cochrane Database Syst Rev* 2017.

28 Malone H, Biggar S, Javadpour S, *et al.* Interventions for promoting participation in shared decision-making for children and adolescents with cystic fibrosis. *Cochrane Database Syst Rev* 2019;**2019**. doi:10.1002/14651858.CD012578.pub2

For peer review only

29 Page MJ, McKenzie JE, Bossuyt PM, *et al.* The PRISMA 2020 statement: An updated guideline for reporting systematic reviews. BMJ. 2021;**372**. doi:10.1136/bmj.n71

30 Cochrane. Data extraction forms | Cochrane Developmental, Psychosocial and Learning Problems. 2021.https://dplp.cochrane.org/data-extraction-forms (accessed 16 Mar 2021).

31 Sterne JAC, Savović J, Page MJ, *et al.* RoB 2: A revised tool for assessing risk of bias in randomised trials. *BMJ* 2019;**366**:l4898. doi:10.1136/bmj.l4898

32 Higgins J, Thomas J, Chandler J, *et al.* Cochrane Handbook for Systematic Reviews of Interventions version 6.3 (updated February 2022). 2022. Available from www.training.cochrane.org/handbook.

33 Sterne JA, Hernán MA, Reeves BC, *et al.* ROBINS-I: a tool for assessing risk of bias in non-randomised studies of interventions. *BMJ* 2016;**355**. doi:10.1136/BMJ.I4919

34 Popay J, Roberts H, Sowden A, *et al.* Guidance on the conduct of narrative synthesis in systematic reviews. A product from the ESRC methods programme. Version, 1.No Title. 2006. doi:10.13140/2.1.1018.4643

35 Au DH, Udris EM, Engelberg RA, *et al.* A randomized trial to improve communication about end-of-life care among patients with COPD. *Chest* 2012;**141**:726–35. doi:10.1378/chest.11-0362

36 Brundage MD, Feldman-Stewart D, Cosby R, *et al.* Phase I study of a decision aid for patients with locally advanced non-small-cell lung cancer. *J Clin Oncol* 2001;**19**:1326–35. doi:10.1200/JCO.2001.19.5.1326

37 Collinsworth AW, Brown RM, Sjames C, *et al.* The impact of patient education and shared decision making on hospital readmissions for COPD. *Int J COPD* 2018;**13**:1325–32. doi:10.2147/COPD.S154414

For peer review only

For peer review only - http://bmjopen.bmj.com/site/about/guidelines.xhtml

38 Gagné ME, Légaré F, Moisan J, *et al.* Impact of Adding a Decision Aid to Patient Education in Adults with Asthma: A Randomized Clinical Trial. *PLoS One* 2017;**12**:e0170055. doi:10.1371/journal.pone.0170055

39 Granados-Santiago M, Valenza MC, López-López L, *et al.* Shared decision-making and patient engagement program during acute exacerbation of COPD hospitalization: A randomized control trial. *Patient Educ Couns* 2019;**103**:702–8. doi:10.1016/j.pec.2019.12.004

40 Walters J, Cameron-Tucker H, Wills K, *et al.* Effects of telephone health mentoring in community-recruited chronic obstructive pulmonary disease on self-management capacity, quality of life and psychological morbidity: A randomised controlled trial. *BMJ Open* 2013;**3**. doi:10.1136/bmjopen-2013-003097

41 Wilson SR, Strub P, Buist AS, *et al.* Shared treatment decision making improves adherence and outcomes in poorly controlled asthma. *Am J Respir Crit Care Med* 2010;**181**:566–77. doi:10.1164/rccm.200906-0907OC

42 Myers RE, Advani SM, Myers P, *et al.* Engaging Patients with Late-Stage Non-Small Cell Lung Cancer in Shared Decision Making about Treatment. *J Pers Med* 2021;**11**. doi:10.3390/JPM11100998

43 Coulter A, Stilwell D, Kryworuchko J, *et al.* A systematic development process for patient decision aids. *BMC Med Inform Decis Mak* 2013;**13**:S2. doi:10.1186/1472-6947-13-S2-S2

44 Stacey D, Légaré F, Boland L, *et al.* 20th Anniversary Ottawa Decision Support Framework: Part 3 Overview of Systematic Reviews and Updated Framework. Med. Decis. Mak. 2020;**40**:379–98. doi:10.1177/0272989X20911870

45 O'Cathain A, Croot L, Duncan E, *et al.* Guidance on how to develop complex interventions to improve health and healthcare. *BMJ Open* 2019;**9**:e029954. doi:10.1136/bmjopen-2019-

For peer review only - http://bmjopen.bmj.com/site/about/guidelines.xhtml

029954

46 Mccaffery KJ, Holmes-Rovner M, Smith SK, *et al.* Addressing health literacy in patient decision aids. 2012. doi:10.1186/1472-6947-13-S2-S10

47 Muscat DM, Shepherd HL, Nutbeam D, *et al.* Health Literacy and Shared Decision-making: Exploring the Relationship to Enable Meaningful Patient Engagement in Healthcare. *J Gen Intern Med* 2020;**36**:521–5. doi:10.1007/s11606-020-05912-0

48 Parker RM, Baker DW, Willia M V., *et al.* The test of functional health literacy in adults. *J Gen Intern Med 1995 1010* 1995;**10**:537–41. doi:10.1007/BF02640361

49 Plain English Campaign. http://www.plainenglish.co.uk/ (accessed 6 Aug 2021).

50 Linder SK, Swank PR, Vernon SW, *et al.* Validity of a low literacy version of the Decisional Conflict Scale. *Patient Educ Couns* 2011;**85**:521–4. doi:10.1016/j.pec.2010.12.012

51 Chen Z, Fan VS, Belza B, *et al.* Association between Social Support and Self-Care Behaviors in Adults with Chronic Obstructive Pulmonary Disease. *Ann Am Thorac Soc* 2017;**14**:1419–27. doi:10.1513/AnnalsATS.201701-026OC

52 Lind N, Nordin M, Palmquist E, *et al.* Coping and social support in asthma and allergy: the Västerbotten Environmental Health Study. *J Asthma* 2015;**52**:622–9. doi:10.3109/02770903.2014.991970

53 Janssen DJA, Curtis JR, Au DH, *et al.* Patient-clinician communication about end-of-life care for Dutch and US patients with COPD. *Eur Respir J* 2011;**38**:268–76. doi:10.1183/09031936.00157710

For peer review only

For peer review only - http://bmjopen.bmj.com/site/about/guidelines.xhtml

Figure legends

Figure I: MIND-IT model; a framework representing informed, evidence-based, and shared decisions with patients and their healthcare professional (Bekker et al., 2015)

Figure II: PRISMA flow diagram of review process

Figure III: SDM intervention principles aligned to the MIND-IT model (Bekker et al., 2015)

Figure IV: Risk of bias in RCT studies

Figure V: Risk of bias in non-RCT studies

Table I: Summary of studies

Table II: Summary of interventions

For peer review only

For peer review only - http://bmjopen.bmj.com/site/about/guidelines.xhtml

Tables

Table I: Summary of studies

First Author, Year, Country, Sample size, Study design, Population	Outcome(s)	Summary of results	Certainty of evidence (GRADE score)
Au, 2012, USA, Clinicians = 92 Patients = 376, RCT, Outpatients with COPD	*Validated:* Quality of communication (QOC). *Non-validated:* Occurrence of discussions about end of life preferences between patients and either clinician or surrogate.	Baseline quality of communication was poor in both groups. Modest improvements in both groups, but significant mean between group difference favouring the intervention group (5.7 points; p<0.05). The occurrence (%) of patient communication about end of life care was significantly higher in the intervention group for three of four questions (mean(SD)): 1. Int: 35.2(74.6) Con: 15.9(58.2) p<0.05, 2. Int: 60.3(122.9) Con: 30.8(175.5) p<0.05, 3. Int: 53.6(115.1) Con: 45.2(73.3) p>0.05, 4. Int: 86.2(84.6) Con: 75.2(115.0) p<0.05.	Moderate ⊕⊕⊕◯ Moderate certainty because of risk of bias.
Brundage, 2001, Canada, Patients = 20, Feasibility CBA, Outpatients with lung cancer	*Validated:* Decisional conflict (DCS). *Non-validated:* Knowledge of treatment options.	Decisional conflict reduced after receiving the intervention in 87% and increased in 13% of participants. Knowledge of the available treatment options improved following the intervention for both 3-year outcome survival[a] and median survival outcome[b]: 1. Knowledge of survival outcomes with treatment 1 alone (mean between group difference 60%[a]; 54%[b]), 2. Knowledge of direction of survival difference between treatments (mean between group difference: 27%[a]; 40%[b]), 3. Knowledge of the magnitude of survival difference between treatments (mean between group difference: 73%[a]; 67%[b]). The number of participants with a treatment preference increased from 80% to 100% following the intervention.	Low ⊕⊕◯◯ Low certainty because of risk of bias and minor publication bias.
Collinsworth, 2018, USA, Patients = 308, RCT, Inpatients with COPD	*Validated:* Patient activation (PAM), Quality of life health status (CAT). *Non-validated:* All-cause hospital admissions.	Both groups had a significant improvement in PAM scores following the intervention (mean(SD) difference: 0.52(0.9) and 0.69(1.0) points respectively, p<0.05). Only the intervention group had significantly increased scores in the CAT following the intervention (mean(SD) difference: Int: 5.27(10.3), p<0.05; Con: 0.38(7.8), p>0.05). There were no significant differences in the total number of all-cause or COPD-related readmissions between groups at 1, 2, 3, 6 and 9 months (p>0.05).	Moderate ⊕⊕⊕◯ Moderate certainty because of risk of bias.
Gagne, 2017, Canada, Patients = 51, RCT, Outpatients with asthma	*Validated:* Asthma knowledge (QCALF), Decisional conflict. *Non-validated*: Appropriate use of asthma pharmacotherapy.	Both groups had significantly increased knowledge scores following the intervention (mean(SD) difference: Int: 3.6(8.9)*, p<0.001; Con: 2(7.3)*, p<0.05). There was no significant between group difference (p>0.05). Both groups had significantly reduced decisional conflict scores following the intervention (mean(SD) difference: Int: 8.1(22.5)*, p<0.05; Con: 9.1(27.7)*, p>0.05). There was no significant between group difference (p>0.05). Both groups had modest, but non-significant, within group improvements in their appropriate use of pharmacotherapy (mean(SD) difference: Int: 0.15(0.7)*, p>0.05; Con: 0.16(0.7)*, p>0.05). There was no significant between group difference (mean(SD) between group difference = 0.17(0.7), p>0.05).	Moderate ⊕⊕⊕◯ Moderate certainty because of risk of bias.

For peer review only

For peer review only - http://bmjopen.bmj.com/site/about/guidelines.xhtml

Granados-Santiago, 2019, Spain, Patients = 42, RCT, Inpatients with COPD	*Validated:* Quality of life and health status (EQ-5D), COPD knowledge (COPD-Q), Medication adherence (TAI), Physical activity (steps/day), Nutritional status (MNA). *Non-validated:* -	Both groups had significant and clinically important improvements in quality of life and health status at discharge (mean(SD) between group difference: 6.15(27.5)*, p<0.05). Only the intervention group maintained this benefit at 3 months (mean(SD) between group difference: 8.28(50.6)*, p<0.05). Knowledge scores significantly improved in the intervention group at discharge (mean(SD) between group difference: 3.89(0.7)*, p<0.05) and maintained at 3 months (mean(SD) between group difference: 3.88(0.8)*, p<0.05). Adherence significantly improved at discharge in the intervention group (mean(SD) difference: Int: 3.58(1.5)*, p<0.05; Con: 1.36(1.6)*, p>0.05). There was no significant between group difference. At 3 months, there was a significant mean(SD) between group difference favouring the intervention group (1.8(2.4)*, p<0.05). Upon discharge, there was a significant reduction in physical activity in both groups (mean(SD) difference: Int: -348.27(2.0)*, p<0.05; Con: -410.05(4.66)*, p<0.05). At 3 months there was a significant improvement in the intervention group alone (mean(SD) difference: Int: 1371.97(895.2)*, p<0.05; Con: 620.18(12.5)*, p>0.05). At 3 months there was a significant improvement in nutritional status both within and between groups for the intervention group mean(SD) difference: Int: 4.15(0.1)*, p<0.05; Con: -3.43(0.12)*, p>0.05	Moderate ⊕⊕⊕◯ Moderate certainty because of risk of bias.
Myers, 2021, USA, Patients = 5, Pilot CBA, Outpatients with non-small cell lung cancer	*Validated:* Decisional Conflict (DCS SURE Test). *Non-validated:* Knowledge of treatment options, Treatment status.	Decisional conflict reduced post intervention (mean difference=1). Awareness of treatment options increased from 40% to all 100% participants post intervention. At 30 days post intervention, 80% of participants had made a decision. 40% of participants had a treatment that matched their treatment preference.	Low ⊕⊕◯◯ Low certainty because of risk of bias.
Walters, 2013, Australia, Patients = 182, Cluster RCT, Outpatients with COPD	*Validated:* Quality of life (SF-36)., Quality of life (SGRQ), Patient involvement in healthcare decisions (PIH). *Non-validated:* Respiratory hospital admissions, Intervention fidelity.	Neither group had a significant change to either their physical[a] or mental[b] health status at 6 months (mean(SD) between group difference: 1.5(0.2)*[a]; 0.8(1.4)*[b], p>0.05) and 12 months (mean(SD) between group difference: 0.0(0.9)*[a]; 0.3(0.9)*[b], p>0.05). Neither group had a significant change to their quality of life at 6 months (mean(SD) between group difference: 1.9(2.7)*, p>0.05) and 12 months (mean(SD) between group difference: 1.4(1.5)*, p>0.05. The intervention group had a significant improvement in PIH scores at 6 months (mean(SD) between group difference: 0.4(0.2)*, p<0.05) and 12 months (mean(SD) between group difference: 0.3(0.1)*, p<0.05). No significant change in hospital admissions for either group at 12 months (χ^2=2.61, p>0.05). Fidelity assessments confirmed components were addressed with some clarity: 1. COPD symptom management, 49%, 2. Unhelpful self-talk explored and identified, 37%, 3. Unhelpful self-talk challenged, and new self-talk developed, 35%, 4. Action plan for achieving goals made, 54%, 5. Problems and barriers to achieving goals identified and clarified, 46%, 6. Positive changes in behaviour praised, 83%.	Very low ⊕◯◯◯ Very low certainty because of significant risk of bias and some inconsistency.
Wilson, 2010, Hawaii, Patients = 612, RCT, Outpatients with asthma	*Validated:* Quality of life (MiniAQLQ). *Non-validated:* Healthcare use, Patients' perceived role in treatment decision, Medication adherence, Intervention fidelity.	Only intervention groups had significant improvements in quality of life at 12 months (SDM to control mean(SD) between group difference = 0.39(1.0)*, p<0.05; SDM–clinician decision-making mean(SD) between group difference = 0.11(1.1)*, p>0.05; clinician decision-making-control mean(SD) between group difference = 0.28(1.0)*, p<0.05. Only intervention groups had significantly reduced healthcare utilisation (SDM-control mean(SD) between group difference =20.36(-1.5)*, p<0.05; SDM-clinician decision-making mean(SD) between group difference =0.01(2.7)*, p>0.05; clinician decision-making-control mean(SD) between group difference= 20.37(-1.5*), p<0.05. The SDM group rated their influence on the treatment decision as approximately the same as the clinicians' influence (mean(SD)= 3.1(0.6). The SDM group ratings were significantly different from the clinician decision-making group, with the latter feeling their clinician had greater influence (mean(SD)=2.5(0.9), p<0.05). Medication adherence significantly increased only in intervention groups (SDM-control mean(SD) between group difference=0.21(0.5)*, p<0.05; SDM–clinician decision-making mean(SD) between group difference= 0.08(0.4)*, p>0.05; CDM-control mean(SD) between group difference= 0.13(0.4)*, p<0.05, but was not sustained at 2 years (SDM-control mean(SD) between group difference= 0.03(0.4)*, p>0.05; SDM-clinician decision-making mean(SD) between group difference= 0.04(0.4)*, p>0.05; clinician decision-making-control mean(SD) between group difference=-0.01(-0.4)*, p>0.05. Protocol adherence was high (SDM=4.0, clinician decision-making=3.9) and did not differ significantly between groups (p>0.05).	Moderate ⊕⊕⊕◯ Moderate certainty because of risk of bias.

For peer review only - http://bmjopen.bmj.com/site/about/guidelines.xhtml

Table II: Summary of interventions

First Author (Year)	Description of intervention	Interventionist (including any training provided)	Intervention dose	Description of control group
Au (2012)	Consultation using a completed patient-specific feedback form which describes patient preferences regarding end of life care. The feedback form was not endorsed for use.	Clinicians. No specific training described.	Single session.	Routine outpatient COPD care.
Brundage (2001)	Consultation using a patient decision aid to facilitate cancer treatment decision-making.	Researcher. No specific training described.	Single session.	Participant outcomes prior to receipt of intervention.
Collinsworth (2018)	COPD education and SDM self-management planning.	Registered respiratory therapists. No specific training described.	Single session and four telephone calls.	Routine inpatient care and COPD education.
Gagne (2017)	Patient decision aid to facilitate asthma treatment decision-making (and asthma education).	Certified asthma educators from the Quebec Asthma and COPD Network.	Single session.	Routine outpatient asthma care, education, and action plan generation.
Granados-Santiago (2019)	A SDM patient involvement programme focussing on COPD self-management goals to facilitate self-management decision-making.	Healthcare professionals. No specific training described.	Delivered alongside routine inpatient care.	Routine inpatient care for an exacerbation of COPD.
Myers (2021)	Personalised patient decision aid used to facilitate 15-20min decision coaching consultation.	Oncology nurses. No training provided.	Single session.	Participant outcomes prior to receipt of intervention.
Walters (2013)	Psychoeducation about common psychological reactions to COPD diagnosis and treatment, self-management skills training, cognitive coping skills training to identify and challenge negative COPD-related cognitions, communication skills to facilitate discussion between the health mentor and the patient and promoting self-efficacy to manage chronic illness.	Community health nurses, termed 'Health Mentors.' Training included 12 hours of training over 2 days that covered COPD management (1 h), chronic disease self-management and health behaviour change components including practice role plays (7.25 hours), online training and study methods (3.75 hours).	Single session and sixteen telephone calls.	Routine outpatient COPD care and monthly telephone calls for 12 months (excluded intervention components).

For peer review only

For peer review only - http://bmjopen.bmj.com/site/about/guidelines.xhtml

Wilson (2010)	Shared decision-making intervention:<br>Consultation to facilitate asthma treatment decision-making followed by four contacts to assess patient progress and medication changes as needed.	Healthcare professionals involved in routine asthma care (nurses, respiratory therapists, pharmacists, nurse practitioners and physician assistants).<br>Training provided but not described.	Two sessions and 3 telephone calls.	Routine asthma outpatient care (including, in some sites, the opportunity to of referral to an asthma care a management programme).
	Clinician decision-making intervention:<br>Consultation for clinicians to obtains a patient's level of asthma control, prescribe an appropriate treatment regimen and communicate that to the patient.	Healthcare professionals involved in routine asthma care (nurses, respiratory therapists, pharmacists, nurse practitioners and physician assistants).<br>Training provided but not described.	Two sessions and 3 telephone calls.	

For peer review only

For peer review only - http://bmjopen.bmj.com/site/about/guidelines.xhtml

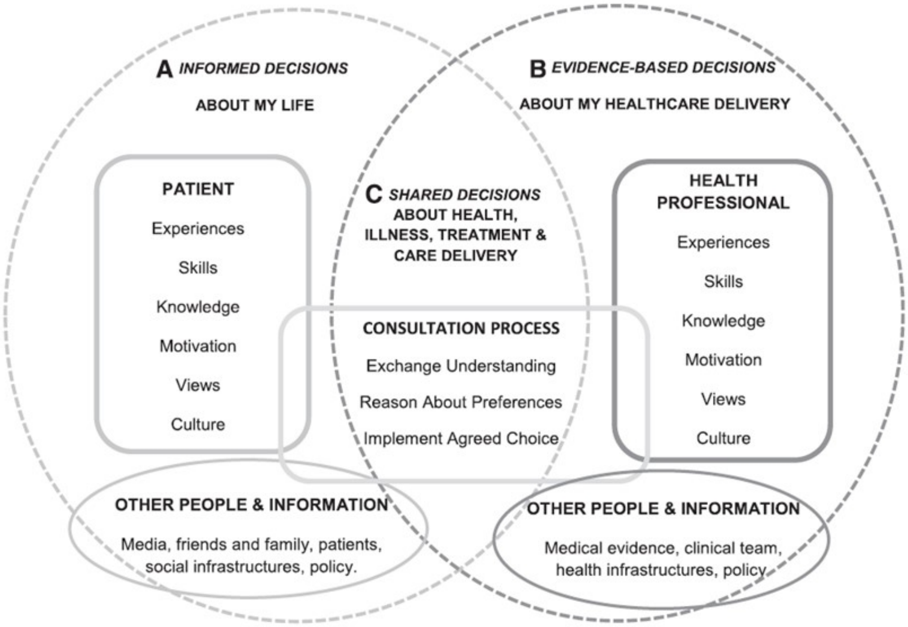

MIND-IT model; a framework representing informed, evidence-based, and shared decisions with patients and their healthcare professional (Bekker et al., 2015)

75x49mm (300 x 300 DPI)

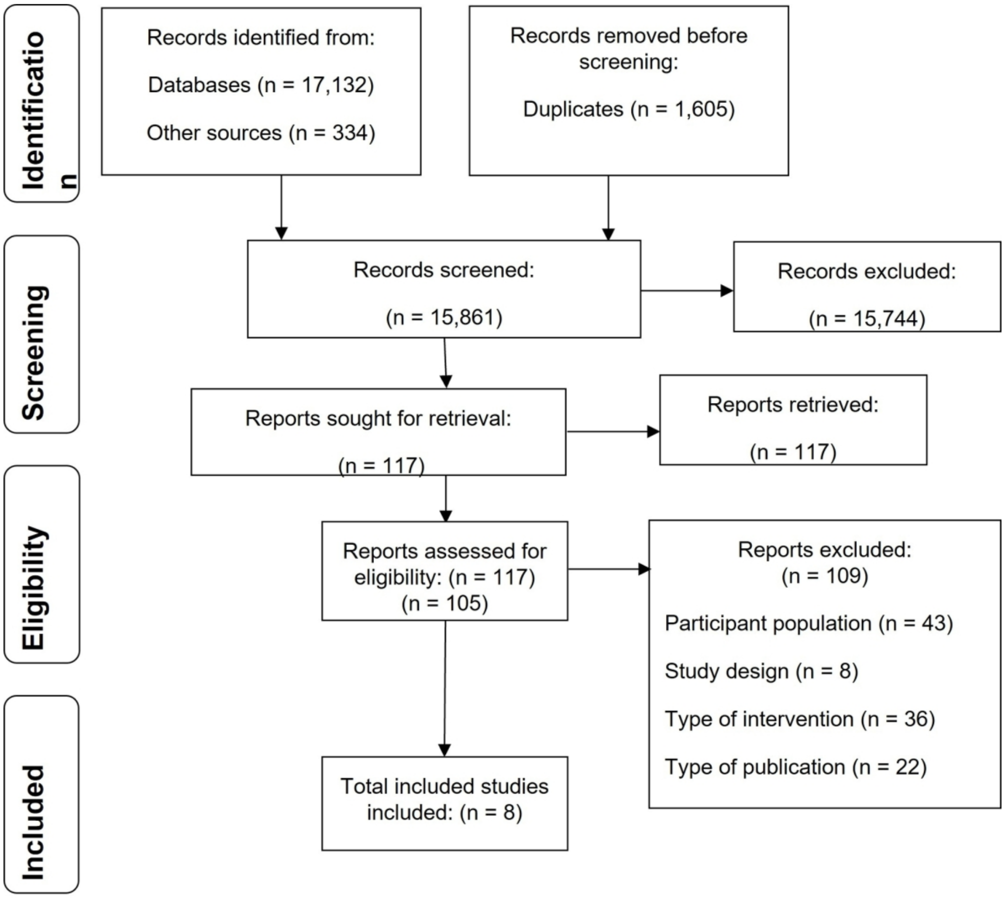

PRISMA flow diagram of review process

162x145mm (300 x 300 DPI)

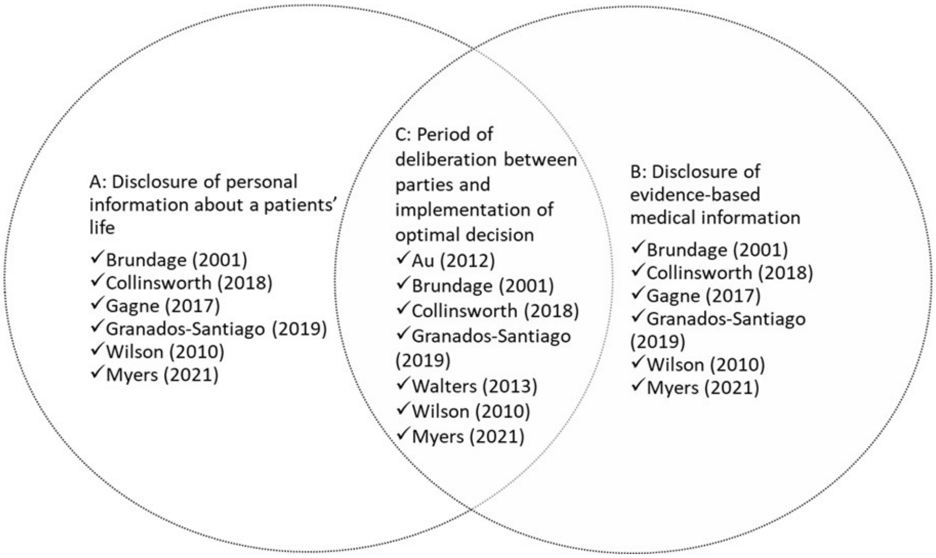

SDM intervention principles aligned to the MIND-IT model (Bekker et al., 2015)

82x45mm (300 x 300 DPI)

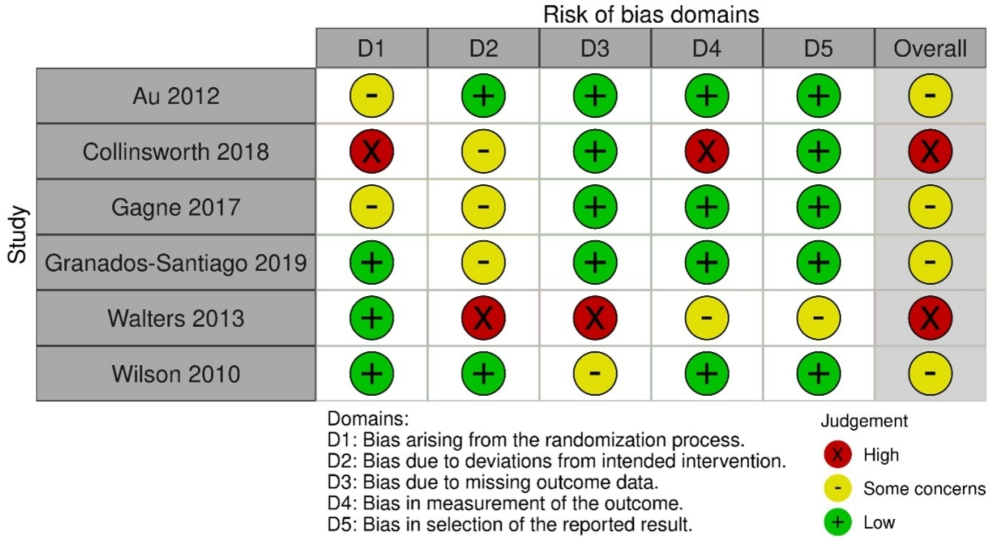

Risk of bias in RCT studies

116x72mm (300 x 300 DPI)

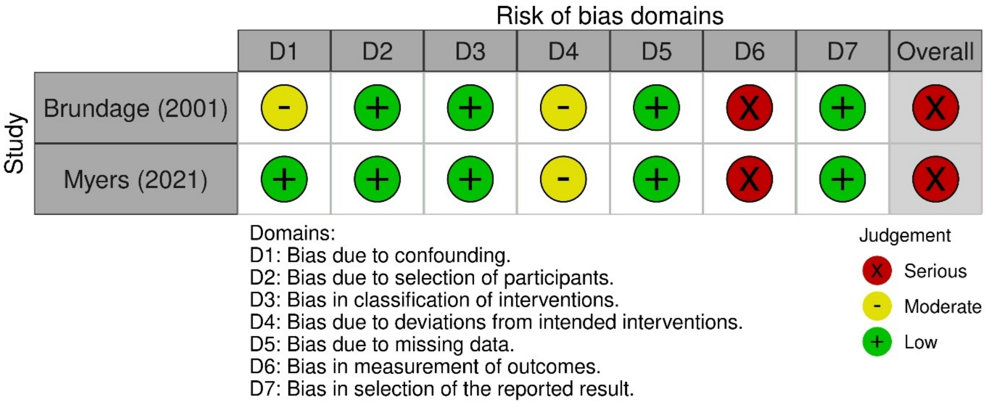

Risk of bias in non-RCT studies

116x57mm (300 x 300 DPI)

Supplementary Material

Title:

A systematic review of shared decision-making interventions for people living with chronic respiratory diseases

Authors:

Barradell, AC; Gerlis, C; Houchen-Wolloff L; Bekker, HL; Robertson, N; Singh, SJ.

For peer review only

For peer review only - http://bmjopen.bmj.com/site/about/guidelines.xhtml

Supplementary material for Methods:

MEDLINE search strategy

1. ((joint* or share* or sharing* or informed*) adj3 (decision* or decid*3 or choice*)).mp. [mp=title, abstract, original title, name of substance word, subject heading word, floating sub-heading word, keyword heading word, organism supplementary concept word, protocol supplementary concept word, rare disease supplementary concept word, unique identifier, synonyms]
2. "DECISION MAKING, SHARED"/
3. exp "DECISION MAKING"/
4. "DECISION SUPPORT TECHNIQUES"/
5. DECISION SUPPORT SYSTEMS, CLINICAL/
6. ((decision* or choice*) and (making* or support* or behavior* or behaviour*)).ti,ab.
7. PATIENT PARTICIPATION/
8. ((patient* or consumer*) adj3 (involvement* or involving* or participation* or participating*)).ti,ab.
9. "PATIENT-CENTERED CARE"/
10. "PROFESSIONAL-PATIENT RELATIONS"/
11. "PHYSICIAN-PATIENT RELATIONS"/
12. ((patient* or person* or client* or consumer*) adj3 (centred or centered or focused or oriented)).ti,ab.
13. "decision tool*".ti,ab.
14. "clinical counsel*".ti,ab.
15. "patient decision guide*".ti,ab.
16. "decision coaching".ti,ab.
17. "decision aid*".ti,ab.
18. or/1-17
19. LUNG DISEASES, OBSTRUCTIVE/
20. exp PULMONARY DISEASE, CHRONIC OBSTRUCTIVE/
21. emphysema*.ti,ab.
22. (chronic* adj3 bronchiti*).ti,ab.
23. (obstruct* adj3 (pulmonary or lung* or airway* or airflow* or bronch* or respirat*)).ti,ab.
24. (coad or cobd or copd or aecb).ti,ab.
25. asthma*.ti,ab.
26. exp ASTHMA/
27. (idiopathic and pulmonary and fibrosis).ti,ab.
28. IDIOPATHIC PULMONARY FIBROSIS/
29. (interstitial and lung and disease).ti,ab.
30. LUNG DISEASES, INTERSTITIAL/
31. (extrinsic and allergic and alveolitis).ti,ab.
32. ALVEOLITIS, EXTRINSIC ALLERGIC/
33. (non and specific and interstitial and (pneumonitis or NSIP)).ti,ab.

For peer review only - http://bmjopen.bmj.com/site/about/guidelines.xhtml

34. (Usual and interstitial and (pneumonia or UIP)).ti,ab.

35. "LUNG DISEASES, OBSTRUCTIVE"/

36. ((lung or pulmonary or airway) adj disease).ti,ab.

37. "cystic fibrosis".ti,ab.

38. CYSTIC FIBROSIS/

39. "SARCOIDOSIS, PULMONARY"/

40. sarcoidosis.ti,ab.

41. exp "LUNG NEOPLASMS"/

42. (lung adj (cancer* or tumo?r* or neoplas*)).ti,ab.

43. "TUBERCULOSIS, PULMONARY"/

44. tuberculosis.ti,ab.

45. BRONCHIECTASIS/

46. bronchiectasis.ti,ab.

47. exp PNEUMOCONIOSIS/

48. (autoimmune adj3 (lung or airway or pulmonary)).ti,ab.

49. or/19-48

50. randomized controlled trial.pt.

51. controlled clinical trial.pt.

52. randomized.ab.

53. placebo.ab.

54. clinical trials as topic.sh.

55. randomly.ab.

56. trial.ti.

57. or/50-56

58. exp ANIMALS/ not humans.sh.

59. 57 not 58

60. 59 and 18 and 49

Data items

Extracted items included: population (age, sex, health literacy skill, diagnosis, disease severity, length of diagnosis, baseline measures, total number randomised), intervention (group names, group sample size, adherence to SDM principles, adherence to complex intervention development guidelines, content, training, timing, frequency, duration and any other interventions), comparator (group names, group sample size, content, training, timing, frequency, duration) and outcomes (type, unit of analysis, reported missing data). Data was also extracted on each study's objectives, study design, unit of allocation, decision posed (e.g. medication, pulmonary rehabilitation, surgery, end of life), duration of the study and follow up.

Sub-group analysis

The following additional data was collected for sub-group analysis: diagnosis, characteristics (age, sex, health literacy skill), decision posed (medication, pulmonary rehabilitation, surgery, end of life), intervention dose (number and length of sessions) and length of follow-up.

For peer review only - http://bmjopen.bmj.com/site/about/guidelines.xhtml

For peer review only

Supplementary Material for Results:

Table 1: Summary of studies (extended version)

First Author, Year, Country, Sample size, Study design, Population	Outcome(s)	Summary of results	Certainty of evidence (GRADE score)
Au, 2012, USA, Clinicians = 92 Patients = 376, RCT, Outpatients with COPD	*Validated:* Quality of communication (QOC). 19-items scored from 0-100 (higher score=better communication). *Non-validated:* Occurrence of discussions about end of life preferences between patients and either clinician or surrogate. 4-item self-reported questionnaire scored from 0-100% (higher%=greater occurrence).	Baseline quality of communication was poor in both groups. Modest improvements in both groups, but significant mean between group difference favouring the intervention group (5.7 points; $p<0.05$). The occurrence (%) of patient communication about end of life care was significantly higher in the intervention group for three of four questions (mean(SD)): 1. Int: 35.2(74.6) Con: 15.9(58.2) $p<0.05$, 2. Int: 60.3(122.9) Con: 30.8(175.5) $p<0.05$, 3. Int: 53.6(115.1) Con: 45.2(73.3) $p>0.05$, 4. Int: 86.2(84.6) Con: 75.2(115.0) $p<0.05$.	Moderate ⊕⊕⊕◯ Moderate certainty because of risk of bias.
Brundage, 2001, Canada, Patients = 20, Feasibility CBA, Outpatients with lung cancer	*Validated:* Decisional conflict (DCS). 16-items using a 4-point Likert scale anchored with strongly agree and strongly disagree. Converted scale scores >37.5 indicate meaningful decisional conflict or delayed decision implementation. *Non-validated:* Knowledge of treatment options. Three open-ended questions. Strength of treatment preference. 1 item using a 7-point Likert scale anchored with definitely wish treatment 1 and definitely wish treatment 2.	Decisional conflict reduced after receiving the intervention in 87% and increased in 13% of participants. Knowledge of the available treatment options improved following the intervention for both 3-year outcome survival[a] and median survival outcome[b]: 1. Knowledge of survival outcomes with treatment 1 alone (mean between group difference 60%[a]; 54%[b]), 2. Knowledge of direction of survival difference between treatments (mean between group difference: 27%[a]; 40%[b]), 3. Knowledge of the magnitude of survival difference between treatments (mean between group difference: 73%[a]; 67%[b]). The number of participants with a treatment preference increased from 80% to 100% following the intervention.	Low ⊕⊕◯◯ Low certainty because of risk of bias and minor publication bias.
Collinsworth, 2018, USA, Patients = 308, RCT, Inpatients with COPD	*Validated:* Patient activation (PAM). 22-items using a 4-point Likert scale anchored with patient is disengaged and overwhelmed and patient has adopted and is maintaining healthy behaviours. Quality of life health status (CAT). 8 item scale with scores ranging from 0-40. Lower scores (<10) indicate COPD causes few problems and higher scores (>30)	Both groups had a significant improvement in PAM scores following the intervention (mean(SD) difference: 0.52(0.9) and 0.69(1.0) points respectively, $p<0.05$). Only the intervention group had significantly increased scores in the CAT following the intervention (mean(SD) difference: Int: 5.27(10.3), $p<0.05$; Con: 0.38(7.8), $p>0.05$). There were no significant differences in the total number of all-cause or COPD-related readmissions between groups at 1, 2, 3, 6 and 9 months ($p>0.05$).	Moderate ⊕⊕⊕◯ Moderate certainty because of risk of bias.

For peer review only - http://bmjopen.bmj.com/site/about/guidelines.xhtml

	indicate COPD prevents patients from doing most things they want to do. *Non-validated:* All-cause hospital admissions extracted from electronic health records. Reported as frequency count.		
Gagne, 2017, Canada, Patients = 51, RCT, Outpatients with asthma	*Validated:* Asthma knowledge (QCALF). 37-items with scores ranging from -37 to +37 (higher scores indicate greater knowledge). Decisional conflict (DCS; see above). *Non-validated*: Appropriate use of asthma pharmacotherapy. 4-item face-to-face interviewer-administered questionnaire. The cut off for appropriate use was meeting eleven hierarchical criteria.	Both groups had significantly increased knowledge scores following the intervention (mean(SD) difference: Int: 3.6(8.9)*, p<0.001; Con: 2(7.3)*, p<0.05). There was no significant between group difference (p>0.05). Both groups had significantly reduced decisional conflict scores following the intervention (mean(SD) difference: Int: 8.1(22.5)*, p<0.05; Con: 9.1(27.7)*, p>0.05). There was no significant between group difference (p>0.05). Both groups had modest, but non-significant, within group improvements in their appropriate use of pharmacotherapy (mean(SD) difference: Int: 0.15(0.7)*, p>0.05; Con: 0.16(0.7)*, p>0.05). There was no significant between group difference (mean(SD) between group difference = 0.17(0.7), p>0.05).	Moderate ⊕⊕⊕◯ Moderate certainty because of risk of bias.
Granados-Santiago, 2019, Spain, Patients = 42, RCT, Inpatients with COPD	*Validated:* Quality of life and health status (EQ-5D). 5-items with scores ranging from 0 (defined as the worst imaginable health state) to 100 (defined as the best imaginable health state). COPD knowledge (COPD-Q). 13-items with scores ranging from 0 (defined as lowest knowledge) to 13 (defined as highest knowledge). Medication adherence (TAI).10-items, with higher scores indicating greater adherence. Physical activity (steps/day) measured using the Fitbit Flex 2 Pedometer. Nutritional status (MNA). 18-items with scores lower than 17 considered malnutrition.<br>*Non-validated:*<br>-	Both groups had significant and clinically important improvements in their quality of life and health status at discharge (mean(SD) between group difference: 6.15(27.5)*, p<0.05) but only the intervention group maintained this benefit 3 months later (mean(SD) between group difference: 8.28(50.6)*, p<0.05). Knowledge scores significantly improved in the intervention group alone at discharge (mean(SD) between group difference: 3.89(0.7)*, p<0.05) and maintained at 3 months follow up (mean(SD) between group difference: 3.88(0.8)*, p<0.05). Adherence significantly improved at discharge in the intervention group alone (mean(SD) difference: Int: 3.58(1.5)*, p<0.05; Con: 1.36(1.6)*, p>0.05) but there was no significant between group difference. At 3 months post intervention there was a significant mean(SD) between group difference favouring the intervention group (1.8(2.4)*, p<0.05). Upon discharge, there was a significant reduction in physical activity in both groups (mean(SD) difference: Int: -348.27(2.0)*, p<0.05; Con: -410.05(4.66)*, p<0.05). At 3 months post intervention there was a significant improvement in the intervention group alone (mean(SD) difference: Int: 1371.97(895.2)*, p<0.05; Con: 620.18(12.5)*, p>0.05). At 3 months post intervention there was a significant improvement in nutritional status both within and between groups for the intervention group alone (mean(SD) difference: Int: 4.15(0.1)*, p<0.05; Con: -3.43(0.12)*, p>0.05	Moderate ⊕⊕⊕◯ Moderate certainty because of risk of bias.
Myers, 2021, USA, Patients = 5, Pilot CBA,	*Validated:* Decisional Conflict (DCS SURE Test). 4-item scale with scores from 0-4, a score <4 indicates clinically significant decisional conflict. *Non-validated:*	Decisional conflict reduced post intervention (mean difference=1). Awareness of treatment options increased from 40% to all 100% participants post intervention. At 30 days post intervention, 80% of participants had made a decision. 40% of participants had a treatment that matched their treatment preference.	Low ⊕⊕◯◯

For peer review only - http://bmjopen.bmj.com/site/about/guidelines.xhtml

For peer review only

Outpatients with non-small cell lung cancer	Knowledge of treatment options. 3-item survey using a 3-point Likert scale (Yes/No/Unsure) to elicit patient beliefs regarding curability of treatment options, awareness of treatment options, and treatment costs. Awareness of treatment options only item included in this analysis as only item measuring knowledge. Treatment status. 30-day treatment status extracted from electronic health records. This was compared to treatment preferences immediately post intervention.		Low certainty because of risk of bias.
Walters, 2013, Australia, Patients = 182, Cluster RCT, Outpatients with COPD	*Validated:* Quality of life (SF-36). 8-items with scores from 0-100, higher score indicates better quality of life. Quality of life (SGRQ). 76-items with scores ranging from 0-100, higher score indicates worse quality of life. Patient involvement in healthcare decisions (PIH). 12-items with scores ranging from 0-8 (higher score indicates more involvement). *Non-validated:* Respiratory hospital admissions extracted from electronic health records. Reported as frequency count. Intervention fidelity. Recorded telephone calls from the first three intervention and 10% of control contacts were coded by two raters for content and fidelity to prespecified components of the intervention to assess adherence. To ensure concordance, both raters coded a random sample of 20 calls.	Neither group had a significant change to either their physical[a] or mental[b] health status at 6 months (mean(SD) between group difference: 1.5(0.2)*[a]; 0.8(1.4)*[b], p>0.05) and 12 months (mean(SD) between group difference: 0.0(0.9)*[a]; 0.3(0.9)*[b], p>0.05) post intervention. Neither group had a significant change to their overall quality of life at 6 months (mean(SD) between group difference: 1.9(2.7)*, p>0.05) and 12 months (mean(SD) between group difference: 1.4(1.5)*, p>0.05 post intervention. Only the intervention group had a significant improvement in PIH scores at 6 months (mean(SD) between group difference: 0.4(0.2)*, p<0.05) and 12 months (mean(SD) between group difference: 0.3(0.1)*, p<0.05) post intervention. No significant change in the number of hospital admissions for either group over the 12 months study period (χ^2=2.61, p>0.05). Concordance between two raters was high (overall weighted κ 0.72). Fidelity assessments confirmed specific health mentoring components were addressed with some clarity: 1. COPD symptom management, 49%, 2. Unhelpful self-talk explored and identified, 37%, 3. Unhelpful self-talk challenged, and new self-talk developed, 35%, 4. Action plan for achieving goals made, 54%, 5. Problems and barriers to achieving goals identified and clarified, 46%, 6. Positive changes in behaviour praised, 83%. Raters concluded there was a lower delivery of cognitive behavioural components of intervention.	Very low ⊕◯◯◯ Very low certainty because of significant risk of bias and some inconsistency.
Wilson, 2010, Hawaii, Patients = 612, RCT,	*Validated:* Quality of life (MiniAQLQ). 5-items with Likert scale ranging from 0 (all of the time) to 7 (none of the time). *Non-validated:* Healthcare use extracted from electronic health records. Reported as frequency count.	Only intervention groups had a significant improvement in quality of life scores at 12 months post intervention (SDM to control mean(SD) between group difference = 0.39(1.0)*, p<0.05; SDM–clinician decision-making mean(SD) between group difference = 0.11(1.1)*, p>0.05; clinician decision-making-control mean(SD) between group difference = 0.28(1.0)*, p<0.05. Only intervention groups had a significantly reduced	Moderate ⊕⊕⊕◯ Moderate certainty

For peer review only

For peer review only - http://bmjopen.bmj.com/site/about/guidelines.xhtml

Outpatients with asthma	Patients' perceived role in treatment decision. 1-item using a 5-point Likert scale ranging from 0 (low involvement in decision-making) to 5 (high involvement in treatment decision-making). Medication adherence. Data extracted from pharmacy records to calculate a continuous medication acquisition index for each year. Intervention fidelity. Audiotapes of both intervention groups of 10% of patients were scored on a detailed performance checklist to determine whether the two protocols were delivered as intended. Scores ranged from 0-4 with higher scores indicated greater adherence.	healthcare utilisation (SDM-control mean(SD) between group difference =20.36(-1.5)*, p<0.05; SDM-clinician decision-making mean(SD) between group difference =0.01(2.7)*, p>0.05; clinician decision-making-control mean(SD) between group difference= 20.37(-1.5*), p<0.05. The SDM group rated their influence on the treatment decision as being approximately the same as the clinicians' influence (mean(SD)= 3.1(0.6). The SDM group ratings were significantly different from the clinician decision-making group, with the latter feeling that their clinician had a greater influence (mean(SD)=2.5(0.9), p<0.05) than they did themselves. Medication adherence significantly increased only in intervention groups (SDM-control mean(SD) between group difference=0.21(0.5)*, p<0.05; SDM–clinician decision-making mean(SD) between group difference= 0.08(0.4)*, p>0.05; CDM-control mean(SD) between group difference= 0.13(0.4)*, p<0.05. This was not sustained at 2 years (SDM-control mean(SD) between group difference= 0.03(0.4)*, p>0.05; SDM-clinician decision-making mean(SD) between group difference= 0.04(0.4)*, p>0.05; clinician decision-making-control mean(SD) between group difference=-0.01(-0.4)*, p>0.05. Protocol adherence scores assigned by the raters were high (SDM=4.0, clinician decision-making=3.9) and did not differ significantly between the two groups (p>0.05).	because of risk of bias.

For peer review only - http://bmjopen.bmj.com/site/about/guidelines.xhtml

For peer review only

Table 2: Characteristics of excluded studies

Lead Author	Year	Reason for exclusion	Categorised reason for exclusion
Aaron[1]	2006	Abstract only (results in Vandemheen 2009)	Type of publication
Aboumatar[2]	2017	No results in paper (in Aboumatar 2019)	Type of publication
Aboumatar[3]	2019	No SDM within intervention	Type of intervention
Braun[4]	2005	Abstract only (in Menon 2016)	Type of publication
Brown [5]	2012	CRD participant data not distinguishable from analysis	Participant population
Brown [6]	2004	CRD participant data not distinguishable from analysis	Participant population
Brundage[7]	1998	CRD participant data not distinguishable from analysis	Participant population
Butow[8]	2015	No CRD participants	Participant population
Carlucci [9]	2016	No comparator group	Study design
Cook[10]	2016	No adult CRD participants	Participant population
Coultas[11]	2005	No SDM within intervention	Type of intervention
Cvietusa[12]	2020	No SDM within intervention	Type of intervention
Daly[13]	2013	CRD participant data not distinguishable from analysis	Participant population
Detmar[14]	2002	CRD participant data not distinguishable from analysis	Participant population
Dyar[15]	2012	CRD participant data not distinguishable from analysis	Participant population
Early[16]	2015	No SDM within intervention	Type of intervention
Fiks[17]	2015	No CRD participants	Participant population
Fors[18]	2018	CRD participant data not distinguishable from analysis	Participant population
Foster[19]	2016	No SDM within intervention	Type of intervention
Foster[20]	2014	No more than one choice	Type of intervention
Fraenkel[21]	2016	No CRD participants	Participant population
Gagne[22]	2016	Abstract only (results in Gagne 2017)	Type of publication

Supplementary material V1.0

George[23]	2017	Abstract only - (results in George 2020)	Type of publication
George[24]	2020	No more than one choice	Type of intervention
Gupta[25]	2019	No SDM within intervention	Type of intervention
Hagan[26]	2008	No SDM within intervention	Type of intervention
Halley[27]	2015	No CRD participants	Participant population
Hilberink[28]	2011	No SDM within intervention	Type of intervention
Hoerger[29]	2017	CRD participant data not distinguishable from analysis	Participant population
Hollen[30]	2013	No comparator group	Study design
Hoskins[31]	2016	No SDM within intervention	Type of intervention
Houben[32]	2019	No SDM within intervention	Type of intervention
Hyatt[33]	2018	CRD participant data not distinguishable from analysis	Participant population
Izumi[34]	2018	CRD participant data not distinguishable from analysis	Participant population
Jonsdottir[35]	2015	No SDM within intervention	Type of intervention
Kass[36]	2009	CRD participant data not distinguishable from analysis	Participant population
Kennedy[37]	2013	CRD participant data not distinguishable from analysis	Participant population
Koerner[38]	2014	No CRD participants	Participant population
Krug[39]	2021	No SDM within intervention	Type of intervention
Lainscak[40]	2013	No SDM within intervention	Type of intervention
Latimer[41]	2007	CRD participant data not distinguishable from analysis	Participant population
Lavoie[42]	2014	No SDM within intervention	Type of intervention
Leung[43]	2012	Cross-sectional study	Study design
Lim[44]	2021	Hypothetical decision making	Type of intervention
Llanos-Paez[45]	2021	No SDM within intervention	Type of intervention
Lopez-Lopez[46]	2020	No SDM within intervention	Type of intervention

For peer review only - http://bmjopen.bmj.com/site/about/guidelines.xhtml

Ludden[47]	2018	Adult CRD participant data not distinguishable from analysis	Participant population
Makimoto[48]	2020	Letter to editor	Type of publication
Markman[49]	2005	No comparator group	Study design
Markun[50]	2018	No participant-facing content	Type of intervention
Mayer[51]	2016	CRD participant data not distinguishable from analysis	Participant population
Meline[52]	2021	No CRD participants	Participant population
Melzer[53]	2018	No SDM within intervention	Type of intervention
Menon[54]	2016	CRD participant data not distinguishable from analysis	Participant population
Meropol[55]	2013	CRD participant data not distinguishable from analysis	Participant population
Moullec[56]	2012	No SDM within intervention	Type of intervention
Murray[57]	2010	CRD participant data not distinguishable from analysis	Participant population
Nedjat-Haiem[58]	2017	CRD participant data not distinguishable from analysis	Participant population
Nugent[59]	2018	No comparator group	Study design
Patel[60]	2019	No participant-facing content	Type of intervention
Rabow[61]	2004	CRD participant data not distinguishable from analysis	Participant population
Rea[62]	2004	No SDM within intervention	Type of intervention
Reddel[63]	2019	Abstract only (results not yet published)	Type of publication
Reinke[64]	2017	No SDM outcomes	Outcome measures
Rhee[65]	2008	Participants <18years old	Participant population
Roelands[66]	2004	No CRD participants	Participant population
Rosemann[67]	2013	Abstract only (results in Steurer 2017)	Type of publication
Ruparel[68]	2019	No CRD participants	Participant population
Rutten-vanMolken[69]	2017	Abstract only - (results in Slok 2016)	Type of publication
Sands[70]	2014	Abstract only - (results not yet published)	Type of publication

Supplementary material V1.0

Schofield[71]	2013	No SDM within intervention	Type of intervention
Schubart[72]	2015	CRD participant data not distinguishable from analysis	Participant population
Schumacher[73]	2002	No quantitative data	Study design
Schwarze[74]	2020	CRD participant data not distinguishable from analysis	Participant population
Shade[75]	2019	Adult CRD participant data not distinguishable from analysis	Participant population
Shirai[76]	2012	CRD participant data not distinguishable from analysis	Participant population
Sinclair[77]	2017	No SDM within intervention	Type of intervention
Skorstengaard[78]	2019	CRD participant data not distinguishable from analysis	Participant population
Slok[79]	2016a	Abstract only (results in Slok 2016b)	Type of publication
Slok[80]	2016b	No SDM within intervention	Type of intervention
Smidth[81]	2013	No comparator group	Study design
Steinhauser[82]	2008	CRD participant data not distinguishable from analysis	Participant population
Steurer[83]	2017	Abstract only (results in Steurer 2015)	Type of publication
Steurer-Stey[84]	2015	No SDM within intervention	Type of intervention
Sullivan[85]	2020	Abstract only	Type of publication
Tai-Seale[86]	2016	CRD participant data not distinguishable from analysis	Participant population
Tamblyn[87]	2005	Abstract only (results in Tamblyn 2015)	Type of publication
Tamblyn[88]	2015	No SDM within intervention	Type of intervention
Tang[89]	2008	No comparator group	Study design
Tapp[90]	2017	No SDM outcomes	Outcome measures
Thee[91]	2021	Protocol paper	Type of publication
Thiam[92]	2007	No SDM within intervention	Type of intervention
Totten[93]	2018	Abstract only (results not yet published)	Type of publication
Tsao[94]	2021	No SDM within intervention	Type of intervention

For peer review only - http://bmjopen.bmj.com/site/about/guidelines.xhtml

Vandemheen[95]	2009	No SDM within intervention	Type of intervention
Vandemheen[95]	2009	Supplement (to be read with Vandemheen 2009)	Type of publication
Wahlberg[96]	2016	CRD participant data not distinguishable from analysis	Participant population
Waller[97]	2012	CRD participant data not distinguishable from analysis	Participant population
Wang[98]	2010	No SDM within intervention	Type of intervention
Weekes[99]	2009	No SDM within intervention	Type of intervention
Williams[100]	2013	CRD participant data not distinguishable from analysis	Participant population
Wilson[101]	2009	Abstract only (results in Wilson 2010)	Type of publication
Wilson[102]	2005	Abstract only (results in Wilson 2010)	Type of publication
Wilson[103]	2005	Abstract only (results in Wilson 2010)	Type of publication
Wood-Baker[104]	2012	No SDM within intervention	Type of intervention
Yoo[105]	2018	No CRD participants	Participant population
Zakrisson[106]	2019	CRD participant data not distinguishable from analysis	Participant population
Zanartu[107]	2017	Letter to editor	Type of publication

For peer review only - http://bmjopen.bmj.com/site/about/guidelines.xhtml

Sub-group analysis

Despite the broad inclusion criteria for respiratory conditions, studies only included those with COPD, lung cancer and asthma (Table 2). Participants diagnosed with lung cancer and COPD were older than those diagnosed with asthma. The split between male and female participants were even across studies apart from in one study where nearly all were male[108] and another where three quarters were female[109]. No specific health literacy outcome measures were used across studies. Baseline educational attainment was reported across all but two studies[110,111]. Across other studies, the percentage of participants who completed higher education varied between 17-59.2%.

SDM interventions for COPD participants aimed to facilitate decisions regarding end-of-life care and positive self-management. Those for lung cancer and asthma participants aimed to facilitate treatment decision-making. Four studies reported a single session SDM intervention[108,109,112,113]. Three studies reported multiple sessions of SDM delivered face to face and with follow up telephone calls[110,114,115]. One study did not report an intervention dose and instead stated the intervention was integrated into routine inpatient care[111]. The length of study follow-up varied from immediately post decision-making to 12 months later.

For peer review only

For peer review only - http://bmjopen.bmj.com/site/about/guidelines.xhtml

Table 2: Sub-group analysis of CRD patients from included studies

First Author (Year)	CRD diagnosis	Age (mean (SD))	Gender (% male)	Measure of health literacy (% achievement)	Decision posed	Intervention dose	Length of follow up
Au (2012)	COPD	IG: 69.4(10.0) years	IG: 97.9%	IG:<br>Higher education = 58.3%	IG: End of life care	IG: Single session	IG: 2 weeks
		CG: 69.4(10.0) years	CG: 96.2%	CG:<br>Higher education = 59.2%	CG: End of life care	CG: N/A	CG: 2 weeks
Brundage (2001)	Lung cancer	IG: 69.2(8.1) years	IG: 55%	IG:<br><High school = 5%<br>High school education = 50%<br>Higher education = 45%	IG: Lung cancer treatments	IG: Single session	IG: Immediately post intervention
Collinsworth (2018)	COPD	IG: 70.0(11.9)	IG: 39.7%	IG: Not disclosed	IG: Self-management plans	IG: Single session and 4 telephone calls	IG: 6 months
		CG: 70.9(12.5)	CG: 43.1%	CG: Not disclosed	CG: Self-management plans	CG: N/A	CG: 6months
Gagne (2017)	Asthma	IG: 46(13.0)	IG: 26.9%	IG:<br>High school education = 57.7%<br>Higher education = 42.3%	IG: Asthma treatments	IG: Single session	IG: 2months
		CG: 41(13.0)	CG: 48%	CG:<br>High school education = 52.0%<br>Higher education = 48.0%	CG: Asthma treatments	CG: N/A	CG: 2months
Granados-Santiago (2019)	COPD	IG: 69.3(9.9)	IG: Not disclosed	IG: Not disclosed	IG: Self-management plans	IG: Integrated into inpatient care	IG: 3 months
		CG: 74.20(9.25)	CG: Not disclosed	CG: Not disclosed	CG: Self-management plans	CG: N/A	CG: 3 months
Myers (2021)	Lung cancer	IG: Not reported	IG: 40%	IG:<br><High school education = 0%<br>High school education = 40%	IG: Lung cancer treatments	IG: Single session	IG: 30 days

For peer review only

For peer review only - http://bmjopen.bmj.com/site/about/guidelines.xhtml

				Higher education = 60%			
Walters (2013)	COPD	IG: 68.2(7.9)	IG: 54.4%	IG: <High school education = 68% High school education = 13% Higher education = 20%	IG: Self-management plans	IG: Single session plus multiple (x16) telephone calls	IG: 12 months
		CG: 67.3(7.6)	CG: 51.1%	CG: <High school education = 75% High school education = 8% Higher education = 17%	CG: Self-management plans	CG: Single session plus multiple (x12) telephone calls	CG: 12 months
Wilson (2010)	Asthma	IG: 45.7(13.3)	IG: 43.6%	IG: <High school education = 2.9% High school education = 55.9% Higher education = 41.1%	IG: Asthma treatments	IG: Multiple sessions (x2) plus multiple telephone calls (x3)	IG: 12 months
		IG2: 46.9(12.1)	IG2: 44.1%	IG2: <High school education = 0.98% High school education = 64.7% Higher education = 33.8%	IG2: Asthma treatments	IG2: Multiple sessions (x2) plus multiple telephone calls (x3)	IG2: 9 months
		CG: 45.1(12.4)	CG: 42.6%	CG: <High school education= 2.9% High school education = 56.9% Higher education = 40.2%	CG: Asthma treatments	CG: Multiple sessions (x2) plus multiple telephone calls (x3)	CG: 9 months

IG = Intervention group; CG = Control group; IG2 = Intervention group 2 (Clinician Decision-Making)

For peer review only - http://bmjopen.bmj.com/site/about/guidelines.xhtml

For peer review only

References

E1. Aaron SD. Evaluation of a Decision Aid for Adult Cystic Fibrosis Patients Considering Bilateral Lung Transplantation. *Clin Regist Clin trials*. 2006.

2. Aboumatar H, Naqibuddin M, Chung S, et al. Better Respiratory Education and Treatment Help Empower (BREATHE) study: Methodology and baseline characteristics of a randomized controlled trial testing a transitional care program to improve patient-centered care delivery among chronic obstructive pulm. *Contemp Clin Trials*. 2017;62(June):159-167. doi:10.1016/j.cct.2017.08.018

3. Aboumatar H, Naqibuddin M, Chung S, et al. Effect of a Hospital-Initiated Program Combining Transitional Care and Long-term Self-management Support on Outcomes of Patients Hospitalized with Chronic Obstructive Pulmonary Disease: A Randomized Clinical Trial. *JAMA - J Am Med Assoc*. 2019;322(14):1371-1380. doi:10.1001/jama.2019.11982

4. Braun U. A Culturally Sensitive Values-Guided Aid for End of Life Decision-Making. *Clin Regist Clin Trials*. 2005. doi:10.1002/CENTRAL/CN-01510546

5. Brown RF, Bylund CL, Li Y, Edgerson S, Butow P. Testing the utility of a cancer clinical trial specific Question Prompt List (QPL-CT) during oncology consultations. *Patient Educ Couns*. 2012;88(2):311-317. doi:10.1016/j.pec.2012.02.009

6. Brown RF, Butow PN, Sharrock MA, et al. Education and role modelling for clinical decisions with female cancer patients. *Heal Expect*. 2004;7(4):303-316. doi:10.1111/j.1369-7625.2004.00294.x

7. Brundage MD, Davidson JR, Mackillop WJ, Feldman-Stewart DEB, Groome P. Using a treatment-tradeoff method to elicit preferences for the treatment of locally advanced non-small-cell lung cancer. *Med Decis Mak*. 1998;18(3):256-267. doi:10.1177/0272989X9801800302

8. Butow P, Brown R, Aldridge J, et al. Can consultation skills training change doctors' behaviour to increase involvement of patients in making decisions about standard treatment and clinical trials: A randomized controlled trial. *Heal Expect*. 2015;18(6):2570-2583. doi:10.1111/hex.12229

9. Carlucci A, Vitacca M, Malovini A, et al. End-of-Life Discussion, Patient Understanding and Determinants of Preferences in Very Severe COPD Patients: A Multicentric Study. *COPD J Chronic Obstr Pulm Dis*. 2016;13(5):632-638. doi:10.3109/15412555.2016.1154034

For peer review only - http://bmjopen.bmj.com/site/about/guidelines.xhtml

10. Cook KA, Modena BD, Simon RA. Improvement in Asthma Control Using a Minimally Burdensome and Proactive Smartphone Application. *J Allergy Clin Immunol Pract*. 2016;4(4):730-737.e1. doi:10.1016/j.jaip.2016.03.005

11. Coultas D, Frederick J, Barnett B, Singh G, Wludyka P. A randomized trial of two types of nurse-assisted home care for patients with COPD. *Chest*. 2005;128(4):2017-2024. doi:10.1378/chest.128.4.2017

12. Cvietusa PJ, Wagner NM, Shoup JA, et al. Digital Communication Technology: Does Offering a Choice of Modality Improve Medication Adherence and Outcomes in a Persistent Asthma Population? *Perm J*. 2020;25:1. doi:10.7812/TPP/20.189

13. Daly BJ, Douglas SL, Gunzler D, Lipson AR. Clinical trial of a supportive care team for patients with advanced cancer. *J Pain Symptom Manage*. 2013;46(6):775-784. doi:10.1016/j.jpainsymman.2012.12.008

14. Detmar SB, Muller MJ, Schornagel JH, Wever LDV, Aaronson NK. Health-related quality-of-life assessments and patient-physician communication: A randomized controlled trial. *J Am Med Assoc*. 2002;288(23):3027-3034. doi:10.1001/jama.288.23.3027

15. Dyar S, Lesperance M, Shannon R, Sloan J, Colon-Otero G. A nurse practitioner directed intervention improves the quality of life of patients with metastatic cancer: Results of a randomized pilot study. *J Palliat Med*. 2012;15(8):890-895. doi:10.1089/jpm.2012.0014

16. Early F, Everden AJT, OBrien CM, Fagan PL, Fuld JP. Patient agenda setting in respiratory outpatients. *Chron Respir Dis*. 2015;12(4):347-356. doi:10.1177/1479972315598696

17. Fiks AG, Mayne SL, Karavite DJ, et al. Parent-reported outcomes of a shared decision-making portal in asthma: A practice-based RCT. *Pediatrics*. 2015;135(4):e965-e973. doi:10.1542/peds.2014-3167

18. Fors A, Blanck E, Ali L, et al. Effects of a person-centred telephone-support in patients with chronic obstructive pulmonary disease and/or chronic heart failure – A randomized controlled trial. *PLoS One*. 2018;13(8). doi:10.1371/journal.pone.0203031

19. Foster JM, Smith L, Usherwood T, Sawyer SM, Reddel HK. General practitioner-delivered adherence counseling in asthma: Feasibility and usefulness of skills, training and support tools. *J Asthma*. 2016;53(3):311-320. doi:10.3109/02770903.2015.1091473

20. Foster JM, Usherwood T, Smith L, et al. Inhaler reminders improve adherence with controller treatment in primary care patients with asthma. *J Allergy Clin Immunol*. 2014;134(6):1260-

For peer review only - http://bmjopen.bmj.com/site/about/guidelines.xhtml

1268.e3. doi:10.1016/j.jaci.2014.05.041

21. Fraenkel L, Peters E, Tyra S, Oelberg D. Shared Medical Decision Making in Lung Cancer Screening: Experienced versus Descriptive Risk Formats. *Med Decis Mak*. 2016;36(4):518-525. doi:10.1177/0272989X15611083

22. Gagné M, Légaré F, Moisan J, Boulet L. Adding A Decision Aid to Asthma Education: Impact on Decisional Conflict and Appropriate Medication Usage. *Value Heal*. 2016;19(7):A557. doi:10.1016/j.jval.2016.09.1216

23. George M. BREATHE asthma intervention trial. *Clin Regist Clin Trials*. 2017. doi:10.1002/CENTRAL/CN-01380211

24. George M, Bruzzese JM, S. Sommers M, et al. Group-randomized trial of tailored brief shared decision-making to improve asthma control in urban black adults. *J Adv Nurs*. 2020;(September):1-17. doi:10.1111/jan.14646

25. Gupta S, Price C, Agarwal G, et al. *The Electronic Asthma Management System (EAMS) Improves Primary Care Asthma Management*. Vol 53.; 2019. doi:10.1183/13993003.02241-2018

26. Hagan L, Valois P, Patenaude H, Boutin H, Boulet LP, Lafrenière F. Asthma counselling targeted to removal of domestic animals. *Can Respir J*. 2008;15(1):33-38. doi:10.1155/2008/247027

27. Halley MC, Rendle KAS, Gillespie KA, Stanley KM, Frosch DL. An exploratory mixed-methods crossover study comparing DVD- vs. Web-based patient decision support in three conditions: The importance of patient perspectives. *Heal Expect*. 2015;18(6):2880-2891. doi:10.1111/hex.12273

28. Hilberink SR, Jacobs JE, Breteler MHM, de Vries H, Grol RPTM. General practice counseling for patients with chronic obstructive pulmonary disease to quit smoking: Impact after 1year of two complex interventions. *Patient Educ Couns*. 2011;83(1):120-124. doi:10.1016/j.pec.2010.04.009

29. Hoerger M, Perry LM, Gramling R, Epstein RM, Duberstein PR. Does educating patients about the early palliative care study increase preferences for outpatient palliative cancer care? Findings from project EMPOWER. *Heal Psychol*. 2017;36(6):538-548. doi:10.1037/hea0000489

30. Hollen PJ, Gralla RJ, Jones RA, et al. A theory-based decision aid for patients with cancer:

For peer review only - http://bmjopen.bmj.com/site/about/guidelines.xhtml

Results of feasibility and acceptability testing of DecisionKEYS for cancer. *Support Care Cancer*. 2013;21(3):889-899. doi:10.1007/s00520-012-1603-8

31. Hoskins G, Williams B, Abhyankar P, et al. Achieving Good Outcomes for Asthma Living (GOAL): Mixed methods feasibility and pilot cluster randomised controlled trial of a practical intervention for eliciting, setting and achieving goals for adults with asthma. *Trials*. 2016;17(1):1-17. doi:10.1186/s13063-016-1684-7

32. Houben CHM, Spruit MA, Luyten H, et al. Cluster-randomised trial of a nurse-led advance care planning session in patients with COPD and their loved ones. *Thorax*. 2019;74(4):328-336. doi:10.1136/thoraxjnl-2018-211943

33. Hyatt A, Lipson-Smith R, Gough K, et al. Culturally and linguistically diverse oncology patients' perspectives of consultation audio-recordings and question prompt lists. *Psychooncology*. 2018;27(9):2180-2188. doi:10.1002/pon.4789

34. Izumi SS, Basin B, Presley M, et al. Feasibility and Acceptability of Nurse-Led Primary Palliative Care for Older Adults with Chronic Conditions: A Pilot Study. *J Palliat Med*. 2018;21(8):1114-1121. doi:10.1089/jpm.2017.0666

35. Jonsdottir H, Amundadottir OR, Gudmundsson G, et al. Effectiveness of a partnership-based self-management programme for patients with mild and moderate chronic obstructive pulmonary disease: A pragmatic randomized controlled trial. *J Adv Nurs*. 2015;71(11):2634-2649. doi:10.1111/jan.12728

36. Kass NE, Sugarman J, Medley AM, et al. An intervention to improve cancer patients' understanding of early-phase clinical trials. *IRB Ethics Hum Res*. 2009;31(3):1-8.

37. Kennedy A, Bower P, Reeves D, et al. Implementation of self management support for long term conditions in routine primary care settings: Cluster randomised controlled trial. *BMJ*. 2013;346(7913):1-11. doi:10.1136/bmj.f2882

38. Koerner M, Wirtz M, Michaelis M, et al. A multicentre cluster-randomized controlled study to evaluate a train-the-trainer programme for implementing internal and external participation in medical rehabilitation. *Clin Rehabil*. 2014;28(1):20-35. doi:10.1177/0269215513494874

39. Krug K, Bossert J, Deis N, et al. Effects of an Interprofessional Communication Approach on Support Needs, Quality of Life, and Mood of Patients with Advanced Lung Cancer: A Randomized Trial. *Oncologist*. 2021;26(8):e1445-e1459. doi:10.1002/ONCO.13790

40. Lainscak M, Kadivec S, Kosnik M, et al. Discharge coordinator intervention prevents

For peer review only - http://bmjopen.bmj.com/site/about/guidelines.xhtml

hospitalizations in patients with COPD: A randomized controlled trial. *J Am Med Dir Assoc*. 2013;14(6):450.e1-450.e6. doi:10.1016/j.jamda.2013.03.003

41. Latimer AE, Williams-Piehota P, Cox A, Katulak NA, Salovey P, Mowad L. Encouraging cancer patients to talk to their physicians about clinical trials: Considering patients' information needs. *J Appl Biobehav Res*. 2007;12(3-4):178-195. doi:10.1111/j.1751-9861.2008.00020.x

42. Lavoie KL, Moullec G, Lemiere C, et al. Efficacy of brief motivational interviewing to improve adherence to inhaled corticosteroids among adult asthmatics: Results from a randomized controlled pilot feasibility trial. *Patient Prefer Adherence*. 2014;8:1555-1569. doi:10.2147/PPA.S66966

43. Leung JM, Udris EM, Uman J, Au DH. The effect of end-of-life discussions on perceived quality of care and health status among patients with COPD. *Chest*. 2012;142(1):128-133. doi:10.1378/chest.11-2222

44. Lim RK, Semitala FC, Atuhumuza E, et al. Patient choice improves self-efficacy and intention to complete tuberculosis preventive therapy in a routine HIV program setting in Uganda. *PLoS One*. 2021;16(2):e0246113. doi:10.1371/JOURNAL.PONE.0246113

45. Llanos-Paez C, Ambery C, Yang S, et al. Improved Decision-Making Confidence Using Item-Based Pharmacometric Model: Illustration with a Phase II Placebo-Controlled Trial. *AAPS J*. 2021;23(4). doi:10.1208/s12248-021-00600-1

46. Lopez-Lopez L, Valenza MC, Rodriguez-Torres J, Torres-Sanchez I, Granados-Santiago M, Valenza-Demet G. Results on health-related quality of life and functionality of a patient-centered self-management program in hospitalized COPD: a randomized control trial. *Disabil Rehabil*. 2020;42(25):3687-3695. doi:10.1080/09638288.2019.1609099

47. Ludden T, Shade L, Reeves K, et al. Asthma dissemination around patient-centered treatments in North Carolina (ADAPT-NC): a cluster randomized control trial evaluating dissemination of an evidence-based shared decision-making intervention for asthma management. *J Asthma*. September 2018:1-12. doi:10.1080/02770903.2018.1514630

48. Makimoto G, Hotta K, Oze I, et al. Patients' preferences and perceptions of lung cancer treatment decision making: results from Okayama lung cancer study group trial 1406. *Acta Oncol (Madr)*. 2020;59(3):324-328. doi:10.1080/0284186X.2019.1679880

49. Markman M, Petersen J, Montgomery R. An examination of characteristics of lung and colon cancer patients participating in a web-based decision support program: Internet-based

For peer review only - http://bmjopen.bmj.com/site/about/guidelines.xhtml

decision support programs. *Oncology*. 2005;69(4):311-316. doi:10.1159/000089763

50. Markun S, Rosemann T, Dalla-Lana K, Steurer-Stey C. Care in Chronic Obstructive Lung Disease (CAROL): A randomised trial in general practice. *Eur Respir J*. 2018;51(5). doi:10.1183/13993003.01873-2017

51. Mayer DK, Deal AM, Crane JM, et al. Using survivorship care plans to enhance communication and cancer care coordination: Results of a pilot study. *Oncol Nurs Forum*. 2016;43(5):636-645. doi:10.1188/16.ONF.636-645

52. Meline J, Prigge JM, Dye D, et al. Adapting the design of a Web-based decision support clinical trial during the COVID-19 pandemic. *Trials*. 2021;22(1):1-6. doi:10.1186/S13063-021-05700-Z/PEER-REVIEW

53. Melzer AC, Clothier BA, Japuntich SJ, et al. Comparative effectiveness of proactive tobacco treatment among smokers with and without chronic lower respiratory disease. *Ann Am Thorac Soc*. 2018;15(3):341-347. doi:10.1513/AnnalsATS.201707-582OC

54. Menon S, Mccullough LB, Beyth RJ, Ford ME, Espadas D, Braun UK. Use of a values inventory as a discussion aid about end-of-life care: A pilot randomized controlled trial. *Palliat Support Care*. 2016;14(4):330-340. doi:10.1017/S1478951515001091

55. Meropol NJ, Egleston BL, Buzaglo JS, et al. A Web-based communication aid for patients with cancer: The CONNECT Study. *Cancer*. 2013;119(7):1437-1445. doi:10.1002/cncr.27874

56. Moullec G, Lavoie KL, Rabhi K, Julien M, Favreau H, Labrecque M. Effect of an integrated care programme on re-hospitalization of patients with chronic obstructive pulmonary disease. *Respirology*. 2012;17(4):707-714. doi:10.1111/j.1440-1843.2012.02168.x

57. Murray MA, Stacey D, Wilson KG, O'Connor AM. Skills training to support patients considering place of end-of-life care: A randomized control trial. *J Palliat Care*. 2010;26(2):112-121. doi:10.1177/082585971002600207

58. Nedjat-Haiem FR, Carrion I V., Gonzalez K, et al. Implementing an Advance Care Planning Intervention in Community Settings with Older Latinos: A Feasibility Study. *J Palliat Med*. 2017;20(9):984-993. doi:10.1089/jpm.2016.0504

59. Nugent SM, Golden SE, Thomas CR, et al. Patient-clinician communication among patients with stage I lung cancer. *Support Care Cancer*. 2018;26(5):1625-1633. doi:10.1007/s00520-017-3992-1

For peer review only

For peer review only - http://bmjopen.bmj.com/site/about/guidelines.xhtml

60. Patel MR, Smith A, Leo H, Hao W, Zheng K. Improving Patient–Provider Communication and Therapeutic Practice Through Better Integration of Electronic Health Records in the Exam Room: A Pilot Study. *Heal Educ Behav*. 2019;46(3):484-493. doi:10.1177/1090198118796879

61. Rabow MW, Dibble SL, Pantilat SZ, McPhee SJ. The Comprehensive Care Team. *Arch Intern Med*. 2004;164(1):83. doi:10.1001/archinte.164.1.83

62. Rea H, McAuley S, Stewart A, Lamont C, Roseman P, Didsbury P. A chronic disease management programme can reduce days in hospital for patients with chronic obstructive pulmonary disease. *Intern Med J*. 2004;34(11):608-614. doi:10.1111/j.1445-5994.2004.00672.x

63. Reddel HK, Correll J, Foster J, Karlsson N, Martin M, Keen C. Patient Decision-Making Around Use of Reliever Inhalers in Mild Asthma. In: *American Thoracic Society International Conference Meetings Abstracts*. American Thoracic Society; 2019:A5935-A5935. doi:10.1164/ajrccm-conference.2019.199.1_meetingabstracts.a5935

64. Reinke LF, Feemster LC, McDowell J, et al. The long term impact of an end-of-life communication intervention among veterans with COPD. *Hear Lung J Acute Crit Care*. 2017;46(1):30-34. doi:10.1016/j.hrtlng.2016.10.003

65. Rhee H, Hollen PJ, Belyea MJ, Sutherland MA. Decision-Making Program for Rural Adolescents With Asthma: A Pilot Study. *J Pediatr Nurs*. 2008;23(6):439-450. doi:10.1016/j.pedn.2008.01.079

66. Roelands M, Van Oost P, Stevens V, Depoorter AM, Buysse A. Clinical practice guidelines to improve shared decision-making about assistive device use in home care: A pilot intervention study. *Patient Educ Couns*. 2004;55(2):252-264. doi:10.1016/j.pec.2003.10.002

67. Rosemann T. The Improving Care in Chronic Obstructive Lung Disease Study A Cluster Randomized Trial. *https://clinicaltrials.gov/show/NCT01921556*. 2013. doi:10.1002/CENTRAL/CN-02032168

68. Ruparel M, Quaife SL, Ghimire B, et al. Impact of a lung cancer screening information film on informed decision-making: A randomized trial. *Ann Am Thorac Soc*. 2019;16(6):744-751. doi:10.1513/AnnalsATS.201811-841OC

69. Rutten-vanMolken M, Goossens L, Boland M, et al. The Assessment of Burden of COPD (ABC) tool: a shared decision-making instrument that is predictive of healthcare costs. *Int J Integr Care*. 2017;17(5):320. doi:10.5334/ijic.3637

For peer review only - http://bmjopen.bmj.com/site/about/guidelines.xhtml

70. Sands M. A Randomised Control Trial for Advance Care Planning and Symptom Management for patients identified in the emergency department and followed up at home. *Clin Regist Clin Trials*. 2014. doi:10.1002/CENTRAL/CN-01881924

71. Schofield P, Ugalde A, Gough K, et al. A tailored, supportive care intervention using systematic assessment designed for people with inoperable lung cancer: A randomised controlled trial. *Psychooncology*. 2013;22(11):2445-2453. doi:10.1002/pon.3306

72. Schubart JR, Green MJ, Van Scoy LJ, et al. Advanced Cancer and End-of-Life Preferences: Curative Intent Surgery Versus Noncurative Intent Treatment. *J Palliat Med*. 2015;18(12):1015-1018. doi:10.1089/jpm.2015.0021

73. Schumacher KL, Koresawa S, West C, et al. Putting cancer pain management regimens into practice at home. *J Pain Symptom Manage*. 2002;23(5):369-382. doi:10.1016/S0885-3924(02)00385-8

74. Schwarze ML, Buffington A, Tucholka JL, et al. Effectiveness of a Question Prompt List Intervention for Older Patients Considering Major Surgery: A Multisite Randomized Clinical Trial. *JAMA Surg*. 2020;155(1):6-13. doi:10.1001/jamasurg.2019.3778

75. Ludden T, Shade L, Reeves K, et al. Asthma dissemination around patient-centered treatments in North Carolina (ADAPT-NC): a cluster randomized control trial evaluating dissemination of an evidence-based shared decision-making intervention for asthma management. *J Asthma*. 2019;56(10):1087-1098. doi:10.1080/02770903.2018.1514630

76. Shirai Y, Fujimori M, Ogawa A, et al. Patients' perception of the usefulness of a question prompt sheet for advanced cancer patients when deciding the initial treatment: A randomized, controlled trial. *Psychooncology*. 2012;21(7):706-713. doi:10.1002/pon.1955

77. Sinclair C, Auret KA, Evans SF, et al. Advance care planning uptake among patients with severe lung disease: A randomised patient preference trial of a nurse-led, facilitated advance care planning intervention. *BMJ Open*. 2017;7(2):1-11. doi:10.1136/bmjopen-2016-013415

78. Skorstengaard MH, Brogaard T, Bonde Jensen A, et al. Advance care planning for patients and their relatives. *Int J Palliat Nurs*. 2019;25(3):112-127. doi:10.12968/ijpn.2019.25.3.112

79. Slok A, Kotz D, van Breukelen G, et al. The assessment of burden of COPD tool improves health related quality of life. In: *European Respiratory Journal*. Vol 48. European Respiratory Society (ERS); 2016:OA1997. doi:10.1183/13993003.congress-2016.oa1997

80. Slok AHM, Kotz D, Van Breukelen G, et al. Effectiveness of the Assessment of Burden of COPD

For peer review only - http://bmjopen.bmj.com/site/about/guidelines.xhtml

(ABC) tool on health-related quality of life in patients with COPD: A cluster randomised controlled trial in primary and hospital care. *BMJ Open*. 2016;6(7). doi:10.1136/bmjopen-2016-011519

81. Smidth M, Olesen F, Fenger-Grøn M, Vedsted P. Patient-experienced effect of an active implementation of a disease management programme for COPD - A randomised trial. *BMC Fam Pract*. 2013;14(1):1. doi:10.1186/1471-2296-14-147

82. Steinhauser KE, Alexander SC, Byock IR, George LK, Olsen MK, Tulsky JA. Do preparation and life completion discussions improve functioning and quality of life in seriously ill patients? Pilot randomized control trial. *J Palliat Med*. 2008;11(9):1234-1240. doi:10.1089/jpm.2008.0078

83. Steurer C, Markun S, Rosemann T, Dalla Lana K. Improving Care in Chronic Obstructive Lung Disease (CAROL): a cluster randomized trial in primary care. In: European Respiratory Society (ERS); 2017:OA2915. doi:10.1183/1393003.congress-2017.oa2915

84. Steurer-Stey C, Storch M, Benz S, et al. Motivational training improves self-efficacy but not short-term adherence with asthma self-management: a randomized controlled trial. *Prim Health Care Res Dev*. 2015;16(1):32-41. doi:10.1017/S1463423613000480

85. Sullivan DR, Slatore CG, Stone K, et al. Associations Between Treatment and Team Composition with Decisional Regret Among Patients with Early Stage Lung Cancer. May 2020:A4726-A4726. doi:10.1164/AJRCCM-CONFERENCE.2020.201.1_MEETINGABSTRACTS.A4726

86. Tai-Seale M, Elwyn G, Wilson CJ, et al. Enhancing shared decision making through carefully designed interventions that target patient and provider behavior. *Health Aff*. 2016;35(4):605-612. doi:10.1377/hlthaff.2015.1398

87. Tamblyn R. Computer-Based Decision Support in Managing Asthma in Primary Care. *Clin Regist Clin Trials*. 2005. doi:10.1002/CENTRAL/CN-02024751

88. Tamblyn R, Ernst P, Winslade N, et al. Evaluating the impact of an integrated computer-based decision support with person-centered analytics for the management of asthma in primary care: A randomized controlled trial. *J Am Med Informatics Assoc*. 2015;22(4):773-783. doi:10.1093/jamia/ocu009

89. Tang JI, Shakespeare TP, Lu JJ, et al. Patients' preference for radiotherapy fractionation schedule in the palliation of symptomatic unresectable lung cancer. *J Med Imaging Radiat*

For peer review only - http://bmjopen.bmj.com/site/about/guidelines.xhtml

Oncol. 2008;52(5):497-502. doi:10.1111/j.1440-1673.2008.02002.x

90. Tapp H, Shade L, Mahabaleshwarkar R, Taylor YJ, Ludden T, Dulin MF. Results from a pragmatic prospective cohort study: Shared decision making improves outcomes for children with asthma. *J Asthma*. 2017;54(4):392-402. doi:10.1080/02770903.2016.1227333

91. Thee S, Stahl M, Fischer R, et al. A multi-centre, randomized, controlled trial on coaching and telemonitoring in patients with cystic fibrosis: conneCT CF. *BMC Pulm Med*. 2021;21(1):1-11. doi:10.1186/S12890-021-01500-Y/TABLES/1

92. Thiam S, LeFevre AM, Hane F, et al. Effectiveness of a strategy to improve adherence to tuberculosis treatment in a resource-poor setting: A cluster randomized controlled trial. *J Am Med Assoc*. 2007;297(4):380-386. doi:10.1001/jama.297.4.380

93. Totten A. Team-based Versus Primary Care Clinician-led Advance Care Planning in Practice-based Research Networks. *Clin Regist Clin Trials*. 2018. doi:10.1002/CENTRAL/CN-01660857

94. Tsao S, Willard-Grace R, Wolf J, et al. Implementation and Impact of the Pulmonary Specialist Health Coach Consultation Model to Improve Care for Patients with COPD. *Jt Comm J Qual patient Saf*. 2021;47(11):739-747. doi:10.1016/J.JCJQ.2021.08.003

95. Vandemheen KL, O'Connor A, Bell SC, et al. Randomized trial of a decision aid for patients with cystic fibrosis considering lung transplantation. *Am J Respir Crit Care Med*. 2009;180(8):761-768. doi:10.1164/rccm.200903-0421OC

96. Wahlberg H, Braaten T, Broderstad AR. Impact of referral templates on patient experience of the referral and care process: A cluster randomised trial. *BMJ Open*. 2016;6(10):1-8. doi:10.1136/bmjopen-2016-011651

97. Waller A, Girgis A, Johnson C, et al. Improving outcomes for people with progressive cancer: Interrupted time series trial of a needs assessment intervention. *J Pain Symptom Manage*. 2012;43(3):569-581. doi:10.1016/j.jpainsymman.2011.04.020

98. Wang KY, Chian CF, Lai HR, Tarn YH, Wu CP. Clinical pharmacist counseling improves outcomes for Taiwanese asthma patients. *Pharm World Sci*. 2010;32(6):721-729. doi:10.1007/s11096-010-9427-4

99. Weekes CE, Emery PW, Elia M. Dietary counselling and food fortification in stable copd: A randomised trial. *Thorax*. 2009;64(4):326-331. doi:10.1136/thx.2008.097352

100. Williams PD, Graham KM, Storlie DL, et al. Therapy-related symptom checklist use during

For peer review only - http://bmjopen.bmj.com/site/about/guidelines.xhtml

treatments at a cancer center. *Cancer Nurs*. 2013;36(3):245-254. doi:10.1097/NCC.0b013e3182595406

101. Wilson SR, Strub P, Knowles SB, Buist AS, Huang Q, Nguyen M. Ethnicity, Income, and Education as Potential Modifiers of the Effects of Shared Treatment Decision-Making (SDM) Between Asthma Care Manager and Patient on Asthma Controller Medication Adherence: The Better Outcomes of Asthma Treatment (BOAT) Trial. *J Allergy Clin Immunol*. 2009;123(2):S72-S72. doi:10.1016/j.jaci.2008.12.247

102. Wilson S. Does Shared Decision-Making Improve Asthma Outcomes? *Clin Regist Clin Trials*. 2005. doi:10.1002/CENTRAL/CN-02031001

103. Wilson S. Does shared decision-making improve adherence in asthma. *Clin Regist Clin Trials*. 2005. doi:10.1002/CENTRAL/CN-01427587

104. Wood-baker R, Reid D, Robinson A. Wood-Baker et al: Clinical trial of community nurse mentoring to improve self-management in patients with chronic obstructive pulmonary disease. International Journal of COPD 2012, 7:407-13. 2012:407-413. http://www.pubmedcentral.nih.gov/articlerender.fcgi?artid=3402057&tool=pmcentrez&rendertype=abstract.

105. Yoo SH, Yun YH, Kim KN, et al. The impact of caregiver's role preference on decisional conflicts and psychiatric distresses in decision making to help caregiver's disclosure of terminal disease status. *Qual Life Res*. 2018;27(6):1571-1581. doi:10.1007/s11136-018-1814-7

106. Zakrisson AB, Arne M, Hasselgren M, Lisspers K, Ställberg B, Theander K. A complex intervention of self-management for patients with COPD or CHF in primary care improved performance and satisfaction with regard to own selected activities; A longitudinal follow-up. *J Adv Nurs*. 2019;75(1):175-186. doi:10.1111/jan.13899

107. Zanartu CP, Camacho FJ, Nevadunski NS, et al. Speaking the same language: a feasibility trial for a novel visual communication tool for oncologist-patient treatment discussions. *Psychooncology*. 2017;26(7):1050-1052. doi:10.1002/pon.4246

108. Au DH, Udris EM, Engelberg RA, et al. A randomized trial to improve communication about end-of-life care among patients with COPD. *Chest*. 2012;141(3):726-735. doi:10.1378/chest.11-0362

109. Gagné ME, Légaré F, Moisan J, Boulet L-P. Impact of Adding a Decision Aid to Patient

For peer review only - http://bmjopen.bmj.com/site/about/guidelines.xhtml

Education in Adults with Asthma: A Randomized Clinical Trial. Leroyer C, ed. *PLoS One*. 2017;12(1):e0170055. doi:10.1371/journal.pone.0170055

110. Collinsworth AW, Brown RM, Sjames C, stanford RH, Alemayehu D, Priest EL. The impact of patient education and shared decision making on hospital readmissions for COPD. *Int J COPD*. 2018;13:1325-1332. doi:10.2147/COPD.S154414

111. Granados-Santiago M, Valenza MC, López-López L, Prados-Román E, Rodríguez-Torres J, Cabrera-Martos I. Shared decision-making and patient engagement program during acute exacerbation of COPD hospitalization: A randomized control trial. *Patient Educ Couns*. 2019;103(4):702-708. doi:10.1016/j.pec.2019.12.004

112. Brundage MD, Feldman-Stewart D, Cosby R, et al. Phase I study of a decision aid for patients with locally advanced non-small-cell lung cancer. *J Clin Oncol*. 2001;19(5):1326-1335. doi:10.1200/JCO.2001.19.5.1326

113. Myers RE, Advani SM, Myers P, et al. Engaging Patients with Late-Stage Non-Small Cell Lung Cancer in Shared Decision Making about Treatment. *J Pers Med*. 2021;11(10). doi:10.3390/JPM11100998

114. Walters J, Cameron-Tucker H, Wills K, et al. Effects of telephone health mentoring in community-recruited chronic obstructive pulmonary disease on self-management capacity, quality of life and psychological morbidity: A randomised controlled trial. *BMJ Open*. 2013;3(9). doi:10.1136/bmjopen-2013-003097

115. Wilson SR, Strub P, Buist AS, et al. Shared treatment decision making improves adherence and outcomes in poorly controlled asthma. *Am J Respir Crit Care Med*. 2010;181(6):566-577. doi:10.1164/rccm.200906-0907OC

For peer review only

For peer review only - http://bmjopen.bmj.com/site/about/guidelines.xhtml

PRISMA 2020 Checklist

Section and Topic	Item #	Checklist item	Location where item is reported
**TITLE**			
Title	1	Identify the report as a systematic review.	Title, pg1
**ABSTRACT**			
Abstract	2	See the PRISMA 2020 for Abstracts checklist.	Abstract, pg3-4
**INTRODUCTION**			
Rationale	3	Describe the rationale for the review in the context of existing knowledge.	Introduction, pg6-8
Objectives	4	Provide an explicit statement of the objective(s) or question(s) the review addresses.	Introduction, pg8
**METHODS**			
Eligibility criteria	5	Specify the inclusion and exclusion criteria for the review and how studies were grouped for the syntheses.	Methods, pg8-9
Information sources	6	Specify all databases, registers, websites, organisations, reference lists and other sources searched or consulted to identify studies. Specify the date when each source was last searched or consulted.	Methods, pg9
Search strategy	7	Present the full search strategies for all databases, registers and websites, including any filters and limits used.	Methods, pg10 & Supplementary Material
Selection process	8	Specify the methods used to decide whether a study met the inclusion criteria of the review, including how many reviewers screened each record and each report retrieved, whether they worked independently, and if applicable, details of automation tools used in the process.	Methods, pg10
Data collection process	9	Specify the methods used to collect data from reports, including how many reviewers collected data from each report, whether they worked independently, any processes for obtaining or confirming data from study investigators, and if applicable, details of automation tools used in the process.	Methods, pg10
Data items	10a	List and define all outcomes for which data were sought. Specify whether all results that were compatible with each outcome domain in each study were sought (e.g. for all measures, time points, analyses), and if not, the methods used to decide which results to collect.	Supplementary Material
	10b	List and define all other variables for which data were sought (e.g. participant and intervention characteristics, funding sources). Describe any assumptions made about any missing or unclear information.	Supplementary Material
Study risk of bias assessment	11	Specify the methods used to assess risk of bias in the included studies, including details of the tool(s) used, how many reviewers assessed each study and whether they worked independently, and if applicable, details of automation tools used in the process.	Methods, pg10-11
Effect measures	12	Specify for each outcome the effect measure(s) (e.g. risk ratio, mean difference) used in the synthesis or presentation of results.	Methods, pg11
Synthesis methods	13a	Describe the processes used to decide which studies were eligible for each synthesis (e.g. tabulating the study intervention characteristics and comparing against the planned groups for each synthesis (item #5)).	Methods, pg11
	13b	Describe any methods required to prepare the data for presentation or synthesis, such as handling of missing summary statistics, or data conversions.	Methods, pg11
	13c	Describe any methods used to tabulate or visually display results of individual studies and syntheses.	Methods, pg11
	13d	Describe any methods used to synthesize results and provide a rationale for the choice(s). If meta-analysis was performed, describe the model(s), method(s) to identify the presence and extent of statistical heterogeneity, and software package(s) used.	Methods, pg11

For peer review only - http://bmjopen.bmj.com/site/about/guidelines.xhtml

PRISMA 2020 Checklist

Section and Topic	Item #	Checklist item	Location where item is reported
	13e	Describe any methods used to explore possible causes of heterogeneity among study results (e.g. subgroup analysis, meta-regression).	Methods, pg11
	13f	Describe any sensitivity analyses conducted to assess robustness of the synthesized results.	N/A
Reporting bias assessment	14	Describe any methods used to assess risk of bias due to missing results in a synthesis (arising from reporting biases).	Methods, pg10-11
Certainty assessment	15	Describe any methods used to assess certainty (or confidence) in the body of evidence for an outcome.	Methods, pg11
**RESULTS**			
Study selection	16a	Describe the results of the search and selection process, from the number of records identified in the search to the number of studies included in the review, ideally using a flow diagram.	Results, pg11-12, Figure II
	16b	Cite studies that might appear to meet the inclusion criteria, but which were excluded, and explain why they were excluded.	Results, pg11-12, Figure II, Supplementary Material
Study characteristics	17	Cite each included study and present its characteristics.	Results, pg12, Tables I and II
Risk of bias in studies	18	Present assessments of risk of bias for each included study.	Results, pg12, Figure IV and V
Results of individual studies	19	For all outcomes, present, for each study: (a) summary statistics for each group (where appropriate) and (b) an effect estimate and its precision (e.g. confidence/credible interval), ideally using structured tables or plots.	Table I
Results of syntheses	20a	For each synthesis, briefly summarise the characteristics and risk of bias among contributing studies.	Results, pg12, Table I, Figure IV and V
	20b	Present results of all statistical syntheses conducted. If meta-analysis was done, present for each the summary estimate and its precision (e.g. confidence/credible interval) and measures of statistical heterogeneity. If comparing groups, describe the direction of the effect.	Results, pg13-16, Table I
	20c	Present results of all investigations of possible causes of heterogeneity among study results.	Supplementary Material
	20d	Present results of all sensitivity analyses conducted to assess the robustness of the synthesized results.	N/A
Reporting biases	21	Present assessments of risk of bias due to missing results (arising from reporting biases) for each synthesis assessed.	Results, pg12, Figure IV and V
Certainty of evidence	22	Present assessments of certainty (or confidence) in the body of evidence for each outcome assessed.	Table I
**DISCUSSION**			
Discussion	23a	Provide a general interpretation of the results in the context of other evidence.	Discussion, pg16-18
	23b	Discuss any limitations of the evidence included in the review.	Discussion, pg18

For peer review only

PRISMA 2020 Checklist

Section and Topic	Item #	Checklist item	Location where item is reported
	23c	Discuss any limitations of the review processes used.	Discussion, pg18-19
	23d	Discuss implications of the results for practice, policy, and future research.	Discussion, pg19
**OTHER INFORMATION**			
Registration and protocol	24a	Provide registration information for the review, including register name and registration number, or state that the review was not registered.	Other information, pg20
	24b	Indicate where the review protocol can be accessed, or state that a protocol was not prepared.	Other information, pg20
	24c	Describe and explain any amendments to information provided at registration or in the protocol.	N/A
Support	25	Describe sources of financial or non-financial support for the review, and the role of the funders or sponsors in the review.	Other information, pg20
Competing interests	26	Declare any competing interests of review authors.	Other information, pg20
Availability of data, code and other materials	27	Report which of the following are publicly available and where they can be found: template data collection forms; data extracted from included studies; data used for all analyses; analytic code; any other materials used in the review.	Other information, pg20

From: Page MJ, McKenzie JE, Bossuyt PM, Boutron I, Hoffmann TC, Mulrow CD, et al. The PRISMA 2020 statement: an updated guideline for reporting systematic reviews. BMJ 2021;372:n71. doi: 10.1136/bmj.n71

For more information, visit: http://www.prisma-statement.org/

For peer review only

Synthesis Without Meta-analysis (SWiM) reporting items

The citation for the Synthesis Without Meta-analysis explanation and elaboration article is: Campbell M, McKenzie JE, Sowden A, Katikireddi SV, Brennan SE, Ellis S, Hartmann-Boyce J, Ryan R, Shepperd S, Thomas J, Welch V, Thomson H. Synthesis without meta-analysis (SWiM) in systematic reviews: reporting guideline BMJ 2020;368:l6890 http://dx.doi.org/10.1136/bmj.l6890

**SWiM is intended to complement and be used as an extension to PRISMA**			
**SWiM reporting item**	**Item description**	**Page in manuscript where item is reported**	**Other***
Methods			
**1** Grouping studies for synthesis	1a) Provide a description of, and rationale for, the groups used in the synthesis (e.g., groupings of populations, interventions, outcomes, study design)	Lines 168-192	PROSPERO: CRD42020169897
	1b) Detail and provide rationale for any changes made subsequent to the protocol in the groups used in the synthesis	N/A	
**2** Describe the standardised metric and transformation methods used	Describe the standardised metric for each outcome. Explain why the metric(s) was chosen, and describe any methods used to transform the intervention effects, as reported in the study, to the standardised metric, citing any methodological guidance consulted	Lines 243-248	
**3** Describe the synthesis methods	Describe and justify the methods used to synthesise the effects for each outcome when it was not possible to undertake a meta-analysis of effect estimates	Lines 237-242	
**4** Criteria used to prioritise results for summary and synthesis	Where applicable, provide the criteria used, with supporting justification, to select the particular studies, or a particular study, for the main synthesis or to draw conclusions from the synthesis (e.g., based on study design, risk of bias assessments, directness in relation to the review question)	Lines 238-241	PROSPERO: CRD42020169897

For peer review only - http://bmjopen.bmj.com/site/about/guidelines.xhtml

Synthesis Without Meta-analysis (SWiM) reporting items

**SWiM reporting item**	**Item description**	**Page in manuscript where item is reported**	**Other***
**5** Investigation of heterogeneity in reported effects	State the method(s) used to examine heterogeneity in reported effects when it was not possible to undertake a meta-analysis of effect estimates and its extensions to investigate heterogeneity	Lines 237-241	
**6** Certainty of evidence	Describe the methods used to assess certainty of the synthesis findings	Lines 229-235	
**7** Data presentation methods	Describe the graphical and tabular methods used to present the effects (e.g., tables, forest plots, harvest plots).<br><br>Specify key study characteristics (e.g., study design, risk of bias) used to order the studies, in the text and any tables or graphs, clearly referencing the studies included	Lines 243-244	
Results			
**8** Reporting results	For each comparison and outcome, provide a description of the synthesised findings, and the certainty of the findings. Describe the result in language that is consistent with the question the synthesis addresses, and indicate which studies contribute to the synthesis	Lines 280-370 and Table 1	
Discussion			
**9** Limitations of the synthesis	Report the limitations of the synthesis methods used and/or the groupings used in the synthesis, and how these affect the conclusions that can be drawn in relation to the original review question	Lines 422-444	

For peer review only - http://bmjopen.bmj.com/site/about/guidelines.xhtml

Synthesis Without Meta-analysis (SWiM) reporting items

PRISMA=Preferred Reporting Items for Systematic Reviews and Meta-Analyses.
*If the information is not provided in the systematic review, give details of where this information is available (e.g., protocol, other published papers (provide citation details), or website (provide the URL)).

For peer review only

BMJ Open

A systematic review of shared decision-making interventions for people living with chronic respiratory diseases

Journal:	*BMJ Open*
Manuscript ID	bmjopen-2022-069461.R2
Article Type:	Original research
Date Submitted by the Author:	13-Apr-2023
Complete List of Authors:	Barradell, Amy; University of Leicester, Department of Respiratory Sciences; University Hospitals of Leicester NHS Trust, Centre for Exercise and Rehabilitation Science<br>Gerlis, Charlotte; University Hospitals of Leicester NHS Trust, Centre of Exercise and Rehabilitation Science<br>Houchen-Wolloff, Linzy; University Hospitals of Leicester NHS Trust, Centre of Exercise and Rehabilitation Science<br>Bekker, Hilary; University of Leeds, Leeds Institute of Health Sciences; Central Denmark Region and Aarhus University, Research Centre for Patient Involvement<br>Robertson, Noelle; University of Leicester, School of Psychology<br>Singh, Sally; University Hospitals of Leicester NHS Trust, Cardiac/Pulmonary Rehabilitation; University of Leicester, Respiratory Sciences
**Primary Subject Heading**:	Respiratory medicine
Secondary Subject Heading:	Patient-centred medicine, Research methods
Keywords:	RESPIRATORY MEDICINE (see Thoracic Medicine), REHABILITATION MEDICINE, Quality in health care < HEALTH SERVICES ADMINISTRATION & MANAGEMENT, Decision Making, Chronic airways disease < THORACIC MEDICINE

SCHOLARONE™
Manuscripts

I, the Submitting Author has the right to grant and does grant on behalf of all authors of the Work (as defined in the below author licence), an exclusive licence and/or a non-exclusive licence for contributions from authors who are: i) UK Crown employees; ii) where BMJ has agreed a CC-BY licence shall apply, and/or iii) in accordance with the terms applicable for US Federal Government officers or employees acting as part of their official duties; on a worldwide, perpetual, irrevocable, royalty-free basis to BMJ Publishing Group Ltd ("BMJ") its licensees and where the relevant Journal is co-owned by BMJ to the co-owners of the Journal, to publish the Work in this journal and any other BMJ products and to exploit all rights, as set out in our licence.

The Submitting Author accepts and understands that any supply made under these terms is made by BMJ to the Submitting Author unless you are acting as an employee on behalf of your employer or a postgraduate student of an affiliated institution which is paying any applicable article publishing charge ("APC") for Open Access articles. Where the Submitting Author wishes to make the Work available on an Open Access basis (and intends to pay the relevant APC), the terms of reuse of such Open Access shall be governed by a Creative Commons licence – details of these licences and which Creative Commons licence will apply to this Work are set out in our licence referred to above.

Other than as permitted in any relevant BMJ Author's Self Archiving Policies, I confirm this Work has not been accepted for publication elsewhere, is not being considered for publication elsewhere and does not duplicate material already published. I confirm all authors consent to publication of this Work and authorise the granting of this licence.

For peer review only

SDM Systematic Review

A systematic review of shared decision-making interventions for people living with chronic respiratory diseases

Authors

Barradell, AC[1-3]; Gerlis, C[3]; Houchen-Wolloff L[1,3]; Bekker, HL[5,6]; Robertson, N[4]; Singh, SJ[1,3].

Author affiliations:

1. Department of Respiratory Sciences, University of Leicester, University Road, Leicester LE1 7RH
2. National Institute for Health Research (NIHR) Applied Research Collaboration (East Midlands), College of Medicine, Biological Sciences & Psychology, Leicester General Hospital, Gwendolen Road, Leicester, LE5 4PW
3. Centre for Exercise and Rehabilitation Science, Leicester Biomedical Research Centre-Respiratory, Glenfield Hospital, Groby Road, Leicester, LE3 9QP
4. Department of Neuroscience, Psychology and Behaviour, University of Leicester, University Road, Leicester LE1 7RH
5. Leeds Unit of Complex Intervention Development (LUICD), School of Medicine, University of Leeds, Leeds, LS2 9JT
6. Research Centre for Patient Involvement, Central Denmark Region and Aarhus University, DK-8000 Aarhus C

Corresponding author:

Name: Amy C Barradell

Address: Centre for Exercise and Rehabilitation Science, Leicester Biomedical Research Centre-Respiratory, Glenfield Hospital, Groby Road, Leicester, LE3 9QP

Email: ab1081@le.ac.uk

Tel: 0116 258 3035

Short running title

A systematic review of SDM interventions in CRD

For peer review only

For peer review only - http://bmjopen.bmj.com/site/about/guidelines.xhtml

Abstract

Objective:

Shared Decision-Making (SDM) supports patients to make informed and value-based decisions about their care. We are developing an intervention to enable healthcare professionals to support patients' PR decision-making. To identify intervention components we needed to evaluate others carried out in Chronic Respiratory Diseases (CRD). We aimed to evaluate the impact of SDM interventions on patient decision-making (primary outcome) and downstream health-related outcomes (secondary outcome).

Design:

We conducted a systematic review using the risk of bias (Cochrane ROB2, ROBINS-I) and certainty of evidence (GRADE) tools.

Data sources:

MEDLINE, EMBASE, PSYCHINFO, CINAHL, PEDRO, Cochrane Central Register of Controlled Trials, the International Clinical Trials Registry Platform Search Portal, ClinicalTrials.gov, PROSPERO, ISRCTN were search through to 14th December 2021.

Eligibility criteria:

Trials evaluating SDM interventions in patients living with CRD using quantitative or mixed methods were included.

Data extraction and synthesis:

Two independent reviewers extracted data, assessed risk of bias, and certainty of evidence. A narrative synthesis, with reference to the The Making Informed Decisions Individually and Together (MIND-IT) model, was undertaken.

For peer review only

For peer review only - http://bmjopen.bmj.com/site/about/guidelines.xhtml

Results:

Eight studies [n=1,596 (of 17,466 citations identified)] fulfilled the inclusion criteria.

Five studies included components targeting the patient, healthcare professionals and consultation process (demonstrating adherence to the MIND-IT model). All studies reported their interventions improved patient decision-making and health-related outcomes. No outcome was reported consistently across studies. Four studies had high risk of bias, three had low quality of evidence. Intervention fidelity was reported in two studies.

Conclusions:

These findings suggest developing a SDM intervention including a patient decision aid, healthcare professional training, and a consultation prompt could support patient PR decisions, and health-related outcomes. Using a complex intervention development and evaluation research framework will likely lead to more robust research, and a greater understanding of service needs when integrating the intervention within practice.

Strengths and limitations

- The inclusion of studies from across the chronic respiratory disease population enabled us to draw conclusions about the efficacy of shared decision-making within this population when other reviews of specific respiratory conditions have been unable to.
- The search criteria may have meant we missed studies which used alternative terminology for shared decision making.
- We were unable to perform meta-analysis or sub-group analysis due to the heterogeneity of included studies and their outcome measures.

Medical Subject Headings

Informed decision making

Decision support

Patient-centred care

Professional-patient relations

For peer review only - http://bmjopen.bmj.com/site/about/guidelines.xhtml

Lung disease

Registration:

The systematic review protocol was registered on PROSPERO on 8th April 2020 (ID: CRD42020169897).

For peer review only

For peer review only - http://bmjopen.bmj.com/site/about/guidelines.xhtml

Introduction

Patients living with chronic obstructive pulmonary disease (COPD) account for a significant proportion of death and disability worldwide[1,2]. Pulmonary rehabilitation (PR) is a recommended evidence-based intervention to improve the physical and psychological health of this population[3] but patients express significant barriers to accepting PR[4,5]. It is therefore proposed, healthcare professionals should seek to engage patients in informed decisions about their enrolment into a programme[5,6].

Shared Decision-Making (SDM) is a core principle of personalised care, which encourages healthcare professionals to actively engage patients in healthcare decisions[7]. This style of communication requires patients and healthcare professionals to share knowledge about the health condition (i.e., the lived experience and evidence-based treatment), then engage in a period of deliberation to review the pros and cons of each option for the patients' life and agree upon an optimal healthcare choice.

This systematic review will inform the development and evaluation of a SDM intervention enabling healthcare professionals to support patients living with COPD make informed decisions about PR. SDM can be viewed as a complex intervention as it requires engagement from multiple stakeholders to enhance communication within a consultation. The Making Informed Decisions Individually and Together (MIND-IT) model[8] provides a conceptual framework to represent components within a multiple stakeholder decision support intervention and its evaluation (Figure 1). There are a range of interventions currently supporting SDM in consultations, including patient education materials, for example, Patient Decision Aids (PtDAs) with or without decision coaching, and SDM training for healthcare professionals.

PtDAs are resources providing accurate information to help people make informed, value-based decisions between healthcare options[7]. They are informed by decision science research to

For peer review only - http://bmjopen.bmj.com/site/about/guidelines.xhtml

structure the content in a way that reduces the cognitive effort needed to process facts, present balanced and neutral information, and provide prompts encouraging people to trade-off their evaluations of the consequences, and choose an option that is best for their life[9]. The integration of PtDAs into multiple healthcare contexts has shown to improve the frequency of SDM and patient-centred decisions, increase patient knowledge of their condition and the options available, enhance involvement in the decision-making process, and reduce decisional conflict[10–14].

Decision Coaching training for healthcare professionals aims to develop their skills in supporting patients to reason between healthcare options rather than providing information about a medical best treatment plan[15]. The training guides healthcare professionals to adopt a non-directive approach in SDM consultations by supporting patients to consider all available options, preparing them for deliberating over options, and ensuring any chosen option is then implemented. Research suggests decision coaching supports patient engagement in SDM[13,16–18] and in combination with a PtDA, is a robust method to facilitate SDM[16].

SDM training for healthcare professionals aims to help them adopt a SDM approach to delivering care[19]. There is no consensus guiding the content of this training, however NICE offers a training package[19] which includes a range of knowledge and skills-based modules on patient-centred care, presenting risk information and uncertainty, decision and cognitive science, evidence-based medicine, multiple stakeholder collaboration, and consultation skills. The evidence evaluating the effectiveness of this training on SDM use and impact in practice is weak due to the heterogeneity in study designs, methodology, and evaluation strategies[16,17].

SDM interventions are assessed using multiple outcome measures and include: within-consultation communication using healthcare professionals observation (e.g. the Observer OPTION 5 tool[20]) and patient self-report questionnaires (e.g. the Shared Decision-Making Questionnaire[21]), patient knowledge of healthcare options, attitudes, and skills (e.g. self-reported perception of risk, the Values' scale[22], the Decision Self Efficacy Scale[23]), patient decision outcomes (e.g. the choice

For peer review only

For peer review only - http://bmjopen.bmj.com/site/about/guidelines.xhtml

made, the Decisional Conflict Scale[24], the Decision Regret Scale[25]), overall SDM experience from patient and healthcare professionals perspectives (e.g. the iSHARE tool[26]), and downstream patient health-related outcomes (e.g. medication/treatment adherence, healthcare utilisation, disease knowledge, and quality of life measures).

In this systematic review we sought to evaluate the efficacy of SDM interventions compared to usual care in patients living with Chronic Respiratory Disease (CRD). To date, the efficacy of SDM interventions for adults living with CRD is largely unknown. There is a paucity of evidence to suggest patient educational materials (including PtDAs) are beneficial for reducing breathlessness and improving psychological well-being across acute respiratory diseases [27]. Two systematic reviews have been published to synthesise evidence on the impact of SDM interventions on people with asthma[28] and cystic fibrosis[29] but they found the evidence base to be very limited and too heterogeneous to draw meaningful conclusions about the relationship between the interventions and improved SDM or downstream health-related outcomes and nearly all the review population were children and adolescents. Therefore, there is a clear need to evaluate the efficacy of SDM interventions upon adult patients living with Chronic Respiratory Disease (CRD; e.g. COPD, asthma, idiopathic pulmonary fibrosis, lung cancer).

In this review, we aim to identify the components used to support SDM within this health context and synthesise evidence of their impact on patient decision-making and downstream health-related outcomes. We use the Preferred Reporting in Systematic Reviews and Meta-Analyses (PRISMA) guidelines to report this review[30].

(Insert Figure I)

For peer review only - http://bmjopen.bmj.com/site/about/guidelines.xhtml

Methods

Eligibility criteria

Participants

Adults (aged 18+ years) diagnosed with any CRD (and their carers).

Intervention

Interventions which orchestrated SDM between patients and healthcare professionals, with or without disease education, decision coaching training, or SDM training were sought. For the purpose of this review, interventions needed to report a patient-facing component which was designed to facilitate the decision-making process between a patient and clinician during a consultation (e.g. a patient education material).

.Comparison

Any concurrent control groups enrolled in the included studies, but not receiving a SDM intervention.

Outcomes

Primary outcomes of interest included measures of the quality of patient decision-making (e.g. participants' preparedness for decision-making, healthcare professionals and patient involvement in the decision-making process, knowledge of the available options and associated risks, decisional conflict, concordance between the participants' values and the decision made, decision confidence, and decisional regret).

Secondary outcomes of interest were patients' downstream health-related outcomes (e.g. behavioural activation, quality of life, knowledge of condition, healthcare utilisation). Specific qualitative measures of healthcare professionals' attitudes and experience of the SDM intervention, attendance, attrition, and fidelity were also included.

For peer review only

For peer review only - http://bmjopen.bmj.com/site/about/guidelines.xhtml

Study design

Randomised and non-randomised quantitative or mixed method controlled trials were sought for inclusion in this review. This included cluster trials, interrupted time series (ITS) studies with at least one data point before and after the intervention and controlled before-after (CBA) studies.

Exclusions were hypothetical decision scenarios, and studies not reported in English.

Information sources

An electronic search was conducted on the review's inception until 11th April 2023 using the following databases and registries: MEDLINE (1966-), EMBASE (1947-), PSYCHINFO (1887-), CINAHL (1937-), PEDRO (1999-), Cochrane Central Register of Controlled Trials (1993-) the International Clinical Trials Registry Platform Search Portal (2006-), ClinicalTrials.gov (2000-), PROSPERO (2011-), ISRCTN (2000-). Reference lists of selected studies and relevant systematic reviews were also searched to identify further articles for inclusion.

Search strategy

The search algorithm (Supplementary Material 1) was developed and piloted with support from the University Hospitals of Leicester NHS Trust Library Services. It included wide array of chronic respiratory diseases (e.g. tuberculosis, bronchiectasis, idiopathic pulmonary fibrosis). The algorithm was piloted using the MEDLINE database with limits imposed to retrieve articles in English. The algorithm was adapted to the syntax and MESH headings for each database.

Selection process

Articles were uploaded to the web-based platform, Rayaan (https://rayyan.qcri.org/welcome) to facilitate independent review. Duplicate articles were removed and articles reporting findings across multiple outputs were highlighted to review in tandem. Two reviewers (AB, CG) screened titles and abstracts using the inclusion and exclusion criteria. Eligible full texts were reviewed by the same two authors. Any ambiguity in the eligibility of an article was discussed and resolved with the co-authors.

For peer review only

For peer review only - http://bmjopen.bmj.com/site/about/guidelines.xhtml

Data collection process

A review-specific data extraction form was used, informed by Cochrane data extraction forms[31]. This was piloted prior to use to ensure consistency in the extraction process. One reviewer (AB) conducted data extraction. A random 10% selection was reviewed by a co-author (CG) to validate the extracted data. Since there were no errors in data extraction were identified, no further validation was considered necessary. Ambiguity in the extracted data was discussed by all authors and resolved by contacting individual study teams. Specific data items for primary and sub-group analysis are outlined in Supplementary Material 2.

Study risk of bias assessment

RCTs were assessed using the Cochrane Risk of Bias tool[32,33] which measures the following domains: randomisation process, deviations from intended interventions, missing outcome data, measurement of the outcomes, and selection of the reported result. Non-RCTs were assessed using the Cochrane ROBINS-I tool[34] which measures the following domains: confounding, selection of study participants, classification of interventions, deviations from intended interventions, missing data, measurement of outcomes, and selection of the reported result. Two authors (AB, CG) independently rated the studies. Any discrepancy in judgements were reviewed and resolved by the co-authors.

Certainty of evidence

The strength of evidence for all reported outcomes was assessed using the Grading of Recommendations Assessment, Development & Evaluation working group methodology (GRADE) which measures the following domains: risk of bias, inconsistency, indirectness, imprecision, and publication bias. Initial judgements were based upon study designs and then systematically reduced if the quality of evidence was poor or increased if a significant effect, dose response or evidence of the elimination of all plausible residual confounding variables and bias was observed. Two authors (AB, CG) independently assessed the criteria. Any discrepancies were resolved with the co-authors.

For peer review only - http://bmjopen.bmj.com/site/about/guidelines.xhtml

Synthesis methods

Statistical analysis of the results was not appropriate due to the clinical and methodological heterogeneity of the outcome measures between studies. A narrative synthesis, with reference to the MIND-IT model, was undertaken to identify the components used to support SDM in CRD and synthesise the evidence of their impact on patient decision-making and downstream health-related outcomes both between and within included studies. Guidance on the conduct of narrative synthesis in systematic reviews [35] was consulted to guide this process.

All outcomes were tabulated with continuous data presented as mean (standard deviation) and categorical data as percentage proportions. To allow consistency in reporting and synthesis of the results, data that was not published as means and standard deviations (e.g. instead reported as means and confidence intervals (CIs)) were transposed to this format using the formula reported in chapter 6 of the Cochrane Handbook for Systematic Reviews of Interventions (Version 6.3, 2022; (Higgins et al., 2022). These are acknowledged with an '*' in Table I.

Patient and Public Involvement

No patient and public involvement occurred during the development of the systematic review's research question, the choice of outcomes measures, the design and implementation of the review, or the interpretation of the results.

Results

Study selection

The search generated 17,488 articles. Following the removal of duplicates, 15,883 articles were retained for abstract screening. Of these, 117 underwent full text screening and eight were retained for inclusion in this analysis (109 excluded). Most studies were excluded because of the participant population (Figure II). Other reasons included study design, type of intervention, and type of publication. A full list of excluded studies is provided in Supplementary Material 3.

For peer review only - http://bmjopen.bmj.com/site/about/guidelines.xhtml

(Insert Figure II)

Study characteristics

Characteristics of the eight included studies[36–43] are provided in Tables I and II (an extended version of Table I is provided in Supplementary Material 4). Six studies were RCTs[36,38–42], two were non-RCT[37,43]. Two studies were conducted with COPD outpatients[36,41], two with COPD inpatients[38,40], two with lung cancer outpatients[37,43], and two with asthma outpatients[39,42]. The theoretical underpinning of interventions, reported by study authors, included social cognitive theory[36], principles of self-management and SDM[38], SDM[39,42,43], self-management[40] and cognitive behavioural psychology[41]. One study[37] did not report a theoretical underpinning. The interventions of five studies included all three components of the MIND-IT multiple stakeholder model (Figure III). Four studies included additional components to SDM within their intervention groups; two included a self-management programme[40,41], and three included an educational component[38,39,41].

(Insert Tables I and II)

(Insert Figure III)

Risk of bias within studies

Two RCTs were judged to have high risk of bias[38,41] and four RCTs were judged to have some concerns [36,39,40,42] (Figure IV). One study was judged as high due to insufficient efforts in randomisation, blinding and attrition[38], the other was due to attrition and poor fidelity[41]. The two non-RCTs were both judged to have serious risk of bias because of insufficient efforts in blinding[37,43] (Figure V).

(Insert Figure IV and V)

For peer review only

For peer review only - http://bmjopen.bmj.com/site/about/guidelines.xhtml

Results of synthesis

Results of individual studies are presented in turn alongside certainty of evidence assessments in Table I.

Decision-making outcomes

Five outcomes captured the quality of patient decision-making (Table I); quality of communication, involvement in decision-making, decisional conflict, strength of treatment preference, and knowledge of treatment options.

Quality of communication

One study reported that quality of communication increased in intervention and control groups, however, a significantly greater increase occurred in the intervention group alone [mean between group difference: 5.7 points; $p<0.05$][36]. The same study reported an increase in the occurrence of patient-centred communication in the intervention group alone, as measured by a study-specific 4-item self-reported questionnaire [mean(SD) Item 1. Intervention: 35.2(74.6) Control: 15.9(58.2) $p<0.05$; Item 2. Intervention: 60.3(122.9) Control: 30.8(175.5) $p<0.05$; Item 3. Intervention: 53.6(115.1) Control: 45.2(73.3) $p>0.05$; Item 4. Intervention: 86. 2(84.6) Control: 75.2(115.0) $p<0.05$].

Involvement in decision-making

Two studies reported significantly increased patient involvement in the decision-making process when compared to a control group [6months mean(SD) between group difference: 0.4(0.2), $p<0.05$; 12 months mean(SD) between group difference: 0.3(0.1), $p<0.05$][41] and a group where clinicians made treatment decisions [immediately post-intervention mean(SD) between group difference: 2.5(0.9), $p<0.05$][42].

Decisional conflict

Two studies (one feasibility, one pilot controlled before and after study) reported a trend for reducing decisional conflict. The first reported 87% reduction in decisional conflict[37], the other reported a mean difference of +1 indicating increased decision certainty[43]. Elsewhere, a RCT

For peer review only - http://bmjopen.bmj.com/site/about/guidelines.xhtml

For peer review only

measuring disease education with and without a patient decision aid reported significantly reduced decisional conflict in the intervention and control groups [Intervention mean(SD) difference from baseline: 8.1(22.5), p<0.05; Control mean (SD) difference from baseline: 9.1(27.7), p<0.05], but no significant mean difference between groups [between group mean difference: 2.5, p>0.05)[39].

Strength of treatment preference

One feasibility study reported the strength of patients' treatment preference increased from 80-100% following exposure to a patient decision aid[37].

Knowledge of treatment options

One feasibility study reported an increasing trend for treatment specific knowledge for three year outcome survival[a] and median survival outcome[b] [Knowledge of survival outcomes with treatment 1 alone (mean between group difference: 60%[a]; 54%[b]); Knowledge of direction of survival difference between treatments (mean between group difference: 27%[a]; 40%[b]); Knowledge of the magnitude of survival difference between treatments (mean between group difference: 73%[a]; 67%[b])][37].

A pilot controlled before and after study reported awareness of treatment options increased from 40% to 100% of participants post intervention[43].

Health-related outcomes

Six outcomes captured downstream health-related outcomes (Table I); disease knowledge, hospitalisations, behavioural activation quality of life, intervention fidelity, attendance, and attrition.

Disease knowledge

One study reported significantly increased disease-specific knowledge scores in the intervention group [mean(SD) between group difference: 3.89(0.7), p<0.05] which maintained at 3 months follow up [mean(SD) between group difference: 3.88(0.8), p<0.05][40]. However, another study reported knowledge scores increased equally amongst intervention and control groups [mean difference (SD) Intervention: 3.6(8.9), p<0.05; Control: 2(7.3), p<0.05][39].

For peer review only

For peer review only - http://bmjopen.bmj.com/site/about/guidelines.xhtml

Hospitalisations

Two studies reported no change in all-cause hospitalisations between intervention and control groups[38,41]. One study observed a significant reduction in healthcare utilisation in the SDM and clinician decision-making groups but not in the control group [SDM-control mean(SD) between group difference: 20.36(1.5), p<0.05; SDM-clinician decision-making mean(SD) between group difference: 0.01(2.7), p>0.05; clinician decision-making-control mean(SD) between group difference: 20.37(1.5), p<0.05][42].

Behavioural activation

One feasibility study showed both the intervention and control group had a trend for increased levels of activation post intervention [mean(SD) between group difference: 0.52(0.9) and 0.69(1.0) points respectively, p<0.05][38].

One pilot controlled before and after study reported 80% of participants had made a treatment decision at 30 days and 40% had begun a treatment which matched their treatment preference[43].

Two studies reported medication adherence significantly improved in the intervention groups alone[40,42]. However, the control group receiving a clinician decision-making intervention in one of these studies[42] also showed significantly adherent medication behaviour and no between group difference [SDM–clinician decision-making mean(SD) between group difference: 0.08(0.4), p>0.05]. One study measured the correct administration of medication and post intervention reported non-significant improvements in the correct use of asthma medication in the intervention and the control group with no significant difference between groups [mean(SD) between group difference: 0.17(0.7), p>0.05][39].

One study reported significant between group differences for improvements in nutritional behaviour at 3 months post inpatient intervention initiation [mean between group difference: 5.03; p<0.05) and physical activity at 3 months post intervention initiation (mean between group difference: 716.01; p<0.05][40].

For peer review only - http://bmjopen.bmj.com/site/about/guidelines.xhtml

Quality of life

Two studies reported no significant difference between intervention and control groups post intervention [Discharge mean(SD) between group difference: 6.15(27.5), $p>0.05$); 6 months mean(SD) between group difference: 1.9(2.7), $p>0.05$); 12 months mean(SD) between group difference: 1.4(1.5), $p>0.05$][40,41]. However, one of the studies did at 3 months post discharge [mean(SD) between group difference: 8.28(50.6), $p<0.05$][40]. Whilst two further studies reported either a trend for improvement [mean(SD) between group difference: 4.89; $p<0.05$)[38] or a significant improvement in the intervention group alone [SDM to control mean(SD) between group difference: 0.39(1.0), $p<0.05$][42].

Intervention fidelity

Only two studies reported intervention fidelity explicitly[41,42]. Both studies rated a specified selection of intervention audio recordings. One reported high adherence to the intervention protocol [SDM, 4/5; clinician decision-making, 3.9/5][42]. The other reported wide variation in adherence between the different intervention components, with lower delivery of cognitive-behavioural components which were described as the backbone of the intervention[41].

Attendance and attrition

For inventions involving single sessions, there was no attrition during intervention delivery[36,37,39,43]. For interventions involving multiple sessions, completion of inpatient interventions ranged from 37%[38] to 100%[40], whereas, outpatient interventions ranged from 72%[41] to 75.5%[42].

Results from sub-group analysis are provided in Supplementary Material 5a and 5b.

Discussion

This review provides the first synthesis of studies evaluating interventions to support SDM between patients with CRD and healthcare professionals. All eight studies reported their interventions improved patient decision-making and downstream health-related outcomes. There were improvements in the quality of communication with healthcare professionals[36], the occurrence of

For peer review only

368 patient-centred communication[36], involvement in decision-making[41,42], decisional
369 conflict[39,43], health-related knowledge[40], behavioural activation[40,42,43], quality of
370 life[40,42], and healthcare utilisation[42]. There was no one consistent outcome measure used in all
371 studies to enable comparison across studies. Additionally, three studies measured intervention
372 feasibility meaning they were unable to observe intervention efficacy[37,38,43], two RCTs were
373 judged to have a high risk of bias [38,41] and two non-RCTs were judged to have a serious risk of bias
374 [37,43].The most consistently reported outcomes were the downstream health-related outcomes
375 behavioural activation and quality of life. Five studies reported improvements in behavioural
376 activation across six outcome measures[38–40,42,43]. Between group differences were only
377 observed for two of the outcomes (nutritional status and physical activity) and this was in just one
378 study[40]. Two studies reported improved health-related quality of life post intervention[38,42] but
379 one of these was a feasibility study[38].

380 Unlike other reviews in the CRD field[28,29], this review found variation in the intervention
381 components. Our appraisal showed that only five studies contained all active SDM components from
382 the MIND-IT model (Figure III). Gagne's[39] and Au's[36] study did not report a period of deliberation
383 between patients and healthcare professionals, suggesting the decision-making process was not
384 shared, and in Walters[41] study, there was no report of patients and healthcare professionals
385 exchanging personal and evidence-based information suggesting the decision-making process was
386 not informed. Without adequate inclusion of all SDM components, it is difficult to conclude which
387 intervention components support which outcomes.

388 Furthermore, there was little evidence in the descriptions of the interventions to show they had
389 been developed in-line with recommended SDM intervention and user-centred design guidelines
390 (e.g. [44],[45] and [46]). There was also limited evidence to suggest included studies had identified
391 their target populations' (e.g. patient or healthcare professionals) needs when developing their
392 intervention and its evaluation. For example, despite the vast array of participants' educational

For peer review only - http://bmjopen.bmj.com/site/about/guidelines.xhtml

attainment across studies, there was little evidence studies observed this and tailored the SDM materials accordingly. This observation is of concern because lower health literacy has been shown to affect key decision-making outcomes[47]. A recent expanded model of SDM has been proposed to reflect the necessity of patients' health literacy skills as this enables them to have meaningful engagement in the decision-making process[48]. In our review, no study had considered the health literacy of their participants prior to enrolment through the use of validated measures (e.g. test of functional health literacy in adults[49]), nor the use of a standardising health literacy resources such as the Plain English Campaign[50] to ensure materials were at the appropriate comprehension level and, finally, no study had considered using the low literacy version of the Decisional Conflict Scale[51], despite its ease of access. Furthermore, no study reported healthcare professional-directed intervention components (e.g. healthcare professional training) or used a user-centred design or implementation process.

Additionally, studies made little reference to social or familial support in the decision-making process, which is unusual as social support is often associated with improvements in self-management behaviour amongst patients with CRD[52,53]. Social support may contribute to informed information exchange and provide confidence in the decision-making process. Interestingly, one study specifically excluded family members or friends from the SDM consultation as they believed it would introduce bias[37].

Limitations of the evidence included in the review

The interpretation of these results should be considered in light of the inclusion of studies which utilised non-validated outcome measures. Only one study used a dose-matched intervention in the control group[41] and fidelity was only reported explicitly in two studies[41,42].

Limitations of the review process

The inconsistent outcome measures within this review precluded meta-analysis. Four studies had a high risk of bias[37,38,41,43] and three had low or very low quality of evidence[37,41,43], thereby

For peer review only

For peer review only - http://bmjopen.bmj.com/site/about/guidelines.xhtml

limiting the validity and generalisability of the results. The search criteria were broad, however, if studies had used alternate terminology for SDM, it is possible some may have been missed. The multi-component interventions meant intervention effects could not be discernibly attributed to the SDM component or the education and self-management components. Furthermore, two studies both provided their control groups with disease education which may have driven the positive effects observed by the control groups[38,39]. Included studies encompassed a variety of healthcare settings with decisions varying in focus from self-management to treatment decision-making and end of life care. Despite excluding studies involving hypothetical decision-making to reduce the disparity in importance attributed to the decision-making process, we cannot assume participants attributed the same importance to all decisions. Additionally, the studies length of follow-ups varied considerably, however, as the type of decisions were so varied the allocated follow-up period may have been appropriate. For example, Janssen and colleagues showed that patients with COPD change their mind about end of life care throughout the course of their healthcare journey[54], suggesting a long-term follow-up period for end of life decision-making is appropriate. Whereas health decisions with more immediate affect (e.g. acceptance of a referral to pulmonary rehabilitation) may require a shorter follow-up period. As more SDM intervention studies are conducted in CRD, an updated review may well benefit from statistical sub-group analysis for studies length of follow up and/or decision posed. Additionally, the decision to exclude qualitative and cross-sectional studies may have limited our ability to fully explore participants' experiences of the SDM process and the development and implementation of interventions.

Implications of the results

This synthesis provides some evidence that CRD SDM interventions improve the patient decision-making process and downstream health-related outcomes. However, the evidence is weak, and the interventions at times incongruent with all active SDM components. Therefore, this limits the rigour

For peer review only - http://bmjopen.bmj.com/site/about/guidelines.xhtml

in evaluating the impact of SDM interventions within CRD. There were also no studies exploring the efficacy of SDM in PR decision-making and implementation in practice.

Based on the available evidence, this review suggests the development of a SDM intervention to enable healthcare professionals to support COPD patients informed decision-making for PR may be beneficial. It recommends that such intervention should be evaluated within a complex intervention development and evaluation research framework. This is especially relevant given the rise in alternate PR delivery models due to the Covid-19 pandemic. Importantly, this intervention should be underpinned by SDM theoretical models (e.g. MIND-IT[8], extended SDM model involving consideration of health literacy[48]), adhere to the appropriate intervention development guidelines (e.g.[44–46]), and be evaluated in a single or double-blind RCT with dose-matched controls.

Other information

Protocol and registration

This review was conducted in accordance with the Preferred Reporting in Systematic Reviews and Meta-Analyses (PRISMA) and registered on PROSPERO (registration number: CRD42020169897). The completed PRISMA checklist was provided at article submission.

Funding statement

The National Institute for Health Research (NIHR Applied Research Collaboration [East Midlands] (NIHR ARC East Midlands) and the Centre for Exercise and Rehabilitation Science (CERS) funded this research. Award/Grant number is not applicable.

Competing interests

The authors have no conflicts of interest to declare.

For peer review only

For peer review only - http://bmjopen.bmj.com/site/about/guidelines.xhtml

Availability of data, code, and other materials

The MEDLINE search strategy, data items sought, methods for sub-group analysis, characteristics of excluded studies, and results of sub-group analysis are provided in the Supplementary Materials. No additional data available.

Acknowledgements

The authors would like to thank the UHL Library Services for their contribution to the search criteria and piloting the search. We would like to thank all study authors for sharing their data, when requested. Professor Singh is a National Institute for Health Research (NIHR) Senior Investigator. The views expressed in this article are those of the author(s) and not necessarily those of the NIHR, or the Department of Health and Social Care.

Contributorship statement

ACB conceived the review. ACB, CG, LHW, HLB, NR and SJS contributed the planning, design, and protocol development. ACB conducted the search in collaboration with the University Hospitals of Leicester NHS Trust Librarian. ACB and CG screened all citations. ACB contacted study authors for additional data acquisition. ACB, CG, LHW, NR and SJS confirmed studies to be included. ACB conducted data extraction and CG conducted a 10% data quality check to ensure accuracy in this process. ACB and CG conducted risk of bias and certainty of evidence assessments. ACB conducted the narrative synthesis and ACB, CG, LHW, NR and SJS contributed to interpretation of the results. ACB drafted the manuscript, and CG, LHW, NR and SJS critically revised it for significant intellectual content and insight. In addition, ACB, CG, LHW, NR and SJS gave final approval of the manuscript version for publication. ACB, CG, LHW, NR and SJS are responsible for the overall content as guarantors. ACB, CG, LHW, NR and SJS report no conflicts of interest.

For peer review only - http://bmjopen.bmj.com/site/about/guidelines.xhtml

Disclosure statement

The National Institute for Health Research (NIHR Applied Research Collaboration [East Midlands] (NIHR ARC East Midlands) and the Centre for Exercise and Rehabilitation Science (CERS) funded this research. Award/Grant number is not applicable. The funders had no role in the inception, data extraction or analysis phases of this review. The views expressed in this article do not communicate an official position of the University of Leicester, NIHR, CERS, University of Leeds, or Aarhus University.

Ethics Approval Statement

This study does not involve human participants. Research Ethics approval was not warranted.

References

1 Lozano R, Naghavi M, Foreman K, *et al.* Global and regional mortality from 235 causes of death for 20 age groups in 1990 and 2010: A systematic analysis for the Global Burden of Disease Study 2010. *Lancet* 2012;**380**:2095–128. doi:10.1016/S0140-6736(12)61728-0

2 Vos T, Flaxman AD, Naghavi M, *et al.* Years lived with disability (YLDs) for 1160 sequelae of 289 diseases and injuries 1990-2010: A systematic analysis for the Global Burden of Disease Study 2010. *Lancet* 2012;**380**:2163–96. doi:10.1016/S0140-6736(12)61729-2

3 Mccarthy B, Casey D, Devane D, *et al.* Pulmonary rehabilitation for chronic obstructive pulmonary disease. Cochrane Database Syst. Rev. 2015;**23**. doi:10.1002/14651858.CD003793.pub3

4 Keating A, Lee A, Holland AE. What prevents people with chronic obstructive pulmonary disease from attending pulmonary rehabilitation? A systematic review. Chron. Respir. Dis. 2011;**8**:89–99. doi:10.1177/1479972310393756

5 Keating A, Lee AL, Holland AE. Lack of perceived benefit and inadequate transport influence

For peer review only

uptake and completion of pulmonary rehabilitation in people with chronic obstructive pulmonary disease: A qualitative study. *J Physiother* 2011;**57**:183–90. doi:10.1016/S1836-9553(11)70040-6

6 Blackstock FC, Wallack RZ, Nici L, *et al.* Why don't our patients with chronic obstructive pulmonary disease listen to us? the enigma of nonadherence. Ann. Am. Thorac. Soc. 2016;**13**:317–23. doi:10.1513/AnnalsATS.201509-600PS

7 NICE. Shared decision making | NICE guidelines | NICE guidance | Our programmes | What we do | About | NICE. NICE 2021. https://www.nice.org.uk/about/what-we-do/our-programmes/nice-guidance/nice-guidelines/shared-decision-making (accessed 7 Feb 2022).

8 Breckenridge K, Bekker HL, Gibbons E, *et al.* How to routinely collect data on patient-reported outcome and experience measures in renal registries in Europe: An expert consensus meeting. Nephrol. Dial. Transplant. 2015;**30**:1605–14. doi:10.1093/ndt/gfv209

9 Bekker H, Thornton J, Airey C, *et al.* Informed decision making: An annotated bibliography and systematic review. Health Technol. Assess. (Rockv). 1999;**3**. doi:10.3310/hta3010

10 Stacey D, Bennett CL, Barry MJ, *et al.* Decision aids for people facing health treatment or screening decisions. In: Stacey D, ed. *Cochrane Database of Systematic Reviews*. Chichester, UK: : John Wiley & Sons, Ltd 2011. CD001431. doi:10.1002/14651858.cd001431.pub3

11 Stacey D, Légaré F, Lewis K, *et al.* Decision aids for people facing health treatment or screening decisions. Cochrane Database Syst. Rev. 2017;**2017**. doi:10.1002/14651858.CD001431.pub5

12 Hoffman AS, Volk RJ, Saarimaki A, *et al.* Delivering patient decision aids on the Internet: Definitions, theories, current evidence, and emerging research areas. BMC Med. Inform. Decis. Mak. 2013;**13**:S13. doi:10.1186/1472-6947-13-S2-S13

For peer review only

For peer review only - http://bmjopen.bmj.com/site/about/guidelines.xhtml

531 13 Sepucha KR, Borkhoff CM, Lally J, *et al.* Establishing the effectiveness of patient decision aids: Key constructs and measurement instruments. *BMC Med Inform Decis Mak* 2013;**13**:S12. doi:10.1186/1472-6947-13-S2-S12

534 14 Feldman-Stewart D, O'Brien MA, Clayman ML, *et al.* Providing information about options in patient decision aids. *BMC Med Inform Decis Mak* 2013;**13**:S4. doi:10.1186/1472-6947-13-S2-S4

537 15 Jull J, Köpke S, Smith M, *et al.* Decision coaching for people making healthcare decisions. *Cochrane Database Syst Rev* 2021;:CD013385. doi:10.1002/14651858.CD013385.PUB2/MEDIA/CDSR/CD013385/IMAGE_N/NCD013385-CMP-004.01.SVG

541 16 Légaré F, Ratté S, Stacey D, *et al.* Interventions for improving the adoption of shared decision making by healthcare professionals. In: *Cochrane Database of Systematic Reviews*. John Wiley & Sons, Ltd 2010. doi:10.1002/14651858.cd006732.pub2

544 17 Müller E, Diesing A, Rosahl A, *et al.* Evaluation of a shared decision-making communication skills training for physicians treating patients with asthma: A mixed methods study using simulated patients. *BMC Health Serv Res* 2019;**19**:612. doi:10.1186/s12913-019-4445-y

547 18 Stacey D, Kryworuchko J, Belkora J, *et al.* Coaching and guidance with patient decision aids: A review of theoretical and empirical evidence. *BMC Med Inform Decis Mak* 2013;**13**. doi:10.1186/1472-6947-13-S2-S11

550 19 NICE. Shared decision making learning package. 2021.https://www.nice.org.uk/guidance/ng197/resources/shared-decision-making-learning-package-9142488109 (accessed 7 Feb 2022).

553 20 Elwyn G, Tsulukidze M, Edwards A, *et al.* Using a 'talk' model of shared decision making to

For peer review only - http://bmjopen.bmj.com/site/about/guidelines.xhtml

propose an observation-based measure: Observer OPTION5 Item. *Patient Educ Couns* 2013;**93**:265–71. doi:10.1016/j.pec.2013.08.005

21 Simon D, Schorr G, Wirtz M, *et al.* Development and first validation of the shared decision-making questionnaire (SDM-Q). *Patient Educ Couns* 2006;**63**:319–27. doi:10.1016/j.pec.2006.04.012

22 O'Connor A. Sample Tool: Values (Tamoxifen). 1999.www.ohri.ca/decisionaid (accessed 19 Feb 2020).

23 O'Connor AM. Sample Tool: Decision Self-Efficacy Scale. 1995.

24 O'Connor AM. Validation of a Decisional Conflict Scale. *Med Decis Mak* 1995;**15**:25–30. doi:10.1177/0272989X9501500105

25 Brehaut JC, O'Connor AM, Wood TJ, Hack TF, Siminoff L, Gordon E F-SD. Validation of a decision regret scale. *Med Decis Making* 2003;**23**:281–92.

26 Bomhof-Roordink H, Gärtner FR, van Duijn-Bakker N, *et al.* Measuring shared decision making in oncology: Development and first testing of the iSHAREpatient and iSHAREphysician questionnaires. *Heal Expect* 2020;**23**:496–508. doi:10.1111/HEX.13015

27 Sunjaya AP, Bao L, Martin A, *et al.* Systematic review of effectiveness and quality assessment of patient education materials and decision aids for breathlessness. *BMC Pulm Med* 2022;**22**:1–14. doi:10.1186/S12890-022-02032-9/FIGURES/5

28 Kew KM, Malik P, Aniruddhan K NR. Shared decision-making for people with asthma. *Cochrane Database Syst Rev* 2017.

29 Malone H, Biggar S, Javadpour S, *et al.* Interventions for promoting participation in shared decision-making for children and adolescents with cystic fibrosis. *Cochrane Database Syst Rev*

For peer review only

For peer review only - http://bmjopen.bmj.com/site/about/guidelines.xhtml

2019;**2019**. doi:10.1002/14651858.CD012578.pub2

30 Page MJ, McKenzie JE, Bossuyt PM, *et al.* The PRISMA 2020 statement: An updated guideline for reporting systematic reviews. BMJ. 2021;**372**. doi:10.1136/bmj.n71

31 Cochrane. Data extraction forms | Cochrane Developmental, Psychosocial and Learning Problems. 2021.https://dplp.cochrane.org/data-extraction-forms (accessed 16 Mar 2021).

32 Sterne JAC, Savović J, Page MJ, *et al.* RoB 2: A revised tool for assessing risk of bias in randomised trials. *BMJ* 2019;**366**:l4898. doi:10.1136/bmj.l4898

33 Higgins J, Thomas J, Chandler J, *et al.* Cochrane Handbook for Systematic Reviews of Interventions version 6.3 (updated February 2022). 2022. Available from www.training.cochrane.org/handbook.

34 Sterne JA, Hernán MA, Reeves BC, *et al.* ROBINS-I: a tool for assessing risk of bias in non-randomised studies of interventions. *BMJ* 2016;**355**. doi:10.1136/BMJ.I4919

35 Popay J, Roberts H, Sowden A, *et al.* Guidance on the conduct of narrative synthesis in systematic reviews. A product from the ESRC methods programme. Version, 1.No Title. 2006. doi:10.13140/2.1.1018.4643

36 Au DH, Udris EM, Engelberg RA, *et al.* A randomized trial to improve communication about end-of-life care among patients with COPD. *Chest* 2012;**141**:726–35. doi:10.1378/chest.11-0362

37 Brundage MD, Feldman-Stewart D, Cosby R, *et al.* Phase I study of a decision aid for patients with locally advanced non-small-cell lung cancer. *J Clin Oncol* 2001;**19**:1326–35. doi:10.1200/JCO.2001.19.5.1326

38 Collinsworth AW, Brown RM, Sjames C, *et al.* The impact of patient education and shared

For peer review only

For peer review only - http://bmjopen.bmj.com/site/about/guidelines.xhtml

decision making on hospital readmissions for COPD. *Int J COPD* 2018;**13**:1325–32. doi:10.2147/COPD.S154414

39 Gagné ME, Légaré F, Moisan J, *et al.* Impact of Adding a Decision Aid to Patient Education in Adults with Asthma: A Randomized Clinical Trial. *PLoS One* 2017;**12**:e0170055. doi:10.1371/journal.pone.0170055

40 Granados-Santiago M, Valenza MC, López-López L, *et al.* Shared decision-making and patient engagement program during acute exacerbation of COPD hospitalization: A randomized control trial. *Patient Educ Couns* 2019;**103**:702–8. doi:10.1016/j.pec.2019.12.004

41 Walters J, Cameron-Tucker H, Wills K, *et al.* Effects of telephone health mentoring in community-recruited chronic obstructive pulmonary disease on self-management capacity, quality of life and psychological morbidity: A randomised controlled trial. *BMJ Open* 2013;**3**. doi:10.1136/bmjopen-2013-003097

42 Wilson SR, Strub P, Buist AS, *et al.* Shared treatment decision making improves adherence and outcomes in poorly controlled asthma. *Am J Respir Crit Care Med* 2010;**181**:566–77. doi:10.1164/rccm.200906-0907OC

43 Myers RE, Advani SM, Myers P, *et al.* Engaging Patients with Late-Stage Non-Small Cell Lung Cancer in Shared Decision Making about Treatment. *J Pers Med* 2021;**11**. doi:10.3390/JPM11100998

44 Coulter A, Stilwell D, Kryworuchko J, *et al.* A systematic development process for patient decision aids. *BMC Med Inform Decis Mak* 2013;**13**:S2. doi:10.1186/1472-6947-13-S2-S2

45 Stacey D, Légaré F, Boland L, *et al.* 20th Anniversary Ottawa Decision Support Framework: Part 3 Overview of Systematic Reviews and Updated Framework. Med. Decis. Mak. 2020;**40**:379–98. doi:10.1177/0272989X20911870

For peer review only

For peer review only - http://bmjopen.bmj.com/site/about/guidelines.xhtml

46 O'Cathain A, Croot L, Duncan E, *et al.* Guidance on how to develop complex interventions to improve health and healthcare. *BMJ Open* 2019;**9**:e029954. doi:10.1136/bmjopen-2019-029954

47 Mccaffery KJ, Holmes-Rovner M, Smith SK, *et al.* Addressing health literacy in patient decision aids. 2012. doi:10.1186/1472-6947-13-S2-S10

48 Muscat DM, Shepherd HL, Nutbeam D, *et al.* Health Literacy and Shared Decision-making: Exploring the Relationship to Enable Meaningful Patient Engagement in Healthcare. *J Gen Intern Med* 2020;**36**:521–5. doi:10.1007/s11606-020-05912-0

49 Parker RM, Baker DW, Willia M V., *et al.* The test of functional health literacy in adults. *J Gen Intern Med 1995 1010* 1995;**10**:537–41. doi:10.1007/BF02640361

50 Plain English Campaign. http://www.plainenglish.co.uk/ (accessed 6 Aug 2021).

51 Linder SK, Swank PR, Vernon SW, *et al.* Validity of a low literacy version of the Decisional Conflict Scale. *Patient Educ Couns* 2011;**85**:521–4. doi:10.1016/j.pec.2010.12.012

52 Chen Z, Fan VS, Belza B, *et al.* Association between Social Support and Self-Care Behaviors in Adults with Chronic Obstructive Pulmonary Disease. *Ann Am Thorac Soc* 2017;**14**:1419–27. doi:10.1513/AnnalsATS.201701-026OC

53 Lind N, Nordin M, Palmquist E, *et al.* Coping and social support in asthma and allergy: the Västerbotten Environmental Health Study. *J Asthma* 2015;**52**:622–9. doi:10.3109/02770903.2014.991970

54 Janssen DJA, Curtis JR, Au DH, *et al.* Patient-clinician communication about end-of-life care for Dutch and US patients with COPD. *Eur Respir J* 2011;**38**:268–76. doi:10.1183/09031936.00157710

For peer review only

Figure legends

Figure I: MIND-IT model; a framework representing informed, evidence-based, and shared decisions with patients and their healthcare professional (Bekker et al., 2015)

Figure II: PRISMA flow diagram of review process

Figure III: SDM intervention principles aligned to the MIND-IT model (Bekker et al., 2015)

Figure IV: Risk of bias in RCT studies

Figure V: Risk of bias in non-RCT studies

Table I: Summary of studies

Table II: Summary of interventions

For peer review only

For peer review only - http://bmjopen.bmj.com/site/about/guidelines.xhtml

Tables

Table I: Summary of studies

First Author, Year, Country, Sample size, Study design, Population, Healthcare decision	Outcome(s)	Summary of results	Certainty of evidence (GRADE score)
Au, 2012, USA, Clinicians=92 Patients=376, RCT, Outpatients with COPD, end of life care	*Validated:* Quality of communication (QOC). *Non-validated:* Occurrence of discussions about end of life preferences between patients and either clinician or surrogate.	Baseline quality of communication was poor in both groups. Modest improvements in both groups, but significant mean between group difference favouring the intervention group (5.7 points; p<0.05). The occurrence (%) of patient communication about end of life care was significantly higher in the intervention group for three of four questions (mean(SD)): 1. Int:35.2(74.6) Con:15.9(58.2) p<0.05, 2. Int:60.3(122.9) Con:30.8(175.5) p<0.05, 3. Int:53.6(115.1) Con:45.2(73.3) p>0.05, 4. Int:86.2(84.6) Con:75.2(115.0) p<0.05.	⊕⊕⊕◯ Moderate certainty because of risk of bias.
Brundage, 2001, Canada, Patients=20, Feasibility CBA, Outpatients with lung cancer, treatment	*Validated:* Decisional conflict (DCS). *Non-validated:* Knowledge of treatment options.	Decisional conflict reduced after receiving the intervention in 87% and increased in 13% of participants. Knowledge of the available treatment options improved following the intervention for both 3-year outcome survival[a] and median survival outcome[b]: 1. Knowledge of survival outcomes with treatment 1 alone (mean between group difference:60%[a]; 54%[b]), 2. Knowledge of direction of survival difference between treatments (mean between group difference:27%[a]; 40%[b]), 3. Knowledge of the magnitude of survival difference between treatments (mean between group difference:73%[a]; 67%[b]). The number of participants with a treatment preference increased from 80% to 100% following the intervention.	⊕⊕◯◯ Low certainty because of risk of bias and minor publication bias.
Collinsworth, 2018, USA, Patients=308, RCT, Inpatients with COPD, self-management	*Validated:* Patient activation (PAM), Quality of life health status (CAT). *Non-validated:* All-cause hospital admissions.	Both groups had a significant improvement in PAM scores following the intervention (mean(SD)difference:0.52(0.9) and 0.69(1.0) points respectively, p<0.05). Only the intervention group had significantly increased scores in the CAT following the intervention (mean(SD) difference: Int:5.27(10.3), p<0.05; Con:0.38(7.8), p>0.05). There were no significant differences in the total number of all-cause or COPD-related readmissions between groups at 1, 2, 3, 6 and 9 months (p>0.05).	⊕⊕⊕◯ Moderate certainty because of risk of bias.
Gagne, 2017, Canada, Patients=51, RCT, Outpatients with asthma, treatment	*Validated:* Asthma knowledge (QCALF), Decisional conflict. *Non-validated*: Appropriate use of	Both groups had significantly increased knowledge scores following the intervention (mean(SD) difference: Int:3.6(8.9)*, p<0.001; Con:2(7.3)*, p<0.05). There was no significant between group difference (p>0.05). Both groups had significantly reduced decisional conflict scores following the intervention (mean(SD) difference: Int:8.1(22.5)*, p<0.05; Con:9.1(27.7)*, p>0.05). There was no significant between group difference (p>0.05). Both groups had modest, but non-significant, within group improvements in their appropriate use of pharmacotherapy (mean(SD) difference: Int:0.15(0.7)*, p>0.05; Con: 0.16(0.7)*, p>0.05). There was no significant between group difference (mean(SD) between group difference=0.17(0.7), p>0.05).	⊕⊕⊕◯ Moderate certainty because of risk of bias.

For peer review only

For peer review only - http://bmjopen.bmj.com/site/about/guidelines.xhtml

	asthma pharmacotherapy.		
Granados-Santiago, 2019, Spain, Patients=42, RCT, Inpatients with COPD, self-management	*Validated:* Quality of life and health status (EQ-5D), COPD knowledge (COPD-Q), Medication adherence (TAI), Physical activity (steps/day), Nutritional status (MNA). *Non-validated:* -	Both groups had significant, clinically important improvements in quality of life and health status at discharge (mean(SD) between group difference:6.15(27.5)*, p<0.05). Only the intervention group maintained this benefit at 3 months (mean(SD) between group difference:8.28(50.6)*, p<0.05). Knowledge scores significantly improved in the intervention group at discharge (mean(SD) between group difference:3.89(0.7)*, p<0.05) and maintained at 3 months (mean(SD) between group difference:3.88(0.8)*, p<0.05). Adherence significantly improved at discharge in the intervention group (mean(SD) difference: Int:3.58(1.5)*, p<0.05; Con:1.36(1.6)*, p>0.05), but no significant between group difference. At 3 months, there was a significant mean(SD) between group difference favouring the intervention group (1.8(2.4)*, p<0.05). Upon discharge, there was a significant reduction in physical activity in both groups (mean(SD) difference: Int:-348.27(2.0)*, p<0.05; Con:-410.05(4.66)*, p<0.05). At 3 months there was a significant improvement in the intervention group alone (mean(SD) difference: Int:1371.97(895.2)*, p<0.05; Con:620.18(12.5)*, p>0.05). At 3 months there was a significant improvement in nutritional status both within and between groups for the intervention group mean(SD) difference: Int:4.15(0.1)*, p<0.05; Con:-3.43(0.12)*, p>0.05	⊕⊕⊕◯ Moderate certainty because of risk of bias.
Myers, 2021, USA, Patients=5, Pilot CBA, Outpatients with non-small cell lung cancer, treatment	*Validated:* Decisional Conflict (DCS SURE Test). *Non-validated:* Knowledge of treatment options, Treatment status.	Decisional conflict reduced post intervention (mean difference=1). Awareness of treatment options increased from 40% to all 100% participants post intervention. At 30 days post intervention, 80% of participants had made a decision. 40% of participants had a treatment that matched their treatment preference.	⊕⊕◯◯ Low certainty because of risk of bias.
Walters, 2013, Australia, Patients=182, Cluster RCT, Outpatients with COPD, self-management	*Validated:* Quality of life (SF-36)., Quality of life (SGRQ), Patient involvement in healthcare decisions (PIH). *Non-validated:* Respiratory hospital admissions, Intervention fidelity.	Neither group had a significant change to either their physical[a] or mental[b] health status at 6 months (mean(SD) between group difference: 1.5(0.2)*[a]; 0.8(1.4)*[b], p>0.05) and 12 months (mean(SD) between group difference: 0.0(0.9)*[a]; 0.3(0.9)*[b], p>0.05). Neither group had a significant change to their quality of life at 6 months (mean(SD) between group difference: 1.9(2.7)*, p>0.05) and 12 months (mean(SD) between group difference: 1.4(1.5)*, p>0.05. The intervention group had a significant improvement in PIH scores at 6 months (mean(SD) between group difference: 0.4(0.2)*, p<0.05) and 12 months (mean(SD) between group difference: 0.3(0.1)*, p<0.05). No significant change in hospital admissions for either group at 12 months (χ^2=2.61, p>0.05). Fidelity assessments confirmed components were addressed with some clarity: 1. COPD symptom management, 49%, 2. Unhelpful self-talk explored and identified, 37%, 3. Unhelpful self-talk challenged, and new self-talk developed, 35%, 4. Action plan for achieving goals made, 54%, 5. Problems and barriers to achieving goals identified and clarified, 46%, 6. Positive changes in behaviour praised, 83%.	⊕◯◯◯ Very low certainty because of significant risk of bias and some inconsistency.
Wilson, 2010, Hawaii, Patients=612, RCT, Outpatients with asthma, treatment	*Validated:* Quality of life (MiniAQLQ). *Non-validated:* Healthcare use, Patients' perceived role in treatment decision, Medication adherence, Intervention fidelity.	Intervention groups had significant improvements in quality of life at 12 months (SDM to control mean(SD) between group difference= 0.39(1.0)*, p<0.05; SDM–clinician decision-making mean(SD) between group difference=0.11(1.1)*, p>0.05; clinician decision-making-control mean(SD) between group difference=0.28(1.0)*, p<0.05. Intervention groups had significantly reduced healthcare utilisation (SDM-control mean(SD) between group difference=20.36(-1.5)*, p<0.05; SDM-clinician decision-making mean(SD) between group difference=0.01(2.7)*, p>0.05; clinician decision-making-control mean(SD) between group difference=20.37(-1.5*), p<0.05. The SDM group rated their influence on the treatment decision as similar to clinicians' influence (mean(SD)=3.1(0.6). The SDM group ratings were significantly different from the clinician decision-making group, with the latter feeling their clinician had greater influence (mean(SD)=2.5(0.9), p<0.05). Medication adherence significantly increased in intervention groups (SDM-control mean(SD) between group difference=0.21(0.5)*, p<0.05; SDM–clinician decision-making mean(SD) between group difference=0.08(0.4)*, p>0.05; CDM-control mean(SD) between group	⊕⊕⊕◯ Moderate certainty because of risk of bias.

For peer review only - http://bmjopen.bmj.com/site/about/guidelines.xhtml

difference=0.13(0.4)*, p<0.05, but not at 2 years (SDM-control mean(SD) between group difference=0.03(0.4)*, p>0.05; SDM-clinician decision-making mean(SD) between group difference=0.04(0.4)*, p>0.05; clinician decision-making-control mean(SD) between group difference=-0.01(-0.4)*, p>0.05. Protocol adherence was high (SDM=4.0, clinician decision-making=3.9) and did not differ significantly between groups (p>0.05).

For peer review only

For peer review only - http://bmjopen.bmj.com/site/about/guidelines.xhtml

SDM Systematic Review

Table II: Summary of interventions

First Author (Year)	Description of intervention	Interventionist (including any training provided)	Intervention dose	Description of control group
Au (2012)	Consultation using a completed patient-specific feedback form which describes patient preferences regarding end of life care. The feedback form was not endorsed for use.	Clinicians. No specific training described.	Single session.	Routine outpatient COPD care.
Brundage (2001)	Consultation using a patient decision aid to facilitate cancer treatment decision-making.	Researcher. No specific training described.	Single session.	Participant outcomes prior to receipt of intervention.
Collinsworth (2018)	COPD education and SDM self-management planning.	Registered respiratory therapists. No specific training described.	Single session and four telephone calls.	Routine inpatient care and COPD education.
Gagne (2017)	Patient decision aid to facilitate asthma treatment decision-making (and asthma education).	Certified asthma educators from the Quebec Asthma and COPD Network.	Single session.	Routine outpatient asthma care, education, and action plan generation.
Granados-Santiago (2019)	A SDM patient involvement programme focussing on COPD self-management goals to facilitate self-management decision-making.	Healthcare professionals. No specific training described.	Delivered alongside routine inpatient care.	Routine inpatient care for an exacerbation of COPD.
Myers (2021)	Personalised patient decision aid used to facilitate 15-20min decision coaching consultation.	Oncology nurses. No training provided.	Single session.	Participant outcomes prior to receipt of intervention.
Walters (2013)	Psychoeducation about common psychological reactions to COPD diagnosis and treatment, self-management skills training, cognitive coping skills training to identify and challenge negative COPD-related cognitions, communication skills to facilitate discussion between the health mentor and the patient and promoting self-efficacy to manage chronic illness.	Community health nurses, termed 'Health Mentors.' Training included 12 hours of training over 2 days that covered COPD management (1 h), chronic disease self-management and health behaviour change components including practice role plays (7.25 hours), online training and study methods (3.75 hours).	Single session and sixteen telephone calls.	Routine outpatient COPD care and monthly telephone calls for 12 months (excluded intervention components).

For peer review only - http://bmjopen.bmj.com/site/about/guidelines.xhtml

Wilson (2010)	Shared decision-making intervention: Consultation to facilitate asthma treatment decision-making followed by four contacts to assess patient progress and medication changes as needed.	Healthcare professionals involved in routine asthma care (nurses, respiratory therapists, pharmacists, nurse practitioners and physician assistants). Training provided but not described.	Two sessions and 3 telephone calls.	Routine asthma outpatient care (including, in some sites, the opportunity to of referral to an asthma care a management programme).
	Clinician decision-making intervention: Consultation for clinicians to obtains a patient's level of asthma control, prescribe an appropriate treatment regimen and communicate that to the patient.	Healthcare professionals involved in routine asthma care (nurses, respiratory therapists, pharmacists, nurse practitioners and physician assistants). Training provided but not described.	Two sessions and 3 telephone calls.	

For peer review only

For peer review only - http://bmjopen.bmj.com/site/about/guidelines.xhtml

SDM Systematic Review

A systematic review of shared decision-making interventions for people living with chronic respiratory diseases

Authors

Barradell, AC[1-3]; Gerlis, C[3]; Houchen-Wolloff L[1,3]; Bekker, HL[5,6]; Robertson, N[4]; Singh, SJ[1,3].

Author affiliations:

1. Department of Respiratory Sciences, University of Leicester, University Road, Leicester LE1 7RH
2. National Institute for Health Research (NIHR) Applied Research Collaboration (East Midlands), College of Medicine, Biological Sciences & Psychology, Leicester General Hospital, Gwendolen Road, Leicester, LE5 4PW
3. Centre for Exercise and Rehabilitation Science, Leicester Biomedical Research Centre-Respiratory, Glenfield Hospital, Groby Road, Leicester, LE3 9QP
4. Department of Neuroscience, Psychology and Behaviour, University of Leicester, University Road, Leicester LE1 7RH
5. Leeds Unit of Complex Intervention Development (LUICD), School of Medicine, University of Leeds, Leeds, LS2 9JT
6. Research Centre for Patient Involvement, Central Denmark Region and Aarhus University, DK-8000 Aarhus C

Corresponding author:

Name: Amy C Barradell

Address: Centre for Exercise and Rehabilitation Science, Leicester Biomedical Research Centre-Respiratory, Glenfield Hospital, Groby Road, Leicester, LE3 9QP

Email: ab1081@le.ac.uk

Tel: 0116 258 3035

Short running title

A systematic review of SDM interventions in CRD

For peer review only - http://bmjopen.bmj.com/site/about/guidelines.xhtml

Abstract

Objective:

Shared Decision-Making (SDM) supports patients to make informed and value-based decisions about their care. We are developing an intervention to enable healthcare professionals to support patients' PR decision-making. To identify intervention components we needed to evaluate others carried out in Chronic Respiratory Diseases (CRD). We aimed to evaluate the impact of SDM interventions on patient decision-making (primary outcome) and downstream health-related outcomes (secondary outcome).

Design:

We conducted a systematic review using the risk of bias (Cochrane ROB2, ROBINS-I) and certainty of evidence (GRADE) tools.

Data sources:

MEDLINE, EMBASE, PSYCHINFO, CINAHL, PEDRO, Cochrane Central Register of Controlled Trials, the International Clinical Trials Registry Platform Search Portal, ClinicalTrials.gov, PROSPERO, ISRCTN were search through to 14thDecember 2021.

Eligibility criteria:

Trials evaluating SDM interventions in patients living with CRD using quantitative or mixed methods were included.

Data extraction and synthesis:

Two independent reviewers extracted data, assessed risk of bias, and certainty of evidence. A narrative synthesis, with reference to the The Making Informed Decisions Individually and Together (MIND-IT) model, was undertaken.

For peer review only - http://bmjopen.bmj.com/site/about/guidelines.xhtml

Results:

Eight studies [n=1,596 (of 17,466 citations identified)] fulfilled the inclusion criteria.

Five studies included components targeting the patient, healthcare professionals and consultation process (demonstrating adherence to the MIND-IT model). All studies reported their interventions improved patient decision-making and health-related outcomes. No outcome was reported consistently across studies. Four studies had high risk of bias, three had low quality of evidence. Intervention fidelity was reported in two studies.

Conclusions:

These findings suggest developing a SDM intervention including a patient decision aid, healthcare professional training, and a consultation prompt could support patient PR decisions, and health-related outcomes. Using a complex intervention development and evaluation research framework will likely lead to more robust research, and a greater understanding of service needs when integrating the intervention within practice.

Strengths and limitations

- The inclusion of studies from across the chronic respiratory disease population enabled us to draw conclusions about the efficacy of shared decision-making within this population when other reviews of specific respiratory conditions have been unable to.
- The search criteria may have meant we missed studies which used alternative terminology for shared decision making.
- We were unable to perform meta-analysis or sub-group analysis due to the heterogeneity of included studies and their outcome measures.

Medical Subject Headings

Informed decision making

Decision support

Patient-centred care

Professional-patient relations

For peer review only

75 Lung disease

76 Registration:

77 The systematic review protocol was registered on PROSPERO on 8th April 2020 (ID:
78 CRD42020169897).

For peer review only

For peer review only - http://bmjopen.bmj.com/site/about/guidelines.xhtml

Introduction

Patients living with chronic obstructive pulmonary disease (COPD) account for a significant proportion of death and disability worldwide[1,2]. Pulmonary rehabilitation (PR) is a recommended evidence-based intervention to improve the physical and psychological health of this population[3] but patients express significant barriers to accepting PR[4,5]. It is therefore proposed, healthcare professionals should seek to engage patients in informed decisions about their enrolment into a programme[5,6].

Shared Decision-Making (SDM) is a core principle of personalised care, which encourages healthcare professionals to actively engage patients in healthcare decisions[7]. This style of communication requires patients and healthcare professionals to share knowledge about the health condition (i.e., the lived experience and evidence-based treatment), then engage in a period of deliberation to review the pros and cons of each option for the patients' life and agree upon an optimal healthcare choice.

This systematic review will inform the development and evaluation of a SDM intervention enabling healthcare professionals to support patients living with COPD make informed decisions about PR. SDM can be viewed as a complex intervention as it requires engagement from multiple stakeholders to enhance communication within a consultation. The Making Informed Decisions Individually and Together (MIND-IT) model[8] provides a conceptual framework to represent components within a multiple stakeholder decision support intervention and its evaluation (Figure 1). There are a range of interventions currently supporting SDM in consultations, including patient education materials, for example, Patient Decision Aids (PtDAs) with or without decision coaching, and SDM training for healthcare professionals.

PtDAs are resources providing accurate information to help people make informed, value-based decisions between healthcare options[7]. They are informed by decision science research to

For peer review only - http://bmjopen.bmj.com/site/about/guidelines.xhtml

structure the content in a way that reduces the cognitive effort needed to process facts, present balanced and neutral information, and provide prompts encouraging people to trade-off their evaluations of the consequences, and choose an option that is best for their life[9]. The integration of PtDAs into multiple healthcare contexts has shown to improve the frequency of SDM and patient-centred decisions, increase patient knowledge of their condition and the options available, enhance involvement in the decision-making process, and reduce decisional conflict[10–14].

Decision Coaching training for healthcare professionals aims to develop their skills in supporting patients to reason between healthcare options rather than providing information about a medical best treatment plan[15]. The training guides healthcare professionals to adopt a non-directive approach in SDM consultations by supporting patients to consider all available options, preparing them for deliberating over options, and ensuring any chosen option is then implemented. Research suggests decision coaching supports patient engagement in SDM[13,16–18] and in combination with a PtDA, is a robust method to facilitate SDM[16].

For peer review only

SDM training for healthcare professionals aims to help them adopt a SDM approach to delivering care[19]. There is no consensus guiding the content of this training, however NICE offers a training package[19] which includes a range of knowledge and skills-based modules on patient-centred care, presenting risk information and uncertainty, decision and cognitive science, evidence-based medicine, multiple stakeholder collaboration, and consultation skills. The evidence evaluating the effectiveness of this training on SDM use and impact in practice is weak due to the heterogeneity in study designs, methodology, and evaluation strategies[16,17].

SDM interventions are assessed using multiple outcome measures and include: within-consultation communication using healthcare professionals observation (e.g. the Observer OPTION 5 tool[20]) and patient self-report questionnaires (e.g. the Shared Decision-Making Questionnaire[21]), patient knowledge of healthcare options, attitudes, and skills (e.g. self-reported perception of risk, the Values' scale[22], the Decision Self Efficacy Scale[23]), patient decision outcomes (e.g. the choice

For peer review only - http://bmjopen.bmj.com/site/about/guidelines.xhtml

made, the Decisional Conflict Scale[24], the Decision Regret Scale[25]), overall SDM experience from patient and healthcare professionals perspectives (e.g. the iSHARE tool[26]), and downstream patient health-related outcomes (e.g. medication/treatment adherence, healthcare utilisation, disease knowledge, and quality of life measures).

In this systematic review we sought to evaluate the efficacy of SDM interventions compared to usual care in patients living with Chronic Respiratory Disease (CRD). To date, the efficacy of SDM interventions for adults living with CRD is largely unknown. There is a paucity of evidence to suggest patient educational materials (including PtDAs) are beneficial for reducing breathlessness and improving psychological well-being across acute respiratory diseases [27]. Two systematic reviews have been published to synthesise evidence on the impact of SDM interventions on people with asthma[28] and cystic fibrosis[29] but they found the evidence base to be very limited and too heterogeneous to draw meaningful conclusions about the relationship between the interventions and improved SDM or downstream health-related outcomes and nearly all the review population were children and adolescents. Therefore, there is a clear need to evaluate the efficacy of SDM interventions upon adult patients living with Chronic Respiratory Disease (CRD; e.g. COPD, asthma, idiopathic pulmonary fibrosis, lung cancer).

In this review, we aim to identify the components used to support SDM within this health context and synthesise evidence of their impact on patient decision-making and downstream health-related outcomes. We use the Preferred Reporting in Systematic Reviews and Meta-Analyses (PRISMA) guidelines to report this review[30].

(Insert Figure I)

For peer review only - http://bmjopen.bmj.com/site/about/guidelines.xhtml

Methods

Eligibility criteria

Participants

Adults (aged 18+ years) diagnosed with any CRD (and their carers).

Intervention

Interventions which orchestrated SDM between patients and healthcare professionals, with or without disease education, decision coaching training, or SDM training were sought. For the purpose of this review, interventions needed to report a patient-facing component which was designed to facilitate the decision-making process between a patient and clinician during a consultation (e.g. a patient education material).

.Comparison

Any concurrent control groups enrolled in the included studies, but not receiving a SDM intervention.

Outcomes

Primary outcomes of interest included measures of the quality of patient decision-making (e.g. participants' preparedness for decision-making, healthcare professionals and patient involvement in the decision-making process, knowledge of the available options and associated risks, decisional conflict, concordance between the participants' values and the decision made, decision confidence, and decisional regret).

Secondary outcomes of interest were patients' downstream health-related outcomes (e.g. behavioural activation, quality of life, knowledge of condition, healthcare utilisation). Specific qualitative measures of healthcare professionals' attitudes and experience of the SDM intervention, attendance, attrition, and fidelity were also included.

For peer review only - http://bmjopen.bmj.com/site/about/guidelines.xhtml

Study design

Randomised and non-randomised quantitative or mixed method controlled trials were sought for inclusion in this review. This included cluster trials, interrupted time series (ITS) studies with at least one data point before and after the intervention and controlled before-after (CBA) studies.

Exclusions were hypothetical decision scenarios, and studies not reported in English.

Information sources

An electronic search was conducted on the review's inception until 11th April 2023 using the following databases and registries: MEDLINE (1966-), EMBASE (1947-), PSYCHINFO (1887-), CINAHL (1937-), PEDRO (1999-), Cochrane Central Register of Controlled Trials (1993-) the International Clinical Trials Registry Platform Search Portal (2006-), ClinicalTrials.gov (2000-), PROSPERO (2011-), ISRCTN (2000-). Reference lists of selected studies and relevant systematic reviews were also searched to identify further articles for inclusion.

Search strategy

The search algorithm (Supplementary Material 1) was developed and piloted with support from the University Hospitals of Leicester NHS Trust Library Services. It included wide array of chronic respiratory diseases (e.g. tuberculosis, bronchiectasis, idiopathic pulmonary fibrosis). The algorithm was piloted using the MEDLINE database with limits imposed to retrieve articles in English. The algorithm was adapted to the syntax and MESH headings for each database.

Selection process

Articles were uploaded to the web-based platform, Rayaan (https://rayyan.qcri.org/welcome) to facilitate independent review. Duplicate articles were removed and articles reporting findings across multiple outputs were highlighted to review in tandem. Two reviewers (AB, CG) screened titles and abstracts using the inclusion and exclusion criteria. Eligible full texts were reviewed by the same two authors. Any ambiguity in the eligibility of an article was discussed and resolved with the co-authors.

For peer review only

For peer review only - http://bmjopen.bmj.com/site/about/guidelines.xhtml

196 **Data collection process**

197 A review-specific data extraction form was used, informed by Cochrane data extraction forms[31].
198 This was piloted prior to use to ensure consistency in the extraction process. One reviewer (AB)
199 conducted data extraction. A random 10% selection was reviewed by a co-author (CG) to validate
200 the extracted data. Since there were no errors in data extraction were identified, no further
201 validation was considered necessary. Ambiguity in the extracted data was discussed by all authors
202 and resolved by contacting individual study teams. Specific data items for primary and sub-group
203 analysis are outlined in Supplementary Material 2.

204 **Study risk of bias assessment**

205 RCTs were assessed using the Cochrane Risk of Bias tool[32,33] which measures the following
206 domains: randomisation process, deviations from intended interventions, missing outcome data,
207 measurement of the outcomes, and selection of the reported result. Non-RCTs were assessed using
208 the Cochrane ROBINS-I tool[34] which measures the following domains: confounding, selection of
209 study participants, classification of interventions, deviations from intended interventions, missing
210 data, measurement of outcomes, and selection of the reported result. Two authors (AB, CG)
211 independently rated the studies. Any discrepancy in judgements were reviewed and resolved by the
212 co-authors.

213 **Certainty of evidence**

214 The strength of evidence for all reported outcomes was assessed using the Grading of
215 Recommendations Assessment, Development & Evaluation working group methodology (GRADE)
216 which measures the following domains: risk of bias, inconsistency, indirectness, imprecision, and
217 publication bias. Initial judgements were based upon study designs and then systematically reduced
218 if the quality of evidence was poor or increased if a significant effect, dose response or evidence of
219 the elimination of all plausible residual confounding variables and bias was observed. Two authors
220 (AB, CG) independently assessed the criteria. Any discrepancies were resolved with the co-authors.

For peer review only

For peer review only - http://bmjopen.bmj.com/site/about/guidelines.xhtml

Synthesis methods

Statistical analysis of the results was not appropriate due to the clinical and methodological heterogeneity of the outcome measures between studies. A narrative synthesis, with reference to the MIND-IT model, was undertaken to identify the components used to support SDM in CRD and synthesise the evidence of their impact on patient decision-making and downstream health-related outcomes both between and within included studies. Guidance on the conduct of narrative synthesis in systematic reviews [35] was consulted to guide this process.

All outcomes were tabulated with continuous data presented as mean (standard deviation) and categorical data as percentage proportions. To allow consistency in reporting and synthesis of the results, data that was not published as means and standard deviations (e.g. instead reported as means and confidence intervals (CIs)) were transposed to this format using the formula reported in chapter 6 of the Cochrane Handbook for Systematic Reviews of Interventions (Version 6.3, 2022; (Higgins et al., 2022). These are acknowledged with an '*' in Table I.

Patient and Public Involvement

No patient and public involvement occurred during the development of the systematic review's research question, the choice of outcomes measures, the design and implementation of the review, or the interpretation of the results.

Results

Study selection

The search generated 17,488 articles. Following the removal of duplicates, 15,883 articles were retained for abstract screening. Of these, 117 underwent full text screening and eight were retained for inclusion in this analysis (109 excluded). Most studies were excluded because of the participant population (Figure II). Other reasons included study design, type of intervention, and type of publication. A full list of excluded studies is provided in Supplementary Material 3.

For peer review only - http://bmjopen.bmj.com/site/about/guidelines.xhtml

SDM Systematic Review

245 (Insert Figure II)

Study characteristics

Characteristics of the eight included studies[36–43] are provided in Tables I and II (an extended version of Table I is provided in Supplementary Material 4). Six studies were RCTs[36,38–42], two were non-RCT[37,43]. Two studies were conducted with COPD outpatients[36,41], two with COPD inpatients[38,40], two with lung cancer outpatients[37,43], and two with asthma outpatients[39,42]. The theoretical underpinning of interventions, reported by study authors, included social cognitive theory[36], principles of self-management and SDM[38], SDM[39,42,43], self-management[40] and cognitive behavioural psychology[41]. One study[37] did not report a theoretical underpinning. The interventions of five studies included all three components of the MIND-IT multiple stakeholder model (Figure III). Four studies included additional components to SDM within their intervention groups; two included a self-management programme[40,41], and three included an educational component[38,39,41].

(Insert Tables I and II)

(Insert Figure III)

Risk of bias within studies

Two RCTs were judged to have high risk of bias[38,41] and four RCTs were judged to have some concerns [36,39,40,42] (Figure IV). One study was judged as high due to insufficient efforts in randomisation, blinding and attrition[38], the other was due to attrition and poor fidelity[41]. The two non-RCTs were both judged to have serious risk of bias because of insufficient efforts in blinding[37,43] (Figure V).

(Insert Figure IV and V)

For peer review only - http://bmjopen.bmj.com/site/about/guidelines.xhtml

Results of synthesis

Results of individual studies are presented in turn alongside certainty of evidence assessments in Table I.

Decision-making outcomes

Five outcomes captured the quality of patient decision-making (Table I); quality of communication, involvement in decision-making, decisional conflict, strength of treatment preference, and knowledge of treatment options.

Quality of communication

One study reported that quality of communication increased in intervention and control groups, however, a significantly greater increase occurred in the intervention group alone [mean between group difference: 5.7 points; $p<0.05$][36]. The same study reported an increase in the occurrence of patient-centred communication in the intervention group alone, as measured by a study-specific 4-item self-reported questionnaire [mean(SD) Item 1. Intervention: 35.2(74.6) Control: 15.9(58.2) $p<0.05$; Item 2. Intervention: 60.3(122.9) Control: 30.8(175.5) $p<0.05$; Item 3. Intervention: 53.6(115.1) Control: 45.2(73.3) $p>0.05$; Item 4. Intervention: 86. 2(84.6) Control: 75.2(115.0) $p<0.05$].

Involvement in decision-making

Two studies reported significantly increased patient involvement in the decision-making process when compared to a control group [6months mean(SD) between group difference: 0.4(0.2), $p<0.05$; 12 months mean(SD) between group difference: 0.3(0.1), $p<0.05$][41] and a group where clinicians made treatment decisions [immediately post-intervention mean(SD) between group difference: 2.5(0.9), $p<0.05$][42].

Decisional conflict

Two studies (one feasibility, one pilot controlled before and after study) reported a trend for reducing decisional conflict. The first reported 87% reduction in decisional conflict[37], the other reported a mean difference of +1 indicating increased decision certainty[43]. Elsewhere, a RCT

For peer review only - http://bmjopen.bmj.com/site/about/guidelines.xhtml

293 measuring disease education with and without a patient decision aid reported significantly reduced
294 decisional conflict in the intervention and control groups [Intervention mean(SD) difference from
295 baseline: 8.1(22.5), p<0.05; Control mean (SD) difference from baseline: 9.1(27.7), p<0.05], but no
296 significant mean difference between groups [between group mean difference: 2.5, p>0.05)[39].

297 Strength of treatment preference

298 One feasibility study reported the strength of patients' treatment preference increased from 80-
299 100% following exposure to a patient decision aid[37].

300 Knowledge of treatment options

301 One feasibility study reported an increasing trend for treatment specific knowledge for three year
302 outcome survival[a] and median survival outcome[b] [Knowledge of survival outcomes with treatment 1
303 alone (mean between group difference: 60%[a]; 54%[b]); Knowledge of direction of survival difference
304 between treatments (mean between group difference: 27%[a]; 40%[b]); Knowledge of the magnitude of
305 survival difference between treatments (mean between group difference: 73%[a]; 67%[b])][37].

306 A pilot controlled before and after study reported awareness of treatment options increased from
307 40% to 100% of participants post intervention[43].

308 *Health-related outcomes*

309 Six outcomes captured downstream health-related outcomes (Table I); disease knowledge,
310 hospitalisations, behavioural activation quality of life, intervention fidelity, attendance, and attrition.

311 Disease knowledge

312 One study reported significantly increased disease-specific knowledge scores in the intervention
313 group [mean(SD) between group difference: 3.89(0.7), p<0.05] which maintained at 3 months follow
314 up [mean(SD) between group difference: 3.88(0.8), p<0.05][40]. However, another study reported
315 knowledge scores increased equally amongst intervention and control groups [mean difference (SD)
316 Intervention: 3.6(8.9), p<0.05; Control: 2(7.3), p<0.05][39].

For peer review only - http://bmjopen.bmj.com/site/about/guidelines.xhtml

Hospitalisations

Two studies reported no change in all-cause hospitalisations between intervention and control groups[38,41]. One study observed a significant reduction in healthcare utilisation in the SDM and clinician decision-making groups but not in the control group [SDM-control mean(SD) between group difference: 20.36(1.5), $p<0.05$; SDM-clinician decision-making mean(SD) between group difference: 0.01(2.7), $p>0.05$; clinician decision-making-control mean(SD) between group difference: 20.37(1.5), $p<0.05$][42].

Behavioural activation

One feasibility study showed both the intervention and control group had a trend for increased levels of activation post intervention [mean(SD) between group difference: 0.52(0.9) and 0.69(1.0) points respectively, $p<0.05$][38].

One pilot controlled before and after study reported 80% of participants had made a treatment decision at 30 days and 40% had begun a treatment which matched their treatment preference[43].

Two studies reported medication adherence significantly improved in the intervention groups alone[40,42]. However, the control group receiving a clinician decision-making intervention in one of these studies[42] also showed significantly adherent medication behaviour and no between group difference [SDM–clinician decision-making mean(SD) between group difference: 0.08(0.4), $p>0.05$].

One study measured the correct administration of medication and post intervention reported non-significant improvements in the correct use of asthma medication in the intervention and the control group with no significant difference between groups [mean(SD) between group difference: 0.17(0.7), $p>0.05$][39].

One study reported significant between group differences for improvements in nutritional behaviour at 3 months post inpatient intervention initiation [mean between group difference: 5.03; $p<0.05$) and physical activity at 3 months post intervention initiation (mean between group difference: 716.01; $p<0.05$][40].

For peer review only

For peer review only - http://bmjopen.bmj.com/site/about/guidelines.xhtml

Quality of life

Two studies reported no significant difference between intervention and control groups post intervention [Discharge mean(SD) between group difference: 6.15(27.5), $p>0.05$); 6 months mean(SD) between group difference: 1.9(2.7), $p>0.05$); 12 months mean(SD) between group difference: 1.4(1.5), $p>0.05$][40,41]. However, one of the studies did at 3 months post discharge [mean(SD) between group difference: 8.28(50.6), $p<0.05$][40]. Whilst two further studies reported either a trend for improvement [mean(SD) between group difference: 4.89; $p<0.05$)[38] or a significant improvement in the intervention group alone [SDM to control mean(SD) between group difference: 0.39(1.0), $p<0.05$][42].

Intervention fidelity

Only two studies reported intervention fidelity explicitly[41,42]. Both studies rated a specified selection of intervention audio recordings. One reported high adherence to the intervention protocol [SDM, 4/5; clinician decision-making, 3.9/5][42]. The other reported wide variation in adherence between the different intervention components, with lower delivery of cognitive-behavioural components which were described as the backbone of the intervention[41].

Attendance and attrition

For inventions involving single sessions, there was no attrition during intervention delivery[36,37,39,43]. For interventions involving multiple sessions, completion of inpatient interventions ranged from 37%[38] to 100%[40], whereas, outpatient interventions ranged from 72%[41] to 75.5%[42].

Results from sub-group analysis are provided in Supplementary Material 5a and 5b.

Discussion

This review provides the first synthesis of studies evaluating interventions to support SDM between patients with CRD and healthcare professionals. All eight studies reported their interventions improved patient decision-making and downstream health-related outcomes. There were improvements in the quality of communication with healthcare professionals[36], the occurrence of

For peer review only

patient-centred communication[36], involvement in decision-making[41,42], decisional conflict[39,43], health-related knowledge[40], behavioural activation[40,42,43], quality of life[40,42], and healthcare utilisation[42]. There was no one consistent outcome measure used in all studies to enable comparison across studies. Additionally, three studies measured intervention feasibility meaning they were unable to observe intervention efficacy[37,38,43], two RCTs were judged to have a high risk of bias [38,41] and two non-RCTs were judged to have a serious risk of bias [37,43].The most consistently reported outcomes were the downstream health-related outcomes behavioural activation and quality of life. Five studies reported improvements in behavioural activation across six outcome measures[38–40,42,43]. Between group differences were only observed for two of the outcomes (nutritional status and physical activity) and this was in just one study[40]. Two studies reported improved health-related quality of life post intervention[38,42] but one of these was a feasibility study[38].

Unlike other reviews in the CRD field[28,29], this review found variation in the intervention components. Our appraisal showed that only five studies contained all active SDM components from the MIND-IT model (Figure III). Gagne's[39] and Au's[36] study did not report a period of deliberation between patients and healthcare professionals, suggesting the decision-making process was not shared, and in Walters[41] study, there was no report of patients and healthcare professionals exchanging personal and evidence-based information suggesting the decision-making process was not informed. Without adequate inclusion of all SDM components, it is difficult to conclude which intervention components support which outcomes.

Furthermore, there was little evidence in the descriptions of the interventions to show they had been developed in-line with recommended SDM intervention and user-centred design guidelines (e.g. [44],[45] and [46]). There was also limited evidence to suggest included studies had identified their target populations' (e.g. patient or healthcare professionals) needs when developing their intervention and its evaluation. For example, despite the vast array of participants' educational

For peer review only - http://bmjopen.bmj.com/site/about/guidelines.xhtml

attainment across studies, there was little evidence studies observed this and tailored the SDM materials accordingly. This observation is of concern because lower health literacy has been shown to affect key decision-making outcomes[47]. A recent expanded model of SDM has been proposed to reflect the necessity of patients' health literacy skills as this enables them to have meaningful engagement in the decision-making process[48]. In our review, no study had considered the health literacy of their participants prior to enrolment through the use of validated measures (e.g. test of functional health literacy in adults[49]), nor the use of a standardising health literacy resources such as the Plain English Campaign[50] to ensure materials were at the appropriate comprehension level and, finally, no study had considered using the low literacy version of the Decisional Conflict Scale[51], despite its ease of access. Furthermore, no study reported healthcare professional-directed intervention components (e.g. healthcare professional training) or used a user-centred design or implementation process.

Additionally, studies made little reference to social or familial support in the decision-making process, which is unusual as social support is often associated with improvements in self-management behaviour amongst patients with CRD[52,53]. Social support may contribute to informed information exchange and provide confidence in the decision-making process. Interestingly, one study specifically excluded family members or friends from the SDM consultation as they believed it would introduce bias[37].

Limitations of the evidence included in the review

The interpretation of these results should be considered in light of the inclusion of studies which utilised non-validated outcome measures. Only one study used a dose-matched intervention in the control group[41] and fidelity was only reported explicitly in two studies[41,42].

Limitations of the review process

The inconsistent outcome measures within this review precluded meta-analysis. Four studies had a high risk of bias[37,38,41,43] and three had low or very low quality of evidence[37,41,43], thereby

For peer review only - http://bmjopen.bmj.com/site/about/guidelines.xhtml

limiting the validity and generalisability of the results. The search criteria were broad, however, if studies had used alternate terminology for SDM, it is possible some may have been missed. The multi-component interventions meant intervention effects could not be discernibly attributed to the SDM component or the education and self-management components. Furthermore, two studies both provided their control groups with disease education which may have driven the positive effects observed by the control groups[38,39]. Included studies encompassed a variety of healthcare settings with decisions varying in focus from self-management to treatment decision-making and end of life care. Despite excluding studies involving hypothetical decision-making to reduce the disparity in importance attributed to the decision-making process, we cannot assume participants attributed the same importance to all decisions. Additionally, the studies length of follow-ups varied considerably, however, as the type of decisions were so varied the allocated follow-up period may have been appropriate. For example, Janssen and colleagues showed that patients with COPD change their mind about end of life care throughout the course of their healthcare journey[54], suggesting a long-term follow-up period for end of life decision-making is appropriate. Whereas health decisions with more immediate affect (e.g. acceptance of a referral to pulmonary rehabilitation) may require a shorter follow-up period. As more SDM intervention studies are conducted in CRD, an updated review may well benefit from statistical sub-group analysis for studies length of follow up and/or decision posed. Additionally, the decision to exclude qualitative and cross-sectional studies may have limited our ability to fully explore participants' experiences of the SDM process and the development and implementation of interventions.

Implications of the results

This synthesis provides some evidence that CRD SDM interventions improve the patient decision-making process and downstream health-related outcomes. However, the evidence is weak, and the interventions at times incongruent with all active SDM components. Therefore, this limits the rigour

For peer review only - http://bmjopen.bmj.com/site/about/guidelines.xhtml

in evaluating the impact of SDM interventions within CRD. There were also no studies exploring the efficacy of SDM in PR decision-making and implementation in practice.

Based on the available evidence, this review suggests the development of a SDM intervention to enable healthcare professionals to support COPD patients informed decision-making for PR may be beneficial. It recommends that such intervention should be evaluated within a complex intervention development and evaluation research framework. This is especially relevant given the rise in alternate PR delivery models due to the Covid-19 pandemic. Importantly, this intervention should be underpinned by SDM theoretical models (e.g. MIND-IT[8], extended SDM model involving consideration of health literacy[48]), adhere to the appropriate intervention development guidelines (e.g.[44–46]), and be evaluated in a single or double-blind RCT with dose-matched controls.

Other information

Protocol and registration

This review was conducted in accordance with the Preferred Reporting in Systematic Reviews and Meta-Analyses (PRISMA) and registered on PROSPERO (registration number: CRD42020169897). The completed PRISMA checklist was provided at article submission.

Funding statement

The National Institute for Health Research (NIHR Applied Research Collaboration [East Midlands] (NIHR ARC East Midlands) and the Centre for Exercise and Rehabilitation Science (CERS) funded this research. Award/Grant number is not applicable.

Competing interests

The authors have no conflicts of interest to declare.

For peer review only

For peer review only - http://bmjopen.bmj.com/site/about/guidelines.xhtml

Availability of data, code, and other materials

The MEDLINE search strategy, data items sought, methods for sub-group analysis, characteristics of excluded studies, and results of sub-group analysis are provided in the Supplementary Materials. No additional data available.

Acknowledgements

The authors would like to thank the UHL Library Services for their contribution to the search criteria and piloting the search. We would like to thank all study authors for sharing their data, when requested. Professor Singh is a National Institute for Health Research (NIHR) Senior Investigator. The views expressed in this article are those of the author(s) and not necessarily those of the NIHR, or the Department of Health and Social Care.

Contributorship statement

ACB conceived the review. ACB, CG, LHW, HLB, NR and SJS contributed the planning, design, and protocol development. ACB conducted the search in collaboration with the University Hospitals of Leicester NHS Trust Librarian. ACB and CG screened all citations. ACB contacted study authors for additional data acquisition. ACB, CG, LHW, NR and SJS confirmed studies to be included. ACB conducted data extraction and CG conducted a 10% data quality check to ensure accuracy in this process. ACB and CG conducted risk of bias and certainty of evidence assessments. ACB conducted the narrative synthesis and ACB, CG, LHW, NR and SJS contributed to interpretation of the results. ACB drafted the manuscript, and CG, LHW, NR and SJS critically revised it for significant intellectual content and insight. In addition, ACB, CG, LHW, NR and SJS gave final approval of the manuscript version for publication. ACB, CG, LHW, NR and SJS are responsible for the overall content as guarantors. ACB, CG, LHW, NR and SJS report no conflicts of interest.

For peer review only - http://bmjopen.bmj.com/site/about/guidelines.xhtml

Disclosure statement

The National Institute for Health Research (NIHR Applied Research Collaboration [East Midlands] (NIHR ARC East Midlands) and the Centre for Exercise and Rehabilitation Science (CERS) funded this research. Award/Grant number is not applicable. The funders had no role in the inception, data extraction or analysis phases of this review. The views expressed in this article do not communicate an official position of the University of Leicester, NIHR, CERS, University of Leeds, or Aarhus University.

Ethics Approval Statement

This study does not involve human participants. Research Ethics approval was not warranted.

References

1 Lozano R, Naghavi M, Foreman K, *et al.* Global and regional mortality from 235 causes of death for 20 age groups in 1990 and 2010: A systematic analysis for the Global Burden of Disease Study 2010. *Lancet* 2012;**380**:2095–128. doi:10.1016/S0140-6736(12)61728-0

2 Vos T, Flaxman AD, Naghavi M, *et al.* Years lived with disability (YLDs) for 1160 sequelae of 289 diseases and injuries 1990-2010: A systematic analysis for the Global Burden of Disease Study 2010. *Lancet* 2012;**380**:2163–96. doi:10.1016/S0140-6736(12)61729-2

3 Mccarthy B, Casey D, Devane D, *et al.* Pulmonary rehabilitation for chronic obstructive pulmonary disease. Cochrane Database Syst. Rev. 2015;**23**. doi:10.1002/14651858.CD003793.pub3

4 Keating A, Lee A, Holland AE. What prevents people with chronic obstructive pulmonary disease from attending pulmonary rehabilitation? A systematic review. Chron. Respir. Dis. 2011;**8**:89–99. doi:10.1177/1479972310393756

5 Keating A, Lee AL, Holland AE. Lack of perceived benefit and inadequate transport influence

For peer review only

For peer review only - http://bmjopen.bmj.com/site/about/guidelines.xhtml

uptake and completion of pulmonary rehabilitation in people with chronic obstructive pulmonary disease: A qualitative study. *J Physiother* 2011;**57**:183–90. doi:10.1016/S1836-9553(11)70040-6

6 Blackstock FC, Wallack RZ, Nici L, *et al.* Why don't our patients with chronic obstructive pulmonary disease listen to us? the enigma of nonadherence. Ann. Am. Thorac. Soc. 2016;**13**:317–23. doi:10.1513/AnnalsATS.201509-600PS

7 NICE. Shared decision making | NICE guidelines | NICE guidance | Our programmes | What we do | About | NICE. NICE 2021. https://www.nice.org.uk/about/what-we-do/our-programmes/nice-guidance/nice-guidelines/shared-decision-making (accessed 7 Feb 2022).

8 Breckenridge K, Bekker HL, Gibbons E, *et al.* How to routinely collect data on patient-reported outcome and experience measures in renal registries in Europe: An expert consensus meeting. Nephrol. Dial. Transplant. 2015;**30**:1605–14. doi:10.1093/ndt/gfv209

9 Bekker H, Thornton J, Airey C, *et al.* Informed decision making: An annotated bibliography and systematic review. Health Technol. Assess. (Rockv). 1999;**3**. doi:10.3310/hta3010

10 Stacey D, Bennett CL, Barry MJ, *et al.* Decision aids for people facing health treatment or screening decisions. In: Stacey D, ed. *Cochrane Database of Systematic Reviews*. Chichester, UK: : John Wiley & Sons, Ltd 2011. CD001431. doi:10.1002/14651858.cd001431.pub3

11 Stacey D, Légaré F, Lewis K, *et al.* Decision aids for people facing health treatment or screening decisions. Cochrane Database Syst. Rev. 2017;**2017**. doi:10.1002/14651858.CD001431.pub5

12 Hoffman AS, Volk RJ, Saarimaki A, *et al.* Delivering patient decision aids on the Internet: Definitions, theories, current evidence, and emerging research areas. BMC Med. Inform. Decis. Mak. 2013;**13**:S13. doi:10.1186/1472-6947-13-S2-S13

For peer review only - http://bmjopen.bmj.com/site/about/guidelines.xhtml

13 Sepucha KR, Borkhoff CM, Lally J, *et al.* Establishing the effectiveness of patient decision aids: Key constructs and measurement instruments. *BMC Med Inform Decis Mak* 2013;**13**:S12. doi:10.1186/1472-6947-13-S2-S12

14 Feldman-Stewart D, O'Brien MA, Clayman ML, *et al.* Providing information about options in patient decision aids. *BMC Med Inform Decis Mak* 2013;**13**:S4. doi:10.1186/1472-6947-13-S2-S4

15 Jull J, Köpke S, Smith M, *et al.* Decision coaching for people making healthcare decisions. *Cochrane Database Syst Rev* 2021;:CD013385. doi:10.1002/14651858.CD013385.PUB2/MEDIA/CDSR/CD013385/IMAGE_N/NCD013385-CMP-004.01.SVG

16 Légaré F, Ratté S, Stacey D, *et al.* Interventions for improving the adoption of shared decision making by healthcare professionals. In: *Cochrane Database of Systematic Reviews*. John Wiley & Sons, Ltd 2010. doi:10.1002/14651858.cd006732.pub2

17 Müller E, Diesing A, Rosahl A, *et al.* Evaluation of a shared decision-making communication skills training for physicians treating patients with asthma: A mixed methods study using simulated patients. *BMC Health Serv Res* 2019;**19**:612. doi:10.1186/s12913-019-4445-y

18 Stacey D, Kryworuchko J, Belkora J, *et al.* Coaching and guidance with patient decision aids: A review of theoretical and empirical evidence. *BMC Med Inform Decis Mak* 2013;**13**. doi:10.1186/1472-6947-13-S2-S11

19 NICE. Shared decision making learning package. 2021.https://www.nice.org.uk/guidance/ng197/resources/shared-decision-making-learning-package-9142488109 (accessed 7 Feb 2022).

20 Elwyn G, Tsulukidze M, Edwards A, *et al.* Using a 'talk' model of shared decision making to

For peer review only

For peer review only - http://bmjopen.bmj.com/site/about/guidelines.xhtml

propose an observation-based measure: Observer OPTION5 Item. *Patient Educ Couns* 2013;**93**:265–71. doi:10.1016/j.pec.2013.08.005

21 Simon D, Schorr G, Wirtz M, *et al.* Development and first validation of the shared decision-making questionnaire (SDM-Q). *Patient Educ Couns* 2006;**63**:319–27. doi:10.1016/j.pec.2006.04.012

22 O'Connor A. Sample Tool: Values (Tamoxifen). 1999.www.ohri.ca/decisionaid (accessed 19 Feb 2020).

23 O'Connor AM. Sample Tool: Decision Self-Efficacy Scale. 1995.

24 O'Connor AM. Validation of a Decisional Conflict Scale. *Med Decis Mak* 1995;**15**:25–30. doi:10.1177/0272989X9501500105

25 Brehaut JC, O'Connor AM, Wood TJ, Hack TF, Siminoff L, Gordon E F-SD. Validation of a decision regret scale. *Med Decis Making* 2003;**23**:281–92.

26 Bomhof-Roordink H, Gärtner FR, van Duijn-Bakker N, *et al.* Measuring shared decision making in oncology: Development and first testing of the iSHAREpatient and iSHAREphysician questionnaires. *Heal Expect* 2020;**23**:496–508. doi:10.1111/HEX.13015

27 Sunjaya AP, Bao L, Martin A, *et al.* Systematic review of effectiveness and quality assessment of patient education materials and decision aids for breathlessness. *BMC Pulm Med* 2022;**22**:1–14. doi:10.1186/S12890-022-02032-9/FIGURES/5

28 Kew KM, Malik P, Aniruddhan K NR. Shared decision-making for people with asthma. *Cochrane Database Syst Rev* 2017.

29 Malone H, Biggar S, Javadpour S, *et al.* Interventions for promoting participation in shared decision-making for children and adolescents with cystic fibrosis. *Cochrane Database Syst Rev*

For peer review only

For peer review only - http://bmjopen.bmj.com/site/about/guidelines.xhtml

2019;**2019**. doi:10.1002/14651858.CD012578.pub2

30 Page MJ, McKenzie JE, Bossuyt PM, *et al.* The PRISMA 2020 statement: An updated guideline for reporting systematic reviews. BMJ. 2021;**372**. doi:10.1136/bmj.n71

31 Cochrane. Data extraction forms | Cochrane Developmental, Psychosocial and Learning Problems. 2021.https://dplp.cochrane.org/data-extraction-forms (accessed 16 Mar 2021).

32 Sterne JAC, Savović J, Page MJ, *et al.* RoB 2: A revised tool for assessing risk of bias in randomised trials. *BMJ* 2019;**366**:l4898. doi:10.1136/bmj.l4898

33 Higgins J, Thomas J, Chandler J, *et al.* Cochrane Handbook for Systematic Reviews of Interventions version 6.3 (updated February 2022). 2022. Available from www.training.cochrane.org/handbook.

34 Sterne JA, Hernán MA, Reeves BC, *et al.* ROBINS-I: a tool for assessing risk of bias in non-randomised studies of interventions. *BMJ* 2016;**355**. doi:10.1136/BMJ.I4919

35 Popay J, Roberts H, Sowden A, *et al.* Guidance on the conduct of narrative synthesis in systematic reviews. A product from the ESRC methods programme. Version, 1.No Title. 2006. doi:10.13140/2.1.1018.4643

36 Au DH, Udris EM, Engelberg RA, *et al.* A randomized trial to improve communication about end-of-life care among patients with COPD. *Chest* 2012;**141**:726–35. doi:10.1378/chest.11-0362

37 Brundage MD, Feldman-Stewart D, Cosby R, *et al.* Phase I study of a decision aid for patients with locally advanced non-small-cell lung cancer. *J Clin Oncol* 2001;**19**:1326–35. doi:10.1200/JCO.2001.19.5.1326

38 Collinsworth AW, Brown RM, Sjames C, *et al.* The impact of patient education and shared

For peer review only

For peer review only - http://bmjopen.bmj.com/site/about/guidelines.xhtml

decision making on hospital readmissions for COPD. *Int J COPD* 2018;**13**:1325–32. doi:10.2147/COPD.S154414

39 Gagné ME, Légaré F, Moisan J, *et al.* Impact of Adding a Decision Aid to Patient Education in Adults with Asthma: A Randomized Clinical Trial. *PLoS One* 2017;**12**:e0170055. doi:10.1371/journal.pone.0170055

40 Granados-Santiago M, Valenza MC, López-López L, *et al.* Shared decision-making and patient engagement program during acute exacerbation of COPD hospitalization: A randomized control trial. *Patient Educ Couns* 2019;**103**:702–8. doi:10.1016/j.pec.2019.12.004

41 Walters J, Cameron-Tucker H, Wills K, *et al.* Effects of telephone health mentoring in community-recruited chronic obstructive pulmonary disease on self-management capacity, quality of life and psychological morbidity: A randomised controlled trial. *BMJ Open* 2013;**3**. doi:10.1136/bmjopen-2013-003097

42 Wilson SR, Strub P, Buist AS, *et al.* Shared treatment decision making improves adherence and outcomes in poorly controlled asthma. *Am J Respir Crit Care Med* 2010;**181**:566–77. doi:10.1164/rccm.200906-0907OC

43 Myers RE, Advani SM, Myers P, *et al.* Engaging Patients with Late-Stage Non-Small Cell Lung Cancer in Shared Decision Making about Treatment. *J Pers Med* 2021;**11**. doi:10.3390/JPM11100998

44 Coulter A, Stilwell D, Kryworuchko J, *et al.* A systematic development process for patient decision aids. *BMC Med Inform Decis Mak* 2013;**13**:S2. doi:10.1186/1472-6947-13-S2-S2

45 Stacey D, Légaré F, Boland L, *et al.* 20th Anniversary Ottawa Decision Support Framework: Part 3 Overview of Systematic Reviews and Updated Framework. Med. Decis. Mak. 2020;**40**:379–98. doi:10.1177/0272989X20911870

For peer review only

46 O'Cathain A, Croot L, Duncan E, *et al.* Guidance on how to develop complex interventions to improve health and healthcare. *BMJ Open* 2019;**9**:e029954. doi:10.1136/bmjopen-2019-029954

47 Mccaffery KJ, Holmes-Rovner M, Smith SK, *et al.* Addressing health literacy in patient decision aids. 2012. doi:10.1186/1472-6947-13-S2-S10

48 Muscat DM, Shepherd HL, Nutbeam D, *et al.* Health Literacy and Shared Decision-making: Exploring the Relationship to Enable Meaningful Patient Engagement in Healthcare. *J Gen Intern Med* 2020;**36**:521–5. doi:10.1007/s11606-020-05912-0

49 Parker RM, Baker DW, Willia M V., *et al.* The test of functional health literacy in adults. *J Gen Intern Med 1995 1010* 1995;**10**:537–41. doi:10.1007/BF02640361

50 Plain English Campaign. http://www.plainenglish.co.uk/ (accessed 6 Aug 2021).

51 Linder SK, Swank PR, Vernon SW, *et al.* Validity of a low literacy version of the Decisional Conflict Scale. *Patient Educ Couns* 2011;**85**:521–4. doi:10.1016/j.pec.2010.12.012

52 Chen Z, Fan VS, Belza B, *et al.* Association between Social Support and Self-Care Behaviors in Adults with Chronic Obstructive Pulmonary Disease. *Ann Am Thorac Soc* 2017;**14**:1419–27. doi:10.1513/AnnalsATS.201701-026OC

53 Lind N, Nordin M, Palmquist E, *et al.* Coping and social support in asthma and allergy: the Västerbotten Environmental Health Study. *J Asthma* 2015;**52**:622–9. doi:10.3109/02770903.2014.991970

54 Janssen DJA, Curtis JR, Au DH, *et al.* Patient-clinician communication about end-of-life care for Dutch and US patients with COPD. *Eur Respir J* 2011;**38**:268–76. doi:10.1183/09031936.00157710

For peer review only

For peer review only - http://bmjopen.bmj.com/site/about/guidelines.xhtml

Figure legends

Figure I: MIND-IT model; a framework representing informed, evidence-based, and shared decisions with patients and their healthcare professional (Bekker et al., 2015)

Figure II: PRISMA flow diagram of review process

Figure III: SDM intervention principles aligned to the MIND-IT model (Bekker et al., 2015)

Figure IV: Risk of bias in RCT studies

Figure V: Risk of bias in non-RCT studies

Table I: Summary of studies

Table II: Summary of interventions

For peer review only

For peer review only - http://bmjopen.bmj.com/site/about/guidelines.xhtml

SDM Systematic Review

Tables

Table I: Summary of studies

First Author, Year, Country, Sample size, Study design, Population, Healthcare decision	Outcome(s)	Summary of results	Certainty of evidence (GRADE score)
Au, 2012, USA, Clinicians=92 Patients=376, RCT, Outpatients with COPD, end of life care	*Validated:* Quality of communication (QOC). *Non-validated:* Occurrence of discussions about end of life preferences between patients and either clinician or surrogate.	Baseline quality of communication was poor in both groups. Modest improvements in both groups, but significant mean between group difference favouring the intervention group (5.7 points; p<0.05). The occurrence (%) of patient communication about end of life care was significantly higher in the intervention group for three of four questions (mean(SD)): 1. Int:35.2(74.6) Con:15.9(58.2) p<0.05, 2. Int:60.3(122.9) Con:30.8(175.5) p<0.05, 3. Int:53.6(115.1) Con:45.2(73.3) p>0.05, 4. Int:86.2(84.6) Con:75.2(115.0) p<0.05.	⊕⊕⊕◯ Moderate certainty because of risk of bias.
Brundage, 2001, Canada, Patients=20, Feasibility CBA, Outpatients with lung cancer, treatment	*Validated:* Decisional conflict (DCS). *Non-validated:* Knowledge of treatment options.	Decisional conflict reduced after receiving the intervention in 87% and increased in 13% of participants. Knowledge of the available treatment options improved following the intervention for both 3-year outcome survival[a] and median survival outcome[b]: 1. Knowledge of survival outcomes with treatment 1 alone (mean between group difference:60%[a]; 54%[b]), 2. Knowledge of direction of survival difference between treatments (mean between group difference:27%[a]; 40%[b]), 3. Knowledge of the magnitude of survival difference between treatments (mean between group difference:73%[a]; 67%[b]). The number of participants with a treatment preference increased from 80% to 100% following the intervention.	⊕⊕◯◯ Low certainty because of risk of bias and minor publication bias.
Collinsworth, 2018, USA, Patients=308, RCT, Inpatients with COPD, self-management	*Validated:* Patient activation (PAM), Quality of life health status (CAT). *Non-validated:* All-cause hospital admissions.	Both groups had a significant improvement in PAM scores following the intervention (mean(SD)difference:0.52(0.9) and 0.69(1.0) points respectively, p<0.05). Only the intervention group had significantly increased scores in the CAT following the intervention (mean(SD) difference: Int:5.27(10.3), p<0.05; Con:0.38(7.8), p>0.05). There were no significant differences in the total number of all-cause or COPD-related readmissions between groups at 1, 2, 3, 6 and 9 months (p>0.05).	⊕⊕⊕◯ Moderate certainty because of risk of bias.
Gagne, 2017, Canada, Patients=51, RCT, Outpatients with asthma, treatment	*Validated:* Asthma knowledge (QCALF), Decisional conflict. *Non-validated*: Appropriate use of	Both groups had significantly increased knowledge scores following the intervention (mean(SD) difference: Int:3.6(8.9)*, p<0.001; Con:2(7.3)*, p<0.05). There was no significant between group difference (p>0.05). Both groups had significantly reduced decisional conflict scores following the intervention (mean(SD) difference: Int:8.1(22.5)*, p<0.05; Con:9.1(27.7)*, p>0.05). There was no significant between group difference (p>0.05). Both groups had modest, but non-significant, within group improvements in their appropriate use of pharmacotherapy (mean(SD) difference: Int:0.15(0.7)*, p>0.05; Con: 0.16(0.7)*, p>0.05). There was no significant between group difference (mean(SD) between group difference=0.17(0.7), p>0.05).	⊕⊕⊕◯ Moderate certainty because of risk of bias.

For peer review only

For peer review only - http://bmjopen.bmj.com/site/about/guidelines.xhtml

	asthma pharmacotherapy.		
Granados-Santiago, 2019, Spain, Patients=42, RCT, Inpatients with COPD, self-management	*Validated:* Quality of life and health status (EQ-5D), COPD knowledge (COPD-Q), Medication adherence (TAI), Physical activity (steps/day), Nutritional status (MNA). *Non-validated:* -	Both groups had significant, clinically important improvements in quality of life and health status at discharge (mean(SD) between group difference:6.15(27.5)*, p<0.05). Only the intervention group maintained this benefit at 3 months (mean(SD) between group difference:8.28(50.6)*, p<0.05). Knowledge scores significantly improved in the intervention group at discharge (mean(SD) between group difference:3.89(0.7)*, p<0.05) and maintained at 3 months (mean(SD) between group difference:3.88(0.8)*, p<0.05). Adherence significantly improved at discharge in the intervention group (mean(SD) difference: Int:3.58(1.5)*, p<0.05; Con:1.36(1.6)*, p>0.05), but no significant between group difference. At 3 months, there was a significant mean(SD) between group difference favouring the intervention group (1.8(2.4)*, p<0.05). Upon discharge, there was a significant reduction in physical activity in both groups (mean(SD) difference: Int:-348.27(2.0)*, p<0.05; Con:-410.05(4.66)*, p<0.05). At 3 months there was a significant improvement in the intervention group alone (mean(SD) difference: Int:1371.97(895.2)*, p<0.05; Con:620.18(12.5)*, p>0.05). At 3 months there was a significant improvement in nutritional status both within and between groups for the intervention group mean(SD) difference: Int:4.15(0.1)*, p<0.05; Con:-3.43(0.12)*, p>0.05	⊕⊕⊕◯ Moderate certainty because of risk of bias.
Myers, 2021, USA, Patients=5, Pilot CBA, Outpatients with non-small cell lung cancer, treatment	*Validated:* Decisional Conflict (DCS SURE Test). *Non-validated:* Knowledge of treatment options, Treatment status.	Decisional conflict reduced post intervention (mean difference=1). Awareness of treatment options increased from 40% to all 100% participants post intervention. At 30 days post intervention, 80% of participants had made a decision. 40% of participants had a treatment that matched their treatment preference.	⊕⊕◯◯ Low certainty because of risk of bias.
Walters, 2013, Australia, Patients=182, Cluster RCT, Outpatients with COPD, self-management	*Validated:* Quality of life (SF-36)., Quality of life (SGRQ), Patient involvement in healthcare decisions (PIH). *Non-validated:* Respiratory hospital admissions, Intervention fidelity.	Neither group had a significant change to either their physical[a] or mental[b] health status at 6 months (mean(SD) between group difference: 1.5(0.2)*[a]; 0.8(1.4)*[b], p>0.05) and 12 months (mean(SD) between group difference: 0.0(0.9)*[a]; 0.3(0.9)*[b], p>0.05). Neither group had a significant change to their quality of life at 6 months (mean(SD) between group difference: 1.9(2.7)*, p>0.05) and 12 months (mean(SD) between group difference: 1.4(1.5)*, p>0.05. The intervention group had a significant improvement in PIH scores at 6 months (mean(SD) between group difference: 0.4(0.2)*, p<0.05) and 12 months (mean(SD) between group difference: 0.3(0.1)*, p<0.05). No significant change in hospital admissions for either group at 12 months (χ^2=2.61, p>0.05). Fidelity assessments confirmed components were addressed with some clarity: 1. COPD symptom management, 49%, 2. Unhelpful self-talk explored and identified, 37%, 3. Unhelpful self-talk challenged, and new self-talk developed, 35%, 4. Action plan for achieving goals made, 54%, 5. Problems and barriers to achieving goals identified and clarified, 46%, 6. Positive changes in behaviour praised, 83%.	⊕◯◯◯ Very low certainty because of significant risk of bias and some inconsistency.
Wilson, 2010, Hawaii, Patients=612, RCT, Outpatients with asthma, treatment	*Validated:* Quality of life (MiniAQLQ). *Non-validated:* Healthcare use, Patients' perceived role in treatment decision, Medication adherence, Intervention fidelity.	Intervention groups had significant improvements in quality of life at 12 months (SDM to control mean(SD) between group difference= 0.39(1.0)*, p<0.05; SDM–clinician decision-making mean(SD) between group difference=0.11(1.1)*, p>0.05; clinician decision-making-control mean(SD) between group difference=0.28(1.0)*, p<0.05. Intervention groups had significantly reduced healthcare utilisation (SDM-control mean(SD) between group difference=20.36(-1.5)*, p<0.05; SDM-clinician decision-making mean(SD) between group difference=0.01(2.7)*, p>0.05; clinician decision-making-control mean(SD) between group difference=20.37(-1.5*), p<0.05. The SDM group rated their influence on the treatment decision as similar to clinicians' influence (mean(SD)=3.1(0.6). The SDM group ratings were significantly different from the clinician decision-making group, with the latter feeling their clinician had greater influence (mean(SD)=2.5(0.9), p<0.05). Medication adherence significantly increased in intervention groups (SDM-control mean(SD) between group difference=0.21(0.5)*, p<0.05; SDM–clinician decision-making mean(SD) between group difference=0.08(0.4)*, p>0.05; CDM-control mean(SD) between group	⊕⊕⊕◯ Moderate certainty because of risk of bias.

For peer review only - http://bmjopen.bmj.com/site/about/guidelines.xhtml

difference=0.13(0.4)*, p<0.05, but not at 2 years (SDM-control mean(SD) between group difference=0.03(0.4)*, p>0.05; SDM-clinician decision-making mean(SD) between group difference=0.04(0.4)*, p>0.05; clinician decision-making-control mean(SD) between group difference=-0.01(-0.4)*, p>0.05. Protocol adherence was high (SDM=4.0, clinician decision-making=3.9) and did not differ significantly between groups (p>0.05).

For peer review only

For peer review only - http://bmjopen.bmj.com/site/about/guidelines.xhtml

SDM Systematic Review

Table II: Summary of interventions

First Author (Year)	Description of intervention	Interventionist (including any training provided)	Intervention dose	Description of control group
Au (2012)	Consultation using a completed patient-specific feedback form which describes patient preferences regarding end of life care. The feedback form was not endorsed for use.	Clinicians.<br>No specific training described.	Single session.	Routine outpatient COPD care.
Brundage (2001)	Consultation using a patient decision aid to facilitate cancer treatment decision-making.	Researcher.<br>No specific training described.	Single session.	Participant outcomes prior to receipt of intervention.
Collinsworth (2018)	COPD education and SDM self-management planning.	Registered respiratory therapists.<br>No specific training described.	Single session and four telephone calls.	Routine inpatient care and COPD education.
Gagne (2017)	Patient decision aid to facilitate asthma treatment decision-making (and asthma education).	Certified asthma educators from the Quebec Asthma and COPD Network.	Single session.	Routine outpatient asthma care, education, and action plan generation.
Granados-Santiago (2019)	A SDM patient involvement programme focussing on COPD self-management goals to facilitate self-management decision-making.	Healthcare professionals.<br>No specific training described.	Delivered alongside routine inpatient care.	Routine inpatient care for an exacerbation of COPD.
Myers (2021)	Personalised patient decision aid used to facilitate 15-20min decision coaching consultation.	Oncology nurses.<br>No training provided.	Single session.	Participant outcomes prior to receipt of intervention.
Walters (2013)	Psychoeducation about common psychological reactions to COPD diagnosis and treatment, self-management skills training, cognitive coping skills training to identify and challenge negative COPD-related cognitions, communication skills to facilitate discussion between the health mentor and the patient and promoting self-efficacy to manage chronic illness.	Community health nurses, termed 'Health Mentors.'<br>Training included 12 hours of training over 2 days that covered COPD management (1 h), chronic disease self-management and health behaviour change components including practice role plays (7.25 hours), online training and study methods (3.75 hours).	Single session and sixteen telephone calls.	Routine outpatient COPD care and monthly telephone calls for 12 months (excluded intervention components).

For peer review only - http://bmjopen.bmj.com/site/about/guidelines.xhtml

For peer review only

Wilson (2010)	Shared decision-making intervention:<br>Consultation to facilitate asthma treatment decision-making followed by four contacts to assess patient progress and medication changes as needed.	Healthcare professionals involved in routine asthma care (nurses, respiratory therapists, pharmacists, nurse practitioners and physician assistants).<br>Training provided but not described.	Two sessions and 3 telephone calls.	Routine asthma outpatient care (including, in some sites, the opportunity to of referral to an asthma care a management programme).
	Clinician decision-making intervention:<br>Consultation for clinicians to obtains a patient's level of asthma control, prescribe an appropriate treatment regimen and communicate that to the patient.	Healthcare professionals involved in routine asthma care (nurses, respiratory therapists, pharmacists, nurse practitioners and physician assistants).<br>Training provided but not described.	Two sessions and 3 telephone calls.	

For peer review only

For peer review only - http://bmjopen.bmj.com/site/about/guidelines.xhtml

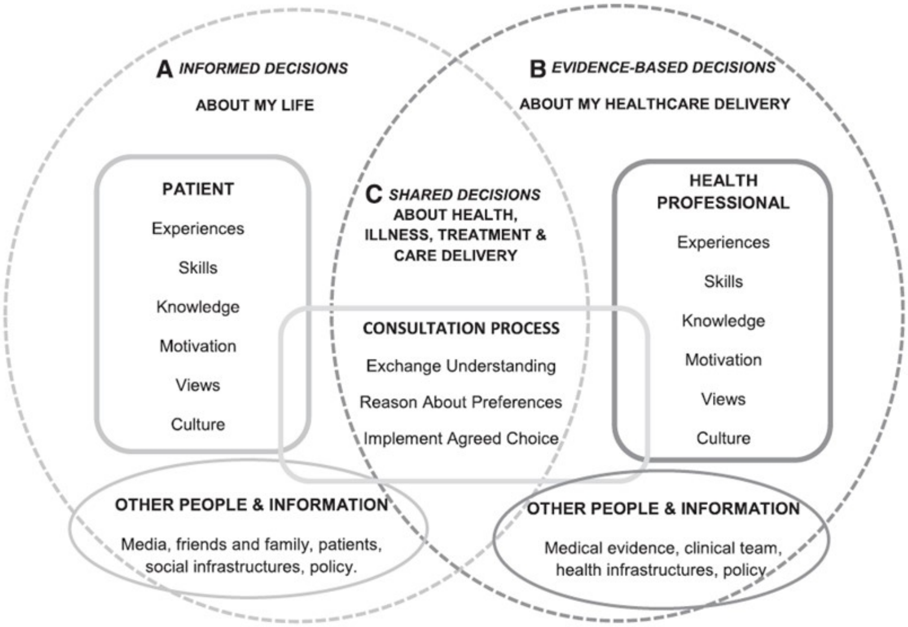

MIND-IT model; a framework representing informed, evidence-based, and shared decisions with patients and their healthcare professional (Bekker et al., 2015)

75x49mm (300 x 300 DPI)

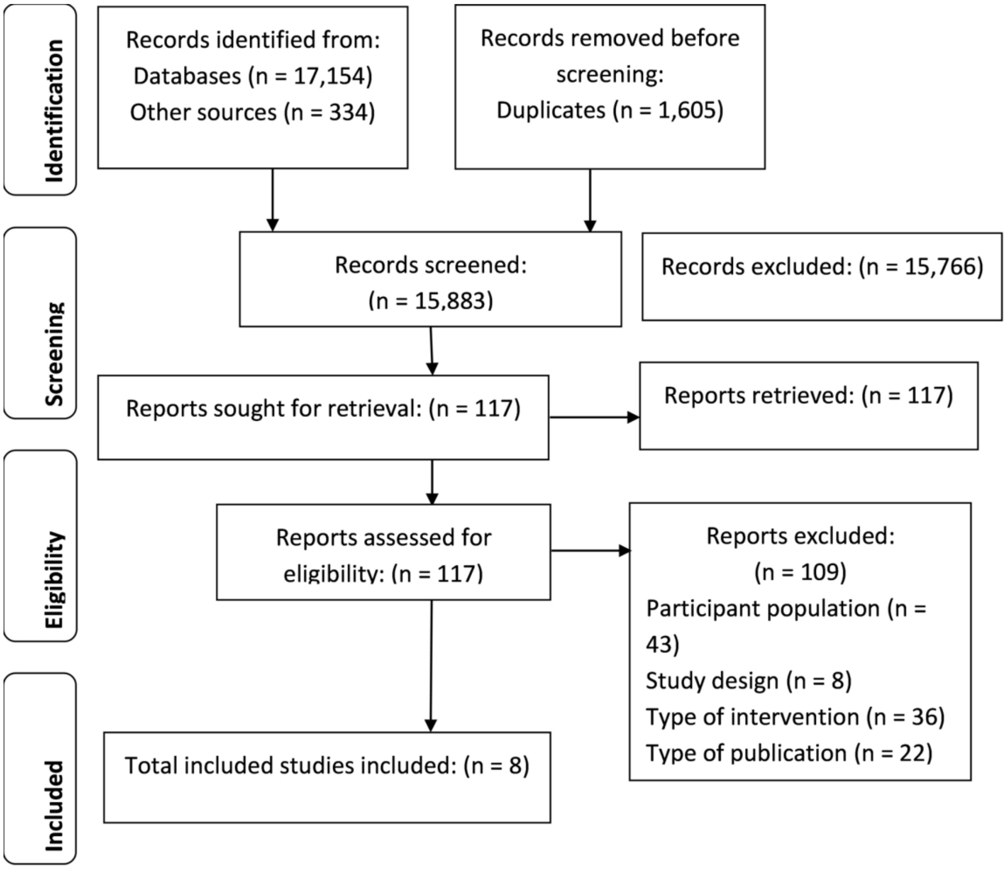

Figure II: PRISMA flow diagram of review process

159x143mm (300 x 300 DPI)

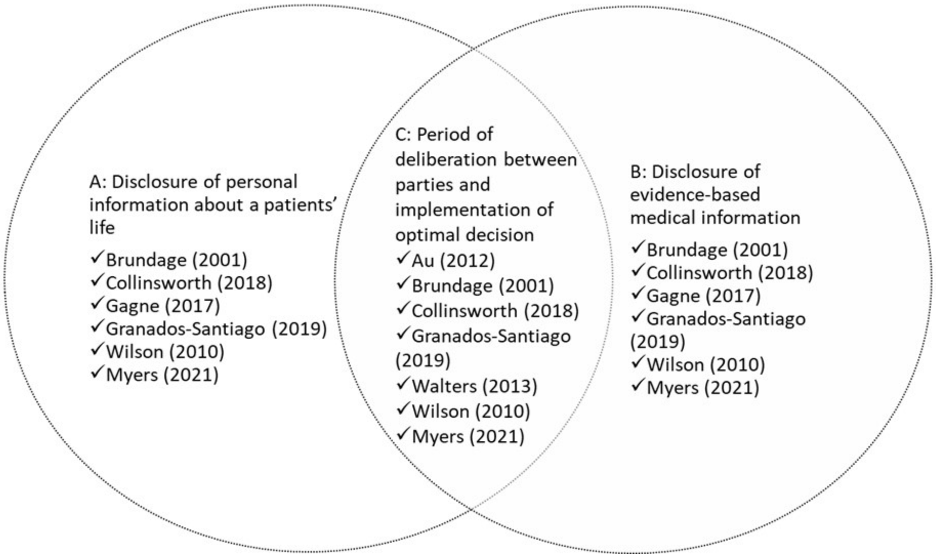

SDM intervention principles aligned to the MIND-IT model (Bekker et al., 2015)

82x45mm (300 x 300 DPI)

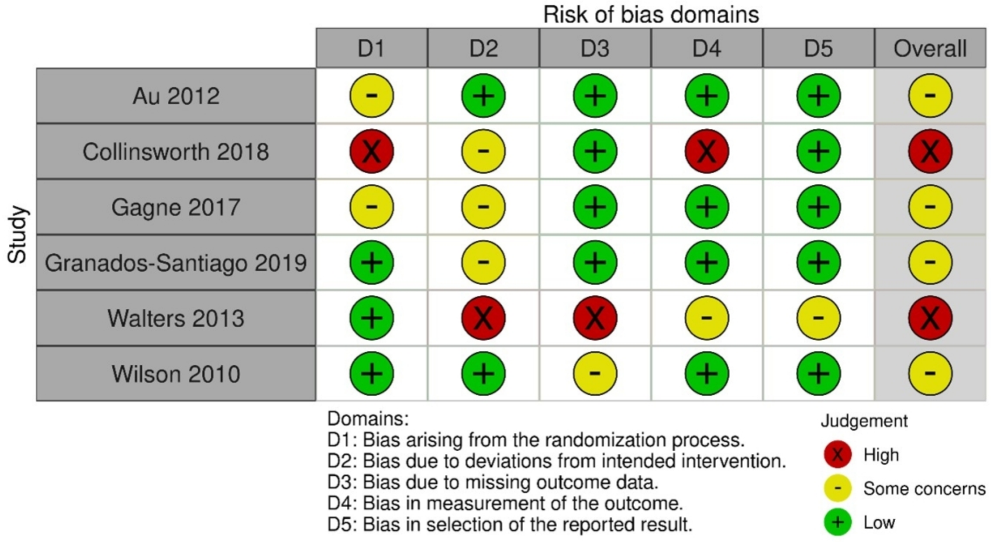

Risk of bias in RCT studies

116x72mm (300 x 300 DPI)

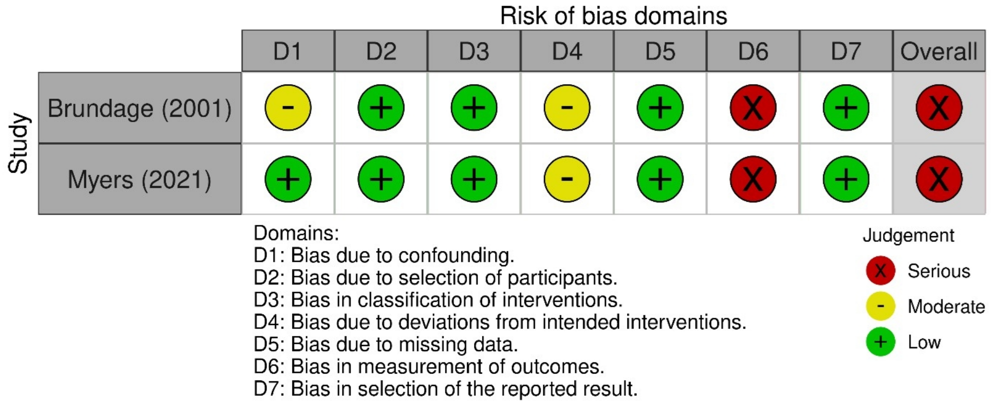

Risk of bias in non-RCT studies

116x57mm (300 x 300 DPI)

Supplementary Material

Title:

A systematic review of shared decision-making interventions for people living with chronic respiratory diseases

Authors:

Barradell, AC; Gerlis, C; Houchen-Wolloff L; Bekker, HL; Robertson, N; Singh, SJ.

For peer review only

For peer review only - http://bmjopen.bmj.com/site/about/guidelines.xhtml

Supplementary Material 1:

MEDLINE search strategy

1. ((joint* or share* or sharing* or informed*) adj3 (decision* or decid*3 or choice*)).mp. [mp=title, abstract, original title, name of substance word, subject heading word, floating sub-heading word, keyword heading word, organism supplementary concept word, protocol supplementary concept word, rare disease supplementary concept word, unique identifier, synonyms]

2. "DECISION MAKING, SHARED"/

3. exp "DECISION MAKING"/

4. "DECISION SUPPORT TECHNIQUES"/

5. DECISION SUPPORT SYSTEMS, CLINICAL/

6. ((decision* or choice*) and (making* or support* or behavior* or behaviour*)).ti,ab.

7. PATIENT PARTICIPATION/

8. ((patient* or consumer*) adj3 (involvement* or involving* or participation* or participating*)).ti,ab.

9. "PATIENT-CENTERED CARE"/

10. "PROFESSIONAL-PATIENT RELATIONS"/

11. "PHYSICIAN-PATIENT RELATIONS"/

12. ((patient* or person* or client* or consumer*) adj3 (centred or centered or focused or oriented)).ti,ab.

13. "decision tool*".ti,ab.

14. "clinical counsel*".ti,ab.

15. "patient decision guide*".ti,ab.

16. "decision coaching".ti,ab.

17. "decision aid*".ti,ab.

18. or/1-17

19. LUNG DISEASES, OBSTRUCTIVE/

20. exp PULMONARY DISEASE, CHRONIC OBSTRUCTIVE/

21. emphysema*.ti,ab.

22. (chronic* adj3 bronchiti*).ti,ab.

23. (obstruct* adj3 (pulmonary or lung* or airway* or airflow* or bronch* or respirat*)).ti,ab.

24. (coad or cobd or copd or aecb).ti,ab.

25. asthma*.ti,ab.

26. exp ASTHMA/

27. (idiopathic and pulmonary and fibrosis).ti,ab.

28. IDIOPATHIC PULMONARY FIBROSIS/

29. (interstitial and lung and disease).ti,ab.

30. LUNG DISEASES, INTERSTITIAL/

31. (extrinsic and allergic and alveolitis).ti,ab.

32. ALVEOLITIS, EXTRINSIC ALLERGIC/

33. (non and specific and interstitial and (pneumonitis or NSIP)).ti,ab.

For peer review only
For peer review only - http://bmjopen.bmj.com/site/about/guidelines.xhtml

34. (Usual and interstitial and (pneumonia or UIP)).ti,ab.

35. "LUNG DISEASES, OBSTRUCTIVE"/

36. ((lung or pulmonary or airway) adj disease).ti,ab.

37. "cystic fibrosis".ti,ab.

38. CYSTIC FIBROSIS/

39. "SARCOIDOSIS, PULMONARY"/

40. sarcoidosis.ti,ab.

41. exp "LUNG NEOPLASMS"/

42. (lung adj (cancer* or tumo?r* or neoplas*)).ti,ab.

43. "TUBERCULOSIS, PULMONARY"/

44. tuberculosis.ti,ab.

45. BRONCHIECTASIS/

46. bronchiectasis.ti,ab.

47. exp PNEUMOCONIOSIS/

48. (autoimmune adj3 (lung or airway or pulmonary)).ti,ab.

49. or/19-48

50. randomized controlled trial.pt.

51. controlled clinical trial.pt.

52. randomized.ab.

53. placebo.ab.

54. clinical trials as topic.sh.

55. randomly.ab.

56. trial.ti.

57. or/50-56

58. exp ANIMALS/ not humans.sh.

59. 57 not 58

60. 59 and 18 and 49

For peer review only

For peer review only - http://bmjopen.bmj.com/site/about/guidelines.xhtml

Supplementary Material 2:

Data items

Extracted items included: population (age, sex, health literacy skill, diagnosis, disease severity, length of diagnosis, baseline measures, total number randomised), intervention (group names, group sample size, adherence to SDM principles, adherence to complex intervention development guidelines, content, training, timing, frequency, duration and any other interventions), comparator (group names, group sample size, content, training, timing, frequency, duration) and outcomes (type, unit of analysis, reported missing data). Data was also extracted on each study’s objectives, study design, unit of allocation, decision posed (e.g. medication, pulmonary rehabilitation, surgery, end of life), duration of the study and follow up.

Sub-group analysis

The following additional data was collected for sub-group analysis: diagnosis, characteristics (age, sex, health literacy skill), decision posed (medication, pulmonary rehabilitation, surgery, end of life), intervention dose (number and length of sessions) and length of follow-up.

For peer review only

For peer review only - http://bmjopen.bmj.com/site/about/guidelines.xhtml

Supplementary Material 3:

Characteristics of excluded studies

Lead Author	Year	Reason for exclusion	Categorised reason for exclusion
Aaron[1]	2006	Abstract only (results in Vandemheen 2009)	Type of publication
Aboumatar[2]	2017	No results in paper (in Aboumatar 2019)	Type of publication
Aboumatar[3]	2019	No SDM within intervention	Type of intervention
Braun[4]	2005	Abstract only (in Menon 2016)	Type of publication
Brown [5]	2012	CRD participant data not distinguishable from analysis	Participant population
Brown [6]	2004	CRD participant data not distinguishable from analysis	Participant population
Brundage[7]	1998	CRD participant data not distinguishable from analysis	Participant population
Butow[8]	2015	No CRD participants	Participant population
Carlucci [9]	2016	No comparator group	Study design
Cook[10]	2016	No adult CRD participants	Participant population
Coultas[11]	2005	No SDM within intervention	Type of intervention
Cvietusa[12]	2020	No SDM within intervention	Type of intervention
Daly[13]	2013	CRD participant data not distinguishable from analysis	Participant population
Detmar[14]	2002	CRD participant data not distinguishable from analysis	Participant population
Dyar[15]	2012	CRD participant data not distinguishable from analysis	Participant population
Early[16]	2015	No SDM within intervention	Type of intervention
Fiks[17]	2015	No CRD participants	Participant population
Fors[18]	2018	CRD participant data not distinguishable from analysis	Participant population
Foster[19]	2016	No SDM within intervention	Type of intervention
Foster[20]	2014	No more than one choice	Type of intervention
Fraenkel[21]	2016	No CRD participants	Participant population

For peer review only - http://bmjopen.bmj.com/site/about/guidelines.xhtml

Gagne[22]	2016	Abstract only (results in Gagne 2017)	Type of publication
George[23]	2017	Abstract only - (results in George 2020)	Type of publication
George[24]	2020	No more than one choice	Type of intervention
Gupta[25]	2019	No SDM within intervention	Type of intervention
Hagan[26]	2008	No SDM within intervention	Type of intervention
Halley[27]	2015	No CRD participants	Participant population
Hilberink[28]	2011	No SDM within intervention	Type of intervention
Hoerger[29]	2017	CRD participant data not distinguishable from analysis	Participant population
Hollen[30]	2013	No comparator group	Study design
Hoskins[31]	2016	No SDM within intervention	Type of intervention
Houben[32]	2019	No SDM within intervention	Type of intervention
Hyatt[33]	2018	CRD participant data not distinguishable from analysis	Participant population
Izumi[34]	2018	CRD participant data not distinguishable from analysis	Participant population
Jonsdottir[35]	2015	No SDM within intervention	Type of intervention
Kass[36]	2009	CRD participant data not distinguishable from analysis	Participant population
Kennedy[37]	2013	CRD participant data not distinguishable from analysis	Participant population
Koerner[38]	2014	No CRD participants	Participant population
Krug[39]	2021	No SDM within intervention	Type of intervention
Lainscak[40]	2013	No SDM within intervention	Type of intervention
Latimer[41]	2007	CRD participant data not distinguishable from analysis	Participant population
Lavoie[42]	2014	No SDM within intervention	Type of intervention
Leung[43]	2012	Cross-sectional study	Study design
Lim[44]	2021	Hypothetical decision making	Type of intervention
Llanos-Paez[45]	2021	No SDM within intervention	Type of intervention

For peer review only - http://bmjopen.bmj.com/site/about/guidelines.xhtml

Lopez-Lopez[46]	2020	No SDM within intervention	Type of intervention
Ludden[47]	2018	Adult CRD participant data not distinguishable from analysis	Participant population
Makimoto[48]	2020	Letter to editor	Type of publication
Markman[49]	2005	No comparator group	Study design
Markun[50]	2018	No participant-facing content	Type of intervention
Mayer[51]	2016	CRD participant data not distinguishable from analysis	Participant population
Meline[52]	2021	No CRD participants	Participant population
Melzer[53]	2018	No SDM within intervention	Type of intervention
Menon[54]	2016	CRD participant data not distinguishable from analysis	Participant population
Meropol[55]	2013	CRD participant data not distinguishable from analysis	Participant population
Moullec[56]	2012	No SDM within intervention	Type of intervention
Murray[57]	2010	CRD participant data not distinguishable from analysis	Participant population
Nedjat-Haiem[58]	2017	CRD participant data not distinguishable from analysis	Participant population
Nugent[59]	2018	No comparator group	Study design
Patel[60]	2019	No participant-facing content	Type of intervention
Rabow[61]	2004	CRD participant data not distinguishable from analysis	Participant population
Rea[62]	2004	No SDM within intervention	Type of intervention
Reddel[63]	2019	Abstract only (results not yet published)	Type of publication
Reinke[64]	2017	No SDM outcomes	Outcome measures
Rhee[65]	2008	Participants <18years old	Participant population
Roelands[66]	2004	No CRD participants	Participant population
Rosemann[67]	2013	Abstract only (results in Steurer 2017)	Type of publication
Ruparel[68]	2019	No CRD participants	Participant population
Rutten-vanMolken[69]	2017	Abstract only - (results in Slok 2016)	Type of publication

Supplementary material V1.0

Sands[70]	2014	Abstract only - (results not yet published)	Type of publication
Schofield[71]	2013	No SDM within intervention	Type of intervention
Schubart[72]	2015	CRD participant data not distinguishable from analysis	Participant population
Schumacher[73]	2002	No quantitative data	Study design
Schwarze[74]	2020	CRD participant data not distinguishable from analysis	Participant population
Shade[75]	2019	Adult CRD participant data not distinguishable from analysis	Participant population
Shirai[76]	2012	CRD participant data not distinguishable from analysis	Participant population
Sinclair[77]	2017	No SDM within intervention	Type of intervention
Skorstengaard[78]	2019	CRD participant data not distinguishable from analysis	Participant population
Slok[79]	2016a	Abstract only (results in Slok 2016b)	Type of publication
Slok[80]	2016b	No SDM within intervention	Type of intervention
Smidth[81]	2013	No comparator group	Study design
Steinhauser[82]	2008	CRD participant data not distinguishable from analysis	Participant population
Steurer[83]	2017	Abstract only (results in Steurer 2015)	Type of publication
Steurer-Stey[84]	2015	No SDM within intervention	Type of intervention
Sullivan[85]	2020	Abstract only	Type of publication
Tai-Seale[86]	2016	CRD participant data not distinguishable from analysis	Participant population
Tamblyn[87]	2005	Abstract only (results in Tamblyn 2015)	Type of publication
Tamblyn[88]	2015	No SDM within intervention	Type of intervention
Tang[89]	2008	No comparator group	Study design
Tapp[90]	2017	No SDM outcomes	Outcome measures
Thee[91]	2021	Protocol paper	Type of publication
Thiam[92]	2007	No SDM within intervention	Type of intervention
Totten[93]	2018	Abstract only (results not yet published)	Type of publication

For peer review only - http://bmjopen.bmj.com/site/about/guidelines.xhtml

Tsao[94]	2021	No SDM within intervention	Type of intervention
Vandemheen[95]	2009	No SDM within intervention	Type of intervention
Vandemheen[95]	2009	Supplement (to be read with Vandemheen 2009)	Type of publication
Wahlberg[96]	2016	CRD participant data not distinguishable from analysis	Participant population
Waller[97]	2012	CRD participant data not distinguishable from analysis	Participant population
Wang[98]	2010	No SDM within intervention	Type of intervention
Weekes[99]	2009	No SDM within intervention	Type of intervention
Williams[100]	2013	CRD participant data not distinguishable from analysis	Participant population
Wilson[101]	2009	Abstract only (results in Wilson 2010)	Type of publication
Wilson[102]	2005	Abstract only (results in Wilson 2010)	Type of publication
Wilson[103]	2005	Abstract only (results in Wilson 2010)	Type of publication
Wood-Baker[104]	2012	No SDM within intervention	Type of intervention
Yoo[105]	2018	No CRD participants	Participant population
Zakrisson[106]	2019	CRD participant data not distinguishable from analysis	Participant population
Zanartu[107]	2017	Letter to editor	Type of publication

only

For peer review only - http://bmjopen.bmj.com/site/about/guidelines.xhtml

Supplementary Material 4:

Summary of studies (extended version)

First Author, Year, Country, Sample size, Study design, Population	Outcome(s)	Summary of results	Certainty of evidence (GRADE score)
Au, 2012, USA, Clinicians = 92 Patients = 376, RCT, Outpatients with COPD	*Validated:* Quality of communication (QOC). 19-items scored from 0-100 (higher score=better communication). *Non-validated:* Occurrence of discussions about end of life preferences between patients and either clinician or surrogate. 4-item self-reported questionnaire scored from 0-100% (higher%=greater occurrence).	Baseline quality of communication was poor in both groups. Modest improvements in both groups, but significant mean between group difference favouring the intervention group (5.7 points; p<0.05). The occurrence (%) of patient communication about end of life care was significantly higher in the intervention group for three of four questions (mean(SD)): 1. Int: 35.2(74.6) Con: 15.9(58.2) p<0.05, 2. Int: 60.3(122.9) Con: 30.8(175.5) p<0.05, 3. Int: 53.6(115.1) Con: 45.2(73.3) p>0.05, 4. Int: 86.2(84.6) Con: 75.2(115.0) p<0.05.	Moderate ⊕⊕⊕◯ Moderate certainty because of risk of bias.
Brundage, 2001, Canada, Patients = 20, Feasibility CBA, Outpatients with lung cancer	*Validated:* Decisional conflict (DCS). 16-items using a 4-point Likert scale anchored with strongly agree and strongly disagree. Converted scale scores >37.5 indicate meaningful decisional conflict or delayed decision implementation. *Non-validated:* Knowledge of treatment options. Three open-ended questions. Strength of treatment preference. 1 item using a 7-point Likert scale anchored with definitely wish treatment 1 and definitely wish treatment 2.	Decisional conflict reduced after receiving the intervention in 87% and increased in 13% of participants. Knowledge of the available treatment options improved following the intervention for both 3-year outcome survival[a] and median survival outcome[b]: 1. Knowledge of survival outcomes with treatment 1 alone (mean between group difference 60%[a]; 54%[b]), 2. Knowledge of direction of survival difference between treatments (mean between group difference: 27%[a]; 40%[b]), 3. Knowledge of the magnitude of survival difference between treatments (mean between group difference: 73%[a]; 67%[b]). The number of participants with a treatment preference increased from 80% to 100% following the intervention.	Low ⊕⊕◯◯ Low certainty because of risk of bias and minor publication bias.
Collinsworth, 2018, USA, Patients = 308, RCT, Inpatients with COPD	*Validated:* Patient activation (PAM). 22-items using a 4-point Likert scale anchored with patient is disengaged and overwhelmed and patient has adopted and is maintaining healthy behaviours. Quality of life health status (CAT). 8 item scale with scores ranging from 0-40. Lower scores (<10) indicate COPD	Both groups had a significant improvement in PAM scores following the intervention (mean(SD) difference: 0.52(0.9) and 0.69(1.0) points respectively, p<0.05). Only the intervention group had significantly increased scores in the CAT following the intervention (mean(SD) difference: Int: 5.27(10.3), p<0.05; Con: 0.38(7.8), p>0.05). There were no significant differences in the total number of all-cause or COPD-related readmissions between groups at 1, 2, 3, 6 and 9 months (p>0.05).	Moderate ⊕⊕⊕◯ Moderate certainty because of risk of bias.

For peer review only - http://bmjopen.bmj.com/site/about/guidelines.xhtml

For peer review only

	causes few problems and higher scores (>30) indicate COPD prevents patients from doing most things they want to do. *Non-validated:* All-cause hospital admissions extracted from electronic health records. Reported as frequency count.		
Gagne, 2017, Canada, Patients = 51, RCT, Outpatients with asthma	*Validated:* Asthma knowledge (QCALF). 37-items with scores ranging from -37 to +37 (higher scores indicate greater knowledge). Decisional conflict (DCS; see above). *Non-validated*: Appropriate use of asthma pharmacotherapy. 4-item face-to-face interviewer-administered questionnaire. The cut off for appropriate use was meeting eleven hierarchical criteria.	Both groups had significantly increased knowledge scores following the intervention (mean(SD) difference: Int: 3.6(8.9)*, p<0.001; Con: 2(7.3)*, p<0.05). There was no significant between group difference (p>0.05). Both groups had significantly reduced decisional conflict scores following the intervention (mean(SD) difference: Int: 8.1(22.5)*, p<0.05; Con: 9.1(27.7)*, p>0.05). There was no significant between group difference (p>0.05). Both groups had modest, but non-significant, within group improvements in their appropriate use of pharmacotherapy (mean(SD) difference: Int: 0.15(0.7)*, p>0.05; Con: 0.16(0.7)*, p>0.05). There was no significant between group difference (mean(SD) between group difference = 0.17(0.7), p>0.05).	Moderate ⊕⊕⊕◯ Moderate certainty because of risk of bias.
Granados-Santiago, 2019, Spain, Patients = 42, RCT, Inpatients with COPD	*Validated:* Quality of life and health status (EQ-5D). 5-items with scores ranging from 0 (defined as the worst imaginable health state) to 100 (defined as the best imaginable health state). COPD knowledge (COPD-Q). 13-items with scores ranging from 0 (defined as lowest knowledge) to 13 (defined as highest knowledge). Medication adherence (TAI).10-items, with higher scores indicating greater adherence. Physical activity (steps/day) measured using the Fitbit Flex 2 Pedometer. Nutritional status (MNA). 18-items with scores lower than 17 considered malnutrition. *Non-validated:* -	Both groups had significant and clinically important improvements in their quality of life and health status at discharge (mean(SD) between group difference: 6.15(27.5)*, p<0.05) but only the intervention group maintained this benefit 3 months later (mean(SD) between group difference: 8.28(50.6)*, p<0.05). Knowledge scores significantly improved in the intervention group alone at discharge (mean(SD) between group difference: 3.89(0.7)*, p<0.05) and maintained at 3 months follow up (mean(SD) between group difference: 3.88(0.8)*, p<0.05). Adherence significantly improved at discharge in the intervention group alone (mean(SD) difference: Int: 3.58(1.5)*, p<0.05; Con: 1.36(1.6)*, p>0.05) but there was no significant between group difference. At 3 months post intervention there was a significant mean(SD) between group difference favouring the intervention group (1.8(2.4)*, p<0.05). Upon discharge, there was a significant reduction in physical activity in both groups (mean(SD) difference: Int: -348.27(2.0)*, p<0.05; Con: -410.05(4.66)*, p<0.05). At 3 months post intervention there was a significant improvement in the intervention group alone (mean(SD) difference: Int: 1371.97(895.2)*, p<0.05; Con: 620.18(12.5)*, p>0.05). At 3 months post intervention there was a significant improvement in nutritional status both within and between groups for the intervention group alone (mean(SD) difference: Int: 4.15(0.1)*, p<0.05; Con: -3.43(0.12)*, p>0.05	Moderate ⊕⊕⊕◯ Moderate certainty because of risk of bias.
Myers, 2021, USA, Patients = 5,	*Validated:* Decisional Conflict (DCS SURE Test). 4-item scale with scores from 0-4, a score <4 indicates clinically significant	Decisional conflict reduced post intervention (mean difference=1). Awareness of treatment options increased from 40% to all 100% participants post intervention. At 30 days post intervention, 80% of participants had made a decision. 40% of participants had	Low ⊕⊕◯◯ Low certainty

For peer review only - http://bmjopen.bmj.com/site/about/guidelines.xhtml

Pilot CBA, Outpatients with non-small cell lung cancer	decisional conflict. *Non-validated:* Knowledge of treatment options. 3-item survey using a 3-point Likert scale (Yes/No/Unsure) to elicit patient beliefs regarding curability of treatment options, awareness of treatment options, and treatment costs. Awareness of treatment options only item included in this analysis as only item measuring knowledge. Treatment status. 30-day treatment status extracted from electronic health records. This was compared to treatment preferences immediately post intervention.	a treatment that matched their treatment preference.	because of risk of bias.
Walters, 2013, Australia, Patients = 182, Cluster RCT, Outpatients with COPD	*Validated:* Quality of life (SF-36). 8-items with scores from 0-100, higher score indicates better quality of life. Quality of life (SGRQ). 76-items with scores ranging from 0-100, higher score indicates worse quality of life. Patient involvement in healthcare decisions (PIH). 12-items with scores ranging from 0-8 (higher score indicates more involvement). *Non-validated:* Respiratory hospital admissions extracted from electronic health records. Reported as frequency count. Intervention fidelity. Recorded telephone calls from the first three intervention and 10% of control contacts were coded by two raters for content and fidelity to prespecified components of the intervention to assess adherence. To ensure concordance, both raters coded a random sample of 20 calls.	Neither group had a significant change to either their physical[a] or mental[b] health status at 6 months (mean(SD) between group difference: 1.5(0.2)*[a]; 0.8(1.4)*[b], p>0.05) and 12 months (mean(SD) between group difference: 0.0(0.9)*[a]; 0.3(0.9)*[b], p>0.05) post intervention. Neither group had a significant change to their overall quality of life at 6 months (mean(SD) between group difference: 1.9(2.7)*, p>0.05) and 12 months (mean(SD) between group difference: 1.4(1.5)*, p>0.05 post intervention. Only the intervention group had a significant improvement in PIH scores at 6 months (mean(SD) between group difference: 0.4(0.2)*, p<0.05) and 12 months (mean(SD) between group difference: 0.3(0.1)*, p<0.05) post intervention. No significant change in the number of hospital admissions for either group over the 12 months study period (χ^2=2.61, p>0.05). Concordance between two raters was high (overall weighted κ 0.72). Fidelity assessments confirmed specific health mentoring components were addressed with some clarity: 1. COPD symptom management, 49%, 2. Unhelpful self-talk explored and identified, 37%, 3. Unhelpful self-talk challenged, and new self-talk developed, 35%, 4. Action plan for achieving goals made, 54%, 5. Problems and barriers to achieving goals identified and clarified, 46%, 6. Positive changes in behaviour praised, 83%. Raters concluded there was a lower delivery of cognitive behavioural components of intervention.	Very low ⊕◯◯◯ Very low certainty because of significant risk of bias and some inconsistency.
Wilson, 2010, Hawaii, Patients = 612, RCT, Outpatients	*Validated:* Quality of life (MiniAQLQ). 5-items with Likert scale ranging from 0 (all of the time) to 7 (none of the time). *Non-validated:* Healthcare use extracted from electronic health records. Reported as frequency count. Patients' perceived role in treatment decision.	Only intervention groups had a significant improvement in quality of life scores at 12 months post intervention (SDM to control mean(SD) between group difference = 0.39(1.0)*, p<0.05; SDM–clinician decision-making mean(SD) between group difference = 0.11(1.1)*, p>0.05; clinician decision-making-control mean(SD) between group difference = 0.28(1.0)*, p<0.05. Only intervention groups had a significantly reduced healthcare utilisation (SDM-control mean(SD) between group difference =20.36(-1.5)*, p<0.05; SDM-	Moderate ⊕⊕⊕◯ Moderate certainty because of risk of bias.

For peer review only

For peer review only - http://bmjopen.bmj.com/site/about/guidelines.xhtml

with asthma	1-item using a 5-point Likert scale ranging from 0 (low involvement in decision-making) to 5 (high involvement in treatment decision-making). Medication adherence. Data extracted from pharmacy records to calculate a continuous medication acquisition index for each year. Intervention fidelity. Audiotapes of both intervention groups of 10% of patients were scored on a detailed performance checklist to determine whether the two protocols were delivered as intended. Scores ranged from 0-4 with higher scores indicated greater adherence.	clinician decision-making mean(SD) between group difference =0.01(2.7)*, p>0.05; clinician decision-making-control mean(SD) between group difference= 20.37(-1.5*), p<0.05. The SDM group rated their influence on the treatment decision as being approximately the same as the clinicians' influence (mean(SD)= 3.1(0.6). The SDM group ratings were significantly different from the clinician decision-making group, with the latter feeling that their clinician had a greater influence (mean(SD)=2.5(0.9), p<0.05) than they did themselves. Medication adherence significantly increased only in intervention groups (SDM-control mean(SD) between group difference=0.21(0.5)*, p<0.05; SDM–clinician decision-making mean(SD) between group difference= 0.08(0.4)*, p>0.05; CDM-control mean(SD) between group difference= 0.13(0.4)*, p<0.05. This was not sustained at 2 years (SDM-control mean(SD) between group difference= 0.03(0.4)*, p>0.05; SDM-clinician decision-making mean(SD) between group difference= 0.04(0.4)*, p>0.05; clinician decision-making-control mean(SD) between group difference=-0.01(-0.4)*, p>0.05. Protocol adherence scores assigned by the raters were high (SDM=4.0, clinician decision-making=3.9) and did not differ significantly between the two groups (p>0.05).

For peer review only - http://bmjopen.bmj.com/site/about/guidelines.xhtml

For peer review only

Supplementary Material 5:

5a: Sub-group analysis

Despite the broad inclusion criteria for respiratory conditions, studies only included those with COPD, lung cancer and asthma (Table below). Participants diagnosed with lung cancer and COPD were older than those diagnosed with asthma. The split between male and female participants were even across studies apart from in one study where nearly all were male[108] and another where three quarters were female[109]. No specific health literacy outcome measures were used across studies. Baseline educational attainment was reported across all but two studies[110,111]. Across other studies, the percentage of participants who completed higher education varied between 17-59.2%.

SDM interventions for COPD participants aimed to facilitate decisions regarding end-of-life care and positive self-management. Those for lung cancer and asthma participants aimed to facilitate treatment decision-making. Four studies reported a single session SDM intervention[108,109,112,113]. Three studies reported multiple sessions of SDM delivered face to face and with follow up telephone calls[110,114,115]. One study did not report an intervention dose and instead stated the intervention was integrated into routine inpatient care[111]. The length of study follow-up varied from immediately post decision-making to 12 months later.

For peer review only

For peer review only - http://bmjopen.bmj.com/site/about/guidelines.xhtml

5b: Sub-group analysis of CRD patients from included studies

First Author (Year)	CRD diagnosis	Age (mean (SD))	Gender (% male)	Measure of health literacy (% achievement)	Decision posed	Intervention dose	Length of follow up
Au (2012)	COPD	IG: 69.4(10.0) years<br>CG: 69.4(10.0) years	IG: 97.9%<br>CG: 96.2%	IG: Higher education = 58.3%<br>CG: Higher education = 59.2%	IG: End of life care<br>CG: End of life care	IG: Single session<br>CG: N/A	IG: 2 weeks<br>CG: 2 weeks
Brundage (2001)	Lung cancer	IG: 69.2(8.1) years	IG: 55%	IG: <High school = 5%<br>High school education = 50%<br>Higher education = 45%	IG: Lung cancer treatments	IG: Single session	IG: Immediately post intervention
Collinsworth (2018)	COPD	IG: 70.0(11.9)<br>CG: 70.9(12.5)	IG: 39.7%<br>CG: 43.1%	IG: Not disclosed<br>CG: Not disclosed	IG: Self-management plans<br>CG: Self-management plans	IG: Single session and 4 telephone calls<br>CG: N/A	IG: 6 months<br>CG: 6months
Gagne (2017)	Asthma	IG: 46(13.0)<br>CG: 41(13.0)	IG: 26.9%<br>CG: 48%	IG: High school education = 57.7%<br>Higher education = 42.3%<br>CG: High school education = 52.0%<br>Higher education = 48.0%	IG: Asthma treatments<br>CG: Asthma treatments	IG: Single session<br>CG: N/A	IG: 2months<br>CG: 2months
Granados-Santiago (2019)	COPD	IG: 69.3(9.9)<br>CG: 74.20(9.25)	IG: Not disclosed<br>CG: Not disclosed	IG: Not disclosed<br>CG: Not disclosed	IG: Self-management plans<br>CG: Self-management plans	IG: Integrated into inpatient care<br>CG: N/A	IG: 3 months<br>CG: 3 months
Myers (2021)	Lung cancer	IG: Not reported	IG: 40%	IG: <High school education = 0%<br>High school education = 40%	IG: Lung cancer treatments	IG: Single session	IG: 30 days

Supplementary material V1.0

For peer review only - http://bmjopen.bmj.com/site/about/guidelines.xhtml

				Higher education = 60%			
Walters (2013)	COPD	IG: 68.2(7.9)	IG: 54.4%	IG:<br><High school education = 68%<br>High school education = 13%<br>Higher education = 20%	IG: Self-management plans	IG: Single session plus multiple (x16) telephone calls	IG: 12 months
		CG: 67.3(7.6)	CG: 51.1%	CG:<br><High school education = 75%<br>High school education = 8%<br>Higher education = 17%	CG: Self-management plans	CG: Single session plus multiple (x12) telephone calls	CG: 12 months
Wilson (2010)	Asthma	IG: 45.7(13.3)	IG: 43.6%	IG:<br><High school education = 2.9%<br>High school education = 55.9%<br>Higher education = 41.1%	IG: Asthma treatments	IG: Multiple sessions (x2) plus multiple telephone calls (x3)	IG: 12 months
		IG2: 46.9(12.1)	IG2: 44.1%	IG2:<br><High school education = 0.98%<br>High school education = 64.7%<br>Higher education = 33.8%	IG2: Asthma treatments	IG2: Multiple sessions (x2) plus multiple telephone calls (x3)	IG2: 9 months
		CG: 45.1(12.4)	CG: 42.6%	CG:<br><High school education= 2.9%<br>High school education = 56.9%<br>Higher education = 40.2%	CG: Asthma treatments	CG: Multiple sessions (x2) plus multiple telephone calls (x3)	CG: 9 months

IG = Intervention group; CG = Control group; IG2 = Intervention group 2 (Clinician Decision-Making)

For peer review only

For peer review only - http://bmjopen.bmj.com/site/about/guidelines.xhtml

References

E1. Aaron SD. Evaluation of a Decision Aid for Adult Cystic Fibrosis Patients Considering Bilateral Lung Transplantation. *Clin Regist Clin trials*. 2006.

2. Aboumatar H, Naqibuddin M, Chung S, et al. Better Respiratory Education and Treatment Help Empower (BREATHE) study: Methodology and baseline characteristics of a randomized controlled trial testing a transitional care program to improve patient-centered care delivery among chronic obstructive pulm. *Contemp Clin Trials*. 2017;62(June):159-167. doi:10.1016/j.cct.2017.08.018

3. Aboumatar H, Naqibuddin M, Chung S, et al. Effect of a Hospital-Initiated Program Combining Transitional Care and Long-term Self-management Support on Outcomes of Patients Hospitalized with Chronic Obstructive Pulmonary Disease: A Randomized Clinical Trial. *JAMA - J Am Med Assoc*. 2019;322(14):1371-1380. doi:10.1001/jama.2019.11982

4. Braun U. A Culturally Sensitive Values-Guided Aid for End of Life Decision-Making. *Clin Regist Clin Trials*. 2005. doi:10.1002/CENTRAL/CN-01510546

5. Brown RF, Bylund CL, Li Y, Edgerson S, Butow P. Testing the utility of a cancer clinical trial specific Question Prompt List (QPL-CT) during oncology consultations. *Patient Educ Couns*. 2012;88(2):311-317. doi:10.1016/j.pec.2012.02.009

6. Brown RF, Butow PN, Sharrock MA, et al. Education and role modelling for clinical decisions with female cancer patients. *Heal Expect*. 2004;7(4):303-316. doi:10.1111/j.1369-7625.2004.00294.x

7. Brundage MD, Davidson JR, Mackillop WJ, Feldman-Stewart DEB, Groome P. Using a treatment-tradeoff method to elicit preferences for the treatment of locally advanced non-small-cell lung cancer. *Med Decis Mak*. 1998;18(3):256-267. doi:10.1177/0272989X9801800302

8. Butow P, Brown R, Aldridge J, et al. Can consultation skills training change doctors' behaviour to increase involvement of patients in making decisions about standard treatment and clinical trials: A randomized controlled trial. *Heal Expect*. 2015;18(6):2570-2583. doi:10.1111/hex.12229

9. Carlucci A, Vitacca M, Malovini A, et al. End-of-Life Discussion, Patient Understanding and Determinants of Preferences in Very Severe COPD Patients: A Multicentric Study. *COPD J Chronic Obstr Pulm Dis*. 2016;13(5):632-638. doi:10.3109/15412555.2016.1154034

For peer review only - http://bmjopen.bmj.com/site/about/guidelines.xhtml

10. Cook KA, Modena BD, Simon RA. Improvement in Asthma Control Using a Minimally Burdensome and Proactive Smartphone Application. *J Allergy Clin Immunol Pract*. 2016;4(4):730-737.e1. doi:10.1016/j.jaip.2016.03.005

11. Coultas D, Frederick J, Barnett B, Singh G, Wludyka P. A randomized trial of two types of nurse-assisted home care for patients with COPD. *Chest*. 2005;128(4):2017-2024. doi:10.1378/chest.128.4.2017

12. Cvietusa PJ, Wagner NM, Shoup JA, et al. Digital Communication Technology: Does Offering a Choice of Modality Improve Medication Adherence and Outcomes in a Persistent Asthma Population? *Perm J*. 2020;25:1. doi:10.7812/TPP/20.189

13. Daly BJ, Douglas SL, Gunzler D, Lipson AR. Clinical trial of a supportive care team for patients with advanced cancer. *J Pain Symptom Manage*. 2013;46(6):775-784. doi:10.1016/j.jpainsymman.2012.12.008

14. Detmar SB, Muller MJ, Schornagel JH, Wever LDV, Aaronson NK. Health-related quality-of-life assessments and patient-physician communication: A randomized controlled trial. *J Am Med Assoc*. 2002;288(23):3027-3034. doi:10.1001/jama.288.23.3027

15. Dyar S, Lesperance M, Shannon R, Sloan J, Colon-Otero G. A nurse practitioner directed intervention improves the quality of life of patients with metastatic cancer: Results of a randomized pilot study. *J Palliat Med*. 2012;15(8):890-895. doi:10.1089/jpm.2012.0014

16. Early F, Everden AJT, OBrien CM, Fagan PL, Fuld JP. Patient agenda setting in respiratory outpatients. *Chron Respir Dis*. 2015;12(4):347-356. doi:10.1177/1479972315598696

17. Fiks AG, Mayne SL, Karavite DJ, et al. Parent-reported outcomes of a shared decision-making portal in asthma: A practice-based RCT. *Pediatrics*. 2015;135(4):e965-e973. doi:10.1542/peds.2014-3167

18. Fors A, Blanck E, Ali L, et al. Effects of a person-centred telephone-support in patients with chronic obstructive pulmonary disease and/or chronic heart failure – A randomized controlled trial. *PLoS One*. 2018;13(8). doi:10.1371/journal.pone.0203031

19. Foster JM, Smith L, Usherwood T, Sawyer SM, Reddel HK. General practitioner-delivered adherence counseling in asthma: Feasibility and usefulness of skills, training and support tools. *J Asthma*. 2016;53(3):311-320. doi:10.3109/02770903.2015.1091473

20. Foster JM, Usherwood T, Smith L, et al. Inhaler reminders improve adherence with controller treatment in primary care patients with asthma. *J Allergy Clin Immunol*. 2014;134(6):1260-

For peer review only - http://bmjopen.bmj.com/site/about/guidelines.xhtml

1268.e3. doi:10.1016/j.jaci.2014.05.041

21. Fraenkel L, Peters E, Tyra S, Oelberg D. Shared Medical Decision Making in Lung Cancer Screening: Experienced versus Descriptive Risk Formats. *Med Decis Mak*. 2016;36(4):518-525. doi:10.1177/0272989X15611083

22. Gagné M, Légaré F, Moisan J, Boulet L. Adding A Decision Aid to Asthma Education: Impact on Decisional Conflict and Appropriate Medication Usage. *Value Heal*. 2016;19(7):A557. doi:10.1016/j.jval.2016.09.1216

23. George M. BREATHE asthma intervention trial. *Clin Regist Clin Trials*. 2017. doi:10.1002/CENTRAL/CN-01380211

24. George M, Bruzzese JM, S. Sommers M, et al. Group-randomized trial of tailored brief shared decision-making to improve asthma control in urban black adults. *J Adv Nurs*. 2020;(September):1-17. doi:10.1111/jan.14646

25. Gupta S, Price C, Agarwal G, et al. *The Electronic Asthma Management System (EAMS) Improves Primary Care Asthma Management*. Vol 53.; 2019. doi:10.1183/13993003.02241-2018

26. Hagan L, Valois P, Patenaude H, Boutin H, Boulet LP, Lafrenière F. Asthma counselling targeted to removal of domestic animals. *Can Respir J*. 2008;15(1):33-38. doi:10.1155/2008/247027

27. Halley MC, Rendle KAS, Gillespie KA, Stanley KM, Frosch DL. An exploratory mixed-methods crossover study comparing DVD- vs. Web-based patient decision support in three conditions: The importance of patient perspectives. *Heal Expect*. 2015;18(6):2880-2891. doi:10.1111/hex.12273

28. Hilberink SR, Jacobs JE, Breteler MHM, de Vries H, Grol RPTM. General practice counseling for patients with chronic obstructive pulmonary disease to quit smoking: Impact after 1year of two complex interventions. *Patient Educ Couns*. 2011;83(1):120-124. doi:10.1016/j.pec.2010.04.009

29. Hoerger M, Perry LM, Gramling R, Epstein RM, Duberstein PR. Does educating patients about the early palliative care study increase preferences for outpatient palliative cancer care? Findings from project EMPOWER. *Heal Psychol*. 2017;36(6):538-548. doi:10.1037/hea0000489

30. Hollen PJ, Gralla RJ, Jones RA, et al. A theory-based decision aid for patients with cancer:

For peer review only - http://bmjopen.bmj.com/site/about/guidelines.xhtml
For peer review only

Results of feasibility and acceptability testing of DecisionKEYS for cancer. *Support Care Cancer*. 2013;21(3):889-899. doi:10.1007/s00520-012-1603-8

31. Hoskins G, Williams B, Abhyankar P, et al. Achieving Good Outcomes for Asthma Living (GOAL): Mixed methods feasibility and pilot cluster randomised controlled trial of a practical intervention for eliciting, setting and achieving goals for adults with asthma. *Trials*. 2016;17(1):1-17. doi:10.1186/s13063-016-1684-7

32. Houben CHM, Spruit MA, Luyten H, et al. Cluster-randomised trial of a nurse-led advance care planning session in patients with COPD and their loved ones. *Thorax*. 2019;74(4):328-336. doi:10.1136/thoraxjnl-2018-211943

33. Hyatt A, Lipson-Smith R, Gough K, et al. Culturally and linguistically diverse oncology patients' perspectives of consultation audio-recordings and question prompt lists. *Psychooncology*. 2018;27(9):2180-2188. doi:10.1002/pon.4789

34. Izumi SS, Basin B, Presley M, et al. Feasibility and Acceptability of Nurse-Led Primary Palliative Care for Older Adults with Chronic Conditions: A Pilot Study. *J Palliat Med*. 2018;21(8):1114-1121. doi:10.1089/jpm.2017.0666

35. Jonsdottir H, Amundadottir OR, Gudmundsson G, et al. Effectiveness of a partnership-based self-management programme for patients with mild and moderate chronic obstructive pulmonary disease: A pragmatic randomized controlled trial. *J Adv Nurs*. 2015;71(11):2634-2649. doi:10.1111/jan.12728

36. Kass NE, Sugarman J, Medley AM, et al. An intervention to improve cancer patients' understanding of early-phase clinical trials. *IRB Ethics Hum Res*. 2009;31(3):1-8.

37. Kennedy A, Bower P, Reeves D, et al. Implementation of self management support for long term conditions in routine primary care settings: Cluster randomised controlled trial. *BMJ*. 2013;346(7913):1-11. doi:10.1136/bmj.f2882

38. Koerner M, Wirtz M, Michaelis M, et al. A multicentre cluster-randomized controlled study to evaluate a train-the-trainer programme for implementing internal and external participation in medical rehabilitation. *Clin Rehabil*. 2014;28(1):20-35. doi:10.1177/0269215513494874

39. Krug K, Bossert J, Deis N, et al. Effects of an Interprofessional Communication Approach on Support Needs, Quality of Life, and Mood of Patients with Advanced Lung Cancer: A Randomized Trial. *Oncologist*. 2021;26(8):e1445-e1459. doi:10.1002/ONCO.13790

40. Lainscak M, Kadivec S, Kosnik M, et al. Discharge coordinator intervention prevents

For peer review only - http://bmjopen.bmj.com/site/about/guidelines.xhtml

hospitalizations in patients with COPD: A randomized controlled trial. *J Am Med Dir Assoc*. 2013;14(6):450.e1-450.e6. doi:10.1016/j.jamda.2013.03.003

41. Latimer AE, Williams-Piehota P, Cox A, Katulak NA, Salovey P, Mowad L. Encouraging cancer patients to talk to their physicians about clinical trials: Considering patients' information needs. *J Appl Biobehav Res*. 2007;12(3-4):178-195. doi:10.1111/j.1751-9861.2008.00020.x

42. Lavoie KL, Moullec G, Lemiere C, et al. Efficacy of brief motivational interviewing to improve adherence to inhaled corticosteroids among adult asthmatics: Results from a randomized controlled pilot feasibility trial. *Patient Prefer Adherence*. 2014;8:1555-1569. doi:10.2147/PPA.S66966

43. Leung JM, Udris EM, Uman J, Au DH. The effect of end-of-life discussions on perceived quality of care and health status among patients with COPD. *Chest*. 2012;142(1):128-133. doi:10.1378/chest.11-2222

44. Lim RK, Semitala FC, Atuhumuza E, et al. Patient choice improves self-efficacy and intention to complete tuberculosis preventive therapy in a routine HIV program setting in Uganda. *PLoS One*. 2021;16(2):e0246113. doi:10.1371/JOURNAL.PONE.0246113

45. Llanos-Paez C, Ambery C, Yang S, et al. Improved Decision-Making Confidence Using Item-Based Pharmacometric Model: Illustration with a Phase II Placebo-Controlled Trial. *AAPS J*. 2021;23(4). doi:10.1208/s12248-021-00600-1

46. Lopez-Lopez L, Valenza MC, Rodriguez-Torres J, Torres-Sanchez I, Granados-Santiago M, Valenza-Demet G. Results on health-related quality of life and functionality of a patient-centered self-management program in hospitalized COPD: a randomized control trial. *Disabil Rehabil*. 2020;42(25):3687-3695. doi:10.1080/09638288.2019.1609099

47. Ludden T, Shade L, Reeves K, et al. Asthma dissemination around patient-centered treatments in North Carolina (ADAPT-NC): a cluster randomized control trial evaluating dissemination of an evidence-based shared decision-making intervention for asthma management. *J Asthma*. September 2018:1-12. doi:10.1080/02770903.2018.1514630

48. Makimoto G, Hotta K, Oze I, et al. Patients' preferences and perceptions of lung cancer treatment decision making: results from Okayama lung cancer study group trial 1406. *Acta Oncol (Madr)*. 2020;59(3):324-328. doi:10.1080/0284186X.2019.1679880

49. Markman M, Petersen J, Montgomery R. An examination of characteristics of lung and colon cancer patients participating in a web-based decision support program: Internet-based

For peer review only - http://bmjopen.bmj.com/site/about/guidelines.xhtml

decision support programs. *Oncology*. 2005;69(4):311-316. doi:10.1159/000089763

50. Markun S, Rosemann T, Dalla-Lana K, Steurer-Stey C. Care in Chronic Obstructive Lung Disease (CAROL): A randomised trial in general practice. *Eur Respir J*. 2018;51(5). doi:10.1183/13993003.01873-2017

51. Mayer DK, Deal AM, Crane JM, et al. Using survivorship care plans to enhance communication and cancer care coordination: Results of a pilot study. *Oncol Nurs Forum*. 2016;43(5):636-645. doi:10.1188/16.ONF.636-645

52. Meline J, Prigge JM, Dye D, et al. Adapting the design of a Web-based decision support clinical trial during the COVID-19 pandemic. *Trials*. 2021;22(1):1-6. doi:10.1186/S13063-021-05700-Z/PEER-REVIEW

53. Melzer AC, Clothier BA, Japuntich SJ, et al. Comparative effectiveness of proactive tobacco treatment among smokers with and without chronic lower respiratory disease. *Ann Am Thorac Soc*. 2018;15(3):341-347. doi:10.1513/AnnalsATS.201707-582OC

54. Menon S, Mccullough LB, Beyth RJ, Ford ME, Espadas D, Braun UK. Use of a values inventory as a discussion aid about end-of-life care: A pilot randomized controlled trial. *Palliat Support Care*. 2016;14(4):330-340. doi:10.1017/S1478951515001091

55. Meropol NJ, Egleston BL, Buzaglo JS, et al. A Web-based communication aid for patients with cancer: The CONNECT Study. *Cancer*. 2013;119(7):1437-1445. doi:10.1002/cncr.27874

56. Moullec G, Lavoie KL, Rabhi K, Julien M, Favreau H, Labrecque M. Effect of an integrated care programme on re-hospitalization of patients with chronic obstructive pulmonary disease. *Respirology*. 2012;17(4):707-714. doi:10.1111/j.1440-1843.2012.02168.x

57. Murray MA, Stacey D, Wilson KG, O'Connor AM. Skills training to support patients considering place of end-of-life care: A randomized control trial. *J Palliat Care*. 2010;26(2):112-121. doi:10.1177/082585971002600207

58. Nedjat-Haiem FR, Carrion I V., Gonzalez K, et al. Implementing an Advance Care Planning Intervention in Community Settings with Older Latinos: A Feasibility Study. *J Palliat Med*. 2017;20(9):984-993. doi:10.1089/jpm.2016.0504

59. Nugent SM, Golden SE, Thomas CR, et al. Patient-clinician communication among patients with stage I lung cancer. *Support Care Cancer*. 2018;26(5):1625-1633. doi:10.1007/s00520-017-3992-1

For peer review only

For peer review only - http://bmjopen.bmj.com/site/about/guidelines.xhtml

60. Patel MR, Smith A, Leo H, Hao W, Zheng K. Improving Patient–Provider Communication and Therapeutic Practice Through Better Integration of Electronic Health Records in the Exam Room: A Pilot Study. *Heal Educ Behav*. 2019;46(3):484-493. doi:10.1177/1090198118796879

61. Rabow MW, Dibble SL, Pantilat SZ, McPhee SJ. The Comprehensive Care Team. *Arch Intern Med*. 2004;164(1):83. doi:10.1001/archinte.164.1.83

62. Rea H, McAuley S, Stewart A, Lamont C, Roseman P, Didsbury P. A chronic disease management programme can reduce days in hospital for patients with chronic obstructive pulmonary disease. *Intern Med J*. 2004;34(11):608-614. doi:10.1111/j.1445-5994.2004.00672.x

63. Reddel HK, Correll J, Foster J, Karlsson N, Martin M, Keen C. Patient Decision-Making Around Use of Reliever Inhalers in Mild Asthma. In: *American Thoracic Society International Conference Meetings Abstracts*. American Thoracic Society; 2019:A5935-A5935. doi:10.1164/ajrccm-conference.2019.199.1_meetingabstracts.a5935

64. Reinke LF, Feemster LC, McDowell J, et al. The long term impact of an end-of-life communication intervention among veterans with COPD. *Hear Lung J Acute Crit Care*. 2017;46(1):30-34. doi:10.1016/j.hrtlng.2016.10.003

65. Rhee H, Hollen PJ, Belyea MJ, Sutherland MA. Decision-Making Program for Rural Adolescents With Asthma: A Pilot Study. *J Pediatr Nurs*. 2008;23(6):439-450. doi:10.1016/j.pedn.2008.01.079

66. Roelands M, Van Oost P, Stevens V, Depoorter AM, Buysse A. Clinical practice guidelines to improve shared decision-making about assistive device use in home care: A pilot intervention study. *Patient Educ Couns*. 2004;55(2):252-264. doi:10.1016/j.pec.2003.10.002

67. Rosemann T. The Improving Care in Chronic Obstructive Lung Disease Study A Cluster Randomized Trial. *https://clinicaltrials.gov/show/NCT01921556*. 2013. doi:10.1002/CENTRAL/CN-02032168

68. Ruparel M, Quaife SL, Ghimire B, et al. Impact of a lung cancer screening information film on informed decision-making: A randomized trial. *Ann Am Thorac Soc*. 2019;16(6):744-751. doi:10.1513/AnnalsATS.201811-841OC

69. Rutten-vanMolken M, Goossens L, Boland M, et al. The Assessment of Burden of COPD (ABC) tool: a shared decision-making instrument that is predictive of healthcare costs. *Int J Integr Care*. 2017;17(5):320. doi:10.5334/ijic.3637

For peer review only - http://bmjopen.bmj.com/site/about/guidelines.xhtml

70. Sands M. A Randomised Control Trial for Advance Care Planning and Symptom Management for patients identified in the emergency department and followed up at home. *Clin Regist Clin Trials*. 2014. doi:10.1002/CENTRAL/CN-01881924

71. Schofield P, Ugalde A, Gough K, et al. A tailored, supportive care intervention using systematic assessment designed for people with inoperable lung cancer: A randomised controlled trial. *Psychooncology*. 2013;22(11):2445-2453. doi:10.1002/pon.3306

72. Schubart JR, Green MJ, Van Scoy LJ, et al. Advanced Cancer and End-of-Life Preferences: Curative Intent Surgery Versus Noncurative Intent Treatment. *J Palliat Med*. 2015;18(12):1015-1018. doi:10.1089/jpm.2015.0021

73. Schumacher KL, Koresawa S, West C, et al. Putting cancer pain management regimens into practice at home. *J Pain Symptom Manage*. 2002;23(5):369-382. doi:10.1016/S0885-3924(02)00385-8

74. Schwarze ML, Buffington A, Tucholka JL, et al. Effectiveness of a Question Prompt List Intervention for Older Patients Considering Major Surgery: A Multisite Randomized Clinical Trial. *JAMA Surg*. 2020;155(1):6-13. doi:10.1001/jamasurg.2019.3778

75. Ludden T, Shade L, Reeves K, et al. Asthma dissemination around patient-centered treatments in North Carolina (ADAPT-NC): a cluster randomized control trial evaluating dissemination of an evidence-based shared decision-making intervention for asthma management. *J Asthma*. 2019;56(10):1087-1098. doi:10.1080/02770903.2018.1514630

76. Shirai Y, Fujimori M, Ogawa A, et al. Patients' perception of the usefulness of a question prompt sheet for advanced cancer patients when deciding the initial treatment: A randomized, controlled trial. *Psychooncology*. 2012;21(7):706-713. doi:10.1002/pon.1955

77. Sinclair C, Auret KA, Evans SF, et al. Advance care planning uptake among patients with severe lung disease: A randomised patient preference trial of a nurse-led, facilitated advance care planning intervention. *BMJ Open*. 2017;7(2):1-11. doi:10.1136/bmjopen-2016-013415

78. Skorstengaard MH, Brogaard T, Bonde Jensen A, et al. Advance care planning for patients and their relatives. *Int J Palliat Nurs*. 2019;25(3):112-127. doi:10.12968/ijpn.2019.25.3.112

79. Slok A, Kotz D, van Breukelen G, et al. The assessment of burden of COPD tool improves health related quality of life. In: *European Respiratory Journal*. Vol 48. European Respiratory Society (ERS); 2016:OA1997. doi:10.1183/13993003.congress-2016.oa1997

80. Slok AHM, Kotz D, Van Breukelen G, et al. Effectiveness of the Assessment of Burden of COPD

For peer review only - http://bmjopen.bmj.com/site/about/guidelines.xhtml

(ABC) tool on health-related quality of life in patients with COPD: A cluster randomised controlled trial in primary and hospital care. *BMJ Open*. 2016;6(7). doi:10.1136/bmjopen-2016-011519

81. Smidth M, Olesen F, Fenger-Grøn M, Vedsted P. Patient-experienced effect of an active implementation of a disease management programme for COPD - A randomised trial. *BMC Fam Pract*. 2013;14(1):1. doi:10.1186/1471-2296-14-147

82. Steinhauser KE, Alexander SC, Byock IR, George LK, Olsen MK, Tulsky JA. Do preparation and life completion discussions improve functioning and quality of life in seriously ill patients? Pilot randomized control trial. *J Palliat Med*. 2008;11(9):1234-1240. doi:10.1089/jpm.2008.0078

83. Steurer C, Markun S, Rosemann T, Dalla Lana K. Improving Care in Chronic Obstructive Lung Disease (CAROL): a cluster randomized trial in primary care. In: European Respiratory Society (ERS); 2017:OA2915. doi:10.1183/1393003.congress-2017.oa2915

84. Steurer-Stey C, Storch M, Benz S, et al. Motivational training improves self-efficacy but not short-term adherence with asthma self-management: a randomized controlled trial. *Prim Health Care Res Dev*. 2015;16(1):32-41. doi:10.1017/S1463423613000480

85. Sullivan DR, Slatore CG, Stone K, et al. Associations Between Treatment and Team Composition with Decisional Regret Among Patients with Early Stage Lung Cancer. May 2020:A4726-A4726. doi:10.1164/AJRCCM-CONFERENCE.2020.201.1_MEETINGABSTRACTS.A4726

86. Tai-Seale M, Elwyn G, Wilson CJ, et al. Enhancing shared decision making through carefully designed interventions that target patient and provider behavior. *Health Aff*. 2016;35(4):605-612. doi:10.1377/hlthaff.2015.1398

87. Tamblyn R. Computer-Based Decision Support in Managing Asthma in Primary Care. *Clin Regist Clin Trials*. 2005. doi:10.1002/CENTRAL/CN-02024751

88. Tamblyn R, Ernst P, Winslade N, et al. Evaluating the impact of an integrated computer-based decision support with person-centered analytics for the management of asthma in primary care: A randomized controlled trial. *J Am Med Informatics Assoc*. 2015;22(4):773-783. doi:10.1093/jamia/ocu009

89. Tang JI, Shakespeare TP, Lu JJ, et al. Patients' preference for radiotherapy fractionation schedule in the palliation of symptomatic unresectable lung cancer. *J Med Imaging Radiat*

For peer review only - http://bmjopen.bmj.com/site/about/guidelines.xhtml

Oncol. 2008;52(5):497-502. doi:10.1111/j.1440-1673.2008.02002.x

90. Tapp H, Shade L, Mahabaleshwarkar R, Taylor YJ, Ludden T, Dulin MF. Results from a pragmatic prospective cohort study: Shared decision making improves outcomes for children with asthma. *J Asthma*. 2017;54(4):392-402. doi:10.1080/02770903.2016.1227333

91. Thee S, Stahl M, Fischer R, et al. A multi-centre, randomized, controlled trial on coaching and telemonitoring in patients with cystic fibrosis: conneCT CF. *BMC Pulm Med*. 2021;21(1):1-11. doi:10.1186/S12890-021-01500-Y/TABLES/1

92. Thiam S, LeFevre AM, Hane F, et al. Effectiveness of a strategy to improve adherence to tuberculosis treatment in a resource-poor setting: A cluster randomized controlled trial. *J Am Med Assoc*. 2007;297(4):380-386. doi:10.1001/jama.297.4.380

93. Totten A. Team-based Versus Primary Care Clinician-led Advance Care Planning in Practice-based Research Networks. *Clin Regist Clin Trials*. 2018. doi:10.1002/CENTRAL/CN-01660857

94. Tsao S, Willard-Grace R, Wolf J, et al. Implementation and Impact of the Pulmonary Specialist Health Coach Consultation Model to Improve Care for Patients with COPD. *Jt Comm J Qual patient Saf*. 2021;47(11):739-747. doi:10.1016/J.JCJQ.2021.08.003

95. Vandemheen KL, O'Connor A, Bell SC, et al. Randomized trial of a decision aid for patients with cystic fibrosis considering lung transplantation. *Am J Respir Crit Care Med*. 2009;180(8):761-768. doi:10.1164/rccm.200903-0421OC

96. Wahlberg H, Braaten T, Broderstad AR. Impact of referral templates on patient experience of the referral and care process: A cluster randomised trial. *BMJ Open*. 2016;6(10):1-8. doi:10.1136/bmjopen-2016-011651

97. Waller A, Girgis A, Johnson C, et al. Improving outcomes for people with progressive cancer: Interrupted time series trial of a needs assessment intervention. *J Pain Symptom Manage*. 2012;43(3):569-581. doi:10.1016/j.jpainsymman.2011.04.020

98. Wang KY, Chian CF, Lai HR, Tarn YH, Wu CP. Clinical pharmacist counseling improves outcomes for Taiwanese asthma patients. *Pharm World Sci*. 2010;32(6):721-729. doi:10.1007/s11096-010-9427-4

99. Weekes CE, Emery PW, Elia M. Dietary counselling and food fortification in stable copd: A randomised trial. *Thorax*. 2009;64(4):326-331. doi:10.1136/thx.2008.097352

100. Williams PD, Graham KM, Storlie DL, et al. Therapy-related symptom checklist use during

For peer review only - http://bmjopen.bmj.com/site/about/guidelines.xhtml

treatments at a cancer center. *Cancer Nurs*. 2013;36(3):245-254. doi:10.1097/NCC.0b013e3182595406

101. Wilson SR, Strub P, Knowles SB, Buist AS, Huang Q, Nguyen M. Ethnicity, Income, and Education as Potential Modifiers of the Effects of Shared Treatment Decision-Making (SDM) Between Asthma Care Manager and Patient on Asthma Controller Medication Adherence: The Better Outcomes of Asthma Treatment (BOAT) Trial. *J Allergy Clin Immunol*. 2009;123(2):S72-S72. doi:10.1016/j.jaci.2008.12.247

102. Wilson S. Does Shared Decision-Making Improve Asthma Outcomes? *Clin Regist Clin Trials*. 2005. doi:10.1002/CENTRAL/CN-02031001

103. Wilson S. Does shared decision-making improve adherence in asthma. *Clin Regist Clin Trials*. 2005. doi:10.1002/CENTRAL/CN-01427587

104. Wood-baker R, Reid D, Robinson A. Wood-Baker et al: Clinical trial of community nurse mentoring to improve self-management in patients with chronic obstructive pulmonary disease. International Journal of COPD 2012, 7:407-13. 2012:407-413. http://www.pubmedcentral.nih.gov/articlerender.fcgi?artid=3402057&tool=pmcentrez&rendertype=abstract.

105. Yoo SH, Yun YH, Kim KN, et al. The impact of caregiver's role preference on decisional conflicts and psychiatric distresses in decision making to help caregiver's disclosure of terminal disease status. *Qual Life Res*. 2018;27(6):1571-1581. doi:10.1007/s11136-018-1814-7

106. Zakrisson AB, Arne M, Hasselgren M, Lisspers K, Ställberg B, Theander K. A complex intervention of self-management for patients with COPD or CHF in primary care improved performance and satisfaction with regard to own selected activities; A longitudinal follow-up. *J Adv Nurs*. 2019;75(1):175-186. doi:10.1111/jan.13899

107. Zanartu CP, Camacho FJ, Nevadunski NS, et al. Speaking the same language: a feasibility trial for a novel visual communication tool for oncologist-patient treatment discussions. *Psychooncology*. 2017;26(7):1050-1052. doi:10.1002/pon.4246

108. Au DH, Udris EM, Engelberg RA, et al. A randomized trial to improve communication about end-of-life care among patients with COPD. *Chest*. 2012;141(3):726-735. doi:10.1378/chest.11-0362

109. Gagné ME, Légaré F, Moisan J, Boulet L-P. Impact of Adding a Decision Aid to Patient

For peer review only - http://bmjopen.bmj.com/site/about/guidelines.xhtml

Education in Adults with Asthma: A Randomized Clinical Trial. Leroyer C, ed. *PLoS One*. 2017;12(1):e0170055. doi:10.1371/journal.pone.0170055

110. Collinsworth AW, Brown RM, Sjames C, stanford RH, Alemayehu D, Priest EL. The impact of patient education and shared decision making on hospital readmissions for COPD. *Int J COPD*. 2018;13:1325-1332. doi:10.2147/COPD.S154414

111. Granados-Santiago M, Valenza MC, López-López L, Prados-Román E, Rodríguez-Torres J, Cabrera-Martos I. Shared decision-making and patient engagement program during acute exacerbation of COPD hospitalization: A randomized control trial. *Patient Educ Couns*. 2019;103(4):702-708. doi:10.1016/j.pec.2019.12.004

112. Brundage MD, Feldman-Stewart D, Cosby R, et al. Phase I study of a decision aid for patients with locally advanced non-small-cell lung cancer. *J Clin Oncol*. 2001;19(5):1326-1335. doi:10.1200/JCO.2001.19.5.1326

113. Myers RE, Advani SM, Myers P, et al. Engaging Patients with Late-Stage Non-Small Cell Lung Cancer in Shared Decision Making about Treatment. *J Pers Med*. 2021;11(10). doi:10.3390/JPM11100998

114. Walters J, Cameron-Tucker H, Wills K, et al. Effects of telephone health mentoring in community-recruited chronic obstructive pulmonary disease on self-management capacity, quality of life and psychological morbidity: A randomised controlled trial. *BMJ Open*. 2013;3(9). doi:10.1136/bmjopen-2013-003097

115. Wilson SR, Strub P, Buist AS, et al. Shared treatment decision making improves adherence and outcomes in poorly controlled asthma. *Am J Respir Crit Care Med*. 2010;181(6):566-577. doi:10.1164/rccm.200906-0907OC

For peer review only

For peer review only - http://bmjopen.bmj.com/site/about/guidelines.xhtml

PRISMA 2020 Checklist

Section and Topic	Item #	Checklist item	Location where item is reported
**TITLE**			
Title	1	Identify the report as a systematic review.	Title, pg1
**ABSTRACT**			
Abstract	2	See the PRISMA 2020 for Abstracts checklist.	Abstract, pg3-4
**INTRODUCTION**			
Rationale	3	Describe the rationale for the review in the context of existing knowledge.	Introduction, pg6-8
Objectives	4	Provide an explicit statement of the objective(s) or question(s) the review addresses.	Introduction, pg8
**METHODS**			
Eligibility criteria	5	Specify the inclusion and exclusion criteria for the review and how studies were grouped for the syntheses.	Methods, pg8-9
Information sources	6	Specify all databases, registers, websites, organisations, reference lists and other sources searched or consulted to identify studies. Specify the date when each source was last searched or consulted.	Methods, pg9
Search strategy	7	Present the full search strategies for all databases, registers and websites, including any filters and limits used.	Methods, pg10 & Supplementary Material
Selection process	8	Specify the methods used to decide whether a study met the inclusion criteria of the review, including how many reviewers screened each record and each report retrieved, whether they worked independently, and if applicable, details of automation tools used in the process.	Methods, pg10
Data collection process	9	Specify the methods used to collect data from reports, including how many reviewers collected data from each report, whether they worked independently, any processes for obtaining or confirming data from study investigators, and if applicable, details of automation tools used in the process.	Methods, pg10
Data items	10a	List and define all outcomes for which data were sought. Specify whether all results that were compatible with each outcome domain in each study were sought (e.g. for all measures, time points, analyses), and if not, the methods used to decide which results to collect.	Supplementary Material
	10b	List and define all other variables for which data were sought (e.g. participant and intervention characteristics, funding sources). Describe any assumptions made about any missing or unclear information.	Supplementary Material
Study risk of bias assessment	11	Specify the methods used to assess risk of bias in the included studies, including details of the tool(s) used, how many reviewers assessed each study and whether they worked independently, and if applicable, details of automation tools used in the process.	Methods, pg10-11
Effect measures	12	Specify for each outcome the effect measure(s) (e.g. risk ratio, mean difference) used in the synthesis or presentation of results.	Methods, pg11
Synthesis methods	13a	Describe the processes used to decide which studies were eligible for each synthesis (e.g. tabulating the study intervention characteristics and comparing against the planned groups for each synthesis (item #5)).	Methods, pg11
	13b	Describe any methods required to prepare the data for presentation or synthesis, such as handling of missing summary statistics, or data conversions.	Methods, pg11
	13c	Describe any methods used to tabulate or visually display results of individual studies and syntheses.	Methods, pg11
	13d	Describe any methods used to synthesize results and provide a rationale for the choice(s). If meta-analysis was performed, describe the model(s), method(s) to identify the presence and extent of statistical heterogeneity, and software package(s) used.	Methods, pg11

For peer review only - http://bmjopen.bmj.com/site/about/guidelines.xhtml

PRISMA 2020 Checklist

Section and Topic	Item #	Checklist item	Location where item is reported
	13e	Describe any methods used to explore possible causes of heterogeneity among study results (e.g. subgroup analysis, meta-regression).	Methods, pg11
	13f	Describe any sensitivity analyses conducted to assess robustness of the synthesized results.	N/A
Reporting bias assessment	14	Describe any methods used to assess risk of bias due to missing results in a synthesis (arising from reporting biases).	Methods, pg10-11
Certainty assessment	15	Describe any methods used to assess certainty (or confidence) in the body of evidence for an outcome.	Methods, pg11
**RESULTS**			
Study selection	16a	Describe the results of the search and selection process, from the number of records identified in the search to the number of studies included in the review, ideally using a flow diagram.	Results, pg11-12, Figure II
	16b	Cite studies that might appear to meet the inclusion criteria, but which were excluded, and explain why they were excluded.	Results, pg11-12, Figure II, Supplementary Material
Study characteristics	17	Cite each included study and present its characteristics.	Results, pg12, Tables I and II
Risk of bias in studies	18	Present assessments of risk of bias for each included study.	Results, pg12, Figure IV and V
Results of individual studies	19	For all outcomes, present, for each study: (a) summary statistics for each group (where appropriate) and (b) an effect estimate and its precision (e.g. confidence/credible interval), ideally using structured tables or plots.	Table I
Results of syntheses	20a	For each synthesis, briefly summarise the characteristics and risk of bias among contributing studies.	Results, pg12, Table I, Figure IV and V
	20b	Present results of all statistical syntheses conducted. If meta-analysis was done, present for each the summary estimate and its precision (e.g. confidence/credible interval) and measures of statistical heterogeneity. If comparing groups, describe the direction of the effect.	Results, pg13-16, Table I
	20c	Present results of all investigations of possible causes of heterogeneity among study results.	Supplementary Material
	20d	Present results of all sensitivity analyses conducted to assess the robustness of the synthesized results.	N/A
Reporting biases	21	Present assessments of risk of bias due to missing results (arising from reporting biases) for each synthesis assessed.	Results, pg12, Figure IV and V
Certainty of evidence	22	Present assessments of certainty (or confidence) in the body of evidence for each outcome assessed.	Table I
**DISCUSSION**			
Discussion	23a	Provide a general interpretation of the results in the context of other evidence.	Discussion, pg16-18
	23b	Discuss any limitations of the evidence included in the review.	Discussion, pg18

For peer review only

PRISMA 2020 Checklist

Section and Topic	Item #	Checklist item	Location where item is reported
	23c	Discuss any limitations of the review processes used.	Discussion, pg18-19
	23d	Discuss implications of the results for practice, policy, and future research.	Discussion, pg19
**OTHER INFORMATION**			
Registration and protocol	24a	Provide registration information for the review, including register name and registration number, or state that the review was not registered.	Other information, pg20
	24b	Indicate where the review protocol can be accessed, or state that a protocol was not prepared.	Other information, pg20
	24c	Describe and explain any amendments to information provided at registration or in the protocol.	N/A
Support	25	Describe sources of financial or non-financial support for the review, and the role of the funders or sponsors in the review.	Other information, pg20
Competing interests	26	Declare any competing interests of review authors.	Other information, pg20
Availability of data, code and other materials	27	Report which of the following are publicly available and where they can be found: template data collection forms; data extracted from included studies; data used for all analyses; analytic code; any other materials used in the review.	Other information, pg20

From: Page MJ, McKenzie JE, Bossuyt PM, Boutron I, Hoffmann TC, Mulrow CD, et al. The PRISMA 2020 statement: an updated guideline for reporting systematic reviews. BMJ 2021;372:n71. doi: 10.1136/bmj.n71

For more information, visit: http://www.prisma-statement.org/

For peer review only

Synthesis Without Meta-analysis (SWiM) reporting items

The citation for the Synthesis Without Meta-analysis explanation and elaboration article is: Campbell M, McKenzie JE, Sowden A, Katikireddi SV, Brennan SE, Ellis S, Hartmann-Boyce J, Ryan R, Shepperd S, Thomas J, Welch V, Thomson H. Synthesis without meta-analysis (SWiM) in systematic reviews: reporting guideline BMJ 2020;368:l6890 http://dx.doi.org/10.1136/bmj.l6890

**SWiM is intended to complement and be used as an extension to PRISMA**			
**SWiM reporting item**	**Item description**	**Page in manuscript where item is reported**	**Other***
Methods			
**1** Grouping studies for synthesis	1a) Provide a description of, and rationale for, the groups used in the synthesis (e.g., groupings of populations, interventions, outcomes, study design)	Lines 168-192	PROSPERO: CRD42020169897
	1b) Detail and provide rationale for any changes made subsequent to the protocol in the groups used in the synthesis	N/A	
**2** Describe the standardised metric and transformation methods used	Describe the standardised metric for each outcome. Explain why the metric(s) was chosen, and describe any methods used to transform the intervention effects, as reported in the study, to the standardised metric, citing any methodological guidance consulted	Lines 243-248	
**3** Describe the synthesis methods	Describe and justify the methods used to synthesise the effects for each outcome when it was not possible to undertake a meta-analysis of effect estimates	Lines 237-242	
**4** Criteria used to prioritise results for summary and synthesis	Where applicable, provide the criteria used, with supporting justification, to select the particular studies, or a particular study, for the main synthesis or to draw conclusions from the synthesis (e.g., based on study design, risk of bias assessments, directness in relation to the review question)	Lines 238-241	PROSPERO: CRD42020169897

For peer review only - http://bmjopen.bmj.com/site/about/guidelines.xhtml

Synthesis Without Meta-analysis (SWiM) reporting items

**SWiM reporting item**	**Item description**	**Page in manuscript where item is reported**	**Other***
**5** Investigation of heterogeneity in reported effects	State the method(s) used to examine heterogeneity in reported effects when it was not possible to undertake a meta-analysis of effect estimates and its extensions to investigate heterogeneity	Lines 237-241	
**6** Certainty of evidence	Describe the methods used to assess certainty of the synthesis findings	Lines 229-235	
**7** Data presentation methods	Describe the graphical and tabular methods used to present the effects (e.g., tables, forest plots, harvest plots).<br><br>Specify key study characteristics (e.g., study design, risk of bias) used to order the studies, in the text and any tables or graphs, clearly referencing the studies included	Lines 243-244	
Results			
**8** Reporting results	For each comparison and outcome, provide a description of the synthesised findings, and the certainty of the findings. Describe the result in language that is consistent with the question the synthesis addresses, and indicate which studies contribute to the synthesis	Lines 280-370 and Table 1	
Discussion			
**9** Limitations of the synthesis	Report the limitations of the synthesis methods used and/or the groupings used in the synthesis, and how these affect the conclusions that can be drawn in relation to the original review question	Lines 422-444	

For peer review only - http://bmjopen.bmj.com/site/about/guidelines.xhtml

Synthesis Without Meta-analysis (SWiM) reporting items

PRISMA=Preferred Reporting Items for Systematic Reviews and Meta-Analyses.
*If the information is not provided in the systematic review, give details of where this information is available (e.g., protocol, other published papers (provide citation details), or website (provide the URL)).

For peer review only
